# Integrated pituitary adaptations to the low estrogen state

**DOI:** 10.3389/fendo.2026.1920998

**Published:** 2026-10-05

**Authors:** Tiffany K. Miles, Angela K Odle, Ashley K. Herdman, Lillian G. Clark, Stephanie D. Byrum, Alex Lagasse, Anessa Haney, Angus M. MacNicol, Gwen V. Childs, Melanie C. MacNicol

**Affiliations:** 1 College of Medicine, Department of Neuroscience, University of Arkansas for Medical Science, Little Rock, AR, United States; 2 College of Medicine, Arkansas Children’s Research Institute, University of Arkansas for Medical Science, Little Rock, AR, United States; 3 College of Medicine, Department of Biochemistry and Molecular Biology, University of Arkansas for Medical Science, Little Rock, AR, United States; 4 College of Medicine, Department of Biomedical Informatics, University of Arkansas for Medical Science, Little Rock, AR, United States

**Keywords:** corticotropes, differential gene expression, lactotropes, menopause, pituitary, single-cell RNA-seq, somatotropes, thyrotropes

## Abstract

**Introduction:**

Estrogen’s broad trophic effects on anterior pituitary function are not fully understood. The compound 4-vinyl cyclohexene diepoxide (VCD) induces early ovarian failure and reduced E2 production, which may differentially affect anterior pituitary cells. We recently identified transcriptomic changes in pituitary gonadotropes at the single-cell level in females treated with VCD for 20 days, which were acyclic, with low serum estradiol (E2) levels and 8-12-fold increases in gonadotropins 138 days later.

**Materials and methods:**

We tested vehicle-control and VCD-treated female mice in metabolic cages and assayed serum hormone levels by EIA, and pituitary cell transcripts by qRT-PCR and single-cell RNA-seq.

**Results:**

VCD-treated mice showed normal metabolic health. Compared with controls, VCD-treated females exhibited a 5-fold elevation in serum pituitary thyroid-stimulating hormone (TSH) levels and near-normal thyroid hormone levels. Pituitary *Tshb* mRNA levels were reduced, and pituitary *Gh, Prl*, and *Pomc* mRNA levels were unchanged. Serum growth hormone (GH) levels were higher in VCD-treated mice than in controls 62 days after the start of treatment (perimenopause) and then reduced to control levels after 118–138 days. Ingenuity Pathway Analyses (IPA) of upregulated differentially expressed gene (DEG) transcripts predicted activation of G protein-mediated signaling pathways in one scRNA-seq somatotrope subcluster and in the thyrotrope and lactotrope clusters. In contrast, downregulated DEGs in corticotropes and the remaining two somatotrope clusters predicted downregulation of critical signaling pathways.

**Discussion:**

The unchanged pituitary *Gh, Prl*, and *Pomc* mRNA levels correlated with unchanged serum levels. The interim rise in serum GH 62 days after treatment agrees with reports of perimenopausal responses due to estrogen spikes. Reduced pituitary *Tshb* correlates with the recently reported reduced expression in gonadotropes. IPA analysis of upregulated DEGs in thyrotrope clusters predicted the activation of canonical signaling pathways that support elevated serum TSH levels. The near-normal thyroid hormone levels suggest subclinical hypothyroidism in menopausal women. Downregulated DEGs in corticotropes and in two somatotrope subclusters predicted compromised stress and metabolic responses. Finally, reduced multihormone gene expression suggested compromised plasticity in a low-estrogen environment. These different responses to a sustained low-estrogen state, such as that seen in menopause, reflect the diversity of estrogen’s trophic actions.

## 1 Introduction

Ovarian-derived estrogen (estradiol, E2) plays essential roles in regulating anterior pituitary cell functions by acting both directly on the pituitary and indirectly through hypothalamic neuronal actions. Well-characterized E2 targets include the pituitary gonadotropes, which produce luteinizing hormone (LH) and follicle-stimulating hormone (FSH). E2 exerts negative feedback on gonadotropes indirectly through the hypothalamic Kisspeptin-Gonadotropin-Releasing Hormone (GnRH) circuit during the luteal phase of the cycle (1, 2), and exerts a direct positive effect on both gonadotropes and hypothalamic GnRH neurons during the follicular phase of the female estrous/menstrual cycle, leading to the midcycle, pre-ovulatory LH surge (3–5). Similarly, pituitary lactotropes are stimulated by E2 to proliferate and produce and release prolactin (6)leading to the “prolactin surge” (6–8) that coincides with the midcycle rise in serum E2 (7, 9). Notably, the absence of this prolactin surge may serve as a marker for infertility (10).

The role of E2 in regulating other pituitary cell populations, including thyrotropes, corticotropes, and somatotropes, is less well characterized. E2 has been shown to directly stimulate thyroid-stimulating hormone (TSH) secretion from thyrotropes *in vivo* (11) and *in vitro* (12) and to promote expression of thyrotropin-releasing hormone (TRH) receptors (TRHR) on both thyrotropes and lactotropes, thereby increasing the sensitivity of both cell types to hypothalamic TRH (13). E2 also regulates corticotrope production of adrenocorticotropin (ACTH), and the response to corticotropin-releasing hormone (CRH) stimulation and increases corticotrope proliferation (14, 15). E2 has also been shown to directly stimulate somatotropes to produce growth hormone (GH) (16, 17) and to increase the expression of GH-releasing hormone receptors (GHRHR) (16, 17). E2 works with hypothalamic-derived pulses of GHRH to increase the amplitude of somatotrope GH pulses, thereby potentiating the GH surge during the reproductive cycle (18).

Consistent with E2’s broad role in mediating pituitary function, the significant 35-50% decline in E2 with aging in women (19–21) is linked to effects beyond the decline in reproductive functions (4, 22–28). Importantly, in menopausal women (29, 30), reduced E2 correlates with a decline in serum GH (20, 31, 32), specifically due to reduced GH pulse amplitude (33). Pituitary corticotropes are part of the stress response to hormone fluctuations and hot flashes, which can worsen mood swings, and insomnia (34). Thyroid diseases are also more prevalent during menopause, affecting the pituitary thyroid-stimulating hormone (TSH)-secreting thyrotropes (11, 21, 30, 35–39). Indeed, subclinical hypothyroidism, which is distinguished by elevated levels of TSH but normal levels of thyroid hormone has been found to be associated with dyslipidemia in perimenopausal and menopausal females (38). Insight into the mechanisms underlying the pituitary response to sustained low E2 would assist in our understanding of the wide-ranging effects of menopause.

The compound 4-vinyl cyclohexene diepoxide (VCD) induces early ovarian failure and reduced E2 production in adult female mice (40, 41) and has been used as a menopause model (41–43). We have recently used this VCD model to identify transcriptomic changes in gonadotropes at the single-cell level and showed differentially expressed genes and signaling pathways in both pituitary gonadotrope and stem cell clusters (44). In the present study, we have extended our analyses of this model and tested the hypothesis that sustained low E2 would differentially affect function in pituitary somatotropes, lactotropes, thyrotropes and corticotropes. We identified transcriptomic changes in gene expression and through Ingenuity Pathway Analysis, explored the potential implications of the differentially expressed genes (DEGs) with respect to changes in upstream regulators and canonical signaling pathways in each of the cell types. These findings support our hypothesis about the differential responses by the pituitary cell types, and also suggest additional hypotheses about potential mechanisms underlying their responses to the sustained low-estrogen state, as seen in menopause.

## 2 Materials and methods

### 2.1 Animal handling and VCD treatment

The University of Arkansas for Medical Sciences (UAMS) Institutional Animal Care and Use Committee approved animal protocols, and the UAMS Biosafety Committee approved our use and handling of 4-vinylcyclohexene diepoxide (VCD). We used the hybrid strain of mice, FVB.129P2-Pde6b + Tyr c-ch/AntJ (43). We divided 28 3-4-month-old female mice into two groups: VCD-treated and vehicle-treated controls. We induced ovarian failure by treating 14 of the mice with VCD following well-established rodent protocols (35, 38, 39, 44). Mice were injected intraperitoneally (IP) daily with VCD in sesame oil for 20 days (160 mg.kg-1.day-1), which results in the selective loss of all primary and primordial follicles during the subsequent 15 days. Vehicle-treated control females received sesame oil only.

The age, time of euthanasia, and treatment group for each of the 50 mice is listed in **Supplementary Table S1** in our recent publication (44). Before we remove the pituitaries, the mice are anesthetized and then euthanized as follows. As per our approved animal use protocol, the mice are removed one at a time from their cages, weighted and placed individually in a bell jar. Filter paper soaked with 1-5% isoflurane is placed under a platform in the bell jar. The mice fall asleep in the platform in the jar within 2 minutes after exposure to the isoflurane. The platform prevents them from being in direct contact with the fluid from the isoflurane. After they are deeply asleep, the mice are decapitated with a guillotine within seconds of removal from their cage. The guillotine is used only for mice and thoroughly cleaned after use. It is sharpened and parts oiled regularly to maintain smooth operation. The pituitary is then removed from the head.

As reported in that Table and **Figure 1** (44), we determined that the VCD-treated mice were acyclic 120 days after the start of VCD. At the time of sacrifice (133–138 days after the start of VCD), serum levels of gonadotropins were elevated 15—fold (LH) and 8—fold (FSH) (42). One week prior to euthanasia, mice were transferred to Comprehensive Lab Animal Monitoring cages (CLAMS-Oxymax, Columbus Instruments) (45, 46) in a thermoneutral room to evaluate their metabolic health.

**Figure 1:**
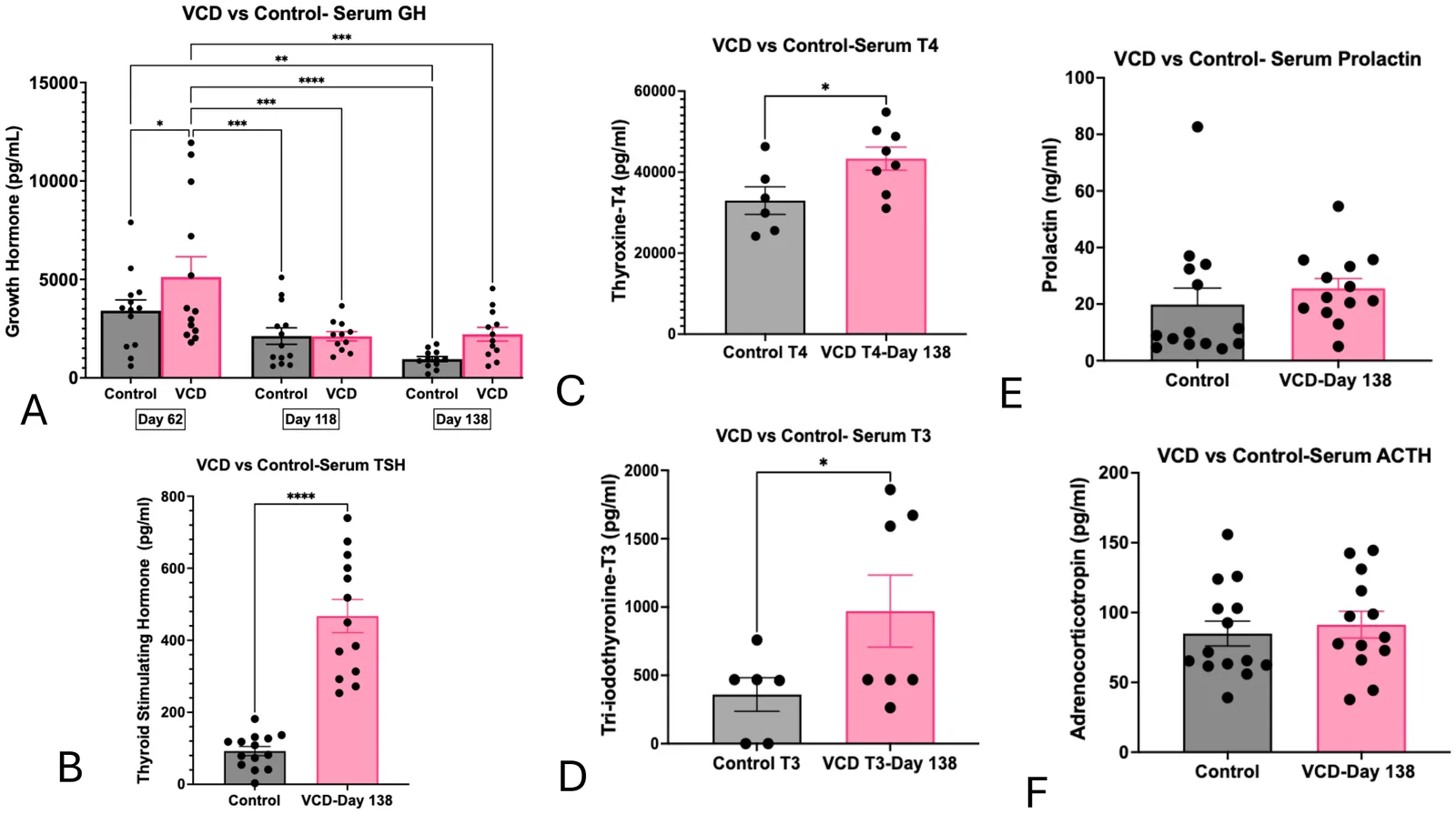
Serum levels of hormones comparing VCD-treated mice with controls. All serum samples were taken from mice 138 days following the start of VCD treatment. Additional serum samples for the GH assay were taken from tail snip serum 62 and 118 days after the beginning of VCD treatment. **(A)** Serum GH; **(B)** Serum TSH; **(C)** Serum T_4_; **(D)** Serum T_3_; **(E)** Serum Prolactin; **(F)** Serum ACTH. *=p<0.05; **=p<0.001; ***=p<0.0001, ****=p=0.0001.

On days 133-138 (with day 1 as the first day of VCD treatment), we collected pituitaries and dispersed the cells. Cells from six (6) pituitaries per group were used for single-cell RNA sequencing (scRNA-seq) (45–50). We created two pools per group, with cells from 3 animals per pool. Serum was collected from all animals and frozen for enzyme immunoassay (EIA). Parallel studies of cycling females from the same strain were used for final assays of serum hormone levels to supplement the Control female group, ensuring sufficient females per stage of the cycle.

### 2.2 Multiplex assays

To detect changes in serum levels of pituitary hormones (prolactin, GH, ACTH, TSH) and thyroid hormones (thyroxine [T4] and triiodothyronine [T3]), we used the Luminex 200 platform and Millipore Sigma Milliplex assay kits (51, 52). Specifically, we used the Mouse Pituitary Panel (MPTMAG-49K), RRID: AB_2811194 and Rat Thyroid Panel (RTHYMAG-30K), RRID: AB_2868475.

### 2.3 Quantitative real time polymerase chain reaction

cDNA was synthesized from whole pituitary mRNA (iScript, Bio-Rad, 170-8890) from control and VCD-treated females. qRT-PCR was performed using Power SYBR Green PCR Master Mix (Applied Biosystems, 4367659) and the QuantStudio 12K Flex system (Applied Biosystems, Life Technologies). Primer sequences were obtained from Luque et al. (53). Expression of each target was normalized to cyclophilin A (*Ppia*), and fold-change was quantified using the delta-delta-CT method as described (49).

### 2.3 Single-cell RNA sequencing and analysis

The protocol for pituitary dispersion and cell handling has been described in detail (46, 47). Two concurrently processed pituitary pools, representing 6 mice per experimental group, were submitted to the UAMS Flow Cytometry Core facility. Microscopic examination (EVOS M7000, Thermo Fisher Scientific) revealed cell debris or aggregates, which were removed by passing the cell suspensions through a Flowmi™ cell strainer (SP BEL-ART, catalog # 136800040, 40 microns). Following centrifugation at 250 g for 10 min, cells were resuspended in 1X Presuspended containing 0.04% (w/v) BSA. Cell concentration and viability were assessed with the ReadyProbes™ _Cell Viability Imaging Kit, Blue/Green (Thermo Fisher Scientific, catalog # R37609), and cells were manually counted. The analysis was performed on 8,861 cells, recovering 4717 from controls and 4144 from VCD-treated cells.

Single-cell 3′ library generation was performed using a 10x Genomics Chromium Controller and the Chromium Next GEM Single Cell 3′ Reagent Kits v3.1 (dual index), as previously described (46). Libraries were sequenced on the Illumina NovaSeq 6000 platform in paired-end mode (read 1: 28 cycles, read 2: 90 cycles, i7: 10 cycles, i5: 10 cycles) to generate a minimum of 20,000 read pairs per cell. The 10x Genomics Cell Ranger 7.1 *mkfastq* wrapper was used to perform sample demultiplexing and generate fastq files.

We performed bioinformatics clustering, quality control, and differential gene expression (DEG) as previously described (46). The UAMS Genomics Core generated demultiplexed fastq files, which were analyzed with the 10x Genomics Cell Ranger 3.1.0 count function for sequence alignment and gene counting. The reads were aligned to the University of California, Santa Cruz (UCSC) GRCm39 reference transcriptome using STAR, and transcript counts were generated (54, 55). We used the R package Seurat to further analyze the filtered, aggregated, and in-depth normalized counts generated by *cellranger count* and *cellranger aggr (*56). Cells with unique feature counts above the 75th percentile plus 1.5 times the interquartile range (IQR) or below 200 unique features; total gene counts above the 75th percentile plus 1.5 times the IQR or below 1000 counts; and/or mitochondrial feature percentage above the 75th percentile plus 1.5 times the IQR or below the 25^th^ percentile minus 1.5 times the IQR were filtered out of the data.

The 2000 highest variable features were selected and the data were scaled by linear transformation and variation associated with cell death and cell cycling, which were regressed from the scaled data using mitochondrial feature percentage and cell cycle scoring values. We normalized counts using the SCTransform, and principal component analysis (PCA) was performed on the scaled data. A Uniform Manifold Approximation and Projection (UMAP) was used to visualize the clustering results and Seurat’s *FindNeighbors* and *FindClusters* functions were optimized to label clusters based on the visual clustering in the projections. We used the Seurat *FindAllMarkers* function to identify gene markers that define cluster cell types. These gene markers were compared to known markers of expected cell types to assign appropriate cell type labels (57). Differential expression analysis was performed using the *FindMarkers* to compare similar cell types across the groups. The MAST test, a GLM framework that treats cellular detection rate as a covariate, was used to determine statistically different transcription (58). The significance threshold included differential expression at an adjusted p-value < 0.055 and a Log2FC ≥ 0.58 (fold change 1.5).). Duplicate samples for each group were processed independently, and the data were computationally combined after identifying a high level of overlap between replicates (data not shown). Original files are available at NCBI/GEO accession number GSE307613.

### 2.4 Statistics and power analyses

GraphPad PRISM (10) was used with p<0.05 as the significance threshold and tests appropriate for the design. For comparison of VCD and Control mouse sera values, we used an unpaired t test with Welch’s Correction; p<0.05 (59–62). We determined the number of replicates using *post hoc* power analyses with the help of the G* Power application (63).

## 3 Results

### 3.1 Serum levels of hormones

By 138 days after the start of VCD treatment, mass spectrometric analysis confirmed a reduction in E2, and mice were no longer cycling (44). Gonadotropin levels (LH and FSH) were 8-15X those of controls, due to reduced E2 negative feedback on hypothalamic GnRH neurons (44). Notably, E2 levels in our mouse strain matched the ranges reported in studies of cycling rats (64) and in VCD-treated, acyclic mice or rats (39, 43, 65).

We also determined biphasic changes in serum GH as the mice entered perimenopause (62 days) (39) and menopause-like states (days 118 and 138). We collected serum from tail snips on these days following the start of VCD treatment. **Figure 1A** compares serum GH levels in VCD-treated mice with those of vehicle-treated controls at each of these time points. There is a rise in GH levels on Day 62, which is considered perimenopause. Recent reports indicate that this period is characterized by relatively high estrogen levels in response to rising FSH secretion (39). This is followed by a steep drop in GH levels on Day 118, which remains low on day 138, and is observed in both the control and VCD-treated mice. This decline may be independent of VCD-treatment, and possibly associated with aging, as it occurs in both groups.

By contrast with the similar levels of serum GH in both VCD-treated and controls, serum TSH levels were significantly elevated in the 138-day VCD-treated mice relative to controls (**Figure 1B**). We further determined that serum levels of thyroxine (T4) and tri-iodothyronine (T3) were slightly elevated in the 138-day VCD-treated mice (**Figures 1C, D**), although significance was seen only at the one-tail t level. Serum levels of elevated TSH with normal or near-normal levels of thyroid hormone are also seen in menopausal human female patients with subclinical hypothyroidism (38). Average levels of serum prolactin and serum ACTH remained unchanged in the 138-day VCD-treated mice (**Figures 1E, F**) compared with controls.

### 3.2 Analysis of metabolic health

Comprehensive Lab Animal Monitoring (CLAMS) cages were used to assess the metabolic health of the mice. **Figure 2A** shows a very slight reduction in the respiratory quotient in VCD-treated females during the light phase, which is more noticeable early in the morning (**Figure 2B**). This indicates preferential use of carbohydrates as fuel. There is also a very slight reduction in energy expenditure in both the light and dark phases in VCD-treated females (**Figures 3C, D**). **Figure 2E** displays the typical nocturnal pattern of increased ambulation and grooming (X-axis activity), while **Figure 2F** shows the normal increase in jumping or rearing (Z-axis activity) at night, with no difference between groups. **Figure 2G** demonstrates the usual reduction in percent time spent sleeping during the dark phase in both groups, with no group differences in sleep patterns.

**Figure 2:**
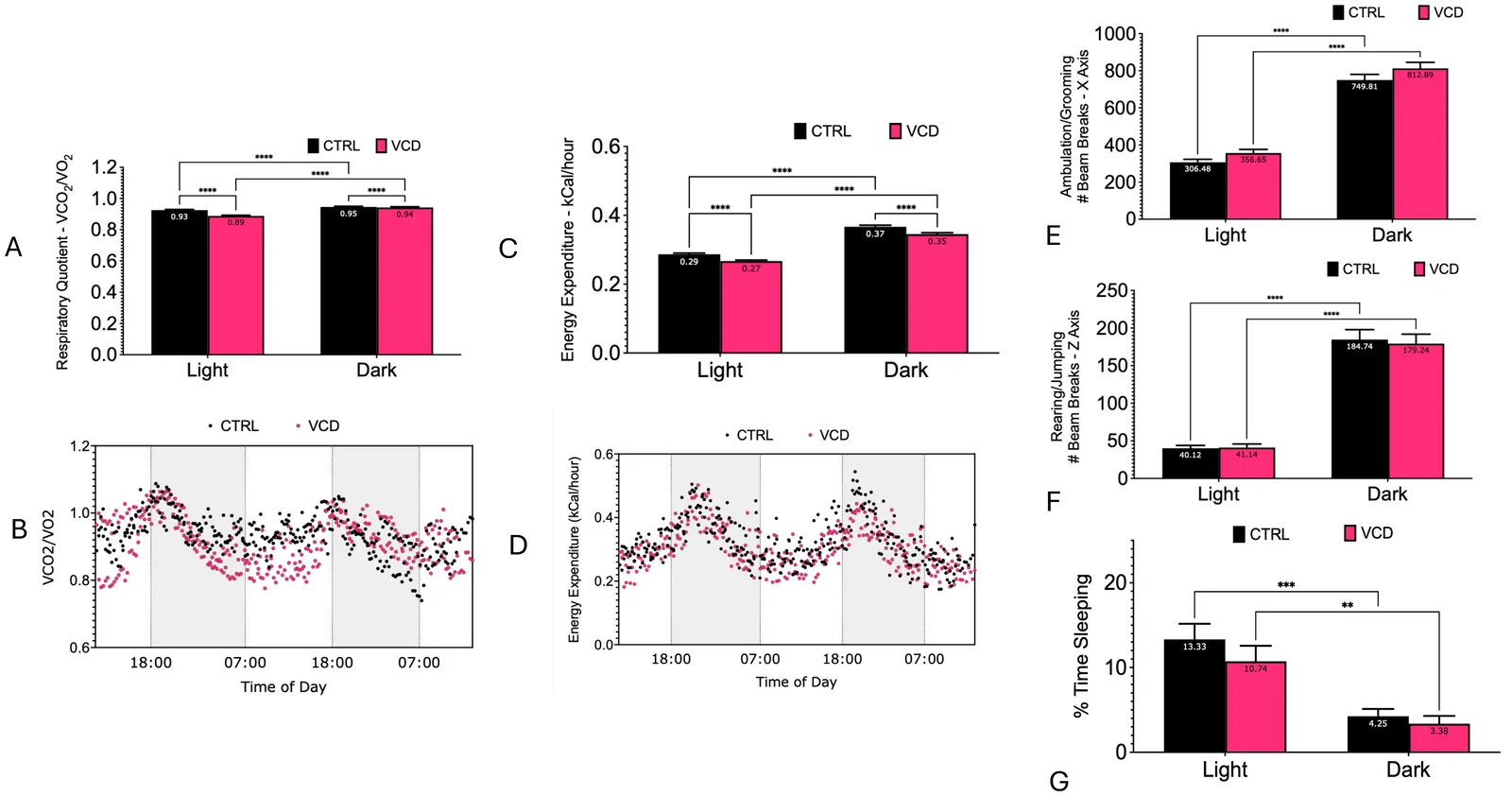
Analysis of metabolic health in mice 138 days after the start of VCD treatment. Treated females show very slight changes in respiratory quotient and energy expenditure. There were no changes in activity when the groups were compared. **(A)** Respiratory Quotient average; **(B)** Respiratory Quotient individual datapoints, **(C)** Energy expenditure averages; **(D)** Energy Expenditure datapoints; **(E)** Ambulation and grooming activity average beam breaks; **(F)** Rearing and jumping activity beam breaks; **(G)** Percent Time Sleeping (no activity). **=p<0.001, ***=P=0.001, ****=p<0.0001.

**Figure 3:**
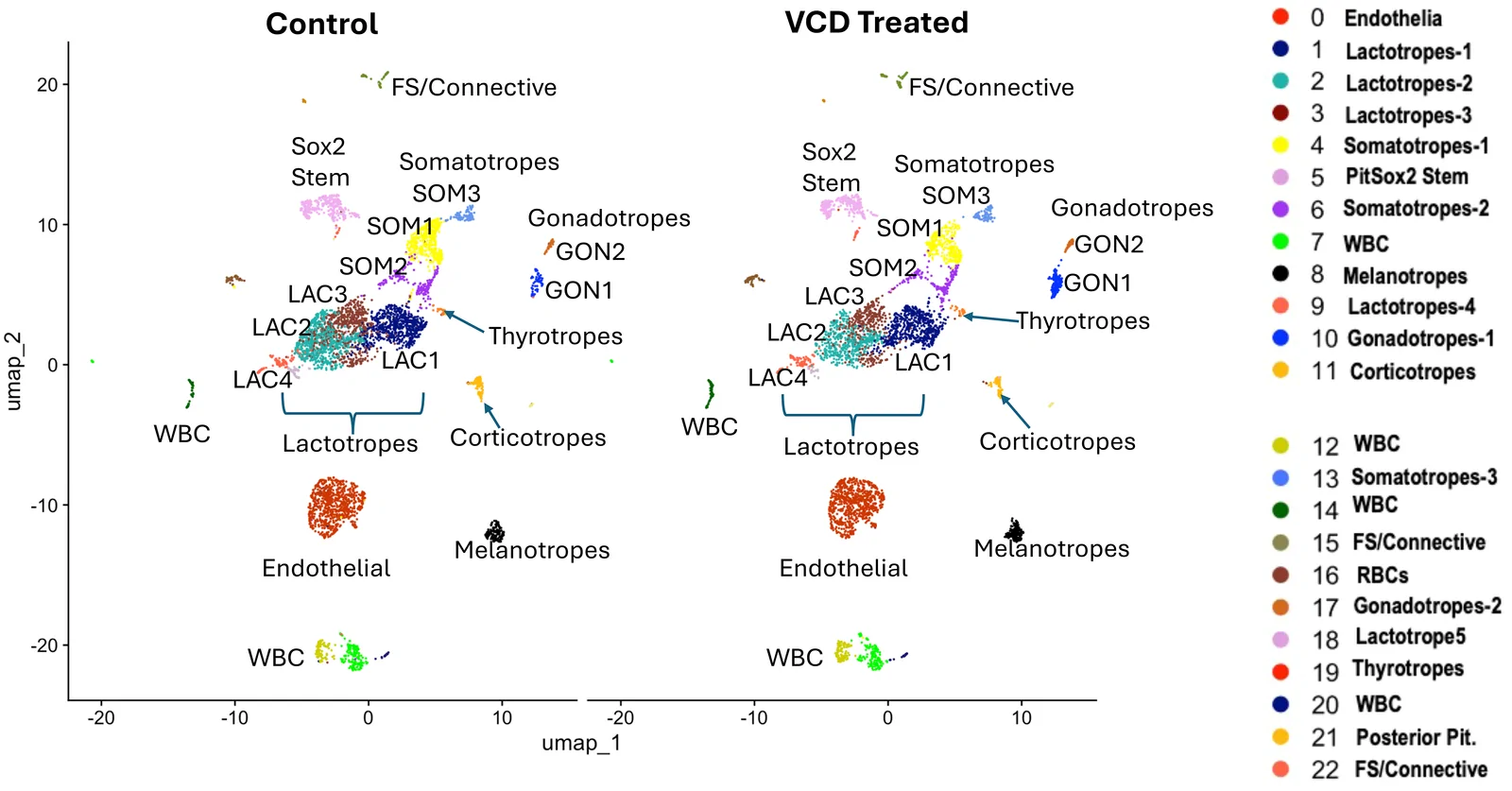
UMAP depiction of pituitary cell clusters comparing Vehicle-treated controls with VCD treated, 138 days after the start of treatment. Somatotropes and Lactotropes showed evidence of subclusters.

### 3.3 Single-cell transcriptomics responses to VCD treatment

Duplicate samples for each group were processed independently, and the data were computationally combined after identifying a high level of overlap between duplicates (data not shown). A Seurat nearest-neighbor algorithm was used to cluster cells computationally based on assigned gene signature identities and established pituitary endocrine and stem cell markers (50, 66, 67) (**Figure 3**). Cell populations with markers of somatotropes, lactotropes, thyrotropes and corticotropes were resolved into three subpopulations of somatotropes, four subpopulations of lactotropes, and one population each of thyrotropes or corticotropes (**Table 1**). Notable differentially expressed genes (DEGs) with VCD treatment are shown in **Table 2**.

**Table 1:** Expression of markers for somatotropes, corticotropes, lactotropes, and thyrotrope clusters.

Gene	Avg_Log2FC	P_val_adj	Cluster	Subcluster
Somatotropes				
*Ghrhr*	4.715589	5.44E-177	4	SOM1
*Ghrhr*	2.1806	6.67E-05	6	SOM2
*Gh*	4.03	0.05	4	SOM1
*Gh*	4.417	9.66E-109	13	SOM3
*Pou1f1*	0.52	4.79E-10	4	SOM1
*Pou1f1*	0.69	5.79E-06	6	SOM2
*Esr1*	0.57	1.18E-19	4	SOM1
*Esr1*	1.2	8.71E-33	6	SOM2
Lactotropes				
*Prl*	0.268	3.9E-69	1	LAC1
*Prl*	2.2	0.05	2	LAC2
*Prl*	1.19	1.59E-217	3	LAC3
*Prl*	2.63	6.4E-34	18	LAC4
*Pou1f1*	1.39	1.67E-48	1	LAC1
*Pou1f1*	0.695	2.82E-56	2	LAC2
*Pou1f1*	1.07	1.55E-49	3	LAC3
*Esr1*	1.677	1.19E-200	1	LAC1
*Esr1*	0.35	1.24E-41	2	LAC2
*Esr1*	0.55	1.23E-35	3	LAC3
*Angpt1*	2.37	0.05	1	LAC1
*Angpt1*	1.02	9.5E-161	2	LAC2
*Angpt1*	0.869	1.59E-88	3	LAC4
*Edil3*	2.3	4.9E-278	1	LAC1
*Edil3*	0.65	7.01E-67	2	LAC2
*Edil3*	1.02	2.76E-79	3	LAC3
*Hepacam2*	1.58	2.51E-42	1	LAC1
*Hepacam2*	1.11	6.8E-35	2	LAC2
*Hepacam2*	1.21	6.16E-29	3	LAC3
Thyrotropes				
*Trhr*	5.18	3.73	19	Thyrotropes
*Tshb*	4	7.19E-58	19	Thyrotropes
*Cga*	0.652	8.74E-27	19	Thyrotropes
Corticotropes				
*Pomc*	3.871	8.53E-137	11	Corticotrope
*Crhr1*	6.76	0	11	Corticotrope

**Table 2:** Differential expression of key hormone/receptor genes: VCD vs control.

Gene	SOM1	SOM2	SOM3	LAC1	LAC2	Thyro	Cort
*Prl*	-1.26, p=0.02*	-0.836, p=4.6E-06*	-0.62,p=0.0001*	-0.33, p=1.5E-05	-0.4, p=2.0E-45	-1.25, p=0.02*	NC
*Pomc*	NC	NC	NC	NC	NC	NC	-0.31, p=0.02
*Fshb*	-1.37, p=0.037*	NC	NC	-1.58, p=0.0019*	NC	NC	NC
*Ghrhr*	-0.67, p=0.05*	NC	NC	NC	NC	NC	NC
*Thra*	NC	-1.35, p=0.005*	NC	0.72, p=0.01*	NC	NC	NC
*Thrb*	NC	NC	NC	0.82, p=5.51E-08	0.5, p=0.0008	NC	NC
*Dio2*	NC	NC	2.8, p=0.03	NC	NC	NC	NC

*Significantly different from control.

### 3.4 Analysis of pituitary hormone mRNA levels

To further assess the scRNA-seq data we measured mRNA levels of specific targets by RT-qPCR assays as shown in **Figure 4** comparing total pituitary mRNA from VCD-treated females with control females taken 118–138 days post VCD treatment. **Table 3** shows the primer sequences (53). **Figure 2A** shows a feature plot, which illustrates the reduction in *Ghrhr* mRNA in somatotrope clusters, as shown in **Table 2**, although total pituitary *Ghrhr* mRNA levels were unchanged (**Figure 2B**). In **Figure 2C**, the lack of change in *Gh* mRNA does match the lack of change in the scRNA-seq data for somatotropes and other cell types. **Figure 2D** shows that prolactin (*Prl)* mRNA levels are unchanged in total pituitary, despite reductions in *Prl* in somatotrope, thyrotrope, and gonadotrope (44) populations. The lack of change in *Prl* in lactotropes (**Table 2**) may have compensated for the *Prl* reductions in non-lactotrope populations.

**Figure 4:**
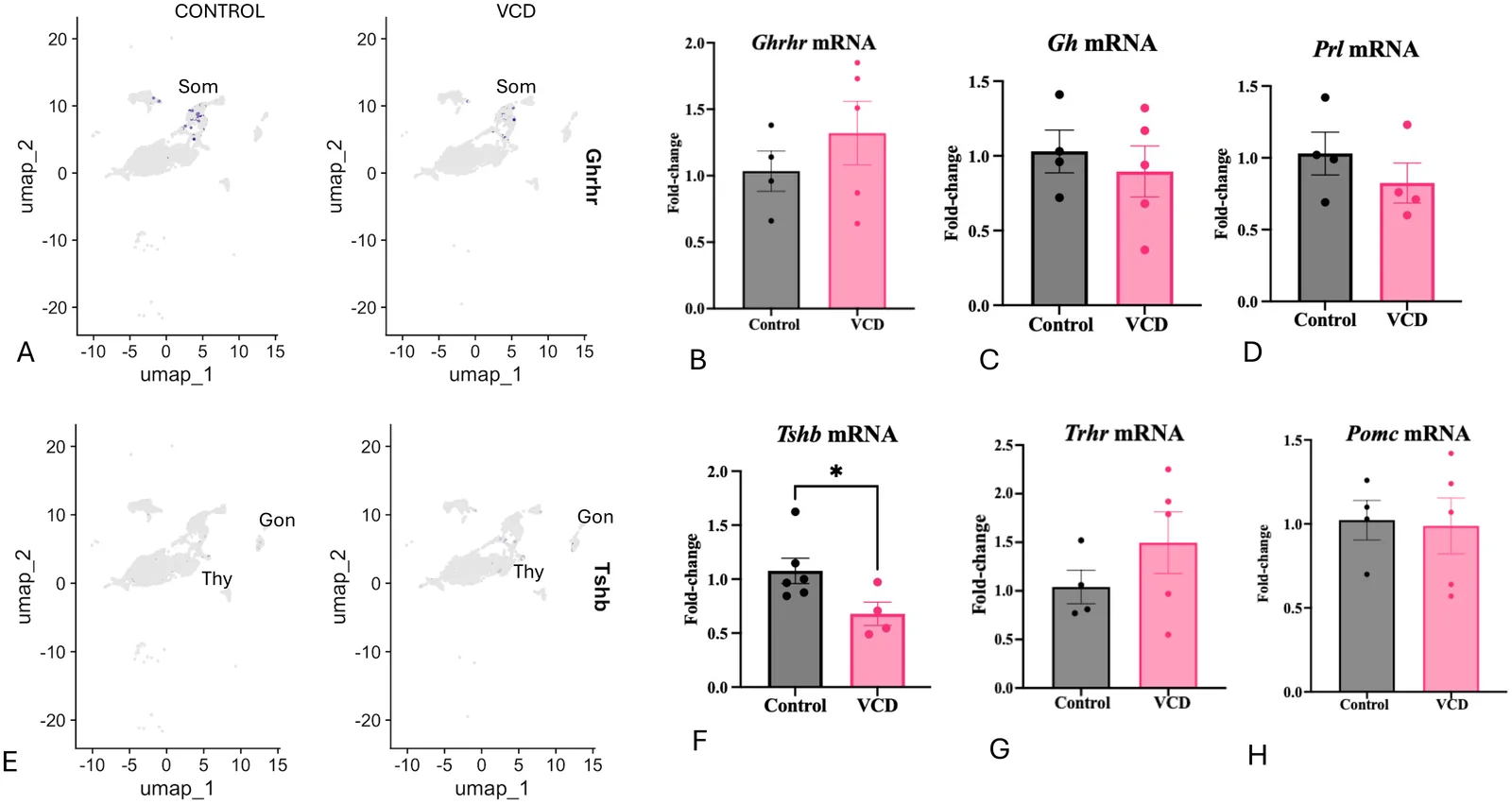
qPCR assays of *Ghrhr, Gh, Prl, Tshb, Trhr*, and *Pomc* mRNA levels in total pituitary extracts **(B–D, F–H)**. These are compared with feature plots showing reduced *Ghrh*r mRNA in the somatotrope cluster **(A)** from VCD group and reduced *Tshb* in gonadotrope cluster from VCD group **(E)**.

**Table 3:** qPCR primer sequences.

Target	Reference sequence #	IDT ID	Forward sequence	Reverse sequence
Ppia	NM_008907.2	Custom Oligo	TGGTCTTTGGGAAGGTGAAAG	TGTCCACAGTCGGAAATGGT
Gh	NM_008117.3	Custom Oligo	CCTCAGCAGGATTTTCACCA	CTTGAGGATCTGCCCAACAC
Ghrhr	NM_001003685.3	Custom Oligo	ACCCGTATCCTCTGCTTGCT	AGGTGTTGTTGGTCCCCTCT
Prl	NM_011164.2	Custom Oligo	GCCATCTTGGAGAAGTGTG	ACAGATTGGCAGAGGCTGAA
Tshb	NM_001165939.1	Custom Oligo	CTCCGTGCTTTTTGCTCTTG	TTGCCATTGATATCCCGTGT
Trhr	NM_003301.7	Custom Oligo	TGACТСААТССATCAGAACAAGA	GGCAAACAGAATTACAACCACT
Pomc	NM_008895.4	Custom Oligo	GAGGCCTTTCCCCTAGAGTT	CACCGTAACGCTTGTCCTT

**Figure 2E** shows the feature plot illustrating the distribution of *Tshb* mRNA and its reduction in gonadotropes, which correlates with the reduced *Tshb* levels detected in both gonadotrope clusters in our previous report (44). This reduction also correlates with the assays of total pituitary mRNA in **Figure 2F**. Finally, *Trhr* and *Pomc* mRNA levels were unchanged in the total pituitary extract (**Figures 2G, H**), which correlates with the scRNA-seq analyses.

### 3.5 Differential gene expression in somatotropes

Somatotropes have been shown to express genes that are markers of other pituitary cell types (4, 44–46, 58, 64, 66–68). Here, VCD treatment resulted in a significant reduction in expression of the lactotrope marker *Prl* across all somatotrope subpopulations, with no pan-somatotrope changes in expression of other pituitary cell-type markers, e.g., *Pomc*, *Tshb*, or *Lhb*. Also, in agreement with the pituitary mRNA content assays (**Figure 2**), there is no change in *Gh* mRNA in any of the somatotrope clusters. Furthermore, the reduced *Prl* in somatotropes did not appear to affect the overall concentration of anterior pituitary *Prl* mRNA as it was also unchanged (**Figure 2**).

The bioinformatic analyses distinguished 3 different clusters that had somatotrope markers (designated SOM1, SOM2, and SOM3) (**Figure 4**), with cell numbers in SOM most abundant. In the VCD-treated mice, the percentage of pituitary cells in SOM1 was reduced by 26%, while the percentage of pituitary cells in SOM2 and SOM3 increased by 9% and 25%, respectively, for an overall 10% reduction in the percentage of pituitary cells with somatotrope markers in the VCD-treated group. Analysis of DEGs showed distinctions among the three somatotrope subpopulation clusters (**Supplementary Tables S1**–**S3**), with most notably, *Ghrhr* mRNA reduced only in the SOM1 cluster.

We used Ingenuity Pathway Analysis (IPA) to predict adaptive changes to the low E2 environment in the transcriptomes of the three somatotrope subpopulations. Changes in a group of DEGs are used to predict that a signaling pathway, biological function, or upstream regulator represented by a set of DEGs may be altered. **Figure 5A** shows this comparative analysis of canonical pathways. Note that, based on the DEG analysis, IPA predicts that translational regulatory pathways are upregulated in SOM1, with mixed responses in SOM2 and SOM3. This figure also shows that, based on the upregulated DEGs representing signaling pathways critical for GH secretion in SOM1 (68), CREB, G-Protein, ERK/MAPK, and Estrogen Receptor signaling pathways are predicted to also be upregulated in SOM1. However, because DEGs representing these same pathways in SOM2 and SOM3 are downregulated, IPA predicts inhibition of these pathways.

**Figure 5:**
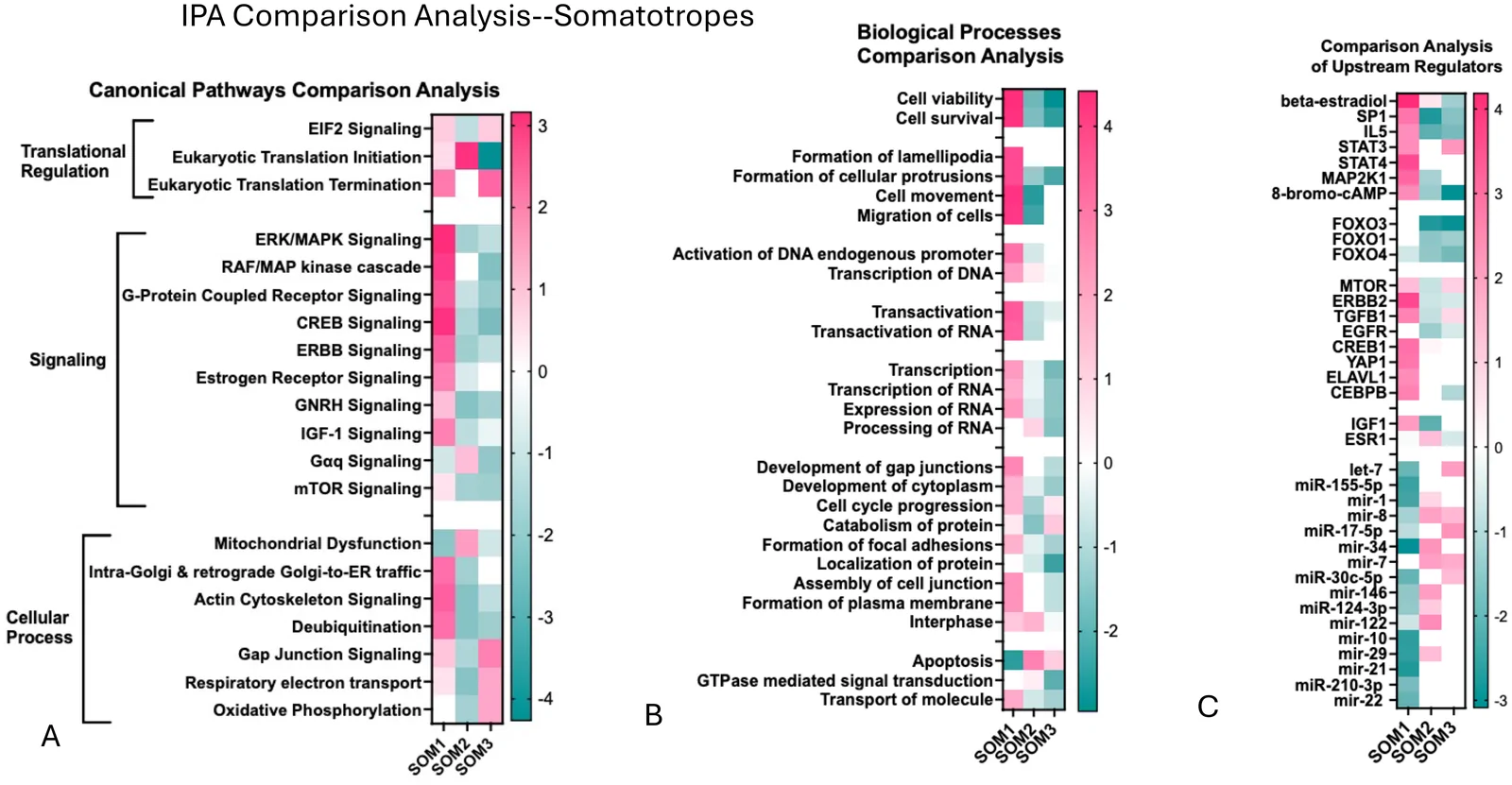
Ingenuity pathway analysis and comparison of canonical pathways, Biological Processes and upstream regulators in the three somatotrope subclusters. Changes are based on differential gene expression in the somatotrope subclusters exposed to low estrogen. The comparison analysis emphasizes the different responses of cells in these subclusters. **(A)** Comparison Analysis of Canonical Pathways; **(B)** Comparison Analysis of Biological Processes; **(C)** Comparison Analysis of Upstream Regulators.

**Figures 5A, B** are based on DEGS representing key cellular functions, e.g., protein trafficking. Because the DEGs are upregulated in the SOM1 cluster and downregulated in SOM2, IPA predicts protein trafficking to be differentially changed in SOM1 vs SOM2. Similarly, based on the direction of transcriptomic change, IPA predicts that respiratory electron transport and oxidative phosphorylation are upregulated in SOM1 and SOM3 but downregulated in SOM2. DEGs representing cell viability and survival were increased in SOM1, but not in SOM2 or SOM3, while DEGs representing apoptosis were decreased in SOM1 and increased in SOM2 and SOM3. The heatmaps in **Figures 5A, B** show similar expression directions in the pathways represented by these genes.

**Figure 5C** shows predicted upstream regulators and the direction of regulation based on the direction of the observed expression of the target DEGs. Note that Estradiol is among the top predicted regulators, and most of its targets in SOM1 are upregulated (in contrast with targets in SOM2 and SOM3). Also, most miRNAs in **Figure 5C** are down-regulated in SOM1. This would be consistent with their identified target DEGs, which are upregulated in SOM1. In contrast, the same target DEGs are downregulated in SOM2 and/or SOM3, which is associated with upregulation of miRNAs. This could hypothetically be a mechanism underlying downregulation of the target DEGs in these subclusters.

This comparative analysis allowed us to identify DEGs and their predicted signaling pathways regulated by estradiol or by GHRH (**Figures 6A–C**; **Supplementary Tables S4**, **S5**) and compare the 3 somatotrope clusters with respect to their responses associated with a low E2 environment. **Figures 6A–C** represents the IPA-generated G Protein-coupled receptor signaling pathway, which is annotated based on the data from each cluster. The three SOM clusters show differences in activation of DEGs representing the G protein-coupled receptor signaling pathway. Some genes are affected by VCD treatment across multiple SOM clusters, but no pathways are predicted to be altered in all three clusters. (e.g., signal transducer and activator of transcription, Stat3; rho-associated coiled-coil-containing protein kinase 2, Rock2; ras-related protein Rap-1b; glutamate metabotropic receptor, Grm5; free fatty acid receptor 5, Ffar5). **Figure 6A** shows the pathway represented by DEGs in the SOM1 cluster, which also shows where the SOM1 DEGs act in the pathway, and the impact of down or up regulation of a DEG on a downstream pathway. **Figure 6A** shows that one of the upregulated DEGs in the SOM1 cluster, Stat3, predicts upregulation of processes dependent on the JAK/STAT pathway.

**Figure 6:**
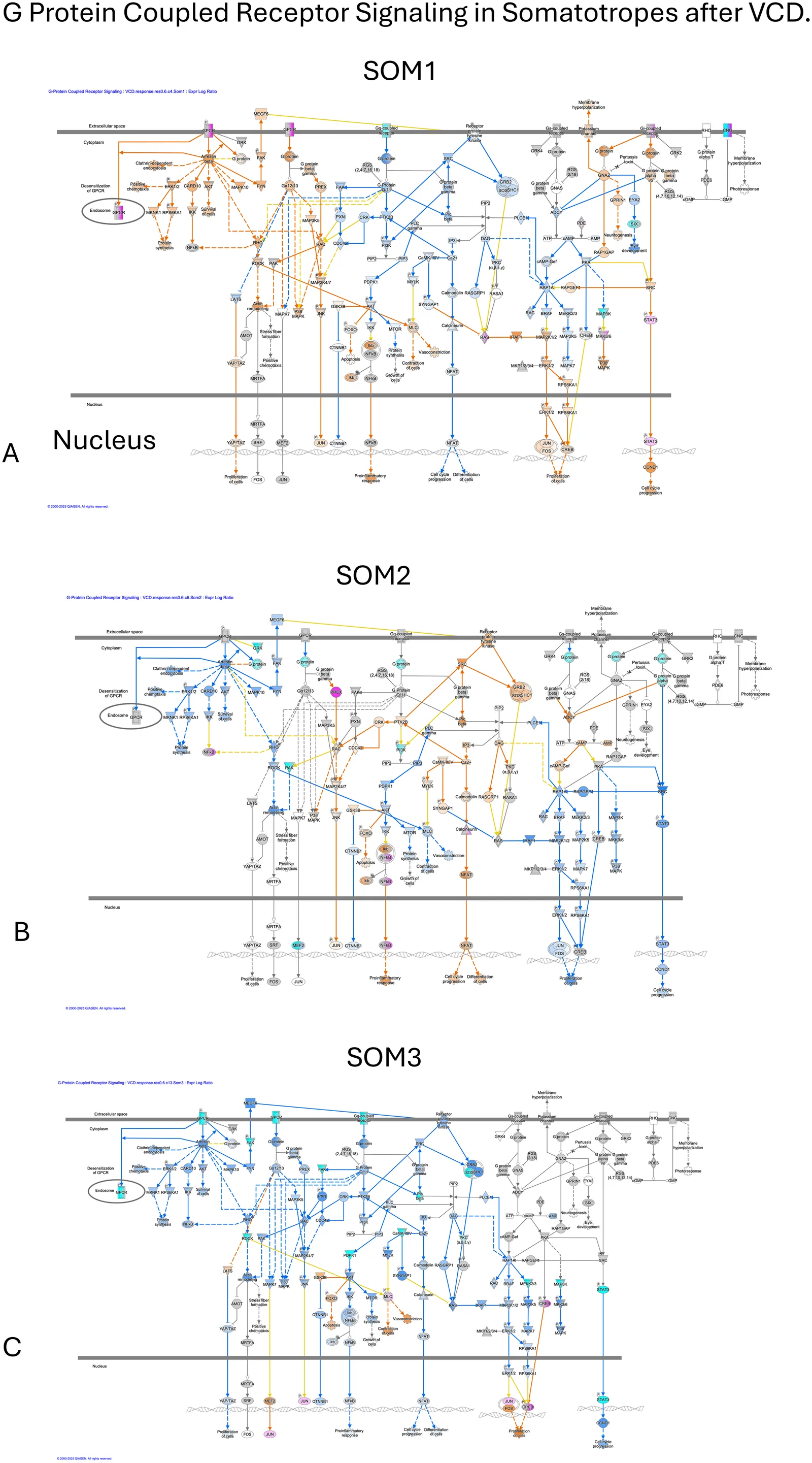
IPA analysis of DEGs in G Protein Coupled Receptor pathways based on analysis of subclusters of Somatotropes exposed to low estrogen. Key: Pink: Upregulated DEG; Turquoise: Downregulated DEG; IPA color codes predictions based on DEGs in the cluster. Orange: predicted upregulation of gene/pathway; Blue: predicted downregulation of pathway/gene. Gray, no predicted change. **(A)** SOM1=somatotrope 1 subcluster; **(B)** SOM2=somatotrope 2 subcluster; **(C)** SOM3=somatotrope 3 subcluster.

In contrast, **Figure 6B** shows that most SOM2 DEGs that are known to be part of the G protein coupled receptor pathway (also shown in **Supplementary Table S4**) are downregulated, which predicts inactivation of this and downstream pathways. Two important examples of pathways that are predicted to be downregulated are p21-activated kinase 1 (Pak), involved in actin remodeling, and phosphoinositide 3-kinase (PI3K), which is crucial in the phosphoinositide pathway). Similarly **Figure 6C** shows that SOM3 cluster DEGs in the G protein-coupled receptor pathway are predicted to be downregulated (notably G proteins, STAT3, mitogen-activated protein kinase kinase kinase (Map3K), and mitogen-activated protein kinase kinase kinase 3(Mekk2/3), thus predicting dysfunction. Only the Jun proto-oncogene (Jun) and cyclic cAMP-responsive element-binding protein 1 (Creb) transcription factors are upregulated in SOM3. Overall, based on the detected, downregulated DEGs, the G protein-coupled receptor pathway data suggest the potential for impaired GH secretion from the SOM2 and SOM3 populations in a low E2 environment.

Upstream regulators predicted to be activated or repressed based on the direction of change in their target DEGs are summarized in **Figure 5C**. Many of the upregulated DEGs in SOM1 represent known somatotrope regulators. **Supplementary Tables S5A–E** summarizes the DEG targets of E2 and the direction of their expression in each cluster. Notable upregulated DEGs in SOM1 include the nuclear receptor subfamily 4 group A member 3 (*Nr4a2*), the Activating Transcription Factor 3 (*Atf3*), which coordinates an adaptive response to cellular stress, and the Adipocyte Enhancer-Binding Protein 1 (*Aebp1*), which is important in epithelial-mesenchymal transition. Important upregulated E2 targets in the SOM2 and SOM3 populations include the gene encoding CXX finger protein 4 (*Cxxc4*), which inhibits the Wnt pathway.

Together, these findings based on differential gene expression predict that the somatotrope subpopulations show distinct responses to a sustained low-estrogen environment. The presumptive primary functional somatotrope population, SOM1, is reduced in proportion and in *Ghrhr* mRNA, while the SOM2 and SOM3 populations also indicate reduced functional responses in the absence of trophic influences from E2.

### 3.6 Differential gene expression in lactotropes

Four lactotrope subpopulations were identified bioinformatically, designated LAC1, LAC2, LAC3 and LAC4, by relative cell number. The overall proportion of pituitary cells with prolactin markers was decreased by 14% in the VCD-treated females, although the proportion of pituitary cells in LAC1 did not change. Despite the decreased proportion of prolactin cells, there were no changes in serum levels of prolactin or in the total pituitary *Prl* mRNA content (**Figure 2**), and no significant change in *Prl* in any lactotrope cluster.

IPA comparative analysis predicts that many fundamental cellular processes are upregulated in LAC1, LAC2, and LAC3, including trafficking, sorting, mitosis, and signaling, while cell death, apoptosis, and necrosis were downregulated (**Figures 7A, B**; **Supplementary Tables S6**–**S9** list the DEGs Lactotrope subclusters). IPA predicts upregulation of the G protein-coupled pathway and the Phosphatidylinositol kinase pathways, which are required for prolactin release (**Figure 8**; **Supplementary Table S10**), despite the absence of E2 trophic influence. **Figure 7C** shows upstream regulators predicted for the Lactotrope clusters, including miRNAs, which are mostly downregulated. The upregulated state of the DEG targets would predict that their miRNAs may be downregulated.

**Figure 7:**
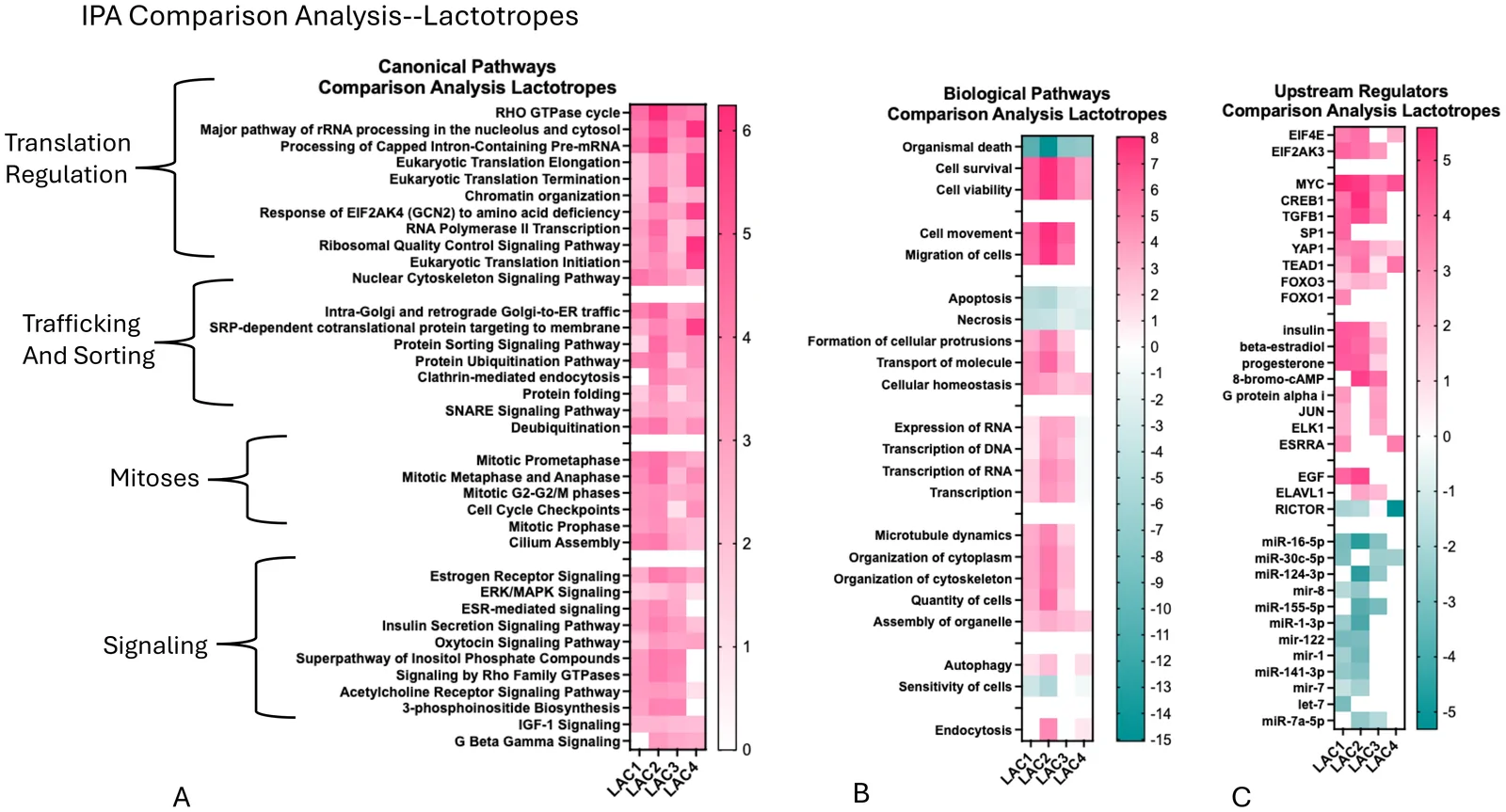
IPA comparison analysis of subclusters of lactotropes based on di:erential gene expression in mice exposed to VCD. Most processes and pathways indicate lactotropes are being activated, likely because of high TRH secretion (based on elevated TSH). **(A)** Canonical pathway comparison, **(B)** Biological pathway comparison, **(C)** Upstream regulators comparison.

**Figure 8:**
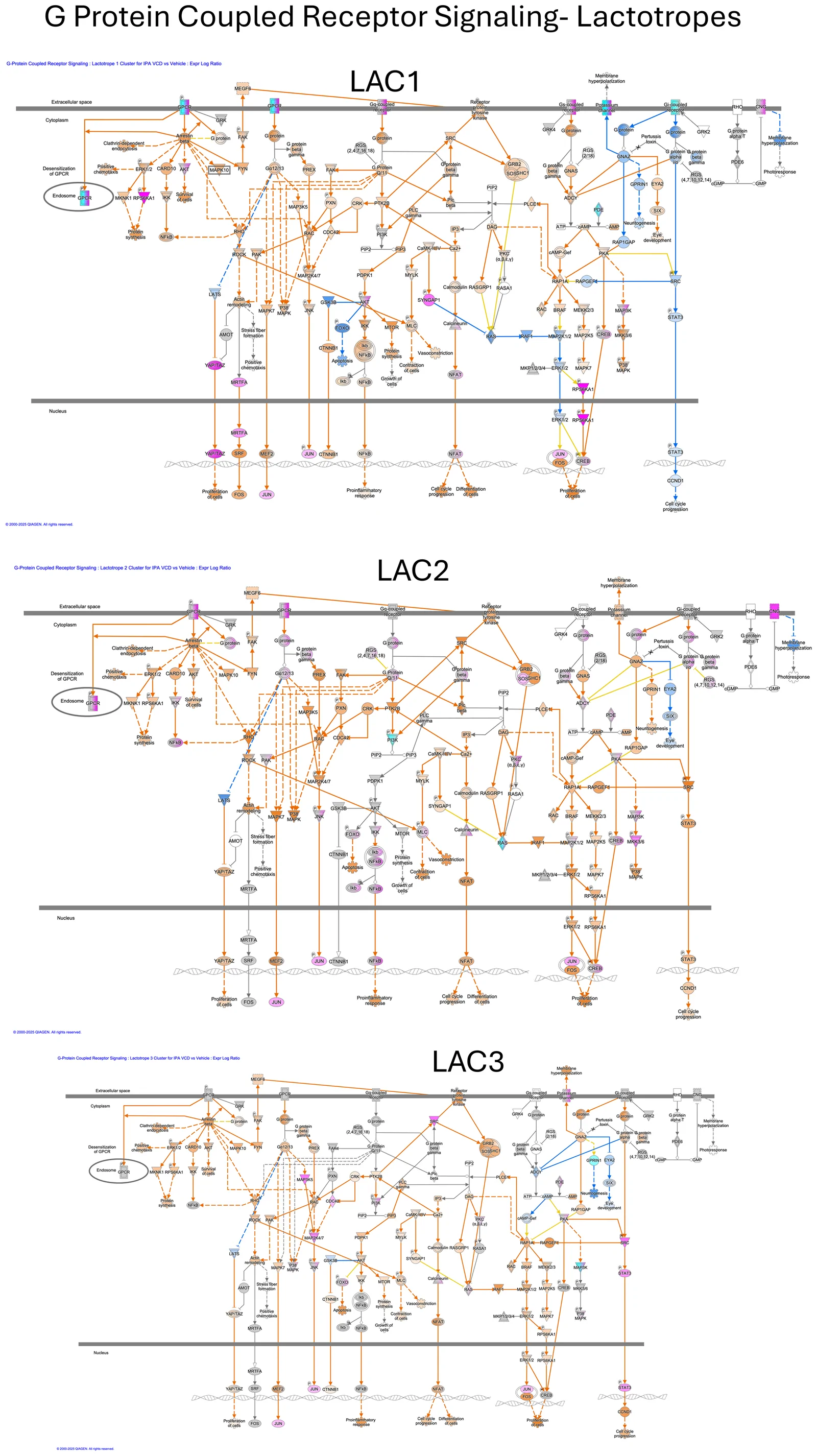
IPA analysis of DEGs in G protein coupled receptor pathways based on analysis of subclusters of lactotropes. Key: Pink: Upregulated DEG; Turquoise: Downregulated DEG; In the pathway, IPA color codes predictions based on DEGs in the cluster. Orange: predicted upregulation of gene/pathway; Blue: predicted downregulation of pathway/gene. Gray, no predicted change. LAC 1, lactotrope 1; LAC 2, lactotrope 2; LAC3, lactotrope 3; LAC4, lactotrope 4.

Our identification of the elevated serum TSH levels suggests stimulation by hypothalamic-derived thyrotropin releasing hormone (TRH), although TRH levels cannot be measured directly in peripheral blood. If elevated, TRH could also stimulate lactotropes and prolactin secretion (9). TRH activation of lactotropes involves G protein-coupled messengers, including Gs and Gq (69–72), and the Inositol phospholipase pathway. We used IPA to determine if the upregulated DEGs represent these pathways. **Figure 8** shows the G-Protein pathway annotated with changed representative DEGs from the VCD-mice LAC1 cluster. Note that several LAC1 upregulated DEGs support upregulated G proteins and pathways. Interestingly, in contrast, LAC2 in **Figure 8** indicates that, whereas it also includes upregulated G proteins, *Pi3k* is reduced, which is predicted by IPA to impair this pathway. LAC3 in **Figure 8** shows that diaerential gene expression is characterized by upregulated *Pi3k, Mapk*, and *Stat3*

Finally, in the estrogen receptor signaling pathway (**Supplementary Tables 11A, B**; **Figure 9**), target DEGs involved in signaling for prolactin secretion include calcium channel subunits, adenylate cyclase, G proteins, MAP kinases, and genes in the IP3 pathway. **Figure 9** shows the widespread distribution of predicted Estrogen receptor regulatory pathways with most pathways upregulated in the lactotropes, suggesting positive influences on downstream processes, such as protein synthesis. Thus, the evidence suggests that, despite the overall loss in proportion of lactotropes in the pituitary, the lactotropes are able to maintain secretion and synthesis of prolactin in a low E2 environment with TRH as a possible candidate upregulator.

**Figure 9:**
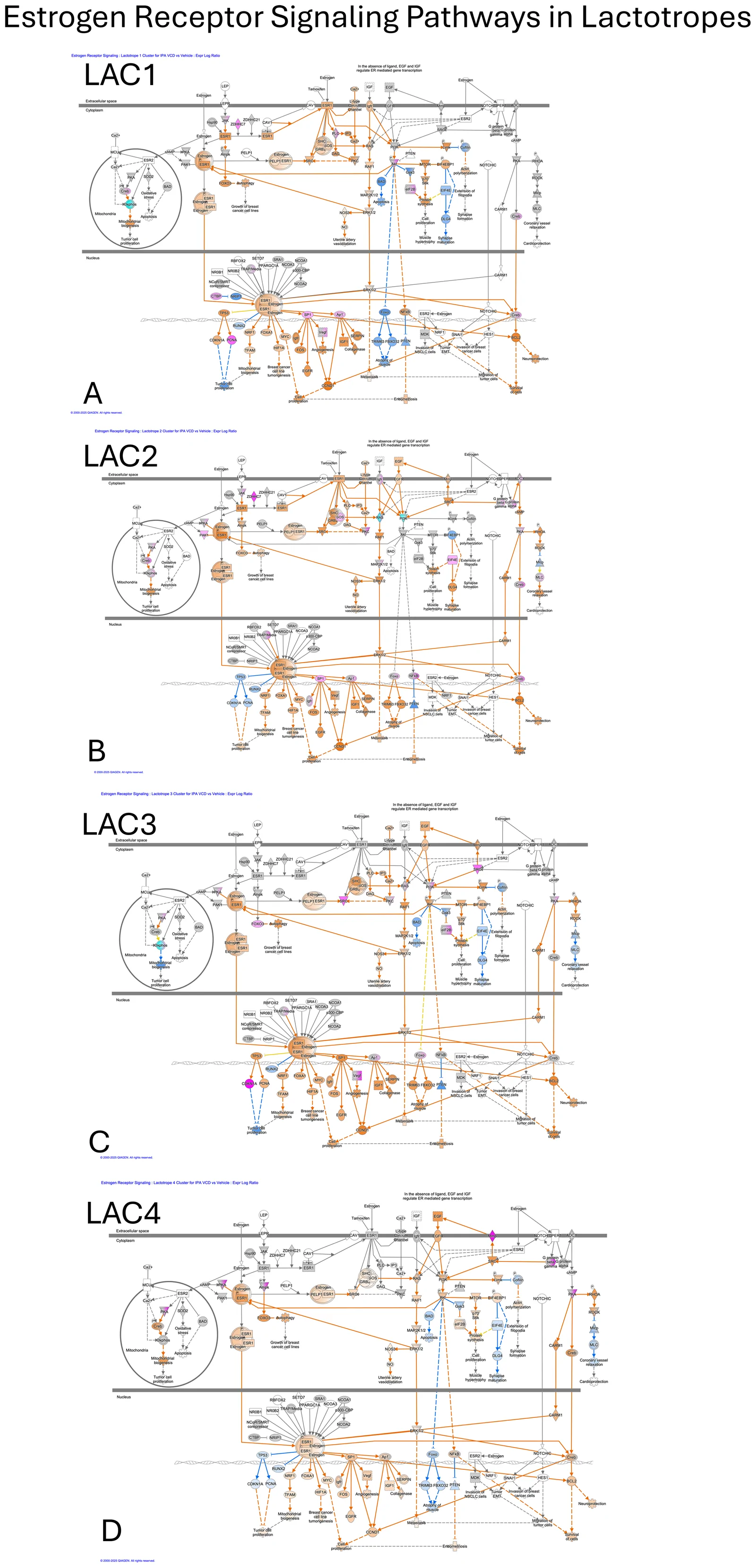
IPA analysis of DEGs in Estrogen Receptor Signaling pathways based on analysis of subclusters of Lactotropes. **(A)** LAC1 pathway; **(B)** LAC2 Pathway; **(C)** LAC3 Pathway; **(D)** LAC4 Pathway. Key: Pink: Upregulated DEG; Turquoise: Downregulated DEG; In the pathway, IPA color codes predictions based on DEGs in the cluster. Orange: predicted upregulation of gene/pathway; Blue: predicted downregulation of pathway/gene. Gray, no predicted change. LAC 1=lactotrope 1; LAC 2=lactotrope 2; LAC3=lactotrope 3; LAC4=lactotrope 4.

### 3.7 Differential gene expression in thyrotropes

As noted earlier, our VCD-treated females showed significantly elevated serum TSH over control females. The identified cluster with expression of both the thyrotrope markers *Tshb* and *Trhr* comprised a very small proportion of the total pituitary total cells in agreement with findings from other studies (50, 73). *Tshb* was also detected in the gonadotrope population (44), without *Trhr* expression. Total pituitary *Tshb* was reduced in VCD-treated females (**Figure 2**), and although the single-cell thyrotrope cluster did not show reduced *Tshb*, it was reduced significantly in the gonadotrope clusters (44). We did not detect a change in the proportion of cells in the thyrotrope cluster from VCD-treated females.

IPA analysis predicts that several canonical pathways are activated, including mitotic activity and protein translation, folding, and sorting, consistent with stimulation by TRH (**Figure 10A**; **Supplementary Tables S12A**, **S12B**). IPA also predicts cell survival and viability and downregulated apoptosis (**Figure 10A**), consistent with the increased serum TSH levels. IPA Comparison Analyses of thyrotropes and lactotropes predict the involvement of potential common canonical pathways and biological processes (**Figures 10B**, **11A, B**), including beta estradiol.

**Figure 10:**
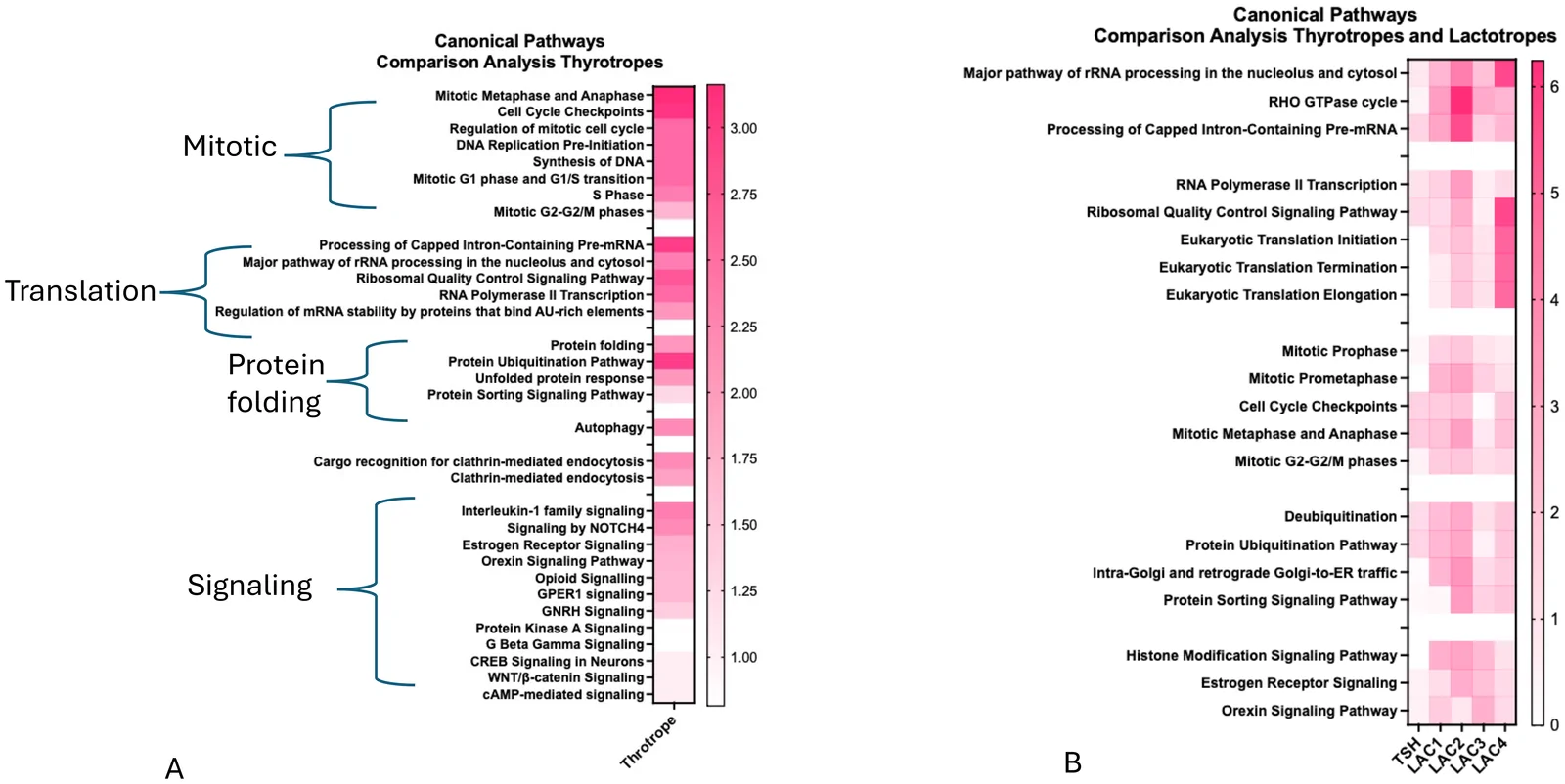
**(A)** IPA analysis of canonical pathways in thyrotropes based on DEGs in VCD-treated mice. **(B)** IPA comparison analysis of canonical pathways in thyrotropes (THY) and lactotropes, showing similar pathways, which are mostly activated, likely by TRH.

**Figure 11:**
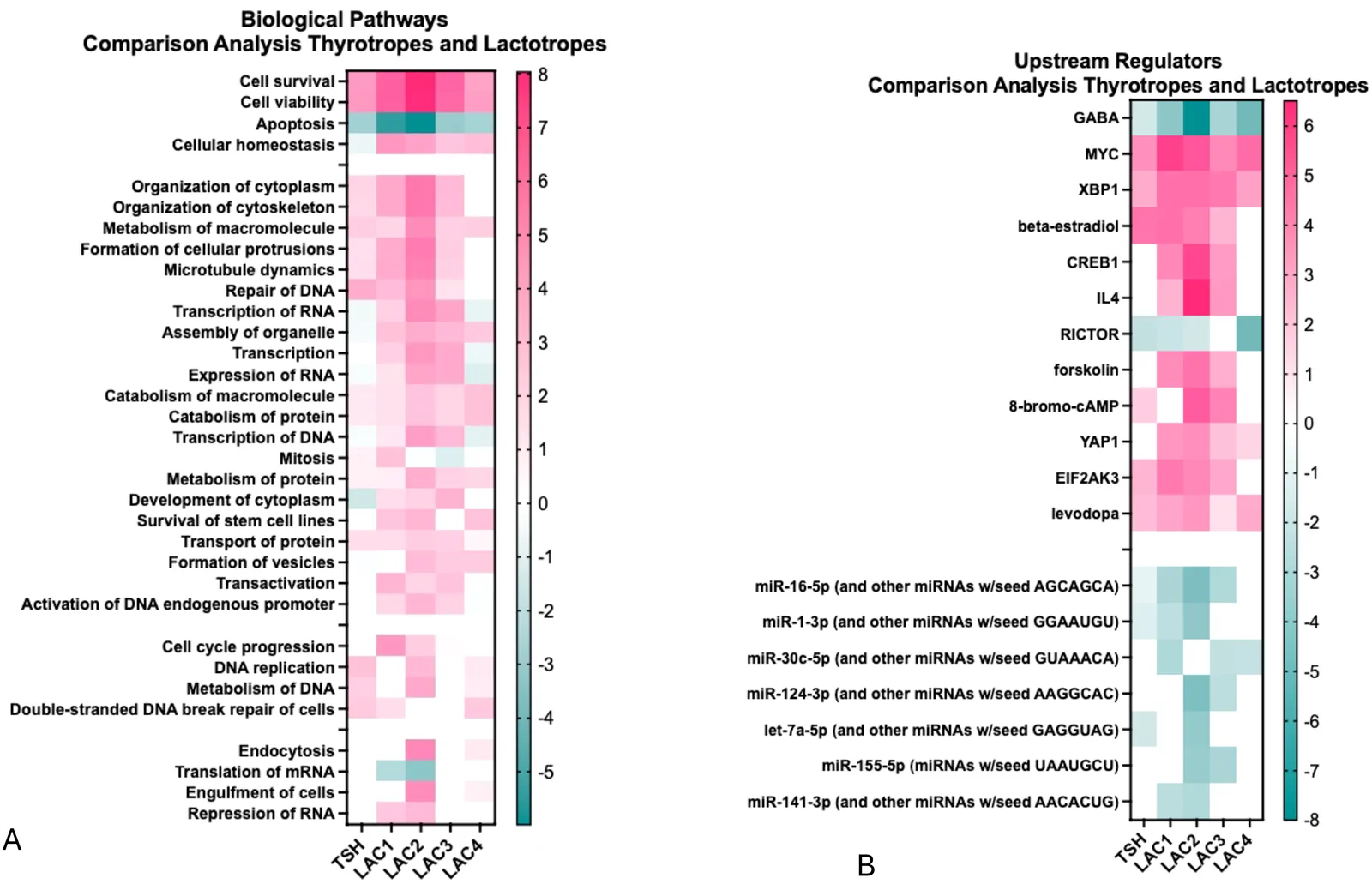
IPA comparison analysis of biological pathways and upstream regulators in Thyrotrope (THY) and lactotrope clusters, showing similar direction of responses. **(A)** Biological pathways comparison. **(B)** Upstream regulators comparison.

**Supplementary Table S13** lists the DEGs in the thyrotrope cluster associated with the Estrogen Receptor Signaling pathway, including those also differentially expressed in the LAC2 and LAC4 clusters, with the locations of these DEGs within the E2 signaling pathways shown in the IPA diagram in **Figure 12**. Notably, the decrease in Protein kinase C (*Pkc*), a PKA subunit (*Prkar1b*), and Heat shock protein 90 (*Hsp90*) in thyrotropes from VCD-treated mice (**Supplementary Table S13**), are predicted to affect several pathways. Comparing expression in thyrotrope and/or lactotrope clusters (**Supplementary Tables S14A–J**), thyroid hormone receptor alpha and beta are both predicted to be upregulated in LAC1 and LAC2 (**Supplementary Table S14I**), but not in the thyrotrope cluster. Only one gene is upregulated across all four clusters: activating transcription factor 3 (Atf3), a stress-inducible gene encoding a transcription factor important in adaptive responses to stress. Notably, this gene was also upregulated in the SOM1 and SOM2 clusters. **Table 4** summarizes known estrogen target DEGs, which are shared by thyrotropes and lactotropes, with all but two upregulated in thyrotropes and at least one lactotrope cluster. Overall, the thyrotrope population is predicted to be stimulated, which would fit with the increased serum TSH levels, although, the reduction in whole pituitary *Tshb* mRNA does not correlate with the apparent stimulated state. However, whole pituitary *Tshb* mRNA does correlate with the significantly reduced *Tshb* seen in the two gonadotrope subclusters reported in our previous paper (44).

**Figure 12:**
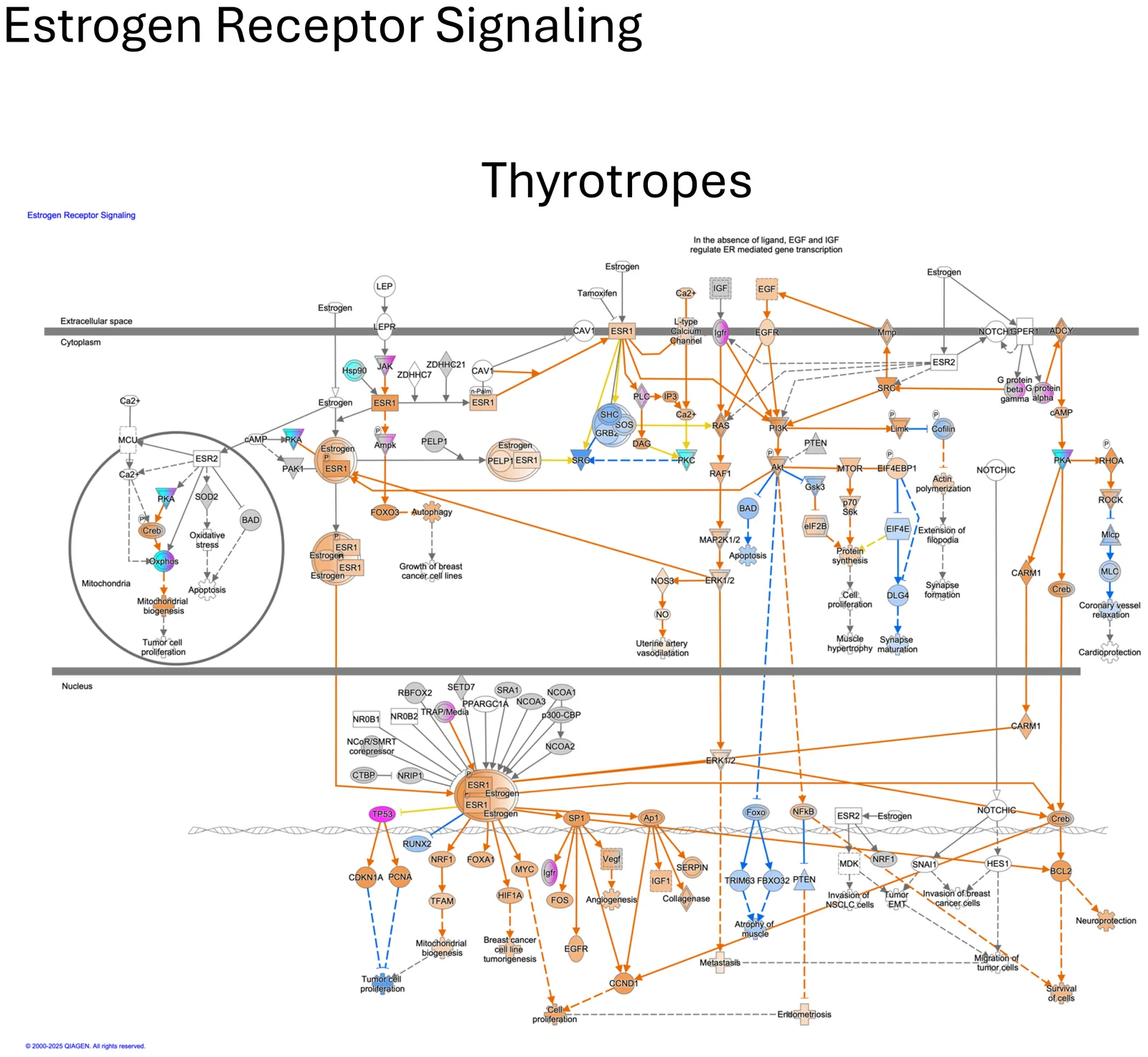
IPA generated estrogen receptor signaling pathway based on DEGs in thyrotrope cluster after exposure to VCD. Key: Pink: Upregulated DEG; Turquoise: Downregulated DEG; In the pathway, IPA color codes predictions based on DEGs in the cluster. Orange: predicted upregulation of gene/pathway; Blue: predicted downregulation of pathway/gene. Gray, no predicted change.

**Table 4:** Estrogen target DEGs shared by thyrotropes and lactotropes.

Gene	Differential expression in cluster	Function
activating transcription factor 3 (Atf3)	Thyrotropes, LAC1, LAC2, LAC3-up	Coordinates adaptive responses to stress, including ER stress
ATPase H+ transporting accessory protein 1 (Atp6ap1)	Thyrotropes, and LAC2-up	Acidification of secretory granules; Protein folding
glucose-6-phosphate dehydrogenase (G6pd)	Thyrotropes and LAC2-up	Pentose phosphate pathway--energy
G protein subunit alpha 11 (Gna11)	Thyrotropes and LAC2-up	TRH-mediated G-protein pathway which activates phospholipase C
3-hydroxy-3-methylglutaryl-CoA synthase 1 (Hmgcs1)	Thyrotropes and LAC3-up	Cholesterol biosynthesis-membrane production
insulin like growth factor 2 receptor (Igf2r)	Thyrotropes and LAC2-up	IGF signaling, proliferation; clearance of lysosomal enzymes at cell periphery
leucine rich repeat containing G protein-coupled receptor 4 (Lgr4)	Thyrotropes and LAC1-up	Marker in stem cells; may reflect the presence of adult progenitor cells
NADH:ubiquinone oxidoreductase subunit B11 (Ndufb11)	Thyrotropes and LAC1-up	Increased mitochondrial activity
protein tyrosine phosphatase non-receptor type 11 (Ptpn11)	Thyrotropes and LAC1-up	Encodes protein tyrosine phosphatase (SHP-2) to regulate RAS/MAPK signaling cascade
semaphorin 3A (Sema3a) (downregulated)	Thyrotropes and LAC3 downregulated	Elevated in hypothyroid patients. Regulates growth and migration
SMAD family member 2 (Smad2) (downregulated	Thyrotropes downregulated and LAC1 upregulated	Transcription factor reflecting differential responses to TRH.
transformer 2 beta homolog (Tra2b)	Thyrotropes and LAC2-up	Pre-mRNA splicing

### 3.8 Differential gene expression in corticotropes

Serum levels of ACTH, and total pituitary *Pomc* mRNA, are unchanged in VCD-treated mice, and similarly, *Pomc* mRNA is not significantly changed in any of the single-cell clusters, including the corticotrope cluster. We observed a 22% decline in the percentage of pituitary cells with corticotrope markers with VCD treatment, consistent with a potential role for E2 in stimulating corticotrope proliferation (14). Analysis of the downregulated DEGs by IPA predicted downregulation of Beta-catenin independent WNT signaling, protein Kinase A Signaling, and JAK/STAT signaling pathways in the corticotrope cluster (**Figures 13**, **14**; **Supplementary Tables S15A–D**, **S16**–**S19**). IPA-analyses of upregulated DEGs predicts translational pathways and G-protein and CREB signaling pathways to be upregulated, consistent with the maintenance of serum ACTH secretion despite the decreased number of corticotropes. IPA comparison of estradiol target DEGs in corticotropes and thyrotropes show two genes were upregulated in both clusters (transformer 2 beta homolog- *Tra2b* and glucose-6-phosphate dehydrogenase -*G6pdx*), with no down-regulated genes in common (**Supplementary Tables S20A–C**).

**Figure 13:**
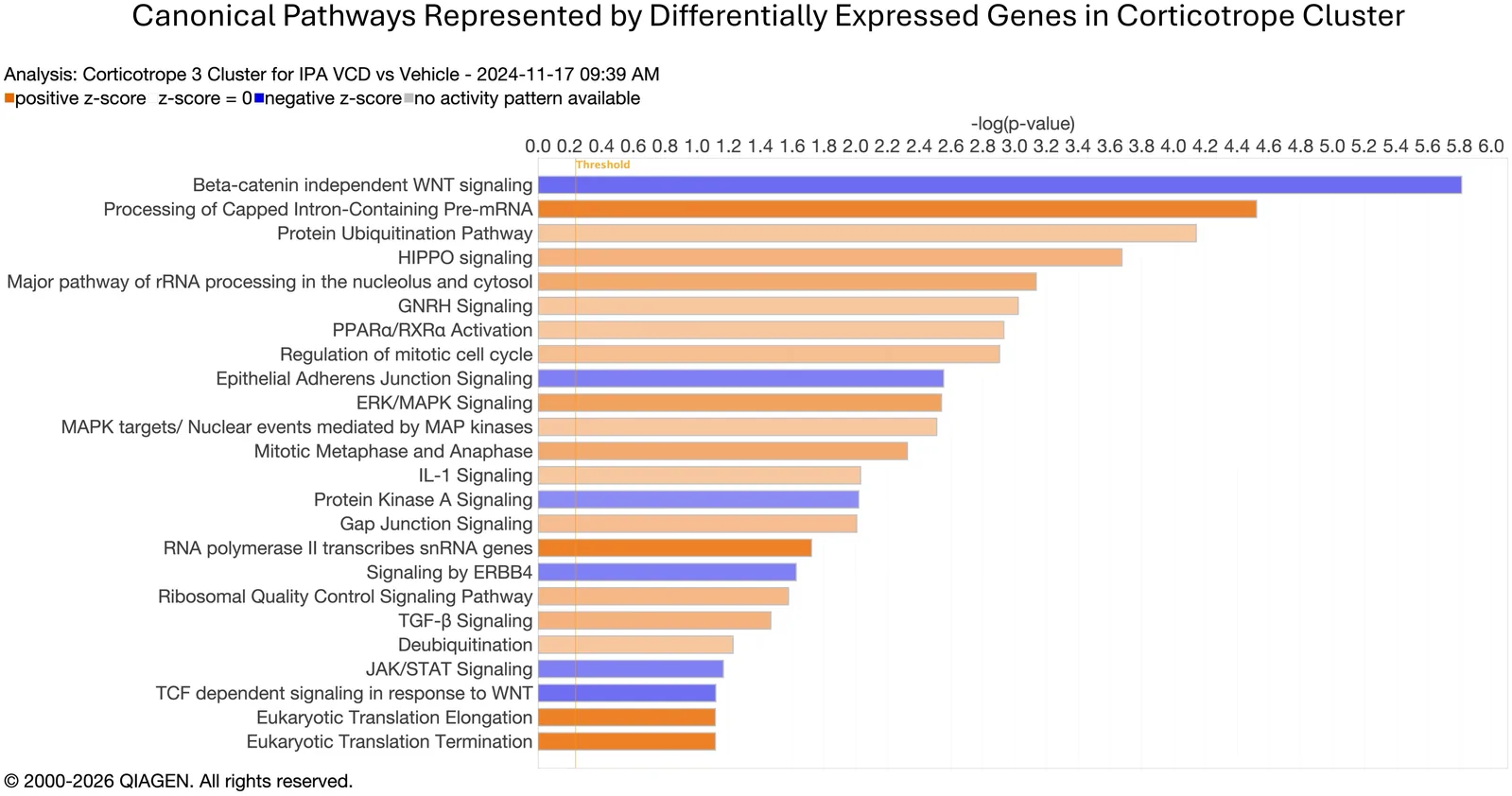
IPA pathway analysis of DEGs in corticotropes showing differential expression of specific signaling pathways based on representative DEGs after VCD exposure. Note that three pathways in corticotropes are compromised in VCD mice: WNT, Protein Kinase A and JAK/STAT signaling. However, a number of pathways have upregulated DEGs, including some related to translation.

**Figure 14:**
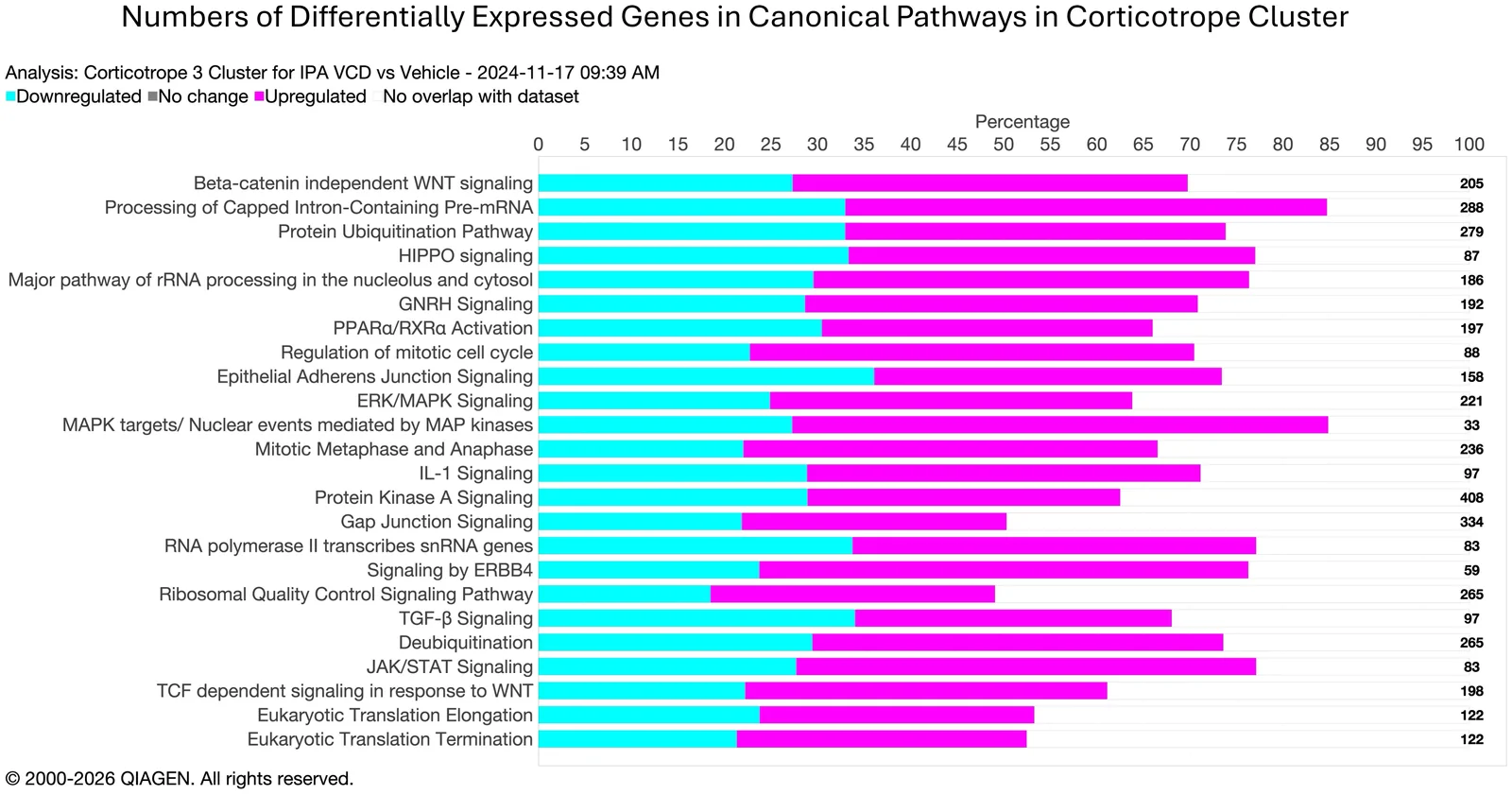
Number of differentially expressed genes in canonical pathways in corticotrope cluster.

## 4 Discussion

Here we utilized the VCD model to gradually decrease serum E2 levels in adult female mice to study pituitary responses to a menopause-like state (35, 40, 41, 44, 74). We show that the metabolic status of these mice is near-normal, suggesting that the VCD treatment had no lasting general toxic effects. In our recent report, we showed that loss of E2 negative feedback to the hypothalamic-pituitary gonadotropic axis resulted in increased serum LH and FSH levels and loss of estrous cycle progression (44). In the present study, we determined the impact of low serum E2 levels on functions in pituitary somatotropes, lactotropes, thyrotropes, and corticotropes.

Of particular interest was the significant elevation of serum TSH levels in VCD-treated females, which has been reported previously in cases of subclinical hypothyroidism in perimenopausal and menopausal human females (38). This state may occur as early as perimenopause in women and mice, which is distinguished by estrogen spikes due to the rising FSH (39). Excess estrogen can block the action of thyroid hormones (38). Therefore, serum tests in these women may show normal thyroid hormone levels, but blunted peripheral responses to thyroid hormone, thereby causing a rise in TSH due to lack of negative feedback. Our assays showed a modest elevation in serum levels of triiodothyronine (T3) and thyroxine (T4), which was significant only by one-tail t test, suggesting relatively normal thyroid function in the VCD-treated mice. Based on this evidence, we hypothesize that the mechanisms underlying the high serum TSH levels observed here may be due to pituitary and hypothalamic insensitivity to thyroid hormone negative feedback. Sun et al. suggest that this is caused by estrogen spikes during perimenopause (38, 39). Further studies are needed, however to support this hypothesis. Our studies show that expression of *Tshb*, *Thra*, *Thrb, and Dio* are unchanged in the thyrotrope population. However, activation of thyrotropes and increased production and secretion of TSH are predicted by the analysis of upregulated DEGs representing G protein signaling pathways in the VCD-treated group, and are consistent with the increased serum levels of TSH in the VCD-treated females.

In the SOM1 somatotrope cluster, upregulated DEGs predict signaling pathways utilized in normal somatotropes, including the cyclic AMP/protein kinase A/cyclic AMP responsive element-binding protein pathway, which is activated when GHRHR couples with the Gs protein (68), and the nitric oxide synthase/nitric oxide/cyclic GMP signaling pathway that is activated when GHRHR couples with the G0 protein (68). On the basis of the DEG upregulation, IPA predicts that SOM1 somatotropes are activated or potentially functional. However, the IPA analysis of downregulated DEGs in the other two somatotrope clusters (SOM2 and SOM3) predict that these pathways are downregulated. Collectively, the evidence suggests that the reduced serum GH 118–138 days after the start of treatment may reflect a reduction in functioning somatotropes compared with controls. The fact that the remaining somatotropes (in SOM1) are able to secrete levels of GH similar to those of controls, suggests resilience in the SOM1 population.

A decrease in serum E2 with aging in women is linked to a decline in serum GH (20, 31, 32), specifically due to reductions in GH pulse amplitude (33). Our study showed the expected variability in serum GH levels among control mice and VCD-treated mice 62 days after the start of VCD. We detected higher average serum GH levels in the VCD group that correlates with the known spikes in serum E2 levels reported during perimenopause in women and mice (39). The higher average levels in some VCD-treated mice in our group may reflect that the individual averages included higher-amplitude GH pulses. The subsequent reduction in serum GH levels was observed in both controls and VCD-treated groups, by days 118-138, and may have been due to aging. Hypothalamic-derived GHRH pulses are known to decline with aging in women (75), which may also have contributed to the downregulation of the GHRH receptor (*Ghrhr*) in SOM1. Analysis of GHRHR protein levels would be needed, however, to determine if translation was also reduced.

Whereas the somatotropes maintained expression of *Gh* mRNA, there was a decrease in the expression of non-somatotropic pituitary hormone genes encoding FSH (*Fshb)* and prolactin (*Prl*). Similar loss of multihormone gene expression is observed in other pituitary populations in the VCD group, e.g., gonadotropes (44), lactotropes, and thyrotropes (**Table 2**). These multihormone-expressing progenitor cells have been shown to contribute to adaptive functional plasticity in the. pituitary (76–78). Collectively, this evidence suggests that in the sustained low-estrogen state, pituitary plasticity may be compromised, which may delay or prevent the restoration and recovery of specific cell populations, in response to stress or changing organismal demands.

Expression of *Prl* in the lactotrope populations was not significantly affected by the VCD treatment, and correspondingly, serum PRL and pituitary *Prl* mRNA levels did not change. However, there was a significant reduction in *Prl* expression in the non-lactotrope populations (**Table 2** and (44)), suggesting a loss of functional plasticity and a potential contribution of PRL from these populations. Notably, all lactotrope populations showed upregulated DEGs associated with pathways supporting cell function and viability, consistent with normal serum prolactin levels. We hypothesize that lactotrope activation may be associated with stimulation, indicated by higher serum TSH levels, by TRH in the VCD-treated mice, as TRH can also stimulate lactotrope production of prolactin (9).

Corticotrope resilience in the VCD model is reflected in our finding that pituitary and corticotrope *Pomc* expression and serum ACTH levels are normal despite the reduction in proportion of corticotropes in the VCD-treated population. These cells normally possess a redundant, multi-layered regulatory network that responds to a variety of neuropeptide, metabolic, and immune regulators (79). This resilience would predictably allow them to respond appropriately to a variety of stressful stimuli. However, IPA analysis predicts that from the transcriptomic evidence, not all regulatory pathways may be available to support full corticotrope function in a low E2 environment. Further tests of corticotrope responses to stress would be needed to determine if they are compromised and thus confirm this hypothesis.

## 5 Summary

The findings in this study supported our hypothesis that a sustained low E2 environment would differentially affect transcriptomic adaptation in anterior pituitary somatotropes, lactotropes, thyrotropes, and corticotropes in a mouse model of menopause. Collectively, the findings suggest that the pituitary loses potential for cell-type plasticity that may be needed to respond to the compromised metabolic and stress responses observed in menopause. Of particular interest was the response of thyrotropes, which appeared to secrete higher levels of TSH despite near-normal levels of thyroid hormone. This state may be similar to subclinical hypothyroidism in menopausal females, believed to be caused by an E2 block in responsiveness to thyroid hormone. This study provides a single-cell description of the effects on the transcriptome of these cells and suggests a basis for future studies of pituitary regulation and signaling in the low-estrogen state. Restricting this analysis to two pools per group limits the statistical significance of the data interpretation, and although our findings suggest that a low-E2 state contributes to reduced potential pituitary function, extensive validation studies are required to support this hypothesis.

## Data Availability

The datasets presented in this study can be found in online repositories. The names of the repository/repositories and accession number(s) can be found in the article/**Supplementary Material**.
