# Supplementary material for: Integrated pituitary adaptations to the low estrogen state
Source: Front Endocrinol (Lausanne). 2026 Oct 5;17:1920998. doi: 10.3389/fendo.2026.1920998 (PMC13677488; doi:10.3389/fendo.2026.1920998)
Supplement: Supplementary file 1 [file Table1.docx]

| **Supplemental Table S1A DEGS SOM1 Cluster; VCD/Vehicle.**  Maroon=upregulated; Blue=downregulated | | | | | | | | |
| --- | --- | --- | --- | --- | --- | --- | --- | --- |
| **gene** | **avg_log2FC** | **p_val** | **gene** | **avg_log2FC** | **p_val** | **gene** | **avg_log2FC** | **p_val** |
| *Amigo3* | 4.428322376 | 3.45E-05 | *Ddhd1* | 0.606170452 | 0.052934187 | *Slc25a13* | -0.822639197 | 0.014000282 |
| *Gm32647* | 0.696101784 | 0.038923113 | *R3hcc1l* | 0.605370599 | 0.019775074 | *Vdac1* | -0.829693955 | 0.028091876 |
| *Ptprj* | 0.694340641 | 0.038431899 | *Ptpn1* | 0.60502591 | 0.024375667 | *Prl* | -0.836878729 | 4.60E-06 |
| *Pdia4* | 0.685097792 | 0.023211845 | *Arhgef12* | 0.600208894 | 0.015778654 | *Slc15a2* | -0.843700813 | 0.026259711 |
| *Pak1* | 0.685097792 | 0.026089915 | *Elf2* | 0.598247378 | 0.030730529 | *Cxxc4* | -0.846158556 | 0.010303947 |
| *Nptxr* | 0.682367999 | 0.002080449 | *Zbtb16* | 0.595582136 | 0.005067918 | *Mrps24* | -0.852963734 | 0.017666897 |
| *Grb10* | 0.680346001 | 0.036651344 | *Rnf14* | 0.592953078 | 0.054730989 | *Stk24* | -0.857079843 | 0.045177673 |
| *Bag2* | 0.679861143 | 0.014868892 | *Tspyl1* | 0.582493306 | 0.03818566 | *Pkd1* | -0.858200383 | 0.026837404 |
| *Nck1* | 0.679861143 | 0.020912868 | *Wdr26* | 0.581973323 | 0.041037493 | *Tmem134* | -0.864459373 | 0.053691918 |
| *Psmd11* | 0.678682952 | 0.014851088 | *Klf13* | 0.58143909 | 0.050103609 | *Rfx7* | -0.879106149 | 0.004083716 |
| *Ddah1* | 0.678452949 | 0.002967987 | *Stat2* | 0.580890015 | 0.042019305 | *Zfp592* | -0.881532886 | 0.025781072 |
| *Irak1* | 0.675415238 | 0.031972466 |  |  |  | *Adh5* | -0.881532886 | 0.03397085 |
| *Brms1l* | 0.675415238 | 0.048020705 | *Slc25a12* | -0.586627965 | 0.045799765 | *Smim19* | -0.885992535 | 0.013574031 |
| *Tacc2* | 0.674738711 | 0.004452457 | *Srgap2* | -0.590536651 | 0.03470715 | *Dctn2* | -0.889994465 | 0.008696365 |
| *Sec61g* | 0.67089268 | 0.004324771 | *Pcdh9* | -0.594421579 | 0.000775016 | *Unc50* | -0.889994465 | 0.016625998 |
| *Arhgap35* | 0.664688832 | 0.049676201 | *Vps35l* | -0.594761237 | 0.02586648 | *Rab22a* | -0.902594502 | 0.021701801 |
| *Rap1b* | 0.664036176 | 0.026740438 | *Mysm1* | -0.597212716 | 0.017170253 | *Eya1* | -0.907411518 | 0.013421804 |
| *Blzf1* | 0.664036176 | 0.034083871 | *Creg1* | -0.601424967 | 0.013249416 | *Cir1* | -0.909547263 | 0.03948592 |
| *Atp13a3* | 0.661609439 | 0.036515398 | *Pdgfd* | -0.610979274 | 0.000933014 | *Add3* | -0.915085446 | 0.049351216 |
| *Whrn* | 0.660495818 | 0.005244268 | *Cped1* | -0.616071743 | 0.046217676 | *Slc25a27* | -0.917452461 | 0.01354609 |
| *Tdp2* | 0.659441602 | 0.053062044 | *Atg10* | -0.622303697 | 0.025404483 | *Baiap3* | -0.926520341 | 0.054140346 |
| *Slc2a1* | 0.655732872 | 0.021279732 | *Kat6b* | -0.625343594 | 0.007309004 | *Iars* | -0.933621398 | 0.013129271 |
| *Ofd1* | 0.655732872 | 0.040329424 | *Gdpd3* | -0.628060094 | 0.008236214 | *Adam23* | -0.938459954 | 0.001325698 |
| *Mapk8ip3* | 0.654134318 | 0.013865957 | *Gpcpd1* | -0.632013287 | 0.019263166 | *Pitx2* | -0.963995047 | 0.019251483 |
| *Tent5c* | 0.653757389 | 0.009367987 | *Dleu2* | -0.638899673 | 0.021206525 | *Rnd2* | -0.963995047 | 0.025085368 |
| *Unk* | 0.653388932 | 0.042385713 | *Edf1* | -0.639268318 | 0.027183197 | *Armc1* | -0.963995047 | 0.026296594 |
| *Mctp1* | 0.65199435 | 0.022115175 | *Gm20275* | -0.646512857 | 0.030529977 | *Ttll11* | -0.963995047 | 0.02739159 |
| *Mt1* | 0.64709343 | 8.54E-06 | *Intu* | -0.647521382 | 0.038474849 | *Ttc33* | -0.963995047 | 0.033681835 |
| *Flrt1* | 0.646962663 | 0.039315325 | *Stmn3* | -0.653654926 | 0.036592551 | *Ndufb4* | -0.963995047 | 0.054260279 |
| *Phyhipl* | 0.644426427 | 0.02997807 | *Wbp2* | -0.655872751 | 0.022818286 | *Ccdc93* | -0.963995047 | 0.031271537 |
| *Ctnnd1* | 0.643335267 | 0.023907895 | *Snx24* | -0.656566521 | 0.053721801 | *Pef1* | -0.963995047 | 0.031271537 |
| *Aopep* | 0.641826059 | 7.02E-05 | *Mtch1* | -0.668539163 | 0.005844314 | *Ammecr1l* | -0.997942378 | 0.053359742 |
| *Nsd2* | 0.641726014 | 0.020086178 | *Asrgl1* | -0.671814295 | 0.055004784 | *Zfp983* | -1.000520923 | 0.015450677 |
| *Zwint* | 0.633245784 | 0.019212554 | *Cbx6* | -0.680202081 | 0.054324161 | *Pomgnt2* | -1.007063768 | 0.041741346 |
| *Snrnp70* | 0.620967454 | 0.002518997 | *Ccnd3* | -0.690234234 | 0.024905053 | *Nek6* | -1.007063768 | 0.053048537 |
| *Pak3* | 0.620967454 | 0.003929733 | *Cdr1os* | -0.700960641 | 0.01099388 | *Ift27* | -1.016462466 | 0.038550551 |
| *Tm9sf3* | 0.620967454 | 0.006098996 | *Rasgef1b* | -0.700960641 | 0.011916809 | *Gm26782* | -1.016462466 | 0.050381398 |
| *Arcn1* | 0.620967454 | 0.014595759 | *Zbtb7a* | -0.700960641 | 0.032837071 | *Dynlrb1* | -1.022888736 | 0.047857992 |
| *Scamp5* | 0.620967454 | 0.018777487 | *Ncoa6* | -0.711607885 | 0.03802099 | *Gm20631* | -1.031109242 | 0.044365674 |
| *Arid5a* | 0.620967454 | 0.020750967 | *N4bp2* | -0.71245628 | 0.038008494 | *Cavin3* | -1.031109242 | 0.044365674 |
| *Yipf5* | 0.620967454 | 0.026806194 | *Gatad1* | -0.720069464 | 0.046086991 | *Ptch1* | -1.041997559 | 0.015844146 |
| *Lgr4* | 0.620967454 | 0.029716117 | *Amz2* | -0.722986947 | 0.045423628 | *Card19* | -1.041997559 | 0.039771833 |
| *Ell* | 0.620967454 | 0.032039329 | *Asb3* | -0.724168032 | 0.011770315 | *Minpp1* | -1.041997559 | 0.039771833 |
| *4932438H23Rik* | 0.620967454 | 0.032767047 | *Blmh* | -0.7326695 | 0.045211228 | *Sfxn5* | -1.041997559 | 0.047369765 |
| *Naa35* | 0.620967454 | 0.034461594 | *Minar2* | -0.748266355 | 0.017691293 | *Uri1* | -1.04996827 | 0.00053294 |
| *Shb* | 0.620967454 | 0.036152325 | *Use1* | -0.75184181 | 0.005067972 | *Eci1* | -1.057104451 | 0.029315695 |
| *Jakmip3* | 0.620967454 | 0.044045624 | *Ncbp2as2* | -0.757544169 | 0.053277622 | *Pla2r1* | -1.057104451 | 0.050774817 |
| *Csnk2a1* | 0.620967454 | 0.045304705 | *Etfa* | -0.761502183 | 0.003241518 | *Mgat5b* | -1.057104451 | 0.053183788 |
| *Sars* | 0.620967454 | 0.046481146 | *Elof1* | -0.762361185 | 0.026470891 | *Actr8* | -1.06708854 | 0.049948493 |
| *Cdk5rap2* | 0.620967454 | 0.046864877 | *Aldh6a1* | -0.768074837 | 0.027667681 | *Nelfe* | -1.06708854 | 0.051434394 |
| *Brd7* | 0.620967454 | 0.051269851 | *Gm43376* | -0.783422801 | 0.036324444 | *Ankfn1* | -1.079472264 | 0.015605301 |
| *Ar* | 0.620967454 | 0.05215948 | *Bcl7c* | -0.794070045 | 0.043941719 | *Ap2s1* | -1.079472264 | 0.02533611 |
| *Kremen1* | 0.620967454 | 0.052541829 | *Polr1c* | -0.794070045 | 0.049655542 | *Plag1* | -1.079472264 | 0.031821992 |
| *E230016M11Rik* | 0.620967454 | 0.054930357 | *Cnot4* | -0.798257842 | 0.012229509 | *Mylip* | -1.079472264 | 0.044384491 |
| *Ttyh1* | 0.620967454 | 0.052272897 | *Mxi1* | -0.816437858 | 0.016875964 | *Ccdc34* | -1.079472264 | 0.0497371 |
| *Enox1* | 0.611865247 | 0.002790836 | *Thsd7a* | -0.818655683 | 0.000984718 | *Rfx5* | -1.079472264 | 0.0497371 |
| *Rpl37* | 0.610600739 | 2.36E-05 | *Hmg20a* | -0.821976042 | 0.008120919 | *Zfp934* | -1.079472264 | 0.052117156 |

| **Supplemental Table S1B DEGS SOM1 Cluster; VCD/Vehicle.** Blue=downregulated | | | | | | | | |
| --- | --- | --- | --- | --- | --- | --- | --- | --- |
| **gene** | **avg_log2FC** | **p_val** | **gene** | **avg_log2FC** | **p_val** | **gene** | **avg_log2FC** | **p_val** |
| *Zmym6* | -1.079472264 | 0.052721034 | *Cnih4* | -1.700960641 | 0.01680071 | *Elmo2* | -2.379032546 | 0.01262646 |
| *Bbs2* | -1.089525929 | 0.024881468 | *Leng1* | -1.700960641 | 0.026066568 | *Tbl3* | -2.379032546 | 0.01262646 |
| *Kif3a* | -1.104857582 | 0.015453383 | *Nap1l2* | -1.700960641 | 0.026066568 | *Fcrla* | -2.379032546 | 0.032234205 |
| *Idh1* | -1.11599814 | 0.039306913 | *Tctn1* | -1.700960641 | 0.036806536 | *Gm44129* | -2.379032546 | 0.032234205 |
| *Mgmt* | -1.11599814 | 0.053822363 | *Larp6* | -1.700960641 | 0.036806536 | *A230056P14Rik* | -2.379032546 | 0.032234205 |
| *Prelid1* | -1.129054293 | 0.005831387 | *Man1b1* | -1.700960641 | 0.051939529 | *Rpusd4* | -2.379032546 | 0.032234205 |
| *Zmpste24* | -1.129054293 | 0.010819089 | *Gm57084* | -1.700960641 | 0.051939529 | *Gm47888* | -2.379032546 | 0.032234205 |
| *Klhl24* | -1.140872809 | 0.002732841 | *Trim32* | -1.794070045 | 0.025957223 | *Sema5b* | -2.379032546 | 0.032234205 |
| *Sugp2* | -1.167528441 | 0.040079974 | *Pbld1* | -1.794070045 | 0.025957223 | *Gm12367* | -2.379032546 | 0.047583704 |
| *Usp54* | -1.186387468 | 0.01000218 | *Selenoo* | -1.794070045 | 0.025957223 | *Gm13648* | -2.379032546 | 0.047583704 |
| *Vmac* | -1.186387468 | 0.019962003 | *Gabra2* | -1.794070045 | 0.036672108 | *Slc29a3* | -2.379032546 | 0.047583704 |
| *Sema3a* | -1.186387468 | 0.028676058 | *4931403E22Rik* | -1.794070045 | 0.036672108 | *4933439C10Rik* | -2.379032546 | 0.047583704 |
| *Myo18a* | -1.209107544 | 0.008315345 | *Morc3* | -1.819605137 | 0.000952755 | *Snhg16* | -2.379032546 | 0.047583704 |
| *Med22* | -1.227029452 | 0.024303326 | *Prkcq* | -1.838464164 | 0.004687569 | *Kcnrg* | -2.379032546 | 0.047583704 |
| *Gm49417* | -1.237013541 | 0.009217396 | *A430073D23Rik* | -1.838464164 | 0.013077439 | *St3gal6* | -2.379032546 | 0.047583704 |
| *Klhl20* | -1.305031964 | 0.033471364 | *Cfap126* | -1.838464164 | 0.038337045 | *Rdh16* | -2.379032546 | 0.047583704 |
| *Csgalnact2* | -1.305031964 | 0.038719932 | *St6galnac4* | -1.838464164 | 0.038337045 | *Fam234b* | -2.466495387 | 0.00875143 |
| *Rfwd3* | -1.305031964 | 0.052548648 | *Zbtb14* | -1.838464164 | 0.038337045 | *Cfhr1* | -2.548957547 | 0.032232442 |
| *Six1* | -1.326565126 | 0.045753974 | *Map3k10* | -1.864459373 | 0.010379718 | *Kti12* | -2.548957547 | 0.032232442 |
| *Phldb2* | -1.330122945 | 0.003979938 | *Nphp4* | -1.881532886 | 0.012928282 | *Adam3* | -2.548957547 | 0.032232442 |
| *Zfp771* | -1.338390561 | 0.007205888 | *Dbn1* | -1.963995047 | 0.003080686 | *Zfp709* | -2.548957547 | 0.032232442 |
| *Itgav* | -1.338390561 | 0.021222097 | *Pced1a* | -1.963995047 | 0.005367739 | *Oaf* | -2.548957547 | 0.032232442 |
| *Gas7* | -1.345865682 | 0.0098525 | *Rnase1* | -1.963995047 | 0.008455113 | *Mrln* | -2.548957547 | 0.032232442 |
| *Grm5* | -1.379032546 | 0.030021052 | *Bcs1l* | -1.963995047 | 0.026480848 | *Gm47484* | -2.548957547 | 0.032232442 |
| *Mecr* | -1.379032546 | 0.031667173 | *B9d2* | -1.963995047 | 0.026480848 | *H3c15* | -2.548957547 | 0.047583704 |
| *Synpo* | -1.379032546 | 0.031667173 | *Gm9967* | -1.963995047 | 0.026480848 | *Slc43a1* | -2.700960641 | 0.015016756 |
| *Fshb* | -1.379032546 | 0.037503151 | *Nudt6* | -1.963995047 | 0.038248592 | *Gm28875* | -2.700960641 | 0.015016756 |
| *Zfp867* | -1.379032546 | 0.038454809 | *Gm4258* | -2.041997559 | 0.02566881 | *A330032B11Rik* | -2.700960641 | 0.015016756 |
| *Slc25a1* | -1.379032546 | 0.038454809 | *Gm56779* | -2.079472264 | 0.018318051 | *Bspry* | -2.700960641 | 0.02195365 |
| *Ap3m2* | -1.379032546 | 0.042524697 | *1700010I14Rik* | -2.079472264 | 0.018318051 | *Afmid* | -2.700960641 | 0.047583704 |
| *Zfp622* | -1.379032546 | 0.047215235 | *Ctdsp1* | -2.079472264 | 0.026417604 | *Gucy2f* | -2.838464164 | 0.00206899 |
| *Chdh* | -1.429658619 | 0.006954014 | *Bbs12* | -2.079472264 | 0.026417604 | *Gnpda1* | -2.838464164 | 0.002911497 |
| *Trit1* | -1.429658619 | 0.019097315 | *Gm26709* | -2.079472264 | 0.026417604 | *Rbbp9* | -2.838464164 | 0.010303865 |
| *Paip2b* | -1.429658619 | 0.025518849 | *Hapln1* | -2.079472264 | 0.026417604 | *Poll* | -2.838464164 | 0.010303865 |
| *Spns1* | -1.449421874 | 0.017252236 | *Bcat2* | -2.079472264 | 0.038167877 | *Gm2824* | -2.838464164 | 0.021954362 |
| *Tpst2* | -1.449421874 | 0.028035131 | *Pcdh17* | -2.186387468 | 0.018272618 | *Abhd17c* | -2.838464164 | 0.032233198 |
| *Wdr90* | -1.466495387 | 0.036020937 | *Gm3227* | -2.186387468 | 0.026361583 | *Ears2* | -2.963995047 | 0.007087791 |
| *Ccdc173* | -1.478568219 | 0.006857156 | *P2rx3* | -2.186387468 | 0.047585215 | *Unc13c* | -2.963995047 | 0.010301989 |
| *Fbxl4* | -1.478568219 | 0.033970186 | *Rab13* | -2.186387468 | 0.047585215 | *A730011C13Rik* | -2.963995047 | 0.015015471 |
| *Rpp25l* | -1.494509763 | 0.052329694 | *Ctsk* | -2.186387468 | 0.047585215 | *Hcn2* | -2.963995047 | 0.015015471 |
| *Rsl24d1* | -1.494509763 | 0.052329694 | *Hpcal4* | -2.186387468 | 0.047585215 | *Nop9* | -2.963995047 | 0.015015471 |
| *L3hypdh* | -1.494509763 | 0.052329694 | *Stx1a* | -2.186387468 | 0.047585215 | *Gm47167* | -2.963995047 | 0.047584459 |
| *Gm34455* | -1.494509763 | 0.052329694 | *Gm57266* | -2.186387468 | 0.047585215 | *Zfp804b* | -3.079472264 | 0.004166599 |
| *Myo5b* | -1.51653607 | 0.00144211 | *Gm54719* | -2.186387468 | 0.047585215 | *Abhd14b* | -3.079472264 | 0.010302552 |
| *Gm43696* | -1.51653607 | 0.017163577 | *Gm30524* | -2.186387468 | 0.047585215 | *Muc19* | -3.079472264 | 0.010302552 |
| *Nup107* | -1.548957547 | 0.016985052 | *Kcnh5* | -2.186387468 | 0.047585215 | *Gm15246* | -3.079472264 | 0.010302552 |
| *Ogfr* | -1.548957547 | 0.023620291 | *Gm40893* | -2.186387468 | 0.047585215 | *Snca* | -3.285923141 | 0.010303318 |
| *Mcm7* | -1.548957547 | 0.023620291 | *5330429C05Rik* | -2.186387468 | 0.047585215 | *Gm8369* | -3.379032546 | 0.00337107 |
| *6430710C18Rik* | -1.548957547 | 0.045305678 | *Top1mt* | -2.186387468 | 0.047585215 | *Tap1* | -3.548957547 | 0.002329859 |
| *Timm22* | -1.548957547 | 0.045305678 | *Gm56488* | -2.186387468 | 0.047585215 |  |  |  |
| *4732471J01Rik* | -1.580666407 | 0.023695097 | *Cep76* | -2.186387468 | 0.047585215 |  |  |  |
| *Zfp758* | -1.601424967 | 0.0369556 | *Gm45330* | -2.186387468 | 0.047585215 |  |  |  |
| *Slc20a1* | -1.601424967 | 0.05212665 | *Mettl8* | -2.253501664 | 0.00894528 |  |  |  |
| *Gm20404* | -1.601424967 | 0.05212665 | *Sgf29* | -2.285923141 | 0.018233758 |  |  |  |
| *Extl3* | -1.601424967 | 0.05212665 | *Gm6277* | -2.285923141 | 0.018233758 |  |  |  |
| *Zbtb37* | -1.626960059 | 0.032578495 | *Mak* | -2.379032546 | 0.008770097 |  |  |  |
| *Glra2* | -1.647521382 | 0.000368617 | *Gm48765* | -2.379032546 | 0.012625518 |  |  |  |

Supplemental Table 1. Differentially Expressed Genes (DEGs) in SOM1 cluster following VCD treatment. Yellow highlight indicates specific genes of interest.

| **Supplemental Table S2A DEGS SOM2 Cluster; VCD/Vehicle.** Maroon=upregulated; Blue=downregulated | | | | | | | | |
| --- | --- | --- | --- | --- | --- | --- | --- | --- |
| **gene** | **avg_log2FC** | **p_val** | **gene** | **avg_log2FC** | **p_val** | **gene** | **avg_log2FC** | **p_val** |
| *Gm12610* | 3.764215629 | 0.01175588 | *Metrnl* | 2.385704005 | 0.040322162 | *Nhsl2* | 1.764215629 | 0.039046142 |
| *Slc39a6* | 3.523207529 | 0.003523101 | *Pycrl* | 2.385704005 | 0.040322162 | *Lrrc42* | 1.764215629 | 0.039483993 |
| *Gm43813* | 3.385704005 | 0.006421807 | *Gpank1* | 2.385704005 | 0.040322162 | *Tubgcp3* | 1.741847816 | 0.008467156 |
| *Gm52988* | 3.385704005 | 0.011755989 | *Pcdhgb4* | 2.385704005 | 0.040322162 | *5430416N02Rik* | 1.741847816 | 0.031642998 |
| *Igf1* | 3.385704005 | 0.040321777 | *AI837181* | 2.385704005 | 0.040322162 | *Pnma8c* | 1.741847816 | 0.031642998 |
| *Atpaf2* | 3.233700912 | 0.011754248 | *Siah1b* | 2.385704005 | 0.040322162 | *Ift172* | 1.648738411 | 0.002357719 |
| *Gm38948* | 3.233700912 | 0.011755336 | *Xdh* | 2.385704005 | 0.044225095 | *Insig1* | 1.648738411 | 0.01279076 |
| *Sh3tc2* | 3.06377591 | 0.002585421 | *Gm50304* | 2.326810316 | 0.001654442 | *Mppe1* | 1.648738411 | 0.015934055 |
| *Cntrob* | 3.06377591 | 0.006422986 | *Mypop* | 2.286168332 | 0.015164833 | *Cux2* | 1.648738411 | 0.015963021 |
| *Mterf2* | 3.06377591 | 0.011754248 | *Tdg* | 2.233700912 | 0.013213581 | *Zyx* | 1.648738411 | 0.015997254 |
| *Arhgef33* | 3.06377591 | 0.02166037 | *Polr1a* | 2.233700912 | 0.022426248 | *Srr* | 1.648738411 | 0.035508456 |
| *Ston1* | 2.970666506 | 0.001442774 | *Shisa9* | 2.233700912 | 0.022690589 | *Tbc1d10b* | 1.648738411 | 0.046513753 |
| *Polr1c* | 2.871130832 | 0.011756424 | *Hjurp* | 2.233700912 | 0.027398615 | *Ctc1* | 1.648738411 | 0.046513753 |
| *Gm38190* | 2.871130832 | 0.021659152 | *Smg5* | 2.233700912 | 0.027398615 | *Tmem109* | 1.648738411 | 0.046513753 |
| *Tti2* | 2.871130832 | 0.021659152 | *Mfsd1* | 2.233700912 | 0.049160738 | *Tsc2* | 1.648738411 | 0.051535942 |
| *Arf6* | 2.871130832 | 0.021659152 | *Toe1* | 2.233700912 | 0.049160738 | *Slc25a14* | 1.648738411 | 0.051535942 |
| *Slc25a37* | 2.871130832 | 0.021659152 | *Rilpl1* | 2.233700912 | 0.049160738 | *Chfr* | 1.578349083 | 0.016447025 |
| *Gm44995* | 2.871130832 | 0.04032062 | *Odad1* | 2.233700912 | 0.049160738 | *Gm12940* | 1.549202738 | 0.02443746 |
| *Slc35c2* | 2.871130832 | 0.040321777 | *Fank1* | 2.233700912 | 0.049160738 | *Gpsm1* | 1.549202738 | 0.040105124 |
| *Mansc1* | 2.871130832 | 0.040321777 | *Trpc6* | 2.233700912 | 0.049160738 | *Rab5if* | 1.549202738 | 0.052228001 |
| *Endod1* | 2.871130832 | 0.040321777 | *Or2v1* | 2.233700912 | 0.049160738 | *Ccdc6* | 1.541823207 | 0.044488611 |
| *Rnf157* | 2.871130832 | 0.040321777 | *Minpp1* | 2.233700912 | 0.049160738 | *Cbl* | 1.523207529 | 0.020715011 |
| *Slc36a4* | 2.7076321 | 0.007633458 | *Acot9* | 2.233700912 | 0.049160738 | *Slc49a4* | 1.504348502 | 0.012754672 |
| *Sv2b* | 2.648738411 | 0.001567108 | *Zbtb1* | 2.179253128 | 0.008746284 | *Zxdc* | 1.504348502 | 0.013042294 |
| *Lrp1* | 2.648738411 | 0.008439112 | *Agrn* | 2.179253128 | 0.044972424 | *Top3b* | 1.504348502 | 0.02653091 |
| *Mob1b* | 2.648738411 | 0.015065136 | *Camta2* | 2.179253128 | 0.044972424 | *Grina* | 1.504348502 | 0.029157053 |
| *Gm38304* | 2.648738411 | 0.021661182 | *Cyp51* | 2.134165238 | 0.002391382 | *1700084C06Rik* | 1.47881341 | 0.024741731 |
| *Gm17231* | 2.648738411 | 0.021661182 | *Zc3h3* | 2.134165238 | 0.004010482 | *Ftsj1* | 1.47881341 | 0.028523695 |
| *Irak4* | 2.648738411 | 0.021661182 | *Cul4b* | 2.06377591 | 0.00678395 | *Pdhb* | 1.47881341 | 0.0422923 |
| *Dipk2a* | 2.648738411 | 0.026993553 | *Ccl25* | 2.06377591 | 0.013534416 | *Gm20470* | 1.47881341 | 0.05088462 |
| *Gm14004* | 2.648738411 | 0.04032062 | *Wdr5* | 2.06377591 | 0.022690665 | *Eif2ak4* | 1.442287534 | 0.010509767 |
| *Arhgef38* | 2.648738411 | 0.04032062 | *Ykt6* | 2.06377591 | 0.026404015 | *Ppp2r3c* | 1.442287534 | 0.040555604 |
| *Dnajb5* | 2.648738411 | 0.04032062 | *Rccd1* | 2.06377591 | 0.027545592 | *Trim46* | 1.442287534 | 0.052926236 |
| *Tma16* | 2.648738411 | 0.04032062 | *Daglb* | 2.06377591 | 0.045348639 | *Ptar1* | 1.442287534 | 0.052926236 |
| *Lactb* | 2.648738411 | 0.04032062 | *Csmd2* | 2.06377591 | 0.049400174 | *Cfap46* | 1.42634599 | 0.033728329 |
| *Pwp2* | 2.648738411 | 0.04032062 | *Kptn* | 2.06377591 | 0.049400174 | *Elavl1* | 1.385704005 | 0.004724276 |
| *Tubg2* | 2.648738411 | 0.04032062 | *Furin* | 2.06377591 | 0.049400174 | *Pals1* | 1.385704005 | 0.032933236 |
| *Dnah11* | 2.648738411 | 0.04032062 | *Zw10* | 2.06377591 | 0.049400174 | *Naxe* | 1.385704005 | 0.042013009 |
| *Eaf1* | 2.648738411 | 0.04032062 | *Ahr* | 2.06377591 | 0.049400174 | *Samd11* | 1.385704005 | 0.047679423 |
| *Sulf1* | 2.648738411 | 0.044597184 | *Nfkb2* | 2.06377591 | 0.049400174 | *Zbed5* | 1.385704005 | 0.047679423 |
| *Crls1* | 2.566276251 | 0.004935371 | *Rhbdd3* | 1.938245028 | 0.015776504 | *Chp1* | 1.385704005 | 0.050202046 |
| *Alas1* | 2.523207529 | 0.008456411 | *Coq5* | 1.938245028 | 0.026629282 | *Zranb2* | 1.333865074 | 0.003570697 |
| *Tmem43* | 2.47881341 | 0.00489102 | *Arl2bp* | 1.938245028 | 0.026629282 | *Rhoq* | 1.331256221 | 0.002629781 |
| *Gsk3a* | 2.47881341 | 0.00489102 | *1110017D15Rik* | 1.938245028 | 0.028102578 | *Rnf122* | 1.326810316 | 0.030393346 |
| *Prpf18* | 2.385704005 | 0.015216392 | *Pced1a* | 1.938245028 | 0.045726325 | *Clk2* | 1.286168332 | 0.01527746 |
| *Ubald1* | 2.385704005 | 0.015216392 | *Ncbp1* | 1.938245028 | 0.046105307 | *Ubp1* | 1.286168332 | 0.020857826 |
| *Fam107b* | 2.385704005 | 0.040322162 | *Rhbdd2* | 1.911772817 | 0.011555359 | *Ninl* | 1.286168332 | 0.054209009 |
| *Prex1* | 2.385704005 | 0.040322162 | *Sptbn2* | 1.911772817 | 0.050849666 | *Ski* | 1.265409772 | 0.006994308 |
| *Rasl11b* | 2.385704005 | 0.040322162 | *Cdipt* | 1.871130832 | 0.013859735 | *Slc44a1* | 1.260173123 | 0.02250547 |
| *Samd9l* | 2.385704005 | 0.040322162 | *Kndc1* | 1.871130832 | 0.023223686 | *Gng4* | 1.248200482 | 0.034619975 |
| *Zfp583* | 2.385704005 | 0.040322162 | *Vps26b* | 1.871130832 | 0.023223686 | *Macrod2* | 1.228835157 | 0.008957976 |
| *Dpf1* | 2.385704005 | 0.040322162 | *Mpp1* | 1.871130832 | 0.023223686 | *Agps* | 1.224240583 | 0.009946241 |
| *2610206C17Rik* | 2.385704005 | 0.040322162 | *Gm20208* | 1.871130832 | 0.049653272 | *Dlg5* | 1.201279434 | 0.0462587 |
| *Adam8* | 2.385704005 | 0.040322162 | *Lrrc41* | 1.841383489 | 0.004634976 | *Nol4* | 1.184070144 | 0.042215136 |
| *Gm20406* | 2.385704005 | 0.040322162 | *Fam187b* | 1.800741505 | 0.046105307 | *Prkab2* | 1.179253128 | 0.031575964 |
| *Chek1* | 2.385704005 | 0.040322162 | *Tbc1d31* | 1.800741505 | 0.046105307 | *Shfl* | 1.179253128 | 0.054931009 |
| *Mettl2* | 2.385704005 | 0.040322162 | *Armcx6* | 1.764215629 | 0.0234929 | *Eif2ak3* | 1.163311584 | 0.047370022 |

| **Supplemental Table S2B DEGS SOM2 Cluster; VCD/Vehicle.** Maroon=upregulated; Blue=downregulated | | | | | | | | |
| --- | --- | --- | --- | --- | --- | --- | --- | --- |
| **gene** | **avg_log2FC** | **p_val** | **gene** | **avg_log2FC** | **p_val** | **gene** | **avg_log2FC** | **p_val** |
| *Sgms2* | 1.154923798 | 0.002673334 | *Macrod1* | 0.701205831 | 0.052950189 | *Depdc5* | -0.79183418 | 0.026471381 |
| *Cdc14b* | 1.141778422 | 0.01285093 | *Pcp4l1* | 0.694542101 | 0.053565016 | *Tbc1d19* | -0.794205085 | 0.03417658 |
| *Ppp2ca* | 1.141778422 | 0.0300082 | *Cyp7b1* | 0.688266775 | 0.042861115 | *Hnrnpk* | -0.798720566 | 0.016243889 |
| *Gcn1* | 1.134165238 | 0.041388392 | *9330159F19Rik* | 0.682685743 | 0.052131825 | *Pak1ip1* | -0.798720566 | 0.048759548 |
| *Ppp2r2d* | 1.122669599 | 0.036437145 | *Greb1l* | 0.671458488 | 0.024612918 | *Ogt* | -0.801294509 | 0.022442869 |
| *Mindy3* | 1.118223694 | 0.014602843 | *Son* | 0.648738411 | 0.01806877 | *B4galt6* | -0.810693207 | 0.04471439 |
| *Epha6* | 1.118223694 | 0.02213157 | *Plxna4* | 0.615571547 | 0.047127698 | *Hycc2* | -0.820746872 | 0.016059249 |
| *Gdap1l1* | 1.118223694 | 0.046426312 | *Birc6* | 0.612861375 | 0.018968067 | *Papss1* | -0.82374936 | 0.019586771 |
| *Cdk17* | 1.105596086 | 0.035754246 | *Sbf2* | 0.608772004 | 0.041920345 | *Hspd1* | -0.82374936 | 0.052934176 |
| *Got1* | 1.101250616 | 0.021778053 | *Zfand3* | 0.601871806 | 0.032299269 | *Elk3* | -0.829308886 | 0.03522704 |
| *Atp6v0a1* | 1.090248122 | 0.037815854 |  |  |  | *Tmem59l* | -0.829308886 | 0.045666358 |
| *Zfp934* | 1.06377591 | 0.017825341 | *Gabarapl2* | -0.588300786 | 0.037446955 | *Bicd1* | -0.83125453 | 0.005266015 |
| *Gdap1* | 1.06377591 | 0.019627547 | *Tmed2* | -0.592269688 | 0.045121471 | *Epb41l5* | -0.836688416 | 0.045839506 |
| *Trim39* | 1.06377591 | 0.022179845 | *Adamts6* | -0.595187172 | 0.021307896 | *Prkacb* | -0.838926888 | 0.041999944 |
| *Tbc1d8b* | 1.06377591 | 0.023151481 | *Npm1* | -0.639412672 | 0.002549458 | *Arf5* | -0.840008774 | 0.002300164 |
| *Mboat2* | 1.06377591 | 0.053409531 | *Ttc28* | -0.640768206 | 0.045201007 | *Cpne4* | -0.840008774 | 0.036314617 |
| *Tyw1* | 1.06377591 | 0.054183037 | *Cd63* | -0.641480824 | 0.045720317 | *Bex3* | -0.843114685 | 0.014516862 |
| *Sorbs1* | 1.028442837 | 0.000988701 | *Mapre1* | -0.646717472 | 0.019131586 | *Akap7* | -0.843114685 | 0.016623598 |
| *Slc6a17* | 1.007192382 | 0.037923517 | *Rab14* | -0.646717472 | 0.023546298 | *Kdm5b* | -0.850494216 | 0.02038342 |
| *Fermt2* | 1.002375366 | 0.006211178 | *Aig1* | -0.646717472 | 0.034521167 | *Ccdc47* | -0.853761929 | 0.046266207 |
| *Aebp1* | 0.962847002 | 0.008318728 | *Pfdn5* | -0.64759797 | 0.004783766 | *Foxk2* | -0.862223508 | 0.02761539 |
| *Snx14* | 0.956860707 | 0.016017837 | *Hdac8* | -0.659706454 | 0.007075585 | *Trpm7* | -0.864670829 | 0.046586471 |
| *Dicer1* | 0.948298693 | 0.021610398 | *Atp5j* | -0.659706454 | 0.051867039 | *Shroom3* | -0.869109894 | 0.036361223 |
| *Stx16* | 0.944194295 | 0.002042332 | *Srebf2* | -0.660589647 | 0.05054207 | *S100a10* | -0.871129061 | 0.006849046 |
| *Lims1* | 0.93371537 | 0.010716844 | *Slc25a12* | -0.662049126 | 0.042550722 | *Bod1l* | -0.871129061 | 0.037911821 |
| *Spock2* | 0.92884633 | 0.006546253 | *Stub1* | -0.662049126 | 0.046061046 | *Atp5b* | -0.879640561 | 0.038475708 |
| *Zbtb16* | 0.918345471 | 0.009082736 | *Angpt1* | -0.668290884 | 0.012226902 | *Ndufa12* | -0.88375667 | 0.044518116 |
| *Rbbp6* | 0.911772817 | 0.027428612 | *Capns1* | -0.673189684 | 0.037276166 | *Lonrf2* | -0.88375667 | 0.0549599 |
| *Ppp2r2c* | 0.900277178 | 0.031109323 | *Rrp1* | -0.673189684 | 0.037783011 | *Rps18-ps6* | -0.893155368 | 0.021962259 |
| *Ssr2* | 0.893850909 | 0.044767495 | *Spidr* | -0.673189684 | 0.047588861 | *Crhbp* | -0.896695725 | 0.02201664 |
| *Azin1* | 0.889746511 | 0.026374876 | *Zmynd11* | -0.679884336 | 0.021638591 | *Immt* | -0.899698214 | 0.008152142 |
| *Gabpb1* | 0.886898148 | 0.052502283 | *Lrp6* | -0.680967034 | 0.022497908 | *Stmn1* | -0.911976543 | 0.00615615 |
| *Tpd52* | 0.874114691 | 0.00415603 | *Mtmr3* | -0.683458019 | 0.025460898 | *Ccpg1* | -0.912765117 | 0.014757505 |
| *Pias1* | 0.871130832 | 0.00853803 | *Tmbim4* | -0.686245837 | 0.034724204 | *G3bp2* | -0.93622409 | 0.011015502 |
| *mt-Nd4l* | 0.871130832 | 0.011350018 | *Pkm* | -0.691111592 | 0.023926893 | *Tecr* | -0.93622409 | 0.013891156 |
| *Srrm1* | 0.871130832 | 0.013950087 | *Cdc42bpa* | -0.701758836 | 0.005179668 | *Nudc* | -0.93622409 | 0.027119986 |
| *Atf3* | 0.856679676 | 0.013649167 | *Gfpt1* | -0.701758836 | 0.035539573 | *Bola3* | -0.93622409 | 0.027125587 |
| *Mef2d* | 0.846184475 | 0.029834684 | *Fggy* | -0.703563333 | 0.012908594 | *Ahsa1* | -0.93622409 | 0.027797999 |
| *Epb41* | 0.830798738 | 0.04257736 | *Cdkn1b* | -0.713831668 | 0.031431955 | *Nrsn1* | -0.93622409 | 0.033192024 |
| *Med27* | 0.825616173 | 0.032569975 | *Ttc8* | -0.713831668 | 0.042206795 | *Ppp1r11* | -0.93622409 | 0.036475912 |
| *Bmper* | 0.805463915 | 0.030182908 | *Chchd7* | -0.713831668 | 0.052475401 | *Pmpcb* | -0.93622409 | 0.038640988 |
| *Rbfox1* | 0.802089188 | 0.009207995 | *Sox5* | -0.718223437 | 0.001745845 | *Taf1b* | -0.93622409 | 0.038640988 |
| *Slc30a9* | 0.800741505 | 0.034358706 | *Mrfap1* | -0.7194127 | 0.013347325 | *Cep128* | -0.93622409 | 0.040467281 |
| *Sipa1l1* | 0.788141468 | 0.009280998 | *Cox7a2l* | -0.7194127 | 0.041178181 | *A430005L14Rik* | -0.93622409 | 0.041915873 |
| *Cert1* | 0.783667991 | 0.050758474 | *Gm14618* | -0.723494766 | 0.00180863 | *Rtl8b* | -0.93622409 | 0.054462721 |
| *Tmsb4x* | 0.78137618 | 0.036458496 | *Micu2* | -0.729773212 | 0.008100999 | *Ddx60* | -0.93622409 | 0.054599344 |
| *Pknox2* | 0.779982944 | 0.028354214 | *Nap1l1* | -0.734590228 | 0.052476741 | *Mmp14* | -0.93622409 | 0.039042681 |
| *Atxn2l* | 0.764215629 | 0.017098116 | *Prl* | -0.735402177 | 0.007576375 | *Rnf25* | -0.93622409 | 0.053598935 |
| *Abcb7* | 0.757672783 | 0.018899181 | *Syne1* | -0.738284712 | 0.025503238 | *Tle5* | -0.963704826 | 0.02726665 |
| *Zfp652* | 0.748274085 | 0.04563462 | *Tsn* | -0.743579012 | 0.037432641 | *Ptbp3* | -0.967250985 | 0.006699704 |
| *Trp53bp1* | 0.741847816 | 0.0421308 | *Rap1b* | -0.749138537 | 0.048181386 | *Dner* | -0.973698795 | 0.003944713 |
| *Spag9* | 0.739503875 | 0.00135851 | *Nptxr* | -0.753360032 | 0.04933677 | *Stau2* | -0.973698795 | 0.035927451 |
| *Pds5b* | 0.737547678 | 0.041801532 | *Desi2* | -0.763387493 | 0.046733052 | *Kpna1* | -0.978044265 | 0.024936602 |
| *Tcf20* | 0.736201252 | 0.016455976 | *U2af1* | -0.770214138 | 0.051514769 | *Mff* | -0.983529804 | 0.034176145 |
| *Nop58* | 0.725359693 | 0.016721725 | *Fchsd2* | -0.771164843 | 0.045829523 | *Ndufb10* | -0.986850163 | 0.01295023 |
| *Plcl2* | 0.706841366 | 0.009664671 | *Klhdc10* | -0.771593388 | 0.024841511 | *Cep350* | -0.992807618 | 0.042516027 |
| *Slc2a13* | 0.701888118 | 0.003332729 | *Tbcb* | -0.784220996 | 0.033002386 | *Diaph3* | -1.000354427 | 0.048530345 |

| **Supplemental Table S2C DEGS SOM2 Cluster; VCD/Vehicle.** Blue=downregulated | | | | | | | | |
| --- | --- | --- | --- | --- | --- | --- | --- | --- |
| **gene** | **avg_log2FC** | **p_val** | **gene** | **avg_log2FC** | **p_val** | **gene** | **avg_log2FC** | **p_val** |
| *Top2b* | -1.006613417 | 0.028756257 | *Ubxn6* | -1.258152184 | 0.00358947 | *Nr2c1* | -1.466738806 | 0.015518823 |
| *Ctsb* | -1.014226602 | 0.008711011 | *Neurl1a* | -1.258152184 | 0.00927151 | *Ppp4r1l-ps* | -1.466738806 | 0.036112439 |
| *Dnttip2* | -1.014226602 | 0.024945042 | *D16Ertd472e* | -1.258152184 | 0.033485624 | *Foxk1* | -1.52118659 | 0.013144217 |
| *Mlh3* | -1.014226602 | 0.037380718 | *Cry1* | -1.258152184 | 0.036414706 | *Ncaph2* | -1.52118659 | 0.014709979 |
| *Mgat4c* | -1.015658557 | 0.00125983 | *Mtrex* | -1.258152184 | 0.036513842 | *Tmcc2* | -1.52118659 | 0.023029591 |
| *Larp4b* | -1.023686931 | 0.00467774 | *Anapc13* | -1.258152184 | 0.037049091 | *Prkd2* | -1.52118659 | 0.023029591 |
| *Asb3* | -1.023686931 | 0.02208505 | *Alg6* | -1.258152184 | 0.040400806 | *Smndc1* | -1.52118659 | 0.023199876 |
| *Dynlt2a2* | -1.023686931 | 0.023100807 | *H1f4* | -1.258152184 | 0.040400806 | *Ppie* | -1.52118659 | 0.02423043 |
| *Tmem50a* | -1.023686931 | 0.027435159 | *Erh* | -1.258152184 | 0.041358802 | *Ndn* | -1.52118659 | 0.027283186 |
| *Etfa* | -1.029333494 | 0.026251962 | *Gm17106* | -1.258152184 | 0.051689182 | *Gm26618* | -1.52118659 | 0.03669912 |
| *Rspry1* | -1.029333494 | 0.041355388 | *Slc6a11* | -1.270643129 | 0.011122869 | *Penk* | -1.52118659 | 0.036964699 |
| *Usp40* | -1.043139293 | 0.031055256 | *Capza2* | -1.277261007 | 0.004866373 | *Gpatch4* | -1.52118659 | 0.038859892 |
| *Eif3m* | -1.043139293 | 0.05102131 | *Vps4b* | -1.280178491 | 0.013853065 | *Lap3* | -1.52118659 | 0.038859892 |
| *Ide* | -1.043139293 | 0.05102131 | *Gm35612* | -1.280178491 | 0.028481938 | *Tmem170* | -1.52118659 | 0.038859892 |
| *E530011L22Rik* | -1.051701307 | 0.045606994 | *Mat2a* | -1.288209418 | 0.017626741 | *Mkx* | -1.52118659 | 0.038859892 |
| *Slbp* | -1.051701307 | 0.053037578 | *Gm16599* | -1.289861044 | 0.004554447 | *Rmi1* | -1.52118659 | 0.039756778 |
| *Fuom* | -1.051701307 | 0.053037578 | *Sec61a2* | -1.293776094 | 0.030188898 | *Snrpb2* | -1.52118659 | 0.049677094 |
| *Ppid* | -1.061754972 | 0.030587014 | *Abcg1* | -1.298794169 | 0.028650208 | *Mrpl2* | -1.52118659 | 0.049677094 |
| *Map1lc3b* | -1.06949062 | 0.001180953 | *Kazald1* | -1.298794169 | 0.036041826 | *Purb* | -1.557712466 | 0.000684639 |
| *Thap2* | -1.073727613 | 0.027760989 | *Ccdc34* | -1.298794169 | 0.053563047 | *Ndufa5* | -1.568492305 | 0.007944067 |
| *Pitx2* | -1.073727613 | 0.032311658 | *Creb1* | -1.298794169 | 0.054681924 | *Chrac1* | -1.57365401 | 0.005301216 |
| *Tent4a* | -1.073727613 | 0.040242503 | *Dock11* | -1.305457899 | 0.007727352 | *Arfip1* | -1.588300786 | 0.034797189 |
| *Spsb1* | -1.073727613 | 0.046691541 | *Fbxo33* | -1.305457899 | 0.047089092 | *Cryl1* | -1.599189102 | 0.017455749 |
| *Aamp* | -1.073727613 | 0.054682082 | *Ddt* | -1.328541512 | 0.015892378 | *Fig4* | -1.599189102 | 0.032487835 |
| *Map6* | -1.088227183 | 0.025744262 | *Tafazzin* | -1.328541512 | 0.053157055 | *Cog7* | -1.614295995 | 0.005664289 |
| *Idnk* | -1.088227183 | 0.029514677 | *Cd99l2* | -1.334773466 | 0.007573397 | *Jade2* | -1.614295995 | 0.014196683 |
| *Fut8* | -1.098495518 | 0.004517912 | *Mapre3* | -1.338322533 | 0.010508432 | *Krt8* | -1.614295995 | 0.022772548 |
| *Psmb10* | -1.106149091 | 0.019758134 | *Mta3* | -1.338322533 | 0.012103336 | *Gpatch11* | -1.614295995 | 0.023199876 |
| *Lyrm9* | -1.106149091 | 0.019758134 | *Thra* | -1.351261589 | 0.00501901 | *Mindy4b-ps* | -1.624280083 | 0.014729346 |
| *Mrpl48* | -1.106149091 | 0.037008151 | *Pik3r4* | -1.351261589 | 0.042872463 | *Ubxn1* | -1.636663808 | 0.002827307 |
| *Acad8* | -1.106149091 | 0.045381115 | *Prdx4* | -1.360721918 | 0.001825352 | *Slc39a3* | -1.636663808 | 0.014587506 |
| *Wasf2* | -1.120648661 | 0.027050637 | *Mrpl30* | -1.365067388 | 0.004865791 | *Bik* | -1.636663808 | 0.014587506 |
| *Gnai3* | -1.128869168 | 0.01233578 | *Wdfy2* | -1.376796681 | 0.006761035 | *E4f1* | -1.636663808 | 0.024128575 |
| *Scamp2* | -1.128869168 | 0.034542694 | *Edem2* | -1.376796681 | 0.028200034 | *Eif3b* | -1.636663808 | 0.036006656 |
| *Fdx1* | -1.137857951 | 0.017564956 | *Ufm1* | -1.376796681 | 0.028200034 | *Bcl2l2* | -1.636663808 | 0.036006656 |
| *Polr3h* | -1.137857951 | 0.02134019 | *Ffar4* | -1.376796681 | 0.043310634 | *Hbp1* | -1.652431124 | 0.017480048 |
| *Prdm10* | -1.137857951 | 0.0256958 | *Rapgef4os1* | -1.376796681 | 0.052663583 | *Timp3* | -1.658690114 | 0.005225561 |
| *Phax* | -1.137857951 | 0.025713149 | *Ypel3* | -1.383683067 | 0.001292564 | *Cibar2* | -1.673189684 | 0.008076011 |
| *Tdrd3* | -1.137857951 | 0.037297825 | *Acot7* | -1.383683067 | 0.003214275 | *Akr7a5* | -1.673189684 | 0.038961702 |
| *H2az1* | -1.141338519 | 0.004649212 | *Cfap68* | -1.395655708 | 0.003966069 | *Gm11508* | -1.673189684 | 0.038961702 |
| *Trim9* | -1.158616511 | 0.012154848 | *Dnajc12* | -1.395655708 | 0.01204505 | *Acot13* | -1.673189684 | 0.038961702 |
| *Yaf2* | -1.158616511 | 0.012154848 | *Catsperg1* | -1.395655708 | 0.040078326 | *Anp32e* | -1.701758836 | 0.012615338 |
| *Clcn4* | -1.158616511 | 0.052360541 | *Stard10* | -1.395655708 | 0.040078326 | *Add3* | -1.701758836 | 0.014034265 |
| *Eif3e* | -1.180149672 | 0.007540234 | *Mrps18b* | -1.395655708 | 0.040078326 | *Ankle2* | -1.701758836 | 0.019905814 |
| *Arhgap1* | -1.184151603 | 0.009239656 | *Eif3j2* | -1.395655708 | 0.040078326 | *Smpd1* | -1.701758836 | 0.019905814 |
| *Galnt1* | -1.189980682 | 0.017425997 | *Smad2* | -1.395655708 | 0.040290661 | *G6pdx* | -1.701758836 | 0.020183305 |
| *Xkr7* | -1.199258495 | 0.024332657 | *Atf7ip* | -1.4033501 | 0.005519624 | *Churc1* | -1.701758836 | 0.031593475 |
| *Washc2* | -1.199258495 | 0.025155795 | *Hdc* | -1.421650917 | 0.03710122 | *Dmtf1* | -1.743579012 | 0.022239944 |
| *Cdcp1* | -1.199258495 | 0.053437856 | *Gm12655* | -1.421650917 | 0.03710122 | *Emc4* | -1.743579012 | 0.035617103 |
| *Opa1* | -1.216332009 | 0.017548244 | *Ecsit* | -1.421650917 | 0.03710122 | *Sgce* | -1.76219469 | 0.00141901 |
| *Soga1* | -1.216332009 | 0.023932135 | *Aamdc* | -1.43872443 | 0.032599493 | *Tmem29* | -1.766299088 | 0.000453228 |
| *Rtl8a* | -1.216332009 | 0.054473654 | *Sergef* | -1.450797262 | 0.018126535 | *Prr13* | -1.784220996 | 0.007825138 |
| *Rubcn* | -1.225730707 | 0.05456364 | *Dnttip1* | -1.450797262 | 0.05177527 | *Zcchc10* | -1.810693207 | 0.038619879 |
| *Ilf3* | -1.231679973 | 0.020197877 | *Mmd* | -1.450797262 | 0.05177527 | *2810001G20Rik* | -1.810693207 | 0.038619879 |
| *Rnf6* | -1.235784371 | 0.007940464 | *Sh3bgrl3* | -1.459786046 | 0.011843342 | *Ptpmt1* | -1.843114685 | 0.008674787 |
| *Cbx3* | -1.235784371 | 0.034324267 | *Sap18* | -1.459786046 | 0.023477079 | *2510002D24Rik* | -1.843114685 | 0.013640315 |
| *Stpg2* | -1.23878686 | 0.003749303 | *Map1lc3a* | -1.459786046 | 0.028287291 | *Ppp1r7* | -1.843114685 | 0.02199235 |

| **Supplemental Table S2D DEGS SOM2 Cluster; VCD/Vehicle.** Blue=downregulated | | | | | | | | |
| --- | --- | --- | --- | --- | --- | --- | --- | --- |
| **gene** | **avg_log2FC** | **p_val** | **gene** | **avg_log2FC** | **p_val** | **gene** | **avg_log2FC** | **p_val** |
| *Alg5* | -1.843114685 | 0.02199235 | *5031434O11Rik* | -2.258152184 | 0.049803716 | *Polr1has* | -2.52118659 | 0.028031012 |
| *Nek4* | -1.843114685 | 0.02199235 | *Glmp* | -2.258152184 | 0.049803716 | *C130036L24Rik* | -2.52118659 | 0.049801953 |
| *Yif1a* | -1.843114685 | 0.02199235 | *Gm17690* | -2.258152184 | 0.049803716 | *Impa1* | -2.52118659 | 0.049801953 |
| *Tmf1* | -1.843114685 | 0.022239944 | *Hmgcs2* | -2.258152184 | 0.049803716 | *Gbp3* | -2.52118659 | 0.049801953 |
| *H1f2* | -1.843114685 | 0.035229938 | *B230208B08Rik* | -2.258152184 | 0.049803716 | *Ncmap* | -2.52118659 | 0.049801953 |
| *Mtln* | -1.862223508 | 0.007712191 | *Nsun7* | -2.258152184 | 0.049803716 | *Rflna* | -2.52118659 | 0.049801953 |
| *Selenoh* | -1.874823545 | 0.001685826 | *0610040F04Rik* | -2.258152184 | 0.049803716 | *Parp11* | -2.52118659 | 0.049801953 |
| *Nsmce1* | -1.88375667 | 0.000731911 | *Zswim9* | -2.258152184 | 0.049803716 | *Coa4* | -2.52118659 | 0.049801953 |
| *Sf3a3* | -1.93622409 | 0.007785819 | *Igflr1* | -2.258152184 | 0.049803716 | *Swap70* | -2.52118659 | 0.049801953 |
| *Parva* | -1.93622409 | 0.003115615 | *Gm27252* | -2.258152184 | 0.049803716 | *Gm26713* | -2.52118659 | 0.049801953 |
| *Taf12* | -1.93622409 | 0.007898775 | *Ccrl2* | -2.258152184 | 0.049803716 | *Zfp830* | -2.52118659 | 0.049801953 |
| *Tob2* | -1.93622409 | 0.008313515 | *Pes1* | -2.258152184 | 0.049803716 | *AA465934* | -2.52118659 | 0.049801953 |
| *Coq10a* | -1.93622409 | 0.008600529 | *Gm57353* | -2.258152184 | 0.049803716 | *Arsg* | -2.52118659 | 0.049801953 |
| *Serp1* | -1.93622409 | 0.010317627 | *Inca1* | -2.258152184 | 0.049803716 | *Tpmt* | -2.52118659 | 0.049801953 |
| *Cacng2* | -1.93622409 | 0.014221865 | *Mrm3* | -2.258152184 | 0.049803716 | *Hic2* | -2.52118659 | 0.049801953 |
| *Hdac11* | -1.93622409 | 0.019630765 | *Sgsh* | -2.258152184 | 0.049803716 | *Crim1* | -2.52118659 | 0.049801953 |
| *Man1c1* | -1.93622409 | 0.02322348 | *Gm26839* | -2.258152184 | 0.049803716 | *Pcdhb19* | -2.52118659 | 0.049801953 |
| *Gm44129* | -1.93622409 | 0.02322348 | *Gm34961* | -2.258152184 | 0.049803716 | *Gm50383* | -2.52118659 | 0.049801953 |
| *Ccdc85a* | -1.93622409 | 0.02322348 | *Dpys* | -2.258152184 | 0.049803716 | *Gdpd2* | -2.52118659 | 0.049801953 |
| *Dgcr6* | -1.93622409 | 0.02322348 | *Zfp472* | -2.258152184 | 0.049803716 | *Zswim5* | -2.599189102 | 0.008012334 |
| *Med19* | -1.93622409 | 0.037761506 | *Taf8* | -2.258152184 | 0.049803716 | *Ppm1d* | -2.636663808 | 0.012652375 |
| *Exosc8* | -1.93622409 | 0.037761506 | *Gm10549* | -2.258152184 | 0.049803716 | *Plek* | -2.636663808 | 0.037392649 |
| *Lrguk* | -1.93622409 | 0.037761506 | *Gm56660* | -2.258152184 | 0.049803716 | *Zfp579* | -2.743579012 | 0.01593817 |
| *Rassf3* | -1.93622409 | 0.037761506 | *Nkrf* | -2.258152184 | 0.049803716 | *Clec11a* | -2.743579012 | 0.01593817 |
| *Septin4* | -1.93622409 | 0.037761506 | *Rap2c* | -2.258152184 | 0.049803716 | *Mef2b* | -2.743579012 | 0.01593817 |
| *Pelo* | -1.93622409 | 0.037761506 | *Gpr165* | -2.258152184 | 0.049803716 | *Nt5c3b* | -2.743579012 | 0.01593817 |
| *Tmem8b* | -1.93622409 | 0.038309572 | *Rai2* | -2.258152184 | 0.049803716 | *Btbd6* | -2.743579012 | 0.01593817 |
| *Nck2* | -1.986850163 | 0.006544575 | *Xpot* | -2.351261589 | 0.004887211 | *Plin3* | -2.743579012 | 0.01593817 |
| *Itgav* | -1.995117779 | 0.049219257 | *D6Wsu163e* | -2.395655708 | 0.021740288 | *Avpi1* | -2.743579012 | 0.01593817 |
| *Abhd12* | -2.023686931 | 0.005038131 | *Cep44* | -2.395655708 | 0.021740288 | *Dars2* | -2.743579012 | 0.028028583 |
| *Tmem14c* | -2.023686931 | 0.008313515 | *9330179D12Rik* | -2.395655708 | 0.021844084 | *1700037H04Rik* | -2.743579012 | 0.028028583 |
| *Ttc39b* | -2.023686931 | 0.00852647 | *Prkra* | -2.395655708 | 0.037215378 | *Pole3* | -2.743579012 | 0.028028583 |
| *Ppp4c* | -2.023686931 | 0.013330214 | *Sntb2* | -2.395655708 | 0.037215378 | *Gm39103* | -2.743579012 | 0.028028583 |
| *1700085D07Rik* | -2.023686931 | 0.013330214 | *Tom1l1* | -2.395655708 | 0.037392649 | *Gm56541* | -2.743579012 | 0.028028583 |
| *Chac2* | -2.051701307 | 0.004558742 | *C230014O12Rik* | -2.395655708 | 0.053673844 | *Tex49* | -2.743579012 | 0.028028583 |
| *AV356131* | -2.051701307 | 0.008381179 | *Etl4* | -2.43872443 | 0.022651572 | *Zfp414* | -2.743579012 | 0.028028583 |
| *Gm52870* | -2.051701307 | 0.023032446 | *Yeats4* | -2.52118659 | 0.004336921 | *Gm42705* | -2.743579012 | 0.049803275 |
| *Cox10* | -2.051701307 | 0.023032446 | *Cd274* | -2.52118659 | 0.012652375 | *Zfp626* | -2.743579012 | 0.049803275 |
| *Enkur* | -2.051701307 | 0.038000169 | *Cd9* | -2.52118659 | 0.013440221 | *Tmc3* | -2.743579012 | 0.049803275 |
| *Prpf4* | -2.051701307 | 0.038000169 | *Mettl14* | -2.52118659 | 0.021636129 | *Abhd13* | -2.743579012 | 0.049803275 |
| *Gm17491* | -2.051701307 | 0.038000169 | *Sumf2* | -2.52118659 | 0.028031012 | *Gm10509* | -2.743579012 | 0.049803275 |
| *Nudt13* | -2.051701307 | 0.038309572 | *Gper1* | -2.52118659 | 0.028031012 | *Ghr* | -2.843114685 | 0.037037722 |
| *Rprm* | -2.051701307 | 0.038619879 | *Elmod3* | -2.52118659 | 0.028031012 | *Zfand2b* | -2.93622409 | 0.009122654 |
| *Mtg1* | -2.106149091 | 0.021961214 | *Gm16315* | -2.52118659 | 0.028031012 | *Ctxn2* | -2.93622409 | 0.009122654 |
| *Pi4k2b* | -2.106149091 | 0.037569526 | *Ftl1-ps1* | -2.52118659 | 0.028031012 | *Igdcc3* | -2.93622409 | 0.009122654 |
| *Lrch4* | -2.106149091 | 0.037569526 | *Ropn1l* | -2.52118659 | 0.028031012 | *Kif26b* | -2.93622409 | 0.015935446 |
| *Snhg10* | -2.106149091 | 0.037569526 | *Nit2* | -2.52118659 | 0.028031012 | *Nol6* | -2.93622409 | 0.015935446 |
| *Serinc5* | -2.106149091 | 0.037569526 | *5031434O11Rik* | -2.258152184 | 0.049803716 | *Atpaf1* | -2.93622409 | 0.015935446 |
| *Lmo1* | -2.158616511 | 0.013788605 | *Glmp* | -2.258152184 | 0.049803716 | *Pak4* | -2.93622409 | 0.015935446 |
| *Lrrc14* | -2.158616511 | 0.022841897 | *Gm17690* | -2.258152184 | 0.049803716 | *Ppfia3* | -2.93622409 | 0.015935446 |
| *Rab4a* | -2.258152184 | 0.008223347 | *Hmgcs2* | -2.258152184 | 0.049803716 | *Snai2* | -2.93622409 | 0.015935446 |
| *Ogfod3* | -2.258152184 | 0.008223347 | *B230208B08Rik* | -2.258152184 | 0.049803716 | *Gm56812* | -2.93622409 | 0.02803004 |
| *Arhgef4* | -2.258152184 | 0.049803716 | *Nsun7* | -2.258152184 | 0.049803716 | *Crtap* | -2.93622409 | 0.02803004 |
| *Gm16150* | -2.258152184 | 0.049803716 | *0610040F04Rik* | -2.258152184 | 0.049803716 | *Rpa1* | -2.93622409 | 0.02803004 |
| *Gm57184* | -2.258152184 | 0.049803716 | *Zswim9* | -2.258152184 | 0.049803716 | *Grk6* | -2.93622409 | 0.02803004 |
| *Traf5* | -2.258152184 | 0.049803716 | *Igflr1* | -2.258152184 | 0.049803716 | *Alg1* | -2.93622409 | 0.02803004 |
| *Cd59b* | -2.258152184 | 0.049803716 | *Gm27252* | -2.258152184 | 0.049803716 | *Nubp2* | -2.93622409 | 0.02803004 |

| **Supplemental Table S2E DEGS SOM2 Cluster; VCD/Vehicle.** Blue=downregulated | | | | | | | | |
| --- | --- | --- | --- | --- | --- | --- | --- | --- |
| **gene** | **avg_log2FC** | **p_val** | **gene** | **avg_log2FC** | **p_val** | **gene** | **avg_log2FC** | **p_val** |
| *4930503L19Rik* | -2.93622409 | 0.02803004 |  |  |  |  |  |  |
| *Ush2a* | -2.93622409 | 0.049803275 |  |  |  |  |  |  |
| *Nhlrc3* | -2.93622409 | 0.049803275 |  |  |  |  |  |  |
| *Pnpla3* | -2.93622409 | 0.049803275 |  |  |  |  |  |  |
| *9630014M24Rik* | -2.93622409 | 0.049803275 |  |  |  |  |  |  |
| *Gm17130* | -3.106149091 | 0.015936808 |  |  |  |  |  |  |
| *Zfp708* | -3.106149091 | 0.015936808 |  |  |  |  |  |  |
| *Shank3* | -3.106149091 | 0.015936808 |  |  |  |  |  |  |
| *Dock8* | -3.106149091 | 0.015936808 |  |  |  |  |  |  |
| *0610009E02Rik* | -3.106149091 | 0.028030769 |  |  |  |  |  |  |
| *Krt10* | -3.258152184 | 0.009119952 |  |  |  |  |  |  |
| *Gm56764* | -3.258152184 | 0.015937625 |  |  |  |  |  |  |
| *Lhfpl2* | -3.395655708 | 0.007186396 |  |  |  |  |  |  |
|  |  |  |  |  |  |  |  |  |
|  |  |  |  |  |  |  |  |  |
|  |  |  |  |  |  |  |  |  |
|  |  |  |  |  |  |  |  |  |
|  |  |  |  |  |  |  |  |  |
|  |  |  |  |  |  |  |  |  |
|  |  |  |  |  |  |  |  |  |
|  |  |  |  |  |  |  |  |  |
|  |  |  |  |  |  |  |  |  |
|  |  |  |  |  |  |  |  |  |
|  |  |  |  |  |  |  |  |  |
|  |  |  |  |  |  |  |  |  |
|  |  |  |  |  |  |  |  |  |
|  |  |  |  |  |  |  |  |  |
|  |  |  |  |  |  |  |  |  |
|  |  |  |  |  |  |  |  |  |
|  |  |  |  |  |  |  |  |  |
|  |  |  |  |  |  |  |  |  |
|  |  |  |  |  |  |  |  |  |
|  |  |  |  |  |  |  |  |  |
|  |  |  |  |  |  |  |  |  |
|  |  |  |  |  |  |  |  |  |
|  |  |  |  |  |  |  |  |  |
|  |  |  |  |  |  |  |  |  |
|  |  |  |  |  |  |  |  |  |

Supplemental Table 2. Differentially Expressed Genes (DEGs) in SOM2 cluster following VCD treatment. Yellow highlight indicates specific genes of interest.

| **Supplemental Table S3A DEGS SOM3 Cluster; VCD/Vehicle.** Maroon=upregulated; Blue=downregulated | | | | | | | | |
| --- | --- | --- | --- | --- | --- | --- | --- | --- |
| **gene** | **avg_log2FC** | **p_val** | **gene** | **avg_log2FC** | **p_val** | **gene** | **avg_log2FC** | **p_val** |
| *Polr2i* | 4.178337241 | 0.003555983 | *Rpl9-ps6* | 1.856409146 | 0.01596629 | *Crip1* | -1.094681253 | 0.029946992 |
| *Pbxip1* | 3.663764068 | 0.010601468 | *Zscan26* | 1.856409146 | 0.026847972 | *Nudc* | -1.143590854 | 0.039528227 |
| *Cdk2ap2* | 3.663764068 | 0.018340613 | *Cmc2* | 1.856409146 | 0.044413217 | *Rab6a* | -1.143590854 | 0.041790944 |
| *Fbxl15* | 3.663764068 | 0.031846241 | *Sdf2* | 1.856409146 | 0.044758483 | *Rora* | -1.174617749 | 0.006788763 |
| *1110038F14Rik* | 3.556848865 | 0.00613978 | *Eef1akmt2* | 1.856409146 | 0.04482886 | *Lrp1b* | -1.198038638 | 0.001899283 |
| *Ccdc47* | 3.441371647 | 0.006137738 | *Kcnk1* | 1.730878264 | 0.027100438 | *Polr2f* | -1.198038638 | 0.041239468 |
| *Cir1* | 3.441371647 | 0.01834 | *C1galt1c1* | 1.730878264 | 0.027100438 | *Btg2* | -1.203891364 | 0.00012421 |
| *Nucb1* | 3.441371647 | 0.018343065 | *Ptprn* | 1.663764068 | 0.022742801 | *Rian* | -1.221593366 | 0.019334286 |
| *Arhgap17* | 3.441371647 | 0.018343065 | *Ndufa3* | 1.663764068 | 0.046227257 | *Ptprd* | -1.231053695 | 9.74E-05 |
| *Stx5a* | 3.441371647 | 0.031839796 | *Cenpb* | 1.593374741 | 0.0423524 | *Mrpl30* | -1.281094377 | 0.029459935 |
| *Creb3l2* | 3.315840765 | 0.010601821 | *Papola* | 1.593374741 | 0.0423524 | *Lsamp* | -1.321928095 | 0.001175238 |
| *Ube2q2* | 3.315840765 | 0.010601821 | *Cct8* | 1.593374741 | 0.0423524 | *Crhbp* | -1.419225296 | 0.029666098 |
| *Arhgap5* | 3.178337241 | 0.018340613 | *Nr2f6* | 1.593374741 | 0.045672536 | *Nrxn1* | -1.450252192 | 0.000363296 |
| *Ss18* | 3.178337241 | 0.018340613 | *Ube2e3* | 1.534481051 | 0.018688634 | *Edf1* | -1.522102477 | 0.019794264 |
| *Sdhd* | 3.178337241 | 0.031850537 | *1110065P20Rik* | 1.534481051 | 0.047759586 | *Sap30l* | -1.558628353 | 0.022967598 |
| *Abcf1* | 3.178337241 | 0.031850537 | *Ndufa2* | 1.473080507 | 0.008832715 | *Txnip* | -1.558628353 | 0.042089805 |
| *Naa35* | 3.026334148 | 0.031839796 | *Tmem176a* | 1.473080507 | 0.010731072 | *Rere* | -1.558628353 | 0.042089805 |
| *Csnk2a1* | 3.026334148 | 0.031849463 | *Rexo2* | 1.441371647 | 0.044913258 | *Ormdl3* | -1.558628353 | 0.042089805 |
| *Snx17* | 3.026334148 | 0.031849463 | *Mettl26* | 1.441371647 | 0.046093324 | *Sugt1* | -1.591049831 | 0.007200622 |
| *Mark3* | 3.026334148 | 0.031849463 | *Mcrip1* | 1.370982319 | 0.045838864 | *Angpt1* | -1.603022472 | 0.042103784 |
| *Bad* | 3.026334148 | 0.031849463 | *Tmem258* | 1.289368554 | 0.015995093 | *Pde7b* | -1.610716864 | 2.02E-05 |
| *Irak1* | 3.026334148 | 0.031849463 | *Selenos* | 1.285252445 | 0.016126155 | *Norad* | -1.617522042 | 0.028097862 |
| *Pja1* | 2.856409146 | 0.018330804 | *Bbln* | 1.178337241 | 0.028404799 | *Sorl1* | -1.629017681 | 0.021152816 |
| *Exosc4* | 2.856409146 | 0.024118459 | *Ndufb1* | 1.15186503 | 0.022946862 | *Clic1* | -1.728553354 | 0.02053382 |
| *Prpf4b* | 2.856409146 | 0.024358092 | *Krtcap2* | 1.078801568 | 0.034604116 | *Papss1* | -1.728553354 | 0.022504038 |
| *Stxbp1* | 2.856409146 | 0.031846241 | *Sec61g* | 1.032731919 | 0.000410518 | *Arih1* | -1.728553354 | 0.036473834 |
| *Chn1* | 2.856409146 | 0.031846241 | *Rplp2* | 0.952061385 | 5.53E-06 | *Rnf167* | -1.728553354 | 0.041670978 |
| *Polr3gl* | 2.856409146 | 0.031846241 | *Srp9* | 0.926798474 | 0.053519144 | *Mrpl28* | -1.844030572 | 0.035202861 |
| *S100pbp* | 2.856409146 | 0.031846241 | *Rpl39* | 0.91085693 | 0.000182654 | *Hnrnpdl* | -1.859797888 | 0.020181078 |
| *Ruvbl2* | 2.856409146 | 0.031846241 | *Atp5md* | 0.908876566 | 0.017093219 | *Spg21* | -1.880556448 | 0.041252042 |
| *Orai3* | 2.856409146 | 0.031846241 | *Rpl22* | 0.837043822 | 0.009549948 | *Lamp1* | -1.921198432 | 0.013884283 |
| *Dio2* | 2.856409146 | 0.031846241 | *Myl6* | 0.83678034 | 0.044673102 | *Phf21a* | -1.950945776 | 0.041676533 |
| *Syp* | 2.856409146 | 0.031846241 | *Dad1* | 0.836231264 | 0.015423149 | *Capza2* | -1.980092121 | 0.010960069 |
| *Yipf4* | 2.795008602 | 0.001813098 | *Nme2* | 0.801267592 | 0.026490603 | *Vapb* | -1.99158776 | 0.019671699 |
| *Tm4sf1* | 2.763299742 | 0.024128552 | *Fosb* | 0.795008602 | 0.016770177 | *2810001A02Rik* | -1.99158776 | 0.025899941 |
| *Rab18* | 2.763299742 | 0.041122524 | *Jun* | 0.726773866 | 0.03086185 | *Nebl* | -2.018059972 | 0.022050266 |
| *Tmed9* | 2.663764068 | 0.001918943 | *Rps18* | 0.666584587 | 0.001295727 | *Dusp3* | -2.018059972 | 0.022050266 |
| *Spp1* | 2.663764068 | 0.031839796 | *Rpl37a* | 0.639753095 | 0.000738694 | *Lhfpl3* | -2.018059972 | 0.026749034 |
| *Skic8* | 2.663764068 | 0.031839796 | *Gas5* | 0.601028313 | 0.003880595 | *Trim2* | -2.018059972 | 0.04083396 |
| *Tpd52l1* | 2.663764068 | 0.031839796 | *Rps26* | 0.57261618 | 0.015321251 | *Slc38a1* | -2.018059972 | 0.04083396 |
| *Mzt2* | 2.663764068 | 0.031839796 | *Rps28* | 0.56321277 | 0.009261684 | *Fuom* | -2.050481449 | 0.019130388 |
| *Gm57403* | 2.663764068 | 0.04192559 | *Rpl38* | 0.560424319 | 0.036413381 | *Cct4* | -2.069590272 | 0.019271317 |
| *Abracl* | 2.593374741 | 0.02446332 | *Lncpint* | 0.556848865 | 0.034800413 | *Rock2* | -2.143590854 | 0.012016533 |
| *Twsg1* | 2.556848865 | 0.024597732 |  |  |  | *Atp2b1* | -2.143590854 | 0.040417671 |
| *Pitx1* | 2.556848865 | 0.041533392 | *Npm1* | -0.553466648 | 0.011407028 | *Hnrnpr* | -2.259068071 | 0.021596204 |
| *Polr2k* | 2.488677362 | 0.000756758 | *Rpl10a* | -0.553679137 | 0.019016622 | *Rps6ka2* | -2.259068071 | 0.039999579 |
| *Sphkap* | 2.441371647 | 0.015131931 | *Pbx1* | -0.60656283 | 0.011315748 | *Pdpk1* | -2.313515855 | 0.03916989 |
| *Rps19bp1* | 2.441371647 | 0.031850537 | *Prl* | -0.618813286 | 0.000188097 | *Dscam* | -2.365983275 | 0.002594175 |
| *Sytl4* | 2.315840765 | 0.025064481 | *Auts2* | -0.728553354 | 0.009926583 | *Cdkn2c* | -2.365983275 | 0.032201949 |
| *Stard3nl* | 2.315840765 | 0.04232424 | *Prkce* | -0.821662759 | 0.023274201 | *Plcb1* | -2.465518949 | 0.034511553 |
| *Slc25a39* | 2.10433666 | 0.016564398 | *Cox7a2l* | -0.880556448 | 0.031157373 | *Pdcd7* | -2.465518949 | 0.034511553 |
| *Ptges3* | 2.026334148 | 0.03172299 | *Atp5a1* | -0.89566334 | 0.032526913 | *Clasp2* | -2.465518949 | 0.034511553 |
| *Map1lc3a* | 2.026334148 | 0.042986887 | *Nalf1* | -0.95855896 | 0.006344025 | *Asrgl1* | -2.465518949 | 0.034511553 |
| *Atp5k* | 2.026334148 | 0.044758483 | *Nrg1* | -0.976480868 | 0.000648509 | *Mast4* | -2.465518949 | 0.03916989 |
| *Pmvk* | 1.971886364 | 0.026839277 | *Rbp4* | -1.018059972 | 0.028783955 | *Irs2* | -2.558628353 | 0.040407686 |
| *Syt7* | 1.971886364 | 0.044407773 | *Pdia6* | -1.054323516 | 0.027187973 | *Dohh* | -2.728553354 | 0.017720085 |
| *Commd6* | 1.943871988 | 0.016713892 | *Map1lc3b* | -1.08914307 | 0.054133071 | *Rragc* | -2.728553354 | 0.034505918 |

| **Supplemental Table S3B DEGS SOM3 Cluster; VCD/Vehicle.** Blue=downregulated | | | | | | | | |
| --- | --- | --- | --- | --- | --- | --- | --- | --- |
| **gene** | **avg_log2FC** | **p_val** | **gene** | **avg_log2FC** | **p_val** | **gene** | **avg_log2FC** | **p_val** |
| *Wdfy3* | -2.728553354 | 0.034505918 | *Cbr1* | -3.465518949 | 0.009161031 |  |  |  |
| *Zranb1* | -2.728553354 | 0.034505918 | *Cdk11b* | -3.465518949 | 0.017720085 |  |  |  |
| *6430548M08Rik* | -2.728553354 | 0.034505918 | *Ccdc90b* | -3.465518949 | 0.017720085 |  |  |  |
| *Tafa2* | -2.728553354 | 0.034505918 | *Odf2* | -3.465518949 | 0.034505918 |  |  |  |
| *Narf* | -2.728553354 | 0.034505918 | *Tmem106c* | -3.465518949 | 0.034505918 |  |  |  |
| *Sik1* | -2.728553354 | 0.034505918 | *Vcl* | -3.465518949 | 0.034511553 |  |  |  |
| *Atad1* | -2.728553354 | 0.034505918 | *Reln* | -3.535908276 | 0.019841981 |  |  |  |
| *Usp11* | -2.728553354 | 0.034505918 | *Ptk2* | -3.603022472 | 0.004751084 |  |  |  |
| *Fam50a* | -2.728553354 | 0.034505918 | *Tifa* | -3.603022472 | 0.009160664 |  |  |  |
| *Fam199x* | -2.728553354 | 0.034505918 | *Yipf2* | -3.603022472 | 0.009162131 |  |  |  |
| *Hddc2* | -2.728553354 | 0.037988758 | *Camk2d* | -3.728553354 | 0.004752222 |  |  |  |
| *Thrb* | -2.728553354 | 0.037988758 | *Smg6* | -3.728553354 | 0.009160664 |  |  |  |
| *Cited2* | -2.844030572 | 0.012985885 | *Dmxl1* | -3.728553354 | 0.009160664 |  |  |  |
| *Ptprk* | -2.844030572 | 0.017442282 | *Bambi* | -3.728553354 | 0.017712948 |  |  |  |
| *Arglu1* | -2.950945776 | 0.017712948 | *Gm1604a* | -3.728553354 | 0.017719371 |  |  |  |
| *Galntl6* | -2.950945776 | 0.017712948 | *Kxd1* | -3.844030572 | 0.004752792 |  |  |  |
| *Akr1c14* | -2.950945776 | 0.017712948 | *Nrd1* | -3.950945776 | 0.002467214 |  |  |  |
| *Wdr36* | -2.950945776 | 0.017712948 | *Rhot1* | -3.950945776 | 0.002467214 |  |  |  |
| *Gria2* | -2.950945776 | 0.034510144 | *A430005L14Rik* | -3.950945776 | 0.004750894 |  |  |  |
| *Ksr2* | -2.950945776 | 0.034510144 | *Stat3* | -3.950945776 | 0.004752981 |  |  |  |
| *Sh3gl3* | -2.950945776 | 0.034510144 | *Meis2* | -4.050481449 | 0.034510144 |  |  |  |
| *Grm5* | -2.950945776 | 0.034510144 | *Kcnb2* | -4.465518949 | 0.0012798 |  |  |  |
| *Katnip* | -2.950945776 | 0.034510144 |  |  |  |  |  |  |
| *Rnf170* | -2.950945776 | 0.034510144 |  |  |  |  |  |  |
| *Pfkl* | -2.950945776 | 0.034510144 |  |  |  |  |  |  |
| *Tmcc3* | -2.950945776 | 0.034510144 |  |  |  |  |  |  |
| *Rdh14* | -2.950945776 | 0.034510144 |  |  |  |  |  |  |
| *Dnaaf2* | -2.950945776 | 0.034510144 |  |  |  |  |  |  |
| *Trerf1* | -2.950945776 | 0.034510144 |  |  |  |  |  |  |
| *Ccs* | -2.950945776 | 0.034510144 |  |  |  |  |  |  |
| *Znhit2* | -2.950945776 | 0.034510144 |  |  |  |  |  |  |
| *Hax1* | -3.143590854 | 0.01771723 |  |  |  |  |  |  |
| *Dctn4* | -3.143590854 | 0.01771723 |  |  |  |  |  |  |
| *Igbp1* | -3.143590854 | 0.01771723 |  |  |  |  |  |  |
| *Ralgds* | -3.143590854 | 0.034505918 |  |  |  |  |  |  |
| *Dennd1b* | -3.143590854 | 0.034511553 |  |  |  |  |  |  |
| *Rxfp1* | -3.143590854 | 0.034511553 |  |  |  |  |  |  |
| *Spty2d1* | -3.143590854 | 0.034511553 |  |  |  |  |  |  |
| *2510046G10Rik* | -3.143590854 | 0.034511553 |  |  |  |  |  |  |
| *Nek9* | -3.143590854 | 0.034511553 |  |  |  |  |  |  |
| *Napg* | -3.143590854 | 0.034511553 |  |  |  |  |  |  |
| *Grik2* | -3.259068071 | 0.001584325 |  |  |  |  |  |  |
| *Mtif3* | -3.313515855 | 0.009158831 |  |  |  |  |  |  |
| *Hs2st1* | -3.313515855 | 0.017719371 |  |  |  |  |  |  |
| *Gm56999* | -3.313515855 | 0.017719371 |  |  |  |  |  |  |
| *Sos1* | -3.313515855 | 0.017719371 |  |  |  |  |  |  |
| *Map3k2* | -3.313515855 | 0.017719371 |  |  |  |  |  |  |
| *Herc3* | -3.313515855 | 0.034510144 |  |  |  |  |  |  |
| *Mat2a* | -3.313515855 | 0.034510144 |  |  |  |  |  |  |
| *Ccdc88a* | -3.313515855 | 0.034510144 |  |  |  |  |  |  |
| *Mgat1* | -3.313515855 | 0.034511553 |  |  |  |  |  |  |
| *Ptrh2* | -3.313515855 | 0.034511553 |  |  |  |  |  |  |
| *Cdc5l* | -3.313515855 | 0.034511553 |  |  |  |  |  |  |
| *Pianp* | -3.465518949 | 0.004749187 |  |  |  |  |  |  |
| *Nop14* | -3.465518949 | 0.009161031 |  |  |  |  |  |  |
| *Hipk2* | -3.465518949 | 0.009161031 |  |  |  |  |  |  |
| *Dusp26* | -3.465518949 | 0.009161031 |  |  |  |  |  |  |

Supplemental Table 3. Differentially Expressed Genes (DEGs) in SOM3 cluster following VCD treatment. Yellow highlight indicates specific genes of interest.

| **Supplemental Table S4. Differentially Expressed Genes Representing G Protein Receptor Canonical Pathway**  **in Somatotropes. VCD vs Vehicle Control females** | | | | | | | |
| --- | --- | --- | --- | --- | --- | --- | --- |
|  |  | **Somatotrope 1 (SOM1)** | | **Somatotrope 2 (SOM2)** | | **Somatotrope 3 (SOM3)** | |
|  | **Gene** | **Expr Log Ratio** | **Expr p-value** | **Expr Log Ratio** | **Expr p-value** | **Expr Log Ratio** | **Expr p-value** |
| adhesion G protein-coupled receptor L1 | ADGRL1 | 1.068 | 0.0143 |  |  |  |  |
| calcium/calmodulin dependent protein kinase II delta | CAMK2D |  |  |  |  | -3.729 | 0.00475 |
| C-C motif chemokine receptor like 2 | CCRL2 |  |  | -2.258 | 0.0498 |  |  |
| calcineurin like EF-hand protein 1 | CHP1 |  |  | 1.386 | 0.05 |  |  |
| cAMP responsive element binding protein 1 | CREB1 |  |  | -1.299 | 0.05 |  |  |
| cAMP responsive element binding protein 3 like 2 | CREB3L2 |  |  |  |  | 3.316 | 0.0106 |
| free fatty acid receptor 4 | FFAR4 |  |  | -1.377 | 0.0433 | -1.144 | 0.5 |
| glycerophosphodiester phosphodiesterase domain containing 3 | GDPD3 | -0.628 | 0.00824 |  |  |  |  |
| G protein subunit alpha i3 | GNAI3 |  |  | -1.129 | 0.0123 |  |  |
| G protein subunit gamma 4 | GNG4 |  |  | 1.248 | 0.0346 |  |  |
| G protein-coupled receptor kinase 6 | GRK6 |  |  | -2.936 | 0.028 |  |  |
| G protein-coupled estrogen receptor 1 | GPER1 |  |  | -2.521 | 0.028 |  |  |
| G protein-coupled receptor 137 | GPR137 | 2.206 | 0.00247 |  |  |  |  |
| G protein-coupled receptor 155 | GPR155 | 1.313 | 0.0182 |  |  |  |  |
| glutamate metabotropic receptor 5 | GRM5 | -1.379 | 0.03 |  |  | -2.951 | 0.0345 |
| hyperpolarization activated cyclic nucleotide gated potassium channel 1 | HCN1 | 3.206 | 0.0269 |  |  |  |  |
| hyperpolarization activated cyclic nucleotide gated potassium and sodium channel 2 | HCN2 | -2.964 | 0.015 |  |  |  |  |
| Jun proto-oncogene, AP-1 transcription factor subunit | JUN |  |  |  |  | 0.727 | 0.0309 |
| leucine rich repeat containing G protein-coupled receptor 4 | LGR4 | 0.621 | 0.0297 |  |  |  |  |
| lysophosphatidic acid receptor 3 | LPAR3 | 3.428 | 0.0316 |  |  |  |  |
| mitogen-activated protein kinase kinase 3 | MAP2K3 | 1.457 | 0.002 |  |  |  |  |
| mitogen-activated protein kinase kinase kinase 10 | MAP3K10 | -1.864 | 0.0104 |  |  |  |  |
| mitogen-activated protein kinase kinase kinase 2 | MAP3K2 |  |  |  |  | -3.314 | 0.0177 |
| myocyte enhancer factor 2B | MEF2B |  |  | -2.744 | 0.0159 |  |  |
| myocyte enhancer factor 2D | MEF2D |  |  | 0.846 | 0.0298 |  |  |
| metallophosphoesterase 1 | MPPE1 |  |  | 1.649 | 0.0159 |  |  |
| myosin light chain 6 | MYL6 |  |  |  |  | 0.837 | 0.0447 |
| nuclear factor kappa B subunit 2 | NFKB2 |  |  | 2.064 | 0.0494 |  |  |
| p21 (RAC1) activated kinase 1 | PAK1 | 0.685 | 0.0261 |  |  |  |  |
| p21 (RAC1) activated kinase 3 | PAK3 | 0.621 | 0.00393 |  |  |  |  |
| p21 (RAC1) activated kinase 4 | PAK4 |  |  | -2.936 | 0.0159 |  |  |
| phosphodiesterase 7B | PDE7B |  |  |  |  | -1.611 | 0.0000202 |
| 3-phosphoinositide dependent protein kinase 1 | PDPK1 |  |  |  |  | -2.314 | 0.0392 |
| phosphoinositide-3-kinase regulatory subunit 4 | PIK3R4 |  |  | -1.351 | 0.0429 |  |  |
| phospholipase C beta 1 | PLCB1 |  |  |  |  | -2.466 | 0.0345 |
| phosphatidylinositol-3,4,5-trisphosphate dependent Rac exchange factor 1 | PREX1 |  |  | 2.386 | 0.0403 |  |  |
| protein kinase cAMP-activated catalytic subunit beta | PRKACB |  |  | -0.839 | 0.042 |  |  |
| protein kinase C epsilon | PRKCE |  |  | -0.262 | 0.0126 |  | 0.0233 |
| protein tyrosine kinase 2 | PTK2 |  |  |  |  | -3.603 | 0.00475 |
| RAP1B, member of RAS oncogene family | RAP1B | 0.664 | 0.0267 | -0.749 | 0.0482 |  |  |
| ras related dexamethasone induced 1 | RASD1 | 0.779 | 0.00659 |  |  |  |  |
| REL proto-oncogene, NF-kB subunit | REL | 0.728 | 0.0365 |  |  |  |  |
| Rho associated coiled-coil containing protein kinase 2 | ROCK2 |  |  | 0.574 | 0.0434 | -2.144 | 0.012 |
| RAS related | RRAS | 2.08 | 0.0109 |  |  |  |  |
| relaxin family peptide receptor 1 | RXFP1 |  |  |  |  | -3.144 | 0.0345 |
| SIX homeobox 1 | SIX1 | -1.327 | 0.0458 |  |  |  |  |
| solute carrier family 52 member 2 | SLC52A2 | 2.621 | 0.0316 |  |  |  |  |
| SOS Ras/Rac guanine nucleotide exchange factor 1 | SOS1 |  |  |  |  | -3.314 | 0.0177 |
| somatostatin receptor 3 | SSTR3 | 1.469 | 0.05 |  |  |  |  |
| signal transducer and activator of transcription 3 | STAT3 | 0.698 | 0.0000177 |  |  | -3.951 | 0.00475 |
| tyrosyl-DNA phosphodiesterase 2 | TDP2 | 0.659 | 0.05 |  |  |  |  |

| **Supplemental Table S5A. Differentially Expressed Genes in Somatotropes That are E2 Targets**  **VCD vs Vehicle control females** | | | | | | | |
| --- | --- | --- | --- | --- | --- | --- | --- |
|  | | **Somatotrope 1** | | **Somatotrope 2** | | **Somatotrope 3** | |
|  | | **avg_log2FC** | **p_val** | **avg_log2FC** | **p_val** | **avg_log2FC** | **p_val** |
| acetyl-Coenzyme A acyltransferase 1A | Acaa1a | 1.469 | 0.0515 |  |  |  |  |
| acyl-CoA thioesterase 9 | ACOT9 |  |  | 2.234 | 0.0492 |  |  |
| AE binding protein 1 | AEBP1 | 1.399 | 0.0000187 | 0.963 | 0.00832 |  |  |
| Agrin | AGRN |  |  | 2.179 | 0.045 |  |  |
| aryl hydrocarbon receptor | AHR |  |  | 2.064 | 0.0494 |  |  |
| A-kinase anchoring protein 12 | AKAP12 | 0.962 | 0.0329 |  |  |  |  |
| 5'-aminolevulinate synthase 1 | ALAS1 |  |  | 2.523 | 0.00846 |  |  |
| aldehyde dehydrogenase 6 family member A1 | ALDH6A1 | -0.768 | 0.0277 |  |  |  |  |
| ALG1 chitobiosyldiphosphodolichol beta-mannosyltransferase | ALG1 |  |  | -2.936 | 0.028 |  |  |
| ALG5 dolichyl-phosphate beta-glucosyltransferase | ALG5 |  |  | -1.843 | 0.022 |  |  |
| angiopoietin 1 | ANGPT1 |  |  | -0.668 | 0.0122 | -1.603 | 0.0421 |
| anoctamin 1 | ANO1 | 2.621 | 0.0316 |  |  |  |  |
| androgen receptor | AR | 0.621 | 0.0522 |  |  |  |  |
| ARF GTPase 5 | ARF5 |  |  | -0.84 | 0.0023 |  |  |
| Rho GTPase activating protein 35 | ARHGAP35 | 0.665 | 0.0497 |  |  |  |  |
| Rho guanine nucleotide exchange factor 12 | ARHGEF12 | 0.6 | 0.0158 |  |  |  |  |
| arylsulfatase G | ARSG |  |  | -2.521 | 0.0498 |  |  |
| ATPase plasma membrane Ca2+ transporting1 | ATP2B1 |  |  |  |  | -2.144 | 0.0404 |
| activating transcription factor 3 | ATF3 | 1.109 | 0.000495 | 0.857 | 0.0136 |  |  |
| -- | ATPAF2 |  |  | 3.234 | 0.0118 |  |  |
| BMP and activin membrane bound inhibitor | BAMBI |  |  |  |  | -3.729 | 0.0177 |
| brain expressed X-linked 3 | BEX3 |  |  | -0.843 | 0.0145 |  |  |
| BICD cargo adaptor 1 | BICD1 |  |  | -0.831 | 0.00527 |  |  |
| BCL2 interacting killer | BIK |  |  | -1.637 | 0.0146 |  |  |
| BCL3 transcription coactivator | BCL3 | 1.206 | 0.0318 |  |  |  |  |
| BCS1 homolog, ubiquinol-cytochrome c reductase complex chaperone | BCS1L | -1.964 | 0.0265 |  |  |  |  |
| brain derived neurotrophic factor | BDNF | 1.461 | 0.00561 |  |  |  |  |
| Biglycan | BGN | 2.943 | 0.013 |  |  |  |  |
| BTG anti-proliferation factor 2 | BTG2 |  |  |  |  | -1.204 | 0.000124 |
| Cbl proto-oncogene | CBL |  |  | 1.523 | 0.0207 |  |  |
| chromobox 6 | CBX6 | -0.68 | 0.0543 |  |  |  |  |
| C-C motif chemokine ligand 25 | CCL25 |  |  | 2.064 | 0.0135 |  |  |
| cellular communication network factor 1 | CCN1 | 0.82 | 0.00964 |  |  |  |  |
| cyclin D3 | CCND3 | -0.69 | 0.0249 |  |  |  |  |
| CDC42 binding protein kinase alpha | CDC42BPA |  |  | -0.702 | 0.00518 |  |  |
| cyclin dependent kinase like 2 | CDKL2 | 0.817 | 0.0085 |  |  |  |  |
| CD274 molecule | CD274 |  |  | -2.521 | 0.0127 |  |  |
| cyclin dependent kinase inhibitor 1B | CDKN1B |  |  | -0.714 | 0.0314 |  |  |
| cerebellar degeneration related antigen 1, opposite strand | Cdr1os | -0.701 | 0.011 |  |  |  |  |
| checkpoint kinase 1 | CHEK1 |  |  | 2.386 | 0.0403 |  |  |
| Cbp/p300 interacting transactivator with Glu/Asp rich carboxy-terminal domain 2 | CITED2 |  |  |  |  | -2.844 | 0.013 |
| cytochrome c oxidase assembly factor 4 homolog | COA4 |  |  | -2.521 | 0.0498 |  |  |
| cAMP responsive element binding protein 1 | CREB1 |  |  | -1.299 | 0.0547 |  |  |
| cAMP responsive element binding protein 3 like 2 | CREB3L2 |  |  |  |  | 3.316 | 0.0106 |
| cysteine rich transmembrane BMP regulator 1 | CRIM1 |  |  | -2.521 | 0.0498 |  |  |
| crooked neck pre-mRNA splicing factor 1 | CRNKL1 | 1.177 | 0.00224 |  |  |  |  |
| cartilage acidic protein 1 | CRTAC1 | 4.08 | 0.000425 |  |  |  |  |
| cartilage associated protein | CRTAP |  |  | -2.936 | 0.028 |  |  |
| chromosome segregation 1 like | CSE1L | 2.165 | 0.00000418 |  |  |  |  |
| cystatin B | CSTB | 0.781 | 0.0391 |  |  |  |  |
| catenin delta 1 | CTNND1 | 0.643 | 0.0239 |  |  |  |  |
| cathepsin B | CTSB |  |  | -1.014 | 0.00871 |  |  |
| cathepsin K | CTSK | -2.186 | 0.0476 |  |  |  |  |
| C-X-C motif chemokine ligand 1 | Cxcl1 | 2.258 | 0.0164 |  |  |  |  |
| CXXC finger protein 4 | CXXC4 | -0.846 | 0.0103 | -0.936 | 0.103 |  |  |
| cytochrome P450 family 51 subfamily A member1 | CYP51A1 | 0.823 | 0.0236 | 2.134 | 0.00239 |  |  |
| drebrin 1 | DBN1 | -1.964 | 0.00308 |  |  |  |  |
| dimethylarginine dimethylaminohydrolase 1 | Ddah1 | 0.678 | 0.0029 |  |  |  |  |

| **Supplemental Table S5B. Differentially Expressed Genes in Somatotropes That are E2 Targets**  **VCD vs Vehicle control females** | | | | | | | |
| --- | --- | --- | --- | --- | --- | --- | --- |
|  | | **Somatotrope 1** | | **Somatotrope 2** | | **Somatotrope 3** | |
| **Gene** |  | **avg_log2FC** | **p_val** | **avg_log2FC** | **p_val** | **avg_log2FC** | **p_val** |
| diaphanous related formin 3 | DIAPH3 |  |  | -1.000 | 0.0485 |  |  |
| discs large MAGUK scaffold protein 4 | DLG4 | 0.714 | 0.00894 |  |  |  |  |
| DS cell adhesion molecule | DSCAM |  |  |  |  | -2.366 | 0.00259 |
| dual specificity phosphatase 3 | DUSP3 |  |  |  |  | -2.018 | 0.0221 |
| dual specificity phosphatase 5 | DUSP5 | 1.036 | 0.0114 |  |  |  |  |
| E2F transcription factor 3 | E2F3 | 1.136 | 0.0314 |  |  |  |  |
| E4F transcription factor 1 | E4F1 |  |  | -1.637 | 0.0241 |  |  |
| enoyl-CoA hydratase domain containing 2 | ECHDC2 | 1.999 | 0.0258 |  |  |  |  |
| early growth response 2 | EGR2 | 1.042 | 0.0000705 |  |  |  |  |
| early growth response 3 | EGR3 | 2.621 | 0.0316 |  |  |  |  |
| eukaryotic translation initiation factor 3 subunit J | EIF3J | 0.962 | 0.00626 |  |  |  |  |
| eukaryotic translation initiation factor 3 subunit B | EIF3B |  |  | -1.637 | 0.036 |  |  |
| ELAV like RNA binding protein 1 | ELAVL1 |  |  | 1.386 | 0.00472 |  |  |
| ETS transcription factor ELK3 | ELK3 |  |  | -0.829 | 0.0352 |  |  |
| ETS transcription factor ELK1 | ELK1 | 1.843 | 0.0385 |  |  |  |  |
| epithelial membrane protein 1 | EMP1 | 0.743 | 0.00864 |  |  |  |  |
| ectonucleotide pyrophosphatase/phosphodiesterase 2 | ENPP2 | 0.725 | 0.0309 |  |  |  |  |
| ERH mRNA splicing and mitosis factor | ERH |  |  | -1.258 | 0.0414 |  |  |
| erb-b2 receptor tyrosine kinase 3 | ERBB3 | 1.621 | 0.054 |  |  |  |  |
| ETS variant transcription factor 6 | ETV6 | 0.787 | 0.00737 |  |  |  |  |
| FosB proto-oncogene, AP-1 transcription factor subunit | FOSB |  |  |  |  | 0.795 | 0.0168 |
| follicle stimulating hormone subunit beta | FSHB | -1.379 | 0.0375 |  |  |  |  |
| fucosyltransferase 8 | FUT8 |  |  | -1.098 | 0.00452 |  |  |
| glucose-6-phosphate dehydrogenase | G6PD |  |  | -1.702 | 0.0202 |  |  |
| growth arrest and DNA damage inducible gamma | GADD45G | 0.707 | 0.0207 |  |  |  |  |
| polypeptide N-acetylgalactosaminyltransferase 1 | GALNT1 |  |  | -1.19 | 0.0174 |  |  |
| growth arrest specific 5 | GAS5 |  |  |  |  | 0.601 | 0.00388 |
| growth hormone receptor | GHR |  |  | -2.843 | 0.037 |  |  |
| galactosidase beta 1 | GLB1 | 0.962 | 0.0462 |  |  |  |  |
| GLI family zinc finger 2 | GLI2 | 0.712 | 0.0157 |  |  |  |  |
| glucosamine (N-acetyl)-6-sulfatase | GNS | 0.736 | 0.0206 |  |  |  |  |
| golgi SNAP receptor complex member 2 | GOSR2 | 1.151 | 0.00448 |  |  |  |  |
| G protein-coupled estrogen receptor 1 | GPER1 |  |  | -2.521 | 0.028 |  |  |
| growth factor receptor bound protein 10 | GRB10 | 0.68 | 0.0367 |  |  |  |  |
| glutamate metabotropic receptor 5 | GRM5 | -1.379 | 0.03 |  |  | -2.951 | 0.0345 |
| HMG-box transcription factor 1 | HBP1 |  |  | -1.652 | 0.0175 |  |  |
| hyperpolarization activated cyclic nucleotide gated potassium channel 1 | HCN1 | 3.206 | 0.0269 |  |  |  |  |
| histone deacetylase 11 | HDAC11 |  |  | -1.936 | 0.0196 |  |  |
| major histocompatibility complex, class I, A | HLA-A | 2.843 | 0.00368 |  |  |  |  |
| 3-hydroxy-3-methylglutaryl-CoA reductase | HMGCR | 0.894 | 0.00977 |  |  |  |  |
| 3-hydroxy-3-methylglutaryl-CoA synthase 1 | HMGCS1 | 1.411 | 0.00342 |  |  |  |  |
| heterogeneous nuclear ribonucleoprotein K | HNRNPK |  |  | -0.799 | 0.0162 |  |  |
| homer scaffold protein 1 | HOMER1 | 1.187 | 0.0151 |  |  |  |  |
| hippocalcin like 4 | HPCAL4 | -2.186 | 0.0476 |  |  |  |  |
| heat shock protein family D (Hsp60) member 1 | HSPD1 |  |  | -0.824 | 0.0529 |  |  |
| insulin degrading enzyme | IDE |  |  | -1.043 | 0.051 |  |  |
| interferon induced transmembrane protein 3 | Ifitm3 | 1.621 | 0.0547 |  |  |  |  |
| interferon related developmental regulator 1 | IFRD1 | 0.821 | 0.00408 |  |  |  |  |
| insulin like growth factor 2 receptor | IGF2R | 0.832 | 0.0025 |  |  |  |  |
| insulin like growth factor 1 | IGF1 |  |  | 3.386 | 0.0403 |  |  |
| interleukin enhancer binding factor 3 | ILF3 |  |  | -1.232 | 0.0202 |  |  |
| insulin induced gene 1 | INSIG1 |  |  | 1.649 | 0.0128 |  |  |
| integrin subunit alpha V | ITGAV | -1.338 | 0.0212 | -1.995 | 0.0492 |  |  |
| insulin receptor substrate 2 | IRS2 |  |  |  |  | -2.559 | 0.0404 |
| Jun proto-oncogene, AP-1 transcription factor subunit | JUN |  |  |  |  | 0.727 | 0.0309 |
| kazrin, periplakin interacting protein | KAZN | 1.116 | 0.00966 |  |  |  |  |
| kinesin family member 1A | KIF1A | 0.779 | 0.000457 |  |  |  |  |
| keratin 8 | KRT8 |  |  | -1.614 | 0.0228 |  |  |

| **Supplemental Table S5C. Differentially Expressed Genes in Somatotropes That are E2 Targets.   VCD Vs control females** | | | | | | | |
| --- | --- | --- | --- | --- | --- | --- | --- |
|  | | **Somatotrope 1** | | **Somatotrope 2** | | **Somatotrope 3** | |
| **Gene** |  | **avg_log2FC** | **p_val** | **avg_log2FC** | **p_val** | **avg_log2FC** | **p_val** |
| keratin 10 | KRT10 |  |  | -3.258 | 0.00912 |  |  |
| lysosomal associated membrane protein 2 | LAMP2 | 0.739 | 0.00102 |  |  |  |  |
| La ribonucleoprotein 6, translational regulator | LARP6 | -1.701 | 0.0368 |  |  |  |  |
| LHFPL tetraspan subfamily member 2 | LHFPL2 |  |  | -3.396 | 0.00719 |  |  |
| leucine rich repeat containing G protein-coupled receptor 4 | LGR4 | 0.621 | 0.0297 |  |  |  |  |
| lipin 1 | LPIN1 | 0.723 | 0.019 |  |  |  |  |
| LDL receptor related protein 1 | LRP1 | 1.621 | 0.0302 | 2.649 | 0.00844 |  |  |
| LDL receptor related protein 8 | LRP8 | 1.189 | 0.00405 |  |  |  |  |
| male germ cell associated kinase | MAK | -2.379 | 0.00877 |  |  |  |  |
| monoamine oxidase B | MAOB | 2.206 | 0.0381 |  |  |  |  |
| mitogen-activated protein kinase kinase 3 | MAP2K3 | 1.457 | 0.002 |  |  |  |  |
| minichromosome maintenance complex component 7 | MCM7 | -1.549 | 0.0236 |  |  |  |  |
| Meis homeobox 2 | MEIS2 |  |  |  |  | -4.05 | 0.0345 |
| alpha-1,3-mannosyl-glycoprotein 2-beta-N-acetylglucosaminyltransferase | MGAT1 |  |  |  |  | -3.314 | 0.0345 |
| monocyte to macrophage differentiation associated | MMD |  |  | -1.451 | 0.0518 |  |  |
| matrix metallopeptidase 14 | MMP14 |  |  | -0.936 | 0.039 |  |  |
| mitochondrial ribosomal protein S24 | MRPS24 | -0.853 | 0.0177 |  |  |  |  |
| methylsterol monooxygenase 1 | MSMO1 | 1.123 | 0.0307 |  |  |  |  |
| metallothionein 2A | MT2A | 0.977 | 0.0000516 |  |  |  |  |
| mitochondrial carrier 1 | MTCH1 | -0.669 | 0.00584 |  |  |  |  |
| myosin VB | MYO5B | -1.517 | 0.00144 |  |  |  |  |
| nuclear prelamin A recognition factor | NARF |  |  |  |  | -2.729 | 0.0345 |
| NADH:ubiquinone oxidoreductase subunit A3 | NDUFA3 |  |  |  |  | 1.664 | 0.0462 |
| nuclear receptor coactivator 6 | NCOA6 | -0.712 | 0.038 |  |  |  |  |
| nuclear transcription factor Y subunit alpha | NFYA | 1.013 | 0.0456 |  |  |  |  |
| nuclear factor kappa B subunit 2 | NFKB2 |  |  | 2.064 | 0.0494 |  |  |
| NOP58 ribonucleoprotein | NOP58 |  |  | 0.725 | 0.0167 |  |  |
| neuronal PAS domain protein 4 | NPAS4 | 0.884 | 0.025 |  |  |  |  |
| nucleophosmin 1 | NPM1 |  |  | -0.639 | 0.00255 | -0.553 | 0.0114 |
| natriuretic peptide receptor 2 | NPR2 | 1.206 | 0.00714 |  |  |  |  |
| neuronal pentraxin receptor | NPTXR | 0.682 | 0.00208 | -0.753 | 0.0493 |  |  |
| nuclear receptor subfamily 2 group C member 1 | NR2C1 |  |  | -1.467 | 0.0155 |  |  |
| nuclear receptor subfamily 4 group A member 3 | NR4A3 | 1.621 | 0.0426 |  |  |  |  |
| neuregulin 1 | NRG1 |  |  |  |  | -0.976 | 0.000649 |
| osteoglycin | OGN | 3.206 | 0.013 |  |  |  |  |
| OPA1 mitochondrial dynamin like GTPase | OPA1 |  |  | -1.216 | 0.0175 |  |  |
| p21 (RAC1) activated kinase 1 | PAK1 | 0.685 | 0.0261 |  |  |  |  |
| -- | PAQR3 | 1.273 | 0.0297 |  |  |  |  |
| PBX homeobox 1 | PBX1 |  |  |  |  | -0.607 | 0.0113 |
| PBX homeobox interacting protein 1 | PBXIP1 |  |  |  |  | 3.664 | 0.0106 |
| Purkinje cell protein 4 like 1 | PCP4L1 |  |  | 0.695 | 0.0536 |  |  |
| platelet derived growth factor D | PDGFD | -0.611 | 0.000933 |  |  |  |  |
| protein disulfide isomerase family A member 4 | PDIA4 | 0.685 | 0.0232 |  |  |  |  |
| phosphatidylethanolamine N-methyltransferase | PEMT | 1.036 | 0.00649 |  |  |  |  |
| proenkephalin | PENK |  |  | -1.521 | 0.037 |  |  |
| pescadillo ribosomal biogenesis factor 1 | PES1 |  |  | -2.258 | 0.0498 |  |  |
| phosphofructokinase, liver type | PFKL |  |  |  |  | -2.951 | 0.0345 |
| phytanoyl-CoA 2-hydroxylase interacting protein like | PHYHIPL | 0.644 | 0.03 |  |  |  |  |
| paired like homeodomain 1 | PITX1 |  |  |  |  | 2.557 | 0.0415 |
| pyruvate kinase M1/2 | PKM |  |  | -0.691 | 0.0239 |  |  |
| PBX/knotted 1 homeobox 2 | PKNOX2 |  |  | 0.78 | 0.0284 |  |  |
| phospholipase A2 receptor 1 | PLA2R1 | -1.057 | 0.0508 |  |  |  |  |
| plexin A4 | PLXNA4 | 0.905 | 0.0019 | 0.616 | 0.0471 |  |  |
| phospholipase C beta 1 | PLCB1 |  |  |  |  | -2.466 | 0.0345 |
| pleckstrin | PLEK |  |  | -2.637 | 0.0374 |  |  |
| peptidase, mitochondrial processing subunit beta | PMPCB |  |  | -0.936 | 0.0386 |  |  |

| **Supplemental Table S5D. Differentially Expressed Genes in Somatotropes That are E2 Targets  VCD vs Vehicle control females** | | | | | | | |
| --- | --- | --- | --- | --- | --- | --- | --- |
|  |  | **Somatotrope 1 (SOM1)** | | **Somatotrope 2 (SOM2)** | | **Somatotrope 3 (SOM3)** | |
|  | **Gene** | **avg_log2FC** | **p_val** | **avg_log2FC** | **p_val** | **p_val** | **avg_log2FC** |
| patatin like phospholipase domain containing 3 | PNPLA3 |  |  | -2.936 | 0.0498 |  |  |
| protein phosphatase, Mg2+/Mn2+ dependent 1D | PPM1D |  |  | -2.637 | 0.0127 |  |  |
| protein phosphatase 2 catalytic subunit alpha | PPP2CA |  |  | 1.142 | 0.03 |  |  |
| protein phosphatase 2 regulatory subunit Bgamma | PPP2R2C |  |  | 0.9 | 0.0311 |  |  |
| peroxiredoxin 4 | PRDX4 |  |  | -1.361 | 0.00183 |  |  |
| prolactin | PRL | -0.837 | 0.0000046 | -0.735 | 0.00758 | -0.619 | 0.000188 |
| protein tyrosine kinase 2 | PTK2 |  |  |  |  | -3.603 | 0.00475 |
| protein tyrosine phosphatase receptor type K | PTPRK | 0.24 | 0.0279 |  |  | -2.844 | 0.0174 |
| protein tyrosine phosphatase receptor type N | PTPRN |  |  |  |  | 1.664 | 0.0227 |
| patched 1 | PTCH1 | -1.042 | 0.0158 |  |  |  |  |
| protein tyrosine phosphatase non-receptor type 1 | PTPN1 | 0.605 | 0.0244 |  |  |  |  |
| protein tyrosine phosphatase receptor type J | PTPRJ | 0.694 | 0.0384 |  |  |  |  |
| purine rich element binding protein B | PURB |  |  | -1.558 | 0.000685 |  |  |
| RAB31, member RAS oncogene family | RAB31 | 1.884 | 0.00149 |  |  |  |  |
| RAB6A, member RAS oncogene family | RAB6A |  |  |  |  | -1.144 | 0.0418 |
| RAD54 like | RAD54L | 1.621 | 0.0182 |  |  |  |  |
| ral guanine nucleotide dissociation stimulator | RALGDS |  |  |  |  | -3.144 | 0.0345 |
| ras related dexamethasone induced 1 | RASD1 | 0.779 | 0.00659 |  |  |  |  |
| RB binding protein 8, endonuclease | RBBP8 | 1.106 | 0.03 |  |  |  |  |
| retinol binding protein 4 | RBP4 |  |  |  |  | -1.018 | 0.0288 |
| REL proto-oncogene, NF-kB subunit | REL | 0.728 | 0.0365 |  |  |  |  |
| regulatory factor X5 | RFX5 | -1.079 | 0.0497 |  |  |  |  |
| ras homolog family member Q | RHOQ |  |  | 1.331 | 0.00263 |  |  |
| RAR related orphan receptor A | RORA |  |  |  |  | -1.175 | 0.00679 |
| RAR related orphan receptor C | RORC | 1.843 | 0.0077 |  |  |  |  |
| reprimo, TP53 dependent G2 arrest mediator homolog | RPRM |  |  | -2.052 | 0.0386 |  |  |
| RUNX family transcription factor 2 | RUNX2 | 1.514 | 0.0291 |  |  |  |  |
| relaxin family peptide receptor 1 | RXFP1 |  |  |  |  | -3.144 | 0.0345 |
| ryanodine receptor 2 | RYR2 | 1.08 | 0.0144 |  |  |  |  |
| semaphorin 3A | SEMA3A | -1.186 | 0.0287 |  |  |  |  |
| septin 4 | SEPTIN4 |  |  | -1.936 | 0.0378 |  |  |
| sideroflexin 5 | SFXN5 | -1.042 | 0.0474 |  |  |  |  |
| N-sulfoglucosamine sulfohydrolase | SGSH |  |  | -2.258 | 0.0498 |  |  |
| salt inducible kinase 1 | SIK1 | 1.008 | 0.0322 |  |  | -2.729 | 0.0345 |
| SKI proto-oncogene | SKI |  |  | 1.265 | 0.00699 |  |  |
| solute carrier family 25 member 12 | SLC25A12 | -0.587 | 0.0458 | -0.662 | 0.0426 |  |  |
| solute carrier family 2 member 13 | SLC2A13 |  |  | 0.702 | 0.00333 |  |  |
| solute carrier family 39 member 6 | SLC39A6 |  |  | 3.523 | 0.00352 |  |  |
| solute carrier family 6 member 17 | SLC6A17 |  |  | 1.007 | 0.0379 |  |  |
| solute carrier family 20 member 1 | SLC20A1 | -1.601 | 0.0521 |  |  |  |  |
| solute carrier family 25 member 12 | SLC25A12 | -0.587 | 0.0458 | -0.662 | 0.0426 |  |  |
| solute carrier family 2 member 1 | SLC2A1 | 0.656 | 0.0213 |  |  |  |  |
| solute carrier family 6 member 8 | SLC6A8 | 1.736 | 0.0286 |  |  |  |  |
| solute carrier organic anion transporter family member 2A1 | SLCO2A1 | 2.405 | 0.0000191 |  |  |  |  |
| SMAD family member 2 | SMAD2 |  |  | -1.396 | 0.0403 |  |  |
| sphingomyelin phosphodiesterase 1 | SMPD1 |  |  | -1.702 | 0.0199 |  |  |
| SMAD specific E3 ubiquitin protein ligase 2 | SMURF2 | 0.77 | 0.0389 |  |  |  |  |
| snail family transcriptional repressor 2 | SNAI2 |  |  | -2.936 | 0.0159 |  |  |
| synuclein alpha | SNCA | -3.286 | 0.0103 |  |  |  |  |
| suppressor of cytokine signaling 1 | SOCS1 | 1.097 | 0.0326 |  |  |  |  |
| SON DNA and RNA binding protein | SON |  |  | 0.649 | 0.0181 |  |  |
| sortilin related VPS10 domain containing receptor 1 | SORCS1 | 0.905 | 0.0105 |  |  |  |  |
| sortilin related receptor 1 | SORL1 |  |  |  |  | -1.629 | 0.0212 |
| SOS Ras/Rac guanine nucleotide exchange factor 1 | SOS1 |  |  |  |  | -3.314 | 0.0177 |
| SRY-box transcription factor 5 | SOX5 |  |  | -0.718 | 0.00175 |  |  |
| SRY-box transcription factor 11 | SOX11 | 2.399 | 0.00381 |  |  |  |  |
| sperm associated antigen 9 | SPAG9 |  |  | 0.74 | 0.00136 |  |  |
| SPARC (osteonectin), cwcv and kazal like domains proteoglycan 2 | SPOCK2 |  |  | 0.929 | 0.00655 |  |  |

| Supplemental Table S5E Differentially Expressed Genes in Somatotropes That are E2 Targets VCD vs Vehicle control females | | | | | | | |
| --- | --- | --- | --- | --- | --- | --- | --- |
|  |  | **Somatotrope 1 (SOM1)** | | **Somatotrope 2 (SOM2)** | | **Somatotrope 3 (SOM3)** | |
|  |  | **avg_log2FC** | **p_val** | **avg_log2FC** | **avg_log2FC** | **p_val** | **avg_log2FC** |
| sprouty RTK signaling antagonist 4 | SPRY4 | 2.621 | 0.0316 |  |  |  |  |
| sterol regulatory element binding transcription factor 2 | SREBF2 |  |  | -0.661 | 0.0505 |  |  |
| serine and arginine repetitive matrix 1 | SRRM1 |  |  | 0.871 | 0.014 |  |  |
| serine and arginine rich splicing factor 7 | SRSF7 | 0.711 | 0.012 |  |  |  |  |
| signal sequence receptor subunit 2 | SSR2 |  |  | 0.894 | 0.0448 |  |  |
| ST6 N-acetylgalactosaminide alpha-2,6-sialyltransferase 4 | SSTR3 | 1.469 | 0.0515 |  |  |  |  |
| signal transducer and activator of transcription 1 | ST6GALNAC4 | -1.838 | 0.0383 |  |  |  |  |
| signal transducer and activator of transcription 3 | STAT1 | 0.897 | 0.0158 |  |  |  |  |
| stonin 1 | STAT3 | 0.698 | 0.0000177 |  |  | -3.951 | 0.00475 |
| syntaxin binding protein 1 | STON1 |  |  | 2.971 | 0.00144 |  |  |
| spectrin repeat containing nuclear envelope protein 1 | STXBP1 |  |  |  |  | 2.856 | 0.0318 |
| spectrin repeat containing nuclear envelope family member 3 | SYNE1 |  |  | -0.738 | 0.0255 |  |  |
| synaptopodin | SYNE3 | 1.02 | 0.0346 |  |  |  |  |
| synaptophysin | SYNPO | -1.379 | 0.0317 |  |  |  |  |
| synaptotagmin like 4 | SYP |  |  |  |  | 2.856 | 0.0318 |
| thyroid hormone receptor alpha | SYTL4 |  |  |  |  | 2.316 | 0.0251 |
| thyroid hormone receptor beta | THRA |  |  | -1.351 | 0.00502 |  |  |
| TIMP metallopeptidase inhibitor 2 | THRB |  |  |  |  | -2.729 | 0.038 |
| TIMP metallopeptidase inhibitor 3 | TIMP2 | 0.749 | 0.0275 |  |  |  |  |
| tight junction protein 3 | TIMP3 |  |  | -1.659 | 0.00523 |  |  |
| TLE family member 5, transcriptional modulator | TJP3 | 3.428 | 0.00544 |  |  |  |  |
| TATA element modulatory factor 1 | TLE5 |  |  | -0.964 | 0.0273 |  |  |
| thymosin beta 4 X-linked | TMF1 |  |  | -1.843 | 0.0222 |  |  |
| transmembrane 4 L six family member 1 | TMSB4X | 0.901 | 0.00262 | 0.781 | 0.0365 |  |  |
| transmembrane and coiled-coil domain family 3 | TM4SF1 |  |  |  |  | 2.763 | 0.0241 |
| transmembrane p24 trafficking protein 9 | TMCC3 |  |  |  |  | -2.951 | 0.0345 |
| transmembrane protein 258 | TMED9 |  |  |  |  | 2.664 | 0.00192 |
| thymosin beta 4 X-linked | TMEM258 |  |  |  |  | 1.289 | 0.016 |
| DNA topoisomerase III beta | TNFAIP3 | 2.313 | 0.00151 |  |  |  |  |
| tumor protein D52 | TOP3B |  |  | 1.504 | 0.0265 |  |  |
| TPD52 like 1 | TPD52 |  |  | 0.874 | 0.00416 |  |  |
| tripartite motif containing 25 | TPD52L1 |  |  |  |  | 2.664 | 0.0318 |
| transient receptor potential cation channel subfamily C member 6 | TRIM25 | 1.943 | 0.000587 |  |  | 1.441 | 0.18 |
| thioredoxin interacting protein | TRPC6 |  |  | 2.234 | 0.0492 |  |  |
| vascular endothelial growth factor A | TXNIP |  |  |  |  | -1.559 | 0.0421 |
| vinculin | VEGFA | 3.943 | 0.000988 |  |  |  |  |
| whirlin | VCL | 0.763 | 0.00118 |  |  | -3.466 | 0.0345 |
| yippee like 3 | WHRN | 0.66 | 0.00524 |  |  |  |  |
| zinc finger and BTB domain containing 16 | YPEL3 |  |  | -1.384 | 0.00129 |  |  |
| zyxin | ZBTB16 | 0.596 | 0.00507 | 0.918 | 0.00908 |  |  |
| ST6 N-acetylgalactosaminide alpha-2,6-sialyltransferase 4 | ZYX |  |  | 1.649 | 0.016 |  |  |

| **Supplemental Table S6A DEGS LAC1 Cluster; VCD/Vehicle.** Maroon=upregulated; | | | | | | | | |
| --- | --- | --- | --- | --- | --- | --- | --- | --- |
| **gene** | **avg_log2FC** | **p_val** | **gene** | **avg_log2FC** | **p_val** | **gene** | **avg_log2FC** | **p_val** |
| Dusp5 | 4.353446037 | 0.000720065 | Col23a1 | 2.298998253 | 0.001796927 | Kcnk10 | 1.959167098 | 2.10E-07 |
| Hrh4 | 4.090411631 | 0.000720049 | Gm28370 | 2.298998253 | 0.01085314 | Slu7 | 1.949055782 | 0.007979712 |
| Tincr | 3.768483536 | 0.002509368 | Ints12 | 2.298998253 | 0.019390942 | Cycs | 1.938408538 | 0.000662949 |
| Rps6ka1 | 3.642952654 | 0.002509337 | Kcnq3 | 2.283056709 | 1.81E-05 | Lrrc42 | 1.92048663 | 0.020957302 |
| Traf2 | 3.505449131 | 0.001341696 | Rpe | 2.283056709 | 4.40E-05 | Rbp1 | 1.92048663 | 0.020957302 |
| Gm49668 | 3.505449131 | 0.004716442 | Zfp651 | 2.270983877 | 0.001768006 | Magohb | 1.92048663 | 0.034794795 |
| Serpine2 | 3.505449131 | 0.008922555 | Sft2d1 | 2.270983877 | 0.003106449 | Gm15687 | 1.92048663 | 0.034794795 |
| Mccc1os | 3.353446037 | 0.00250941 | Phgdh | 2.270983877 | 0.009544558 | Amigo3 | 1.92048663 | 0.034794795 |
| Myh7b | 3.353446037 | 0.004716383 | Ezh2 | 2.253910364 | 0.004618277 | Ccdc137 | 1.92048663 | 0.034794795 |
| 5730409E04Rik | 3.353446037 | 0.004716383 | Gm45307 | 2.183521036 | 0.005457775 | Gpt | 1.92048663 | 0.034794795 |
| Tigit | 3.353446037 | 0.008922555 | A530017D24Rik | 2.183521036 | 0.005457775 | Psmb8 | 1.89972807 | 0.003861192 |
| Myo19 | 3.183521036 | 0.00175699 | Cited1 | 2.183521036 | 0.009544558 | Gm14216 | 1.89972807 | 0.013660738 |
| Il20rb | 3.183521036 | 0.004716521 | Rabggta | 2.183521036 | 0.010882371 | Itga11 | 1.883960754 | 0.009616512 |
| 4930447A16Rik | 3.183521036 | 0.004716521 | Gm4890 | 2.183521036 | 0.019442004 | Cdc34 | 1.883960754 | 0.016659485 |
| Wwtr1 | 3.183521036 | 0.008922479 | Gtf3a | 2.183521036 | 0.019493116 | Nog | 1.883960754 | 0.016659485 |
| Tcf7 | 3.183521036 | 0.008922479 | Tnxb | 2.183521036 | 0.020797413 | Zfp335 | 1.883960754 | 0.028681903 |
| Gm15342 | 3.183521036 | 0.008922479 | Gm34552 | 2.183521036 | 0.026921162 | Pecam1 | 1.883960754 | 0.034929638 |
| Pex10 | 3.090411631 | 0.003253906 | Gm7694 | 2.183521036 | 0.038725209 | Hsf4 | 1.861592941 | 0.016700635 |
| Tcirg1 | 3.041502031 | 0.000311009 | Gm37876 | 2.183521036 | 0.038725209 | Gm56721 | 1.861592941 | 0.037920235 |
| Bfsp1 | 2.990875958 | 0.003248785 | Marcksl1 | 2.183521036 | 0.038725209 | Txnrd2 | 1.861592941 | 0.037920235 |
| Slc35f6 | 2.990875958 | 0.008922631 | Bloc1s4 | 2.183521036 | 0.038725209 | Abcc10 | 1.820950956 | 0.004015834 |
| Brme1 | 2.990875958 | 0.008922631 | Haus5 | 2.183521036 | 0.038725209 | Klhl1 | 1.811552258 | 0.004012274 |
| Gm48606 | 2.990875958 | 0.008922631 | Gm56829 | 2.183521036 | 0.038725209 | Disp3 | 1.808011901 | 0.000127672 |
| Gm28875 | 2.990875958 | 0.008922631 | Gm48830 | 2.183521036 | 0.038725209 | Gcnt2 | 1.808011901 | 0.000360584 |
| Pcdhb12 | 2.990875958 | 0.008922631 | Vdr | 2.183521036 | 0.038725209 | Mdp1 | 1.768483536 | 0.000577846 |
| Msantd3 | 2.883960754 | 0.006008538 | Htr7 | 2.183521036 | 0.038725209 | Dhx37 | 1.768483536 | 0.003187535 |
| Gm46329 | 2.846486048 | 0.001024938 | Hic2 | 2.109520454 | 0.013446466 | E2f4 | 1.768483536 | 0.010502123 |
| Pex11b | 2.768483536 | 0.003259018 | Zswim4 | 2.101058876 | 4.96E-07 | Cngb3 | 1.768483536 | 0.016841008 |
| B3galnt1 | 2.768483536 | 0.00601794 | Zfp276 | 2.090411631 | 0.006118354 | Pwwp3a | 1.768483536 | 0.017983569 |
| Gm50012 | 2.686021376 | 0.00103084 | Gm17056 | 2.068043818 | 1.62E-05 | Etnk2 | 1.768483536 | 0.02288085 |
| Efhd2 | 2.642952654 | 0.01115477 | Gtf3c5 | 2.057990154 | 0.002261048 | Tex49 | 1.768483536 | 0.02288085 |
| Bhlha15 | 2.642952654 | 0.0207333 | Gm26952 | 2.057990154 | 0.010912893 | Kctd5 | 1.768483536 | 0.02288085 |
| Zscan22 | 2.505449131 | 0.00175426 | Tufm | 2.057990154 | 0.011294826 | Tgm4 | 1.768483536 | 0.028780941 |
| Msh6 | 2.505449131 | 0.001866824 | Ldhb | 2.057990154 | 0.019493116 | Yap1 | 1.768483536 | 0.034885444 |
| Nutf2 | 2.505449131 | 0.003356745 | Kics2 | 2.057990154 | 0.019493116 | Llgl2 | 1.768483536 | 0.034885444 |
| Nxn | 2.505449131 | 0.00337605 | Marveld2 | 2.057990154 | 0.019493116 | Map3k11 | 1.768483536 | 0.034885444 |
| Mcat | 2.505449131 | 0.006037258 | Dynlt1a | 2.057990154 | 0.019493116 | Ndrg4 | 1.768483536 | 0.038233843 |
| Gm40893 | 2.505449131 | 0.006042283 | Cacna1h | 2.057990154 | 0.019493116 | Crybb3 | 1.768483536 | 0.049432909 |
| Arntl2 | 2.505449131 | 0.011172008 | Stk11ip | 2.057990154 | 0.034706237 | Rnf40 | 1.731008831 | 0.000436545 |
| Tceanc | 2.505449131 | 0.011172008 | Suv39h1 | 2.031517942 | 0.013446481 | Gm11973 | 1.719573936 | 0.000201317 |
| Gm34590 | 2.505449131 | 0.020764926 | Tnfaip3 | 2.031517942 | 0.016621045 | Akt1 | 1.714035752 | 0.000684462 |
| Chrm3 | 2.505449131 | 0.020829263 | Pym1 | 2.031517942 | 0.022588609 | Klf2 | 1.707082992 | 0.018039375 |
| Gm15972 | 2.505449131 | 0.038609314 | Aup1 | 2.020022303 | 0.000788348 | Trim25 | 1.698094209 | 0.000945545 |
| Gm31333 | 2.505449131 | 0.038667271 | Gsg1l | 2.013596034 | 0.000657159 | Gm4651 | 1.686021376 | 0.010882036 |
| Adnp2 | 2.505449131 | 0.001533552 | L3mbtl2 | 1.990875958 | 0.001589265 | Ipmk | 1.686021376 | 0.029259774 |
| Nek3 | 2.431448549 | 0.009509592 | Gm17089 | 1.990875958 | 0.003830634 | Llgl1 | 1.686021376 | 0.029259774 |
| Zfp977 | 2.405913457 | 0.006042283 | Arf2 | 1.990875958 | 0.009579594 | Ccdc32 | 1.668947863 | 0.009722375 |
| AV356131 | 2.405913457 | 0.006042283 | 1700054A03Rik | 1.990875958 | 0.028681903 | Cad | 1.668947863 | 0.009722375 |
| D2hgdh | 2.353446037 | 0.000998945 | Nexn | 1.990875958 | 0.038784149 | Ptp4a3 | 1.668947863 | 0.01682025 |
| Mx1 | 2.353446037 | 0.009509592 | Angptl7 | 1.990875958 | 0.038784149 | Gpr171 | 1.668947863 | 0.022978839 |
| 9330160F10Rik | 2.353446037 | 0.011189944 | Gm43111 | 1.990875958 | 0.038784149 | Chrna6 | 1.668947863 | 0.022978839 |
| Dph1 | 2.353446037 | 0.011189944 | Foxp2 | 1.990875958 | 0.038784149 | Tmem185a | 1.668947863 | 0.028981988 |
| Tedc2 | 2.353446037 | 0.011189944 | Il12rb2 | 1.990875958 | 0.038784149 | Nup37 | 1.668947863 | 0.038233843 |
| Gm57198 | 2.353446037 | 0.020764926 | Slc2a3 | 1.990875958 | 0.038784149 | Gm26533 | 1.668947863 | 0.038233843 |
| Gm16316 | 2.353446037 | 0.038667271 | Gm47601 | 1.990875958 | 0.038784149 | Vav3 | 1.65995908 | 4.67E-06 |
| Pck2 | 2.353446037 | 0.038725209 | Gm16046 | 1.990875958 | 0.038784149 | Bcl9l | 1.653006319 | 0.001226082 |
| Fzd8 | 2.353446037 | 0.038725209 | Fosl1 | 1.990875958 | 0.038784149 | Ankmy2 | 1.650647046 | 0.000255934 |

| **Supplemental Table S6B DEGS LAC1 Cluster; VCD/Vehicle.** Maroon=upregulated; | | | | | | | | |
| --- | --- | --- | --- | --- | --- | --- | --- | --- |
| **gene** | **avg_log2FC** | **p_val** | **gene** | **avg_log2FC** | **p_val** | **gene** | **avg_log2FC** | **p_val** |
| *Polr2f* | 1.642952654 | 0.000821768 | *Gm43192* | 1.446555442 | 0.011046001 | *4930565N06Rik* | 1.29043624 | 0.044198161 |
| *Mettl22* | 1.642952654 | 0.011060716 | *Gm17103* | 1.446555442 | 0.016940385 | *Ccp110* | 1.283056709 | 0.023277435 |
| *Tiam2* | 1.642952654 | 0.01972643 | *Pi4k2b* | 1.446555442 | 0.023608893 | *Zfp81* | 1.283056709 | 0.029925908 |
| *Proca1* | 1.642952654 | 0.028883181 | *C1qc* | 1.446555442 | 0.029115577 | *Rptoros* | 1.283056709 | 0.038952123 |
| *Zfp971* | 1.642952654 | 0.04932176 | *Gm44956* | 1.446555442 | 0.029115577 | *Bcl10* | 1.283056709 | 0.048232827 |
| *Gm20616* | 1.642952654 | 0.04932176 | *Cbx8* | 1.446555442 | 0.038554072 | *Gm39103* | 1.283056709 | 0.048412918 |
| *Cyren* | 1.624093627 | 0.013902069 | *Gsto2* | 1.446555442 | 0.049652066 | *Ttc41* | 1.27663044 | 0.02651319 |
| *Mylip* | 1.624093627 | 0.018001938 | *Gtpbp10* | 1.446555442 | 0.04976158 | *Emd* | 1.270983877 | 0.00708053 |
| *Ppm1n* | 1.624093627 | 0.023178259 | *ENSMUSG00000121495* | 1.439860789 | 0.000573766 | *Gm3336* | 1.270983877 | 0.029318748 |
| *Zfp119a* | 1.598558535 | 0.01380964 | *Gm49959* | 1.439860789 | 0.000798423 | *Ubald1* | 1.261523548 | 0.04865139 |
| *Cavin3* | 1.598558535 | 0.016960939 | *Nfe2l3* | 1.437277628 | 0.00682788 | *Apbb3* | 1.261523548 | 0.048957928 |
| *Zfp367* | 1.598558535 | 0.018018186 | *Gm28294* | 1.431448549 | 0.008714943 | *Zfp800* | 1.253910364 | 0.004318586 |
| *Spring1* | 1.598558535 | 0.029402921 | *Tacc2* | 1.420560233 | 0.001011217 | *Arl14ep* | 1.253910364 | 0.006933251 |
| *1700010K24Rik* | 1.598558535 | 0.039076351 | *Ube3b* | 1.420560233 | 0.032681432 | *Ccdc84* | 1.253910364 | 0.04309131 |
| *Tppp3* | 1.582070412 | 0.003945699 | *Rabepk* | 1.405913457 | 0.001958969 | *Cul7* | 1.253910364 | 0.054276587 |
| *Mpnd* | 1.575838458 | 0.041663947 | *Mphosph6* | 1.405913457 | 0.006668057 | *Tmod1* | 1.247651373 | 0.015679806 |
| *Klhl11* | 1.575838458 | 0.041909823 | *Crat* | 1.405913457 | 0.020440348 | *Mfap3* | 1.247651373 | 0.032309958 |
| *Lrrc40* | 1.570544159 | 0.0007591 | *Chrnb3* | 1.405913457 | 0.023277435 | *Rab31* | 1.24492158 | 0.014069774 |
| *Fam210b* | 1.562032659 | 0.00331683 | *Birc3* | 1.405913457 | 0.031518148 | *Anxa2* | 1.242414725 | 0.006540073 |
| *Agr2* | 1.562032659 | 0.016880255 | *Haus6* | 1.405913457 | 0.038671063 | *Shank3* | 1.242414725 | 0.032153313 |
| *Tyw5* | 1.562032659 | 0.023081024 | *Lrfn1* | 1.405913457 | 0.039134809 | *Htra2* | 1.23796882 | 0.004109442 |
| *Elmo3* | 1.562032659 | 0.023081024 | *Hal* | 1.405913457 | 0.039134809 | *Rab25* | 1.23796882 | 0.022545748 |
| *Gm26879* | 1.562032659 | 0.038391309 | *Gm47320* | 1.405913457 | 0.047915418 | *Bmper* | 1.236632372 | 2.15E-07 |
| *Dhtkd1* | 1.562032659 | 0.038391309 | *Tcerg1l* | 1.395025141 | 0.006609875 | *Tchp* | 1.234147109 | 0.00587887 |
| *Surf6* | 1.562032659 | 0.038391309 | *Phyhd1* | 1.392974401 | 0.004298982 | *Ppan* | 1.234147109 | 0.022714313 |
| *Mir9-3hg* | 1.562032659 | 0.038391309 | *Hhat* | 1.389971913 | 0.000611604 | *Bcl7b* | 1.2230494 | 0.00219196 |
| *Scube2* | 1.562032659 | 0.038391309 | *Foxj2* | 1.385154897 | 0.023608896 | *Hars* | 1.215942513 | 0.000919943 |
| *Sqle* | 1.562032659 | 0.038391309 | *Syngap1* | 1.381460413 | 0.00023522 | *Wbp11* | 1.214547931 | 0.004798708 |
| *Pisd* | 1.546091115 | 0.00044701 | *Ncln* | 1.376166114 | 0.003715845 | *Anxa4* | 1.214547931 | 0.013894089 |
| *Cnnm4* | 1.546091115 | 0.010998114 | *Ndn* | 1.376166114 | 0.036152679 | *Slc7a1* | 1.183521036 | 0.000199902 |
| *Tmem138* | 1.546091115 | 0.022479677 | *Zbtb10* | 1.353446037 | 0.003139649 | *Heatr1* | 1.183521036 | 0.000641404 |
| *Gm1673* | 1.53715799 | 0.008217947 | *Creld1* | 1.353446037 | 0.004899578 | *Pprc1* | 1.183521036 | 0.002594393 |
| *Zfp518a* | 1.531444339 | 0.006258929 | *Terf2ip* | 1.353446037 | 0.014203976 | *Tmem41b* | 1.183521036 | 0.003359682 |
| *Nop14* | 1.505449131 | 0.002327704 | *Mdc1* | 1.353446037 | 0.014203976 | *Enpp5* | 1.183521036 | 0.004409726 |
| *Pnkp* | 1.505449131 | 0.004014214 | *Dnajc30* | 1.353446037 | 0.035242697 | *Arel1* | 1.183521036 | 0.005173845 |
| *Myo5b* | 1.505449131 | 0.005576159 | *Zfp27* | 1.353446037 | 0.036220977 | *Ptdss2* | 1.183521036 | 0.006525664 |
| *Mfap1b* | 1.505449131 | 0.008662797 | *Gm5577* | 1.353446037 | 0.042404996 | *Recql* | 1.183521036 | 0.010261051 |
| *Rassf3* | 1.505449131 | 0.016840346 | *Ube2g2* | 1.343985708 | 0.004207374 | *Gm9856* | 1.183521036 | 0.013436751 |
| *Atxn7l1os2* | 1.505449131 | 0.019778195 | *Zdhhc8* | 1.335524129 | 0.001411396 | *Gm34102* | 1.183521036 | 0.015130233 |
| *Polr1b* | 1.505449131 | 0.023252475 | *Ly6a* | 1.335524129 | 0.042443976 | *Mgat1* | 1.183521036 | 0.01536628 |
| *Nlgn2* | 1.505449131 | 0.029546573 | *ENSMUSG00000121479* | 1.335524129 | 0.04834633 | *Arv1* | 1.183521036 | 0.015827158 |
| *Cux2* | 1.505449131 | 0.035153165 | *Zmynd12* | 1.332384422 | 5.57E-05 | *Atf6b* | 1.183521036 | 0.01815555 |
| *Rbm19* | 1.505449131 | 0.049488842 | *Uckl1* | 1.321024559 | 0.005320508 | *D830036C21Rik* | 1.183521036 | 0.022913767 |
| *Mtrfr* | 1.505449131 | 0.049488842 | *Zfp316* | 1.321024559 | 0.011010615 | *4933439C10Rik* | 1.183521036 | 0.02357931 |
| *Hps3* | 1.505449131 | 0.012970061 | *Zbtb16* | 1.321024559 | 0.014359288 | *Kif19a* | 1.183521036 | 0.03217512 |
| *Klf4* | 1.501882516 | 5.62E-09 | *Nup133* | 1.321024559 | 0.020741811 | *Zkscan14* | 1.183521036 | 0.035198127 |
| *Tnni3k* | 1.483081318 | 0.00482399 | *Tctn3* | 1.321024559 | 0.023781188 | *Gm26827* | 1.183521036 | 0.038512795 |
| *Nxpe3* | 1.478976919 | 0.008281193 | *Get1* | 1.321024559 | 0.029215904 | *Myadm* | 1.183521036 | 0.040698041 |
| *Kndc1* | 1.478976919 | 0.009841689 | *Slc3a1* | 1.321024559 | 0.0498167 | *Cmbl* | 1.183521036 | 0.048458263 |
| *Pcna* | 1.473027653 | 0.008278151 | *Ttc39c* | 1.318170562 | 3.55E-05 | *Mllt1* | 1.183521036 | 0.048458263 |
| *Lsmem1* | 1.468923255 | 0.000621506 | *Psmg2* | 1.312804053 | 0.040723634 | *Gm12781* | 1.183521036 | 0.054604307 |
| *Edrf1* | 1.463628955 | 0.000122861 | *Vps16* | 1.30551156 | 0.001650581 | *Ost4* | 1.15550666 | 0.000418348 |
| *Nr1h2* | 1.463628955 | 0.001216982 | *Fndc4* | 1.30551156 | 0.013823438 | *Sgms2* | 1.15550666 | 0.039934723 |
| *Mzf1* | 1.463628955 | 0.011055711 | *Acot7* | 1.298998253 | 0.000487643 | *Homer1* | 1.1513774 | 0.000211825 |
| *Rbm34* | 1.463628955 | 0.022606308 | *Atmin* | 1.298998253 | 0.023376899 | *Edem1* | 1.150354172 | 0.00097962 |
| *Ntrk3* | 1.454374946 | 2.89E-11 | *Abhd11* | 1.298998253 | 0.038830172 | *Nt5dc3* | 1.14699516 | 0.003368526 |
| *Plcl1* | 1.446555442 | 0.00536912 | *A330093E20Rik* | 1.298998253 | 0.038830172 | *Tubb4b* | 1.14604633 | 0.002111576 |

| **Supplemental Table S6C DEGS LAC1 Cluster; VCD/Vehicle.** Maroon=upregulated; | | | | | | | | |
| --- | --- | --- | --- | --- | --- | --- | --- | --- |
| **gene** | **avg_log2FC** | **p_val** | **gene** | **avg_log2FC** | **p_val** | **gene** | **avg_log2FC** | **p_val** |
| *Fhl1* | 1.142879051 | 0.013104205 | *Hadhb* | 1.031517942 | 0.003933436 | *Efl1* | 0.950860279 | 0.000395312 |
| *Pdcd11* | 1.140452314 | 0.001905054 | *Ppm1d* | 1.031517942 | 0.005221157 | *Dot1l* | 0.949055782 | 0.000172703 |
| *Tmem164* | 1.140452314 | 0.018973785 | *Wars2* | 1.031517942 | 0.006262093 | *Vegfa* | 0.949055782 | 0.006843984 |
| *Gm3235* | 1.137717346 | 0.017463627 | *Gmeb1* | 1.031517942 | 0.010816448 | *Dnai4* | 0.945361299 | 0.038333792 |
| *Pptc7* | 1.131053616 | 0.003558586 | *Msto1* | 1.031517942 | 0.017895325 | *Cry1* | 0.944333372 | 0.009654335 |
| *Pja1* | 1.129073252 | 0.002093162 | *Trub2* | 1.031517942 | 0.025521933 | *Kctd4* | 0.942512936 | 0.001643097 |
| *Sh3gl1* | 1.126937507 | 0.003041045 | *Ptpn12* | 1.031517942 | 0.031079486 | *Pggt1b* | 0.942512936 | 0.016843247 |
| *Zdhhc7* | 1.126937507 | 0.010369703 | *Entpd6* | 1.031517942 | 0.0315132 | *Ccn1* | 0.938408538 | 0.000845336 |
| *Dennd2b* | 1.126937507 | 0.020647216 | *Gm45894* | 1.031517942 | 0.035242697 | *Sipa1l3* | 0.938408538 | 0.02083392 |
| *Zfp110* | 1.124627347 | 0.001434115 | *Spindoc* | 1.025979759 | 0.002226978 | *Creb3l1* | 0.938234478 | 4.65E-08 |
| *Nfya* | 1.122120491 | 0.015946619 | *Slc25a28* | 1.02482329 | 0.01726603 | *Map6* | 0.935593522 | 0.028380059 |
| *Gm43066* | 1.122120491 | 0.021121376 | *Fam118a* | 1.021769966 | 0.037677952 | *Lpin2* | 0.933542783 | 0.001256699 |
| *Ilkap* | 1.119390698 | 0.004462541 | *Tjap1* | 1.020022303 | 0.008028817 | *Usp6nl* | 0.929764443 | 0.002458915 |
| *Med8* | 1.11640684 | 0.017897329 | *Garem1* | 1.020022303 | 0.03274879 | *Mmd2* | 0.929764443 | 0.00331302 |
| *Ppp1r15b* | 1.11640684 | 0.032416554 | *Kif3b* | 1.017511084 | 0.021481685 | *Micos13* | 0.929764443 | 0.007195448 |
| *Cox18* | 1.11640684 | 0.04950673 | *Kctd20* | 1.013596034 | 0.032330577 | *Otud7a* | 0.927181282 | 0.00017007 |
| *Mapkapk5* | 1.113131708 | 0.001734585 | *Mrtfa* | 1.012040568 | 0.000677372 | *Tmem259* | 0.92048663 | 0.002413202 |
| *Recql5* | 1.113131708 | 0.005547029 | *Nadk* | 1.009491636 | 0.036330607 | *Shb* | 0.92048663 | 0.007980481 |
| *Pim2* | 1.109520454 | 0.034777802 | *Nup88* | 1.009491636 | 0.036846559 | *Ypel4* | 0.92048663 | 0.012244935 |
| *Slc18a1* | 1.109520454 | 0.043236321 | *Baiap2* | 1.007949471 | 0.006355766 | *Hmces* | 0.92048663 | 0.013565791 |
| *Parp1* | 1.109520454 | 0.055005726 | *Tcf3* | 1.000656979 | 0.010114475 | *Slc35a5* | 0.92048663 | 0.025416166 |
| *Zbtb8os* | 1.109520454 | 0.055005726 | *Trim46* | 0.999096465 | 0.003618872 | *Bcl2l2* | 0.92048663 | 0.03283218 |
| *Ncdn* | 1.101058876 | 0.023254635 | *Tdrd7* | 0.999096465 | 0.003618872 | *Optn* | 0.92048663 | 0.040849625 |
| *Ints11* | 1.101058876 | 0.043196739 | *Tbce* | 0.99750905 | 0.000137845 | *Kif22* | 0.92048663 | 0.050482144 |
| *Srek1ip1* | 1.101058876 | 0.043196739 | *Gm26802* | 0.992424745 | 0.010888631 | *Cdr2* | 0.92048663 | 0.005179171 |
| *Nol10* | 1.098632138 | 0.001419001 | *Tmx2* | 0.990875958 | 0.024705863 | *Ccdc157* | 0.92048663 | 0.02219391 |
| *Zfp598* | 1.097791162 | 0.002187748 | *Snhg3* | 0.990875958 | 0.013971639 | *Dag1* | 0.92048663 | 0.054214424 |
| *Asap2* | 1.097791162 | 0.021559974 | *Slc1a5* | 0.990875958 | 0.017173033 | *Atf3* | 0.917185267 | 2.31E-05 |
| *Agrp* | 1.096058194 | 0.008864101 | *Fam183b* | 0.990875958 | 0.017680003 | *Ppp2r2c* | 0.913913976 | 0.001337774 |
| *Ppp6r1* | 1.096058194 | 0.020625071 | *Clcn7* | 0.990875958 | 0.019498678 | *Arhgef10* | 0.909346072 | 0.021182419 |
| *Rdh10* | 1.090411631 | 0.018633416 | *Smad2* | 0.990875958 | 0.03848175 | *Rpl35a* | 0.90598706 | 5.46E-08 |
| *Tor2a* | 1.090411631 | 0.018633416 | *Septin9* | 0.987796565 | 0.000249677 | *Atp6v0a2* | 0.90598706 | 0.000663708 |
| *Timm8b* | 1.090411631 | 0.029955196 | *Mpped2* | 0.983222385 | 0.016631802 | *Cmpk1* | 0.90598706 | 0.008552945 |
| *Pvr* | 1.083985362 | 0.013927619 | *Aasdh* | 0.983222385 | 0.025662931 | *C1d* | 0.90598706 | 0.030963217 |
| *Rhob* | 1.083985362 | 0.045402159 | *Lsr* | 0.979987642 | 0.006904816 | *Ctps* | 0.901750067 | 0.018594103 |
| *Srrm4* | 1.083409551 | 2.39E-05 | *Mpp7* | 0.978442007 | 1.42E-08 | *Ap2a2* | 0.900790829 | 0.000852301 |
| *Gm26532* | 1.081641422 | 0.003447079 | *Agbl5* | 0.977070158 | 0.003575789 | *Ankrd35* | 0.89972807 | 0.041645654 |
| *Vps39* | 1.076605832 | 0.033849867 | *1700028E10Rik* | 0.977070158 | 0.013990738 | *Gm20470* | 0.894014419 | 0.018465062 |
| *Tmem201* | 1.076605832 | 0.036737019 | *Uros* | 0.977070158 | 0.040127121 | *Gpr137* | 0.894014419 | 0.043563033 |
| *Ddx18* | 1.076605832 | 0.045494572 | *Tmie* | 0.977070158 | 0.05333678 | *Rrp7a* | 0.894014419 | 0.050210068 |
| *Zmiz2* | 1.071046306 | 0.008158331 | *Atxn7l1os1* | 0.977070158 | 0.05333678 | *Tcaim* | 0.894014419 | 0.052688602 |
| *4930455C13Rik* | 1.069350016 | 0.00491895 | *Desi1* | 0.975716151 | 0.010721232 | *Grid2* | 0.891340284 | 0.007053793 |
| *Msi1* | 1.068043818 | 0.033892232 | *Nmt1* | 0.974934414 | 0.006069801 | *Ube2j1* | 0.891340284 | 0.019675883 |
| *Fbxo34* | 1.068043818 | 0.041492307 | *Capn15* | 0.973597966 | 0.005023468 | *Mfge8* | 0.889789833 | 0.048469878 |
| *Mast3* | 1.057990154 | 1.72E-05 | *Atxn3* | 0.972016931 | 0.019636718 | *Uchl3* | 0.888065152 | 0.046344975 |
| *Mtmr7* | 1.057990154 | 0.003118971 | *Chl1* | 0.972016931 | 0.028249154 | *Uqcrc2* | 0.887128033 | 0.015046549 |
| *Atrip* | 1.057990154 | 0.013704254 | *Mid1ip1* | 0.967792345 | 0.030299462 | *Zfp451* | 0.883960754 | 0.002738132 |
| *Plekhb1* | 1.057990154 | 0.020993805 | *Frrs1* | 0.967792345 | 0.037881903 | *Ctbp1* | 0.883960754 | 0.006688986 |
| *Gm14703* | 1.057990154 | 0.024527682 | *Eif3b* | 0.967792345 | 0.047702093 | *Rusc1* | 0.883960754 | 0.01594674 |
| *Rfwd3* | 1.057990154 | 0.037045263 | *Hmcn2* | 0.961128614 | 0.011380217 | *Lgr4* | 0.883960754 | 0.019187205 |
| *Eif2b4* | 1.050254505 | 0.011891148 | *Lrp8os2* | 0.961128614 | 0.027811809 | *Eef1akmt3* | 0.883960754 | 0.023679975 |
| *Dimt1* | 1.046017512 | 0.026728393 | *Nt5c3b* | 0.961128614 | 0.034422503 | *Prcc* | 0.883960754 | 0.034915518 |
| *Dync1h1* | 1.04091664 | 2.33E-06 | *Slc25a33* | 0.961128614 | 0.034607809 | *Tuba4a* | 0.883960754 | 0.037899684 |
| *Lmna* | 1.039474666 | 8.26E-08 | *Rtn2* | 0.959480761 | 0.016894134 | *Basp1* | 0.883960754 | 0.039313585 |
| *Azin1* | 1.039131126 | 0.001345227 | *Nptxr* | 0.95611054 | 0.000427166 | *Arhgap26* | 0.883960754 | 0.054102832 |
| *Aff2* | 1.036679647 | 7.48E-06 | *Opn3* | 0.95403919 | 0.013949609 | *Gm41604* | 0.878666454 | 0.00944286 |
| *H2-D1* | 1.031517942 | 1.52E-06 | *Camta2* | 0.95403919 | 0.017837201 | *Zswim6* | 0.877894753 | 0.000586918 |

| **Supplemental Table S6D DEGS LAC1 Cluster; VCD/Vehicle.** Maroon=upregulated; | | | | | | | | |
| --- | --- | --- | --- | --- | --- | --- | --- | --- |
| **gene** | **avg_log2FC** | **p_val** | **gene** | **avg_log2FC** | **p_val** | **gene** | **avg_log2FC** | **p_val** |
| *Pigt* | 0.87539874 | 0.042167664 | *Pola1* | 0.809125521 | 0.007911853 | *Pkn1* | 0.754677737 | 0.042512374 |
| *Ubr4* | 0.871577029 | 0.000787618 | *Kcnab1* | 0.805009412 | 0.003220737 | *Cep57l1* | 0.75383676 | 0.040130634 |
| *Hdgfl2* | 0.871577029 | 0.043640457 | *Bmi1* | 0.805009412 | 0.030511447 | *Tmem258* | 0.752886681 | 0.005867405 |
| *Srp19* | 0.869412445 | 0.001958468 | *Pex11g* | 0.805009412 | 0.038093224 | *Lrrc45* | 0.752886681 | 0.012454097 |
| *Ubtf* | 0.861592941 | 0.014511136 | *Fancl* | 0.803248955 | 0.015816269 | *Atg4b* | 0.752886681 | 0.036458314 |
| *Fhitos* | 0.861592941 | 0.021189721 | *Tmx4* | 0.801187702 | 0.041948018 | *Prkab2* | 0.752886681 | 0.036805468 |
| *Extl3* | 0.861592941 | 0.022646319 | *Sdc2* | 0.800723873 | 9.08E-06 | *Rem2* | 0.751804795 | 0.019465313 |
| *Pabpc4* | 0.861592941 | 0.026897851 | *Pde1c* | 0.800192396 | 0.006345417 | *Ndufa1* | 0.751804795 | 0.028189391 |
| *Mkx* | 0.861592941 | 0.028243619 | *Sf3a3* | 0.800192396 | 0.050908612 | *Atn1* | 0.751804795 | 0.033144292 |
| *Iqsec1* | 0.857292803 | 0.031657622 | *U2af2* | 0.800192396 | 0.037564144 | *Rab1a* | 0.750561628 | 0.000402347 |
| *Noc2l* | 0.854898288 | 0.023790327 | *Rnf217* | 0.799847707 | 0.01423608 | *Ift88* | 0.750561628 | 0.006722321 |
| *Stimate* | 0.854898288 | 0.033589823 | *Tpm3* | 0.799180334 | 0.010529934 | *Smc6* | 0.749118212 | 0.036277788 |
| *Psmd1* | 0.852875724 | 0.000117083 | *Erbin* | 0.798857185 | 0.017760452 | *Prmt3* | 0.747421921 | 0.002760902 |
| *Dgkh* | 0.852006892 | 4.03E-05 | *Rad23a* | 0.796497913 | 0.023959076 | *Dlst* | 0.747421921 | 0.005513916 |
| *Prorsd1* | 0.850945697 | 0.050714476 | *Arntl* | 0.794478745 | 0.000834349 | *Znfx1* | 0.747421921 | 0.018101005 |
| *Pitpnm1* | 0.849101997 | 0.013426798 | *Abcc4* | 0.793574517 | 0.007372774 | *Thap3* | 0.747421921 | 0.02268153 |
| *Ksr1* | 0.849101997 | 0.020789079 | *Rps29* | 0.79293658 | 2.04E-06 | *Hdac4* | 0.744478073 | 3.17E-05 |
| *Cdc23* | 0.849101997 | 0.046835845 | *Txndc12* | 0.791203613 | 0.033226157 | *Ndst3* | 0.74423599 | 0.012727932 |
| *Eefsec* | 0.848336844 | 0.011521851 | *Mpv17l* | 0.791203613 | 0.033514824 | *Sox2ot* | 0.743821482 | 0.048601024 |
| *Cep104* | 0.846486048 | 0.020436903 | *Galnt3* | 0.790178608 | 0.015104204 | *Cs* | 0.742948444 | 0.010897784 |
| *Ciao3* | 0.846486048 | 0.027930688 | *Zcchc9* | 0.789242097 | 0.026850183 | *Mier3* | 0.742948444 | 0.039014387 |
| *Cog4* | 0.846486048 | 0.03081485 | *Ttll11* | 0.789242097 | 0.035882231 | *Crnkl1* | 0.741516489 | 0.00865231 |
| *Dnajb4* | 0.844719122 | 0.002417057 | *Htatip2* | 0.787592359 | 0.03975932 | *Ptpn11* | 0.739914384 | 0.003255943 |
| *Eif4a3* | 0.842484118 | 0.015173065 | *Rnf150* | 0.786185538 | 0.015077941 | *Tceal3* | 0.738109887 | 0.013470625 |
| *Cnih4* | 0.842484118 | 0.018325478 | *Dpm3* | 0.786185538 | 0.026484116 | *Srp68* | 0.738109887 | 0.017917094 |
| *Gm28198* | 0.842484118 | 0.019416432 | *Usp36* | 0.785761528 | 0.000773754 | *Brf1* | 0.738109887 | 0.029338525 |
| *Erlin1* | 0.842484118 | 0.02330803 | *Stx2* | 0.784971659 | 0.035647997 | *Arhgap42* | 0.737456641 | 0.028748462 |
| *Tyk2* | 0.840633322 | 0.029480471 | *Adgrl1* | 0.783433878 | 0.003290107 | *Alg12* | 0.736062059 | 0.028196867 |
| *Bbs4* | 0.840633322 | 0.031862978 | *Rexo1* | 0.782983106 | 0.052054412 | *Smg9* | 0.736062059 | 0.031855481 |
| *Odr4* | 0.840633322 | 0.004518776 | *Ywhab* | 0.780165341 | 0.011271802 | *Fbxo38* | 0.732859627 | 0.027653653 |
| *Iws1* | 0.839566634 | 0.015297998 | *Ptpro* | 0.777280485 | 4.81E-06 | *Dnmt1* | 0.731008831 | 0.011580699 |
| *Stat2* | 0.835597732 | 0.046622689 | *Morc2a* | 0.775863067 | 0.004244497 | *4930512B01Rik* | 0.731008831 | 0.014907321 |
| *Ppp2r3c* | 0.835597732 | 0.050597375 | *Ppme1* | 0.775643328 | 0.009071309 | *Hcfc2* | 0.731008831 | 0.035581632 |
| *Polr3h* | 0.835597732 | 0.006680881 | *Mast4* | 0.769446939 | 2.60E-09 | *Xrn2* | 0.731008831 | 0.010107374 |
| *Rab3a* | 0.835597732 | 0.012653341 | *Pikfyve* | 0.768483536 | 0.001394693 | *Klf9* | 0.729262227 | 0.045978448 |
| *Cbarp* | 0.832613874 | 0.000796514 | *Rnd3* | 0.768483536 | 0.002220009 | *Sec23ip* | 0.728955172 | 0.018300367 |
| *Glud1* | 0.832613874 | 0.00493317 | *Rad18* | 0.768483536 | 0.032008235 | *Sugp2* | 0.727841552 | 0.017533726 |
| *Csrp1* | 0.831219292 | 0.036145939 | *Maml1* | 0.768483536 | 0.04646035 | *Gm26658* | 0.726964267 | 0.009906505 |
| *Syncrip* | 0.830640012 | 0.001802568 | *Btbd10* | 0.768483536 | 0.001932347 | *Rrbp1* | 0.724089417 | 1.64E-06 |
| *Usp20* | 0.828856154 | 0.004892215 | *Ak5* | 0.768483536 | 0.003428787 | *Hsph1* | 0.724089417 | 0.000683694 |
| *Ecd* | 0.827377225 | 0.011089636 | *Ptrh2* | 0.768483536 | 0.003992825 | *Thra* | 0.724089417 | 0.016853921 |
| *Msl1* | 0.827377225 | 0.020678663 | *Abca2* | 0.768483536 | 0.016674469 | *Zfp423* | 0.719573936 | 0.017223228 |
| *Dync1li1* | 0.827377225 | 0.039686868 | *Dhx32* | 0.768483536 | 0.018224137 | *Slc35b1* | 0.719573936 | 0.036535017 |
| *Thrb* | 0.826116679 | 5.51E-08 | *Sp1* | 0.768483536 | 0.02170478 | *Sntb1* | 0.718852769 | 0.000825827 |
| *Zfhx2* | 0.825067065 | 0.001249747 | *Trp53inp2* | 0.768483536 | 0.032819729 | *Odc1* | 0.718558311 | 0.018006913 |
| *Agpat5* | 0.825067065 | 0.03003357 | *G2e3* | 0.768483536 | 0.036292346 | *Bcl2l11* | 0.717857463 | 0.012300264 |
| *Ncoa5* | 0.82293132 | 0.01871947 | *Rnpepl1* | 0.768483536 | 0.047243509 | *Arrdc3* | 0.717857463 | 0.034038528 |
| *Traf3* | 0.8221215 | 0.004624634 | *Srxn1* | 0.768483536 | 0.047820443 | *Tbc1d20* | 0.716953236 | 0.032513263 |
| *Rimkla* | 0.820950956 | 0.001016534 | *Hmox2* | 0.768483536 | 0.051220863 | *Chpf2* | 0.716395025 | 0.029373081 |
| *Srprb* | 0.820950956 | 0.021872227 | *Pstpip2* | 0.768483536 | 0.051220863 | *Mid2* | 0.716016117 | 0.014725723 |
| *Actr1a* | 0.820950956 | 0.024070598 | *Fosb* | 0.761386063 | 3.26E-08 | *Sox5* | 0.7153722 | 0.02732641 |
| *Fancm* | 0.820950956 | 0.024340451 | *Cacna1g* | 0.760829964 | 0.001671852 | *Chd3* | 0.714035752 | 0.033426516 |
| *Mios* | 0.820950956 | 0.026285589 | *6430548M08Rik* | 0.760309605 | 0.004227339 | *Sirt2* | 0.714035752 | 0.045786437 |
| *Srsf9* | 0.820950956 | 0.046661747 | *Tes* | 0.758215201 | 0.00883054 | *BC029722* | 0.714035752 | 0.049789799 |
| *Syt4* | 0.819287907 | 1.12E-05 | *Dop1b* | 0.757991163 | 0.000268158 | *Tmtc1* | 0.712070228 | 0.000382487 |
| *Tmem179* | 0.817393137 | 0.049317757 | *P4ha1* | 0.755772485 | 0.000234429 | *Prdm10* | 0.709589847 | 0.004228701 |
| *Map9* | 0.815789251 | 0.020909096 | *mt-Nd4l* | 0.755247709 | 2.67E-05 | *Tubb2a* | 0.709589847 | 0.0112393 |

| **Supplemental Table S6E DEGS LAC1 Cluster; VCD/Vehicle.** Maroon=upregulated; | | | | | | | | |
| --- | --- | --- | --- | --- | --- | --- | --- | --- |
| **gene** | **avg_log2FC** | **p_val** | **gene** | **avg_log2FC** | **p_val** | **gene** | **avg_log2FC** | **p_val** |
| *Nfatc3* | 0.708982525 | 0.00605959 | *Camk1g* | 0.668947863 | 0.024796462 | *Med25* | 0.619620151 | 0.017119669 |
| *Stt3b* | 0.708982525 | 0.0099543 | *Gm17435* | 0.668947863 | 0.04960525 | *Nr4a3* | 0.619317095 | 0.041314136 |
| *Hnrnpul2* | 0.70861208 | 0.011777049 | *Pus7* | 0.665913803 | 0.026571785 | *Serpinh1* | 0.61792386 | 0.022407358 |
| *Rnf10* | 0.707082992 | 0.003031053 | *Snx12* | 0.665053947 | 0.008907106 | *Gpatch2l* | 0.61792386 | 0.024558583 |
| *Hdac7* | 0.707082992 | 0.040084525 | *Cic* | 0.664146877 | 0.035660286 | *Vps37b* | 0.61792386 | 0.040370679 |
| *Capns1* | 0.705473739 | 0.011406436 | *Serinc3* | 0.664146877 | 0.03772631 | *Myh10* | 0.617174213 | 0.009702155 |
| *Atg5* | 0.705473739 | 0.015976876 | *Cenpc1* | 0.663130536 | 0.002975192 | *Atp9a* | 0.616480443 | 0.00342588 |
| *Ptprs* | 0.705058156 | 4.85E-09 | *Zmym3* | 0.662688872 | 0.052896332 | *Edc4* | 0.616480443 | 0.011047927 |
| *Slc30a4* | 0.704353199 | 0.02741355 | *Prepl* | 0.662174634 | 0.004909729 | *Atp8b2* | 0.616480443 | 0.024956035 |
| *Uso1* | 0.70399481 | 3.91E-05 | *Ubl5* | 0.662174634 | 0.043085056 | *Fndc9* | 0.615471918 | 0.021511019 |
| *Naa40* | 0.702895195 | 0.015581254 | *Cd47* | 0.661100001 | 0.000524251 | *Rps27* | 0.615009056 | 0.017122923 |
| *Alg13* | 0.702181166 | 0.00037637 | *Zswim8* | 0.659440777 | 0.002241248 | *Gls* | 0.614860347 | 0.005418559 |
| *Syne2* | 0.701988125 | 0.000957213 | *Rerg* | 0.658429991 | 0.04024863 | *Sin3b* | 0.61415539 | 0.049049958 |
| *Derl1* | 0.700096561 | 0.002007629 | *Ppa1* | 0.65558948 | 0.026697976 | *Gm15520* | 0.61415539 | 0.052003343 |
| *Ercc6l2* | 0.699770786 | 0.0019458 | *Tuba1a* | 0.655025487 | 0.003679262 | *Dbpht2* | 0.613508876 | 0.055066546 |
| *Ttf1* | 0.698094209 | 0.017467971 | *Gm17106* | 0.654826755 | 0.049017609 | *Acap2* | 0.613385953 | 0.005798531 |
| *Hsf1* | 0.698094209 | 0.047575343 | *Abhd6* | 0.653006319 | 0.001147803 | *Nkain2* | 0.612458226 | 0.001132454 |
| *Exosc10* | 0.698094209 | 0.043755807 | *Jun* | 0.65091394 | 9.86E-05 | *Tnpo2* | 0.612364335 | 0.047130274 |
| *Gcn1* | 0.694482955 | 0.021271219 | *Chn1* | 0.650647046 | 0.014120997 | *Htt* | 0.611202744 | 0.001156815 |
| *Klf13* | 0.694482955 | 0.045622382 | *Rpap2* | 0.650647046 | 0.043062295 | *Rfc1* | 0.610942259 | 0.026389606 |
| *Pde10a* | 0.694224662 | 6.31E-17 | *Fryl* | 0.65030211 | 0.00262266 | *Nup98* | 0.610942259 | 0.000882388 |
| *Celf3* | 0.694136195 | 0.001791197 | *Ppfia1* | 0.648643766 | 0.002676666 | *Eif4a1* | 0.607328745 | 0.006484214 |
| *Cry2* | 0.693382081 | 0.004993746 | *Sfswap* | 0.64848376 | 0.000573375 | *Srsf2* | 0.607020114 | 0.003158531 |
| *Myrip* | 0.690481024 | 0.00065613 | *Grik5* | 0.646147994 | 0.017713623 | *Hdlbp* | 0.605930524 | 0.00045331 |
| *Tdp1* | 0.690481024 | 0.045163624 | *Ptov1* | 0.644852187 | 0.005277178 | *Cdon* | 0.605754036 | 0.051871049 |
| *Itgb1* | 0.689161084 | 0.002545171 | *Elavl1* | 0.644852187 | 0.006640443 | *Usp13* | 0.604984804 | 0.007830718 |
| *Acbd6* | 0.689161084 | 0.006963014 | *Uck2* | 0.644425544 | 0.004635151 | *Xbp1* | 0.60342429 | 0.003500116 |
| *Prkcsh* | 0.688756344 | 0.050571269 | *Nop56* | 0.644250626 | 0.011223675 | *Slc10a7* | 0.60342429 | 0.009508053 |
| *Mia3* | 0.686021376 | 1.44E-05 | *Dnajb14* | 0.642952654 | 0.036405028 | *Nsmaf* | 0.603235022 | 0.003959121 |
| *Ppil4* | 0.686021376 | 0.018567175 | *Gm56714* | 0.642952654 | 0.037176999 | *Fbxw4* | 0.603235022 | 0.0145604 |
| *Tsr2* | 0.686021376 | 0.029024768 | *Abcb1b* | 0.64151068 | 0.002738311 | *Zmiz1* | 0.598558535 | 0.003771708 |
| *Nit1* | 0.686021376 | 0.038892189 | *Gm4876* | 0.640378711 | 0.002850685 | *Ezr* | 0.598558535 | 0.00448932 |
| *Brd1* | 0.684715179 | 0.010322598 | *Cep164* | 0.639585803 | 0.049767895 | *Vps8* | 0.598558535 | 0.009227884 |
| *Dgka* | 0.683950026 | 0.005578519 | *Cadps2* | 0.638242236 | 4.05E-07 | *Rangap1* | 0.598558535 | 0.011804556 |
| *Xpnpep1* | 0.683092045 | 0.010165187 | *Txndc5* | 0.638086899 | 0.026268818 | *Fam110b* | 0.598558535 | 0.017640749 |
| *Dctn3* | 0.683092045 | 0.031838252 | *Rpl37a* | 0.637920967 | 4.38E-06 | *Plxnb1* | 0.598558535 | 0.020509407 |
| *Pms2* | 0.683092045 | 0.035771944 | *Cfap20* | 0.637239003 | 0.005519478 | *Qser1* | 0.598558535 | 0.022122714 |
| *Clptm1l* | 0.681020695 | 0.01367587 | *Cep68* | 0.637239003 | 0.030454785 | *Clstn3* | 0.598558535 | 0.031299239 |
| *Rnf128* | 0.681020695 | 0.024054246 | *Efemp1* | 0.63603324 | 0.012398745 | *Fkbp14* | 0.598558535 | 0.049493141 |
| *Clasrp* | 0.681020695 | 0.051800565 | *Zfp869* | 0.63603324 | 0.045584903 | *Ppid* | 0.598558535 | 0.052193076 |
| *Stard13* | 0.680352626 | 0.003858836 | *Trrap* | 0.635509671 | 0.004769243 | *Mamdc2* | 0.595418827 | 0.011290277 |
| *Abce1* | 0.67947853 | 0.018689418 | *Osbp* | 0.635217006 | 0.005013581 | *Meis2* | 0.594716469 | 0.019500902 |
| *Fam76b* | 0.678285727 | 0.017726894 | *Ptprn* | 0.634981943 | 3.53E-05 | *Irs2* | 0.594418805 | 0.000184262 |
| *Flot2* | 0.678285727 | 0.050123985 | *Chd8* | 0.633046911 | 0.003496807 | *Dpysl2* | 0.592633701 | 0.02260984 |
| *Celsr3* | 0.677335648 | 0.001599759 | *Sirt3* | 0.630980013 | 0.024855023 | *Cert1* | 0.591985881 | 0.006258632 |
| *Plpp5* | 0.676561047 | 0.033002786 | *Eif2ak3* | 0.629922706 | 0.012581739 | *Eml1* | 0.591605774 | 0.034568863 |
| *Senp5* | 0.675917418 | 0.009151666 | *Crppa* | 0.629585431 | 0.04407365 | *Calr* | 0.591398743 | 0.007982838 |
| *Hspa5* | 0.675784047 | 0.008522073 | *Calu* | 0.626464532 | 0.02357923 | *Serf2* | 0.590945351 | 0.005215191 |
| *Chordc1* | 0.674507388 | 0.010274986 | *Ndufv3* | 0.626039271 | 0.016149167 | *Clybl* | 0.58990407 | 9.29E-05 |
| *Strada* | 0.673846662 | 0.004727161 | *Tiparp* | 0.626039271 | 0.000868925 | *Cops2* | 0.589513395 | 0.029712613 |
| *Zkscan1* | 0.673846662 | 0.028232127 | *Chp1* | 0.625525583 | 0.011283197 | *Ube2n* | 0.588777514 | 0.025122341 |
| *Dnajb6* | 0.673571889 | 0.004229838 | *Ptp4a2* | 0.625030746 | 0.022559406 | *Sipa1l2* | 0.588362768 | 0.000364468 |
| *Ddah1* | 0.672559116 | 0.032616321 | *Bora* | 0.624093627 | 0.033361009 | *1810037I17Rik* | 0.587911291 | 0.052275826 |
| *Atp2a2* | 0.671186335 | 0.000190214 | *Tafa2* | 0.622942694 | 1.16E-06 | *Sorbs1* | 0.58577238 | 6.96E-05 |
| *2900026A02Rik* | 0.668947863 | 0.016738385 | *Slc38a2* | 0.622165148 | 0.000395834 | *Eif4h* | 0.585619479 | 0.007540927 |
| *Tsg101* | 0.668947863 | 0.026713102 | *Pik3r3* | 0.621442426 | 0.001156682 | *Prpf38b* | 0.585619479 | 0.007985731 |
| *Gapdh* | 0.668947863 | 0.039704585 | *Unc5c* | 0.620384897 | 0.001182344 | *Stk33* | 0.583324021 | 0.008261899 |

| **Supplemental Table S6F DEGS LAC1 Cluster; VCD/Vehicle.** Maroon=upregulated; Blue=downregulated | | | | | | | | |
| --- | --- | --- | --- | --- | --- | --- | --- | --- |
| **gene** | **avg_log2FC** | **p_val** | **gene** | **avg_log2FC** | **p_val** | **gene** | **avg_log2FC** | **p_val** |
| *Gtf2ird1* | 0.58269213 | 0.000108452 | *Ramac* | -1.138407059 | 0.053176302 | *Gm19605* | -1.623833886 | 0.036188028 |
| *Slc6a17* | 0.582510594 | 0.000765294 | *Scn10a* | -1.160433366 | 0.045651116 | *Gm3363* | -1.664475871 | 0.034803755 |
| *Kdm4b* | 0.582070412 | 0.050034544 | *A230004M16Rik* | -1.164402268 | 0.025247691 | *Gm15559* | -1.664475871 | 0.051964028 |
| *Yy1* | 0.581280544 | 0.00505774 | *Lancl2* | -1.170115919 | 0.027995425 | *4930509H03Rik* | -1.690948082 | 0.011976324 |
| *Sestd1* | 0.580856533 | 0.028641833 | *Tomm22* | -1.179049044 | 0.033445666 | *Il1r1* | -1.70956376 | 0.009945067 |
| *Snhg8* | 0.580856533 | 0.047902083 | *Rara* | -1.179049044 | 0.048948702 | *Sarm1* | -1.72336956 | 0.014328057 |
|  |  |  | *Prrg1* | -1.194990588 | 8.02E-07 | *Rpusd1* | -1.72336956 | 0.035629724 |
| *Mff* | -0.585866036 | 0.031657834 | *Pcx* | -1.218577408 | 0.019720772 | *Sdhd* | -1.75507842 | 0.032243921 |
| *Kcnip4* | -0.587563937 | 1.30E-05 | *Pkd2l2* | -1.231516464 | 0.011218927 | *Gm2516* | -1.816478964 | 0.001153364 |
| *Vezf1* | -0.619787053 | 0.048319191 | *Zfp2* | -1.231516464 | 0.049487539 | *Cfap46* | -1.816478964 | 0.015202153 |
| *Col25a1* | -0.654149511 | 8.85E-06 | *Zfhx2os* | -1.249438372 | 0.035230635 | *AU022252* | -1.816478964 | 0.023427686 |
| *Dennd2a* | -0.664475871 | 0.039150002 | *Senp8* | -1.257051556 | 0.0124305 | *Gcat* | -1.816478964 | 0.035421236 |
| *Gm19938* | -0.696184731 | 0.032312275 | *Dele1* | -1.263937941 | 0.015886088 | *Slc27a3* | -1.816478964 | 0.051107669 |
| *Ccnc* | -0.702822182 | 0.027482769 | *Dnajc27* | -1.275910583 | 0.007870305 | *Gm26648* | -1.816478964 | 0.051107669 |
| *Stmn3* | -0.706854473 | 0.006361781 | *Ndufaf5* | -1.275910583 | 0.015299364 | *Mfap3l* | -1.816478964 | 0.051107669 |
| *Csmd3* | -0.714494932 | 4.65E-06 | *Smyd4* | -1.294526261 | 0.020866403 | *Dynlt1c* | -1.816478964 | 0.051107669 |
| *Slc35f4* | -0.72813949 | 2.09E-08 | *Lin9* | -1.301905791 | 0.036643464 | *Fahd2a* | -1.903941806 | 0.023427686 |
| *4930555F03Rik* | -0.752348627 | 0.012622539 | *Slc25a47* | -1.301905791 | 0.036643464 | *Sspn* | -1.931956182 | 0.011943241 |
| *Dars* | -0.774658789 | 0.048973096 | *Dock11* | -1.31290479 | 0.000809152 | *St6galnac6* | -1.931956182 | 0.014190136 |
| *Rtn4* | -0.782127459 | 0.009731959 | *Alg1* | -1.318979305 | 0.048525482 | *Gm15713* | -1.931956182 | 0.035339236 |
| *Ap3m1* | -0.801048924 | 0.019567165 | *Dffa* | -1.331052137 | 0.020930432 | *Dnaaf2* | -1.986403966 | 0.023278415 |
| *Gm9968* | -0.856007328 | 0.013213884 | *Vps26c* | -1.346993681 | 0.034883244 | *Lzts1* | -1.986403966 | 0.031272788 |
| *Serpinb6a* | -0.859123302 | 0.030117143 | *Mlip* | -1.352531865 | 0.001922916 | *Clba1* | -1.986403966 | 0.031272788 |
| *Tox3* | -0.859980603 | 0.001667912 | *Oprm1* | -1.369019987 | 0.002521202 | *Gm49460* | -1.986403966 | 0.031272788 |
| *Gm19522* | -0.865947641 | 0.009793829 | *Gm2694* | -1.401441465 | 0.015156112 | *Nudt1* | -1.986403966 | 0.051037734 |
| *Ppp1r1c* | -0.872332199 | 0.025798867 | *C79798* | -1.401441465 | 0.023832477 | *Irak2* | -1.986403966 | 0.051037734 |
| *Rnf114* | -0.88359316 | 0.031558716 | *Smpdl3a* | -1.401441465 | 0.023832477 | *Kcnc1* | -1.986403966 | 0.051037734 |
| *Ddb1* | -0.889235307 | 0.042850996 | *Mief1* | -1.401441465 | 0.024544808 | *Kcnn1* | -1.986403966 | 0.051037734 |
| *Gm45321* | -0.894481476 | 0.017159451 | *Styxl1* | -1.401441465 | 0.035837095 | *4833418N02Rik* | -1.986403966 | 0.051037734 |
| *Osbpl1a* | -0.916014638 | 0.042132369 | *Gm11454* | -1.401441465 | 0.036529995 | *Sag* | -2.038871386 | 0.008951484 |
| *Gm3693* | -0.923394168 | 0.016644018 | *Glb1l2* | -1.401441465 | 0.051521728 | *Gmpr2* | -2.038871386 | 0.014153901 |
| *Gm16279* | -0.931956182 | 0.036585689 | *Dnah2* | -1.401441465 | 0.051521728 | *Noa1* | -2.038871386 | 0.022356285 |
| *Ap2s1* | -0.931956182 | 0.045014677 | *9330111N05Rik* | -1.401441465 | 0.051521728 | *Mtmr10* | -2.038871386 | 0.035257296 |
| *Aimp1* | -0.936773198 | 0.02708724 | *4933404O12Rik* | -1.401441465 | 0.052540928 | *Angptl6* | -2.138407059 | 0.014119788 |
| *Irak1bp1* | -0.947723498 | 0.040023484 | *Cdk5r1* | -1.453908885 | 0.024544795 | *Pih1d2* | -2.138407059 | 0.031228922 |
| *Tmeff2* | -0.955882021 | 0.008402158 | *Gm11613* | -1.460335154 | 0.018956364 | *Gm14004* | -2.138407059 | 0.050969368 |
| *Scml4* | -0.986403966 | 0.0106995 | *Rab39b* | -1.479443977 | 0.032969861 | *St8sia1* | -2.138407059 | 0.050969368 |
| *Gm26549* | -0.986403966 | 0.026453718 | *Or4e1* | -1.479443977 | 0.03513965 | *1700125H03Rik* | -2.138407059 | 0.050969368 |
| *Ppp1cc* | -0.986403966 | 0.047372 | *Pard6a* | -1.479443977 | 0.048110214 | *BC048559* | -2.275910583 | 0.019166727 |
| *Flywch1* | -1.005512789 | 0.019702715 | *Gm45341* | -1.488904306 | 0.008702017 | *Serpinb8* | -2.275910583 | 0.03118651 |
| *Sac3d1* | -1.009124042 | 0.009220817 | *Gpr18* | -1.494550869 | 0.035069434 | *Gm19412* | -2.275910583 | 0.03118651 |
| *Ccdc171* | -1.009124042 | 0.02813832 | *Fam151b* | -1.494550869 | 0.052348171 | *Gm47691* | -2.401441465 | 0.005597471 |
| *Cryz* | -1.027983069 | 0.026597014 | *Myo3b* | -1.504534958 | 0.009696301 | *Kcnh7* | -2.401441465 | 0.01914043 |
| *Nrip2* | -1.064406478 | 0.044747058 | *Ctsc* | -1.526972347 | 0.043116497 | *Gm20700* | -2.401441465 | 0.01914043 |
| *C130073E24Rik* | -1.064825927 | 3.91E-10 | *Slc16a4* | -1.532685998 | 0.032710268 | *1700015H07Rik* | -2.516918682 | 0.007271152 |
| *Psmb7* | -1.075213233 | 0.009073714 | *Mir124a-1hg* | -1.553444558 | 0.006113813 | *1700086P04Rik* | -2.516918682 | 0.0191141 |
| *4930447N08Rik* | -1.105985581 | 0.050064848 | *Gm13780* | -1.553444558 | 0.023335756 | *Sord* | -2.516918682 | 0.050900944 |
| *Wdr64* | -1.116039246 | 0.018175157 | *Gm4756* | -1.553444558 | 0.05145241 | *Wrap53* | -2.623833886 | 0.007261045 |
| *Dlgap2* | -1.116039246 | 0.008084811 | *Gm4219* | -1.553444558 | 0.051452702 | *D11Wsu47e* | -2.623833886 | 0.011776786 |
| *Tenm3* | -1.118598578 | 3.09E-05 | *Gm36037* | -1.553444558 | 0.052206831 | *Alkbh7* | -2.623833886 | 0.0310591 |
| *Champ1* | -1.138407059 | 0.027914957 | *Fshb* | -1.566500711 | 0.001974442 | *Gm19990* | -2.816478964 | 0.012840126 |
| *Zbbx* | -1.138407059 | 0.034701898 | *Brms1* | -1.571366466 | 0.012957531 | *Tonsl* | -2.816478964 | 0.012840126 |
| *Neu1* | -1.138407059 | 0.035656678 | *Gm29296* | -1.582013711 | 0.003887453 | *Rs1* | -2.816478964 | 0.012840126 |
| *Mta2* | -1.138407059 | 0.040421724 | *Zfp90* | -1.582013711 | 0.034936433 | *4930550C14Rik* | -2.816478964 | 0.019087714 |
| *Rad52* | -1.138407059 | 0.043909514 | *Poli* | -1.623833886 | 0.022046748 | *1700080N15Rik* | -2.986403966 | 0.012839826 |
| *Rbmxl1* | -1.138407059 | 0.049162989 | *Fiz1* | -1.623833886 | 0.023581439 | *2310047K21Rik* | -2.986403966 | 0.012839826 |
| *Wdr81* | -1.138407059 | 0.051591397 | *Gm44899* | -1.623833886 | 0.036188028 | *Gm36640* | -2.986403966 | 0.012839826 |

| **Supplemental Table S6G DEGS LAC1 Cluster; VCD/Vehicle.** Maroon=upregulated; Blue=downregulated | | | | | | | | |
| --- | --- | --- | --- | --- | --- | --- | --- | --- |
| **gene** | **avg_log2FC** | **p_val** | **gene** | **avg_log2FC** | **p_val** | **gene** | **avg_log2FC** | **p_val** |
| *Trim43c* | -2.986403966 | 0.012839826 |  |  |  |  |  |  |
| *4930428N03Rik* | -2.986403966 | 0.012839826 |  |  |  |  |  |  |
| *Gucy1b2* | -2.986403966 | 0.012839826 |  |  |  |  |  |  |
| *Pipox* | -3.275910583 | 0.0047458 |  |  |  |  |  |  |
| *Gm56522* | -3.275910583 | 0.007787548 |  |  |  |  |  |  |
| *Gm15486* | -3.516918682 | 0.00778762 |  |  |  |  |  |  |
| *Gm44601* | -3.623833886 | 0.001781126 |  |  |  |  |  |  |
| *Gm19744* | -3.623833886 | 0.004745998 |  |  |  |  |  |  |
| *Pdk2* | -3.986403966 | 0.000416534 |  |  |  |  |  |  |
|  |  |  |  |  |  |  |  |  |

Supplemental Table 6: Differentially expressed genes in LAC1 cluster.

| **Supplemental Table S7A DEGS LAC2 Cluster; VCD/Vehicle.** Maroon=upregulated; | | | | | | | | |
| --- | --- | --- | --- | --- | --- | --- | --- | --- |
| **gene** | **avg_log2FC** | **p_val** | **gene** | **avg_log2FC** | **p_val** | **gene** | **avg_log2FC** | **p_val** |
| *Ptgs2* | 3.645159063 | 0.008286184 | *Gpr3* | 2.282588984 | 0.018340523 | *Cdt1* | 2.060196562 | 0.039000569 |
| *Lpcat4* | 3.475234061 | 0.001766867 | *Miip* | 2.282588984 | 0.018340523 | *Gm28085* | 2.060196562 | 0.039000569 |
| *Pola2* | 3.475234061 | 0.001766867 | *Glt1d1* | 2.282588984 | 0.018340523 | *Cd68* | 2.060196562 | 0.039000569 |
| *Prss29* | 3.475234061 | 0.001766867 | *Kcnc1* | 2.282588984 | 0.018340523 | *Hsf5* | 2.060196562 | 0.039000569 |
| *Gm15687* | 3.475234061 | 0.003808948 | *Gm44649* | 2.282588984 | 0.018340523 | *Xrcc3* | 2.060196562 | 0.039000569 |
| *Mfsd2a* | 3.282588984 | 0.003809035 | *Sprn* | 2.282588984 | 0.018340523 | *Lratd2* | 2.060196562 | 0.039000569 |
| *Slc25a29* | 3.282588984 | 0.003809035 | *Ddx28* | 2.282588984 | 0.018340523 | *Gm29994* | 2.060196562 | 0.039000569 |
| *Gm50020* | 3.282588984 | 0.003809035 | *Gm16853* | 2.282588984 | 0.018340523 | *Ehbp1l1* | 2.060196562 | 0.039000569 |
| *Anxa11* | 3.282588984 | 0.008286127 | *Oxsm* | 2.282588984 | 0.018340523 | *Pank1* | 2.060196562 | 0.039000569 |
| *Platr21* | 3.17567378 | 0.000209334 | *Gm48933* | 2.282588984 | 0.018340523 | *Tbc1d1* | 1.993962864 | 1.02E-08 |
| *Wfdc2* | 3.17567378 | 0.038798299 | *1700024B18Rik* | 2.282588984 | 0.018340523 | *Rnf217* | 1.961478511 | 2.04E-13 |
| *2010009K17Rik* | 3.060196562 | 0.008286222 | *Gm49172* | 2.282588984 | 0.018340523 | *1810014B01Rik* | 1.960660889 | 0.003565457 |
| *Spaca1* | 3.060196562 | 0.008286222 | *Pcdhga8* | 2.282588984 | 0.018340523 | *E2f6* | 1.94916525 | 0.000116342 |
| *Arhgef19* | 3.060196562 | 0.008286222 | *Sntb2* | 2.282588984 | 0.038932603 | *Mtbp* | 1.93466568 | 0.003475245 |
| *Lat2* | 3.060196562 | 0.008286222 | *Nlgn2* | 2.240768808 | 0.000487167 | *Usp21* | 1.93466568 | 0.009961213 |
| *Sap25* | 3.060196562 | 0.008286222 | *Zc3h8* | 2.240768808 | 0.000946667 | *Lcmt2* | 1.93466568 | 0.009961213 |
| *Gm47113* | 3.060196562 | 0.008286222 | *Slc19a2* | 2.212199656 | 0.006953393 | *Gm10130* | 1.93466568 | 0.009961213 |
| *Gm11762* | 3.060196562 | 0.008286222 | *Gm11817* | 2.212199656 | 0.006953393 | *Gm17690* | 1.93466568 | 0.009961213 |
| *Cmpk2* | 3.060196562 | 0.008286222 | *Otud3* | 2.212199656 | 0.006953393 | *Isoc2b* | 1.93466568 | 0.009961213 |
| *Gm56549* | 3.060196562 | 0.008286222 | *Brca2* | 2.212199656 | 0.006953393 | *Vgf* | 1.922693038 | 0.000541641 |
| *4930594M22Rik* | 3.060196562 | 0.008286222 | *Srp54c* | 2.212199656 | 0.006953393 | *Pus3* | 1.915806653 | 0.00088172 |
| *Thpo* | 3.060196562 | 0.008286222 | *Il17d* | 2.212199656 | 0.006953393 | *Ctdspl* | 1.915806653 | 0.002997695 |
| *Cbr3* | 3.060196562 | 0.008286222 | *4930447N08Rik* | 2.212199656 | 0.014032822 | *Slco5a1* | 1.908193469 | 0.022672127 |
| *Gpr83* | 3.060196562 | 0.008286222 | *Alpk1* | 2.212199656 | 0.028166184 | *Larp6* | 1.890271561 | 0.002439907 |
| *Gm49490* | 3.060196562 | 0.008286222 | *H3c15* | 2.191441095 | 6.72E-05 | *Snx8* | 1.890271561 | 0.004545256 |
| *Mrps22* | 2.977734402 | 4.58E-05 | *Tedc2* | 2.17567378 | 0.002579773 | *Gm57180* | 1.890271561 | 0.028334502 |
| *Gm42477* | 2.93466568 | 0.00092326 | *Acsl4* | 2.17567378 | 0.002579773 | *Gm57465* | 1.890271561 | 0.028334502 |
| *Gm56813* | 2.797162156 | 0.001942833 | *Mfsd4a* | 2.17567378 | 0.005065532 | *Rad51* | 1.890271561 | 0.028334502 |
| *Cyp2s1* | 2.797162156 | 0.001942833 | *Pcp4* | 2.163290055 | 3.64E-06 | *Cngb3* | 1.890271561 | 0.028334502 |
| *Dgat2* | 2.797162156 | 0.001942833 | *D030028A08Rik* | 2.138199074 | 0.000351065 | *Gm12404* | 1.890271561 | 0.028334502 |
| *Cfp* | 2.797162156 | 0.001942833 | *Apoe* | 2.127310758 | 0.030915873 | *Rflna* | 1.890271561 | 0.028334502 |
| *Nxn* | 2.797162156 | 0.003375391 | *Slc4a7* | 2.060196562 | 0.001632525 | *Elmod3* | 1.890271561 | 0.028334502 |
| *Arpc1b* | 2.797162156 | 0.004088066 | *Gm57315* | 2.060196562 | 0.003545081 | *Chek1* | 1.890271561 | 0.028334502 |
| *Ccdc173* | 2.723161575 | 7.94E-05 | *2810414N06Rik* | 2.060196562 | 0.005088488 | *Gm15809* | 1.890271561 | 0.028334502 |
| *Birc3* | 2.697626483 | 0.000446918 | *Ppox* | 2.060196562 | 0.014076924 | *4930428N03Rik* | 1.890271561 | 0.028334502 |
| *Egr4* | 2.645159063 | 0.000156934 | *Rpia* | 2.060196562 | 0.014076924 | *Bcorl1* | 1.890271561 | 0.028334502 |
| *Fam107b* | 2.645159063 | 0.000645 | *Gm56977* | 2.060196562 | 0.014076924 | *Xlr3b* | 1.890271561 | 0.028334502 |
| *Ints15* | 2.645159063 | 0.004095865 | *Gm29825* | 2.060196562 | 0.014076924 | *Tmem222* | 1.867551484 | 0.000595816 |
| *Get1* | 2.645159063 | 0.004095865 | *Gm30054* | 2.060196562 | 0.014076924 | *Gm56812* | 1.853745685 | 0.006810484 |
| *Lgalsl* | 2.562696903 | 0.000323152 | *Cd9* | 2.060196562 | 0.019163782 | *Zbtb40* | 1.853745685 | 0.012806412 |
| *Dnaaf5* | 2.475234061 | 0.001676117 | *Zfp467* | 2.060196562 | 0.028249505 | *Dusp5* | 1.853745685 | 0.012806412 |
| *Gm47371* | 2.475234061 | 0.001676117 | *Zbed4* | 2.060196562 | 0.028249505 | *Zdhhc7* | 1.837804141 | 0.001645887 |
| *Gm3716* | 2.475234061 | 0.00865482 | *Gm6712* | 2.060196562 | 0.028249505 | *Prkcg* | 1.837804141 | 0.00552553 |
| *Tmem150a* | 2.475234061 | 0.00865482 | *Gpr52* | 2.060196562 | 0.039000569 | *Kcnk10* | 1.819498486 | 2.95E-13 |
| *Susd2* | 2.475234061 | 0.00865482 | *Gm56799* | 2.060196562 | 0.039000569 | *Zbtb24* | 1.797162156 | 0.000265508 |
| *C430042M11Rik* | 2.475234061 | 0.00865482 | *Phtf1os* | 2.060196562 | 0.039000569 | *Lrch2* | 1.797162156 | 0.000476304 |
| *Rhou* | 2.475234061 | 0.018307503 | *Gstm7* | 2.060196562 | 0.039000569 | *Gm3235* | 1.797162156 | 0.008435026 |
| *Spry2* | 2.475234061 | 0.018307503 | *Gm12743* | 2.060196562 | 0.039000569 | *Mocs3* | 1.797162156 | 0.019321216 |
| *Hmcn2* | 2.40123348 | 0.001818703 | *B930046K06Rik* | 2.060196562 | 0.039000569 | *Mob3c* | 1.797162156 | 0.019321216 |
| *1700037H04Rik* | 2.382124657 | 0.000651265 | *Gm10135* | 2.060196562 | 0.039000569 | *Amt* | 1.797162156 | 0.019321216 |
| *H4c8* | 2.382124657 | 0.000651265 | *Pxmp2* | 2.060196562 | 0.039000569 | *Gm17529* | 1.797162156 | 0.019321216 |
| *Gm40893* | 2.382124657 | 0.001293348 | *B230322F03Rik* | 2.060196562 | 0.039000569 | *Bora* | 1.797162156 | 0.019321216 |
| *Gm14617* | 2.349703179 | 0.006930757 | *Pwwp2b* | 2.060196562 | 0.039000569 | *Gm56494* | 1.797162156 | 0.019321216 |
| *Gm14211* | 2.323230968 | 0.000484043 | *Osbpl5* | 2.060196562 | 0.039000569 | *Tnfaip3* | 1.797162156 | 0.036904631 |
| *Tstd2* | 2.282588984 | 9.31E-05 | *Chrna6* | 2.060196562 | 0.039000569 | *1110017D15Rik* | 1.764740679 | 0.000729983 |
| *Cdk5rap1* | 2.282588984 | 0.001299669 | *Gm10629* | 2.060196562 | 0.039000569 | *Tspan9* | 1.755341981 | 0.00131599 |
| *Snx7* | 2.282588984 | 0.002567678 | *Pkd1l2* | 2.060196562 | 0.039000569 | *Icmt* | 1.755341981 | 0.003043172 |

| **Supplemental Table S7B DEGS LAC2 Cluster; VCD/Vehicle.** Maroon=upregulated; | | | | | | | | | |
| --- | --- | --- | --- | --- | --- | --- | --- | --- | --- |
| **gene** | | **avg_log2FC** | **p_val** | **gene** | **avg_log2FC** | **p_val** | **gene** | **avg_log2FC** | **p_val** |
| *Dus4l* | 1.755341981 | | 0.003043172 | *Arid3b* | 1.574769735 | 0.010062416 | *Etaa1* | 1.475234061 | 0.044217789 |
| *Pacc1* | 1.738268467 | | 0.012873786 | *Igsf3* | 1.574769735 | 0.017821482 | *Tha1* | 1.475234061 | 0.044217789 |
| *Ankef1* | 1.738268467 | | 0.012873786 | *Derl3* | 1.562696903 | 0.003101409 | *L3hypdh* | 1.475234061 | 0.044217789 |
| *Brf2* | 1.738268467 | | 0.012873786 | *Edc3* | 1.562696903 | 0.005624325 | *Zfp58* | 1.475234061 | 0.044217789 |
| *Parp16* | 1.738268467 | | 0.012873786 | *4931403E22Rik* | 1.562696903 | 0.006509964 | *1700120C14Rik* | 1.475234061 | 0.044217789 |
| *Ak3* | 1.738268467 | | 0.023916701 | *Ak8* | 1.562696903 | 0.011407175 | *Tmem170* | 1.475234061 | 0.053697347 |
| *Mospd3* | 1.697626483 | | 0.000364813 | *St3gal4* | 1.562696903 | 0.011407175 | *Gyg* | 1.475234061 | 0.054013501 |
| *Aen* | 1.697626483 | | 0.001323016 | *Zbtb2* | 1.562696903 | 0.019726145 | *Csrnp1* | 1.436759914 | 0.00068108 |
| *Chl1* | 1.697626483 | | 0.006881531 | *Zfp524* | 1.553236573 | 0.004212495 | *Acer3* | 1.422766642 | 0.010292349 |
| *Zcchc2* | 1.697626483 | | 0.008488768 | *Zmynd12* | 1.545623389 | 0.004219443 | *Lsmem1* | 1.418650533 | 0.024832773 |
| *Depdc7* | 1.697626483 | | 0.008488768 | *B4galt5* | 1.545623389 | 0.007382282 | *Tigar* | 1.413833517 | 0.005641771 |
| *Rgs19* | 1.697626483 | | 0.039271249 | *Aptx* | 1.539364399 | 0.001763989 | *Ublcp1* | 1.413833517 | 0.005641771 |
| *Mief1* | 1.676867923 | | 0.001528867 | *Acads* | 1.534127751 | 0.000581729 | *Gskip* | 1.413833517 | 0.005641771 |
| *Sin3a* | 1.673173439 | | 2.36E-05 | *Klf4* | 1.50426311 | 7.49E-21 | *Ncdn* | 1.408119866 | 0.005252069 |
| *Wbp1l* | 1.667879139 | | 0.000266246 | *Unc45a* | 1.475234061 | 1.53E-05 | *Gpkow* | 1.408119866 | 0.014581418 |
| *Aldh3b1* | 1.667879139 | | 0.004626416 | *Zswim4* | 1.475234061 | 2.62E-05 | *Jag2* | 1.40123348 | 0.007451964 |
| *Gnptab* | 1.667879139 | | 0.005566986 | *Atad3a* | 1.475234061 | 0.000141229 | *Inpp5e* | 1.40123348 | 0.008182423 |
| *Rel* | 1.667879139 | | 0.009990931 | *Ell2* | 1.475234061 | 0.000215732 | *Cbx8* | 1.40123348 | 0.008182423 |
| *Ppm1n* | 1.667879139 | | 0.017821482 | *4930453N24Rik* | 1.475234061 | 0.000569539 | *Slain1* | 1.40123348 | 0.008182423 |
| *Zfp52* | 1.645159063 | | 0.006456518 | *Mthfr* | 1.475234061 | 0.001474479 | *Slc1a2* | 1.40123348 | 0.013769748 |
| *Dpysl5* | 1.645159063 | | 0.037050051 | *Dhx33* | 1.475234061 | 0.003061676 | *Isyna1* | 1.40123348 | 0.013769748 |
| *Gpat3* | 1.645159063 | | 0.037050051 | *D830024N08Rik* | 1.475234061 | 0.004752474 | *Zbtb5* | 1.392771901 | 0.012776628 |
| *Mia* | 1.645159063 | | 0.037050051 | *Klhdc4* | 1.475234061 | 0.00738544 | *Polr2l* | 1.392771901 | 0.012776628 |
| *Crlf1* | 1.645159063 | | 0.037050051 | *Nprl2* | 1.475234061 | 0.00738544 | *Tmem186* | 1.392771901 | 0.012776628 |
| *Naa80* | 1.645159063 | | 0.037050051 | *Piga* | 1.475234061 | 0.00738544 | *Traf7* | 1.392771901 | 0.012776628 |
| *Gm20470* | 1.645159063 | | 0.037050051 | *Tpst2* | 1.475234061 | 0.008103772 | *Eif3j2* | 1.392771901 | 0.012776628 |
| *Afmid* | 1.645159063 | | 0.037050051 | *Bloc1s6* | 1.475234061 | 0.011497088 | *Ccdc112* | 1.392771901 | 0.012776628 |
| *Actn1* | 1.645159063 | | 0.037050051 | *Ftsj1* | 1.475234061 | 0.011497088 | *Sprtn* | 1.392771901 | 0.021603657 |
| *Gm5087* | 1.645159063 | | 0.037050051 | *Ncln* | 1.475234061 | 0.012668089 | *Dnajc27* | 1.392771901 | 0.021603657 |
| *Pcdh20* | 1.645159063 | | 0.037050051 | *Cd320* | 1.475234061 | 0.012668089 | *Fig4* | 1.392771901 | 0.035977772 |
| *Gm16283* | 1.645159063 | | 0.037050051 | *Pomt1* | 1.475234061 | 0.017942634 | *Nr4a3* | 1.384870034 | 6.76E-14 |
| *Gm30641* | 1.645159063 | | 0.037050051 | *Efcab7* | 1.475234061 | 0.017942634 | *Mapk9* | 1.382124657 | 0.002610017 |
| *Gm13684* | 1.635698734 | | 0.000121391 | *Podxl2* | 1.475234061 | 0.017942634 | *Gm57354* | 1.382124657 | 0.020015635 |
| *Rita1* | 1.627237155 | | 0.002373806 | *Dennd2b* | 1.475234061 | 0.017942634 | *Tnk1* | 1.382124657 | 0.020015635 |
| *Recql5* | 1.627237155 | | 0.004173081 | *Cldn23* | 1.475234061 | 0.017942634 | *Ap5m1* | 1.382124657 | 0.020015635 |
| *Gm26532* | 1.612737585 | | 5.13E-05 | *Gm16845* | 1.475234061 | 0.017942634 | *Hspa12a* | 1.382124657 | 0.020015635 |
| *Ptar1* | 1.612737585 | | 0.004661109 | *Shmt2* | 1.475234061 | 0.017942634 | *Rab30* | 1.37793686 | 0.000503251 |
| *Dph7* | 1.612737585 | | 0.024036295 | *Sptlc1* | 1.475234061 | 0.017942634 | *Map3k4* | 1.375698388 | 0.001517449 |
| *Tmem39b* | 1.612737585 | | 0.024036295 | *Hspe1-rs1* | 1.475234061 | 0.017942634 | *Tmem141* | 1.368318858 | 0.031503026 |
| *Shkbp1* | 1.612737585 | | 0.024036295 | *Mir124a-1hg* | 1.475234061 | 0.019555935 | *Zfp341* | 1.368318858 | 0.031503026 |
| *Slx1b* | 1.612737585 | | 0.024036295 | *Gm17092* | 1.475234061 | 0.028099775 | *Alkbh2* | 1.368318858 | 0.031503026 |
| *Rpf2* | 1.612737585 | | 0.024036295 | *Ndrg4* | 1.475234061 | 0.028099775 | *Slc35b4* | 1.368318858 | 0.031503026 |
| *Mtrr* | 1.612737585 | | 0.024036295 | *Zfp653* | 1.475234061 | 0.028099775 | *Med16* | 1.368318858 | 0.031503026 |
| *Prps2* | 1.612737585 | | 0.024036295 | *Egr2* | 1.475234061 | 0.028099775 | *Ctc1* | 1.368318858 | 0.031503026 |
| *Gm36756* | 1.612737585 | | 0.04401 | *Metrnl* | 1.475234061 | 0.028099775 | *Chrd* | 1.368318858 | 0.031503026 |
| *4932438H23Rik* | 1.612737585 | | 0.04401 | *Rabl2* | 1.475234061 | 0.028099775 | *Garnl3* | 1.368318858 | 0.054013501 |
| *4930503L19Rik* | 1.612737585 | | 0.04401 | *Urb1* | 1.475234061 | 0.028099775 | *Faap24* | 1.368318858 | 0.054013501 |
| *4930455C13Rik* | 1.600764944 | | 0.001005363 | *Suv39h1* | 1.475234061 | 0.028099775 | *Fam83f* | 1.368318858 | 0.054013501 |
| *Mir22hg* | 1.595528295 | | 8.27E-06 | *Tmub1* | 1.475234061 | 0.031301826 | *Tubgcp3* | 1.359756844 | 0.00366698 |
| *Asnsd1* | 1.590711279 | | 0.015555382 | *Nab2* | 1.475234061 | 0.031301826 | *Nfya* | 1.359756844 | 0.00366698 |
| *Lysmd1* | 1.590711279 | | 0.015555382 | *Gm15943* | 1.475234061 | 0.031301826 | *Zfp280c* | 1.349703179 | 0.001094323 |
| *Dnajc16* | 1.590711279 | | 0.015555382 | *Taf9b* | 1.475234061 | 0.031301826 | *Adgra1* | 1.349703179 | 0.005300341 |
| *Gm39214* | 1.590711279 | | 0.015555382 | *Slc30a1* | 1.475234061 | 0.044217789 | *Mtmr4* | 1.349703179 | 0.005707158 |
| *Elp6* | 1.590711279 | | 0.015555382 | *Samd11* | 1.475234061 | 0.044217789 | *Dbr1* | 1.349703179 | 0.009420361 |
| *Rpp40* | 1.590711279 | | 0.015555382 | *Zfp94* | 1.475234061 | 0.044217789 | *Plk2* | 1.349703179 | 0.01536383 |
| *Chrnb3* | 1.590711279 | | 0.027939186 | *Slc19a1* | 1.475234061 | 0.044217789 | *Snapc4* | 1.349703179 | 0.049918754 |
| *Etv5* | 1.574769735 | | 0.00465538 | *Plppr3* | 1.475234061 | 0.044217789 | *Chrnb2* | 1.349703179 | 0.049918754 |

| **Supplemental Table S7C DEGS LAC2 Cluster; VCD/Vehicle.** Maroon=upregulated; | | | | | | | | |
| --- | --- | --- | --- | --- | --- | --- | --- | --- |
| **gene** | **avg_log2FC** | **p_val** | **gene** | **avg_log2FC** | **p_val** | **gene** | **avg_log2FC** | **p_val** |
| *Zfp317* | 1.349703179 | 0.049918754 | *Plrg1* | 1.25284164 | 0.054339709 | *Nudt19* | 1.181502858 | 0.007410512 |
| *Mier2* | 1.349703179 | 0.049918754 | *Ccdc163* | 1.25284164 | 0.054339709 | *Sirt7* | 1.17567378 | 0.004258117 |
| *Tom1l1* | 1.349703179 | 0.049918754 | *Zbtb48* | 1.25284164 | 0.054339709 | *Actr8* | 1.17567378 | 0.00706504 |
| *Grb7* | 1.349703179 | 0.049918754 | *Nat8f1* | 1.25284164 | 0.054339709 | *Tmem242* | 1.17567378 | 0.00706504 |
| *Mxra7* | 1.349703179 | 0.049918754 | *D830035M03Rik* | 1.25284164 | 0.054339709 | *Jmjd8* | 1.17567378 | 0.00706504 |
| *Ranbp6* | 1.349703179 | 0.049918754 | *Osbpl7* | 1.25284164 | 0.054339709 | *Rab26os* | 1.17567378 | 0.01109544 |
| *Zfyve19* | 1.341967531 | 0.001048115 | *Mif4gd* | 1.25284164 | 0.054339709 | *Trank1* | 1.17567378 | 0.031891248 |
| *Sgms2* | 1.341967531 | 0.001702246 | *Hs1bp3* | 1.25284164 | 0.054339709 | *Tctn3* | 1.17567378 | 0.054600784 |
| *Plekhb1* | 1.337730538 | 0.008251791 | *Stn1* | 1.25284164 | 0.054339709 | *Nab1* | 1.171841918 | 9.45E-05 |
| *Gm20186* | 1.337730538 | 0.014715554 | *Rimkla* | 1.246965074 | 1.70E-05 | *Rexo1* | 1.171841918 | 0.001376848 |
| *Pcsk1* | 1.330036146 | 5.82E-05 | *Primpol* | 1.240768808 | 0.023250652 | *Irf2bp2* | 1.171841918 | 0.003169061 |
| *Dhrs11* | 1.323230968 | 0.037432402 | *Dus2* | 1.240768808 | 0.023250652 | *Usp37* | 1.16791038 | 4.87E-08 |
| *Gm7324* | 1.323230968 | 0.037432402 | *Slc25a32* | 1.240768808 | 0.023250652 | *Qtrt1* | 1.167111766 | 0.016352037 |
| *Dbi* | 1.317877734 | 4.29E-13 | *Mpp7* | 1.232776017 | 8.42E-13 | *Mrap2* | 1.167111766 | 0.016352037 |
| *Hyou1* | 1.314769389 | 1.57E-06 | *Baiap2* | 1.230121564 | 0.000154765 | *Slc25a53* | 1.167111766 | 0.016352037 |
| *Ccm2* | 1.314769389 | 0.001153859 | *Ppcdc* | 1.230121564 | 0.004382767 | *Nckap5l* | 1.167111766 | 0.025890629 |
| *Aqr* | 1.314769389 | 0.001845247 | *Itga9* | 1.230121564 | 0.010962351 | *Rnf123* | 1.167111766 | 0.040410874 |
| *Slc35e1* | 1.314769389 | 0.001845247 | *Adgra3* | 1.227306548 | 0.001908714 | *Mtm1* | 1.163290055 | 0.00472927 |
| *Slc66a2* | 1.314769389 | 0.007307804 | *Brinp1* | 1.220304705 | 0.000248771 | *Gga3* | 1.163290055 | 0.007336742 |
| *Kcnk1* | 1.311735329 | 0.002221007 | *Mt3* | 1.212199656 | 1.82E-05 | *Atg4b* | 1.163290055 | 0.011377863 |
| *Obi1* | 1.30530906 | 0.001792157 | *Garin4* | 1.212199656 | 0.00905251 | *Slc25a28* | 1.159732236 | 0.000600748 |
| *Nup107* | 1.30530906 | 0.011677989 | *Ddx25* | 1.212199656 | 0.00905251 | *Sntb1* | 1.153305967 | 3.13E-05 |
| *Zfp146* | 1.30530906 | 0.02177907 | *Tmem183a* | 1.212199656 | 0.010489915 | *Seh1l* | 1.153305967 | 0.000403238 |
| *Nop9* | 1.30530906 | 0.02177907 | *Mfsd5* | 1.212199656 | 0.015675678 | *Gstm1* | 1.153305967 | 0.010925523 |
| *Catspere1* | 1.30530906 | 0.036242522 | *Tcte2* | 1.212199656 | 0.015675678 | *Cmtm4* | 1.153305967 | 0.010925523 |
| *Pls3* | 1.30530906 | 0.036242522 | *Dus3l* | 1.212199656 | 0.015675678 | *Ccdc86* | 1.153305967 | 0.010925523 |
| *Creb3l1* | 1.304175846 | 3.93E-26 | *Gla* | 1.212199656 | 0.015675678 | *Abcf2* | 1.153305967 | 0.017123549 |
| *Uggt1* | 1.300147355 | 1.66E-05 | *Nfs1* | 1.212199656 | 0.016933879 | *Echdc2* | 1.153305967 | 0.038264126 |
| *Rtca* | 1.298356299 | 0.006115801 | *Wdr11* | 1.212199656 | 0.016933879 | *Btbd19* | 1.153305967 | 0.038264126 |
| *Rab5c* | 1.298356299 | 0.009953482 | *Zfp948* | 1.212199656 | 0.017228926 | *Aasdh* | 1.153305967 | 0.038264126 |
| *Atg2a* | 1.298356299 | 0.016009143 | *Tubgcp4* | 1.212199656 | 0.036520149 | *Ino80b* | 1.153305967 | 0.038264126 |
| *Wasf3* | 1.292370004 | 0.00027179 | *Pfdn4* | 1.212199656 | 0.036520149 | *Fam219b* | 1.153305967 | 0.038264126 |
| *Rad54l2* | 1.292370004 | 0.001236001 | *Smarcd3* | 1.212199656 | 0.036520149 | *Gm57027* | 1.153305967 | 0.038264126 |
| *Cep295* | 1.282588984 | 0.002553718 | *Apba3* | 1.212199656 | 0.036520149 | *Mast4* | 1.151806543 | 8.12E-15 |
| *Bmper* | 1.282588984 | 0.00306031 | *Map2k6* | 1.212199656 | 0.036520149 | *Shoc2* | 1.142658722 | 0.000934198 |
| *Scyl2* | 1.282588984 | 0.004678345 | *4930512B01Rik* | 1.212199656 | 0.036520149 | *Fhl1* | 1.142658722 | 0.004474969 |
| *Gm36738* | 1.282588984 | 0.01819353 | *Wdr46* | 1.212199656 | 0.036520149 | *Lasp1* | 1.142658722 | 0.017446245 |
| *Msantd3* | 1.282588984 | 0.03428889 | *Ehd3* | 1.212199656 | 0.036520149 | *Atp8b2* | 1.138199074 | 0.001318249 |
| *Tmem86b* | 1.282588984 | 0.03428889 | *Gnal* | 1.212199656 | 0.036520149 | *Aldh2* | 1.138199074 | 0.011329415 |
| *Ctns* | 1.282588984 | 0.03428889 | *Pop4* | 1.212199656 | 0.044676923 | *Heatr1* | 1.138199074 | 0.015099639 |
| *Bahcc1* | 1.282588984 | 0.03428889 | *Rorb* | 1.208295201 | 5.03E-05 | *Tmem198* | 1.138199074 | 0.02541597 |
| *Slc7a4* | 1.282588984 | 0.03428889 | *Aamp* | 1.205627002 | 3.99E-05 | *Lrrc8b* | 1.138199074 | 0.02541597 |
| *Rrp36* | 1.282588984 | 0.03428889 | *Unc13a* | 1.199599619 | 3.13E-06 | *AI837181* | 1.138199074 | 0.02541597 |
| *Slc6a8* | 1.282588984 | 0.03428889 | *Khsrp* | 1.199599619 | 0.00207519 | *Cfap97d2* | 1.138199074 | 0.040168393 |
| *Homer1* | 1.279010573 | 1.29E-10 | *Cdon* | 1.197700086 | 0.004824398 | *Ntrk3* | 1.133445544 | 2.09E-06 |
| *Map4* | 1.271700667 | 0.000333493 | *Dnajc11* | 1.195126142 | 0.007187281 | *Spidr* | 1.127310758 | 0.003182632 |
| *Ruvbl1* | 1.268783184 | 0.002411046 | *Sclt1* | 1.194605596 | 0.000820645 | *Setd1b* | 1.127310758 | 0.003241873 |
| *Atp5k* | 1.265780696 | 6.82E-06 | *Fdft1* | 1.191441095 | 0.010535221 | *Edc4* | 1.127310758 | 0.016900789 |
| *Gm56506* | 1.263729956 | 0.014860577 | *Clptm1* | 1.188929876 | 0.000354872 | *1500015A07Rik* | 1.127310758 | 0.050443956 |
| *Chp1* | 1.25950537 | 6.56E-05 | *Zswim6* | 1.187108675 | 2.77E-06 | *Tesk1* | 1.121597107 | 0.001362074 |
| *Spag7* | 1.256593775 | 4.06E-05 | *Cbll1* | 1.185727444 | 0.002032245 | *Mrto4* | 1.121597107 | 0.00215717 |
| *Slc6a6* | 1.25284164 | 6.31E-06 | *Pdzk1ip1* | 1.185727444 | 0.024454258 | *Bace1* | 1.121597107 | 0.005011115 |
| *Rpap3* | 1.25284164 | 0.000234348 | *Rrm1* | 1.185727444 | 0.024454258 | *Me1* | 1.121597107 | 0.005011115 |
| *Slc7a6os* | 1.25284164 | 0.001256863 | *Tagap1* | 1.185727444 | 0.024454258 | *Zfp672* | 1.119090251 | 0.006926312 |
| *Zfp180* | 1.25284164 | 0.006477658 | *Ankrd49* | 1.185727444 | 0.039240495 | *Pwp1* | 1.119090251 | 0.011245636 |
| *Smarcal1* | 1.25284164 | 0.016528645 | *Spata2* | 1.181502858 | 0.002977239 | *Pigt* | 1.116780091 | 0.000910612 |
| *Polr1b* | 1.25284164 | 0.028442558 | *Nfxl1* | 1.181502858 | 0.002977239 | *Zfp385b* | 1.115691675 | 0.006307486 |

| **Supplemental Table S7D DEGS LAC2 Cluster; VCD/Vehicle.** Maroon=upregulated; | | | | | | | | |
| --- | --- | --- | --- | --- | --- | --- | --- | --- |
| **gene** | **avg_log2FC** | **p_val** | **gene** | **avg_log2FC** | **p_val** | **gene** | **avg_log2FC** | **p_val** |
| *Ctdspl2* | 1.112663982 | 0.001440407 | *Ifrd2* | 1.060196562 | 0.040543469 | *Cul2* | 0.989807234 | 0.007462385 |
| *Clns1a* | 1.112663982 | 0.001440407 | *Rell2* | 1.060196562 | 0.040543469 | *Dcaf12* | 0.989807234 | 0.01198009 |
| *Ube2j2* | 1.112663982 | 0.002179223 | *5430405H02Rik* | 1.060196562 | 0.041144403 | *Naa16* | 0.989807234 | 0.011989359 |
| *Ykt6* | 1.112663982 | 0.007483768 | *Safb* | 1.049928227 | 0.000110452 | *Ntrk2* | 0.989807234 | 0.013138351 |
| *Pip5k1c* | 1.112663982 | 0.011526628 | *mt-Nd6* | 1.043517821 | 0.001500265 | *Smim1* | 0.989807234 | 0.041572391 |
| *3110082I17Rik* | 1.107502277 | 0.003052146 | *Armc1* | 1.043517821 | 0.00331791 | *Ccdc137* | 0.989807234 | 0.041572391 |
| *Tmem161a* | 1.103265284 | 0.003309909 | *Echs1* | 1.043517821 | 0.00331791 | *Rtl5* | 0.989807234 | 0.001475505 |
| *Matcap1* | 1.103265284 | 0.039585536 | *Sec22a* | 1.040831237 | 0.007635657 | *Spcs3* | 0.989807234 | 0.002158351 |
| *Thop1* | 1.103265284 | 0.039585536 | *Dnajb1* | 1.04001868 | 0.000742117 | *Acsl3* | 0.98744022 | 0.000157115 |
| *Itgav* | 1.099724926 | 7.91E-07 | *Arf6* | 1.039134947 | 0.011690638 | *Trnau1ap* | 0.984247709 | 0.002314768 |
| *Mapkapk5* | 1.099724926 | 0.003336398 | *Cycs* | 1.037112949 | 0.000382472 | *Tapt1* | 0.984247709 | 0.005270731 |
| *Hdgfl2* | 1.099724926 | 0.005039092 | *Wdr41* | 1.037112949 | 0.017555256 | *Setdb1* | 0.984247709 | 0.007820046 |
| *Srrm4* | 1.097532713 | 4.83E-07 | *Hira* | 1.03466147 | 0.000432045 | *Topors* | 0.98219405 | 0.000675163 |
| *Map3k19* | 1.096722438 | 0.026161836 | *Ap1m1* | 1.03466147 | 0.011760766 | *Resf1* | 0.98219405 | 0.018119389 |
| *Kmt5a* | 1.096722438 | 0.026161836 | *2510039O18Rik* | 1.03466147 | 0.011838624 | *Fbf1* | 0.98219405 | 0.018119389 |
| *Bend7* | 1.096722438 | 0.040787738 | *Btbd3* | 1.03466147 | 0.017858904 | *Plekhh2* | 0.98219405 | 0.018128772 |
| *Ppip5k1* | 1.091905422 | 0.004739716 | *Edrf1* | 1.03466147 | 0.017858904 | *Srebf2* | 0.98219405 | 0.026986808 |
| *Kantr* | 1.091905422 | 0.006880294 | *Nomo1* | 1.03466147 | 0.039259706 | *Enc1* | 0.98219405 | 0.039728649 |
| *Trim56* | 1.091905422 | 0.017333389 | *Pold3* | 1.03162741 | 0.011451179 | *Itgb5* | 0.98219405 | 0.039728649 |
| *Znfx1* | 1.091905422 | 0.026792946 | *Cldn9* | 1.03162741 | 0.017894722 | *Prepl* | 0.979706644 | 0.002628948 |
| *Ift46* | 1.088210938 | 0.000944714 | *Tm2d2* | 1.027775085 | 0.000517482 | *Ccdc97* | 0.977734402 | 0.040103739 |
| *Ncstn* | 1.088210938 | 0.011500931 | *Rbm4* | 1.027775085 | 0.001512406 | *Tusc2* | 0.977734402 | 0.005305777 |
| *Fbxw5* | 1.088210938 | 0.017657746 | *Tecpr2* | 1.027775085 | 0.002235471 | *Aifm1* | 0.977734402 | 0.007964199 |
| *Usp35* | 1.088210938 | 0.017657746 | *Lrrc14* | 1.027775085 | 0.041278155 | *Ubap1* | 0.977734402 | 0.017088174 |
| *Pla2g12a* | 1.086668774 | 0.00018211 | *Olfm1* | 1.02624923 | 7.88E-08 | *Dhx15* | 0.975663052 | 0.000933952 |
| *Nmd3* | 1.085287543 | 0.011659299 | *Gm11417* | 1.022721857 | 0.005139216 | *Eed* | 0.972733721 | 0.027454871 |
| *Mfn1* | 1.085287543 | 0.017629512 | *Rab3d* | 1.022721857 | 0.041190436 | *Tfrc* | 0.972733721 | 0.027454871 |
| *Tmem115* | 1.085287543 | 0.017629512 | *Zfp868* | 1.019554578 | 0.005228716 | *Zfp358* | 0.972733721 | 0.02746317 |
| *Wdr13* | 1.080955122 | 0.003365062 | *Ppp5c* | 1.019554578 | 0.011507789 | *Glipr2* | 0.972733721 | 0.041009369 |
| *Cry2* | 1.079825369 | 6.27E-06 | *Mrpl12* | 1.018376387 | 0.000664521 | *Gatad1* | 0.972733721 | 0.001516049 |
| *Dcun1d4* | 1.079305385 | 0.000840019 | *Ankra2* | 1.018376387 | 0.000963891 | *Rsu1* | 0.972733721 | 0.006526928 |
| *Ctps* | 1.077898564 | 0.001458478 | *Cmtm7* | 1.015802443 | 0.003314656 | *Tet3* | 0.971191556 | 0.000991815 |
| *Zbtb4* | 1.076684685 | 0.002210586 | *Zbtb6* | 1.015802443 | 0.038756788 | *Per1* | 0.969998753 | 0.000399211 |
| *Vps37b* | 1.076684685 | 0.003294469 | *Nus1* | 1.012890847 | 0.001516118 | *Bnip3* | 0.969998753 | 0.025858544 |
| *Pard6a* | 1.075626603 | 0.000963718 | *Syap1* | 1.011286962 | 0.011899692 | *Crtc1* | 0.967087158 | 0.002283734 |
| *Ift172* | 1.075626603 | 0.001441413 | *Rbpj* | 1.011286962 | 0.011931433 | *Ndufaf4* | 0.967087158 | 0.003336981 |
| *Rap1b* | 1.073871499 | 0.000933589 | *Maml1* | 1.011286962 | 0.011931433 | *Dhx32* | 0.966220414 | 0.000993512 |
| *Foxj3* | 1.060196562 | 3.47E-05 | *Tuba1b* | 1.011286962 | 0.002014287 | *Dner* | 0.960660889 | 0.000965643 |
| *Cdip1* | 1.060196562 | 0.000414436 | *Edem3* | 1.011286962 | 0.002664107 | *Gramd1a* | 0.960660889 | 0.018128439 |
| *Rnf111* | 1.060196562 | 0.000641125 | *Naa20* | 1.005748778 | 0.000657937 | *Mrps23* | 0.960660889 | 0.018128439 |
| *Mpped2* | 1.060196562 | 0.002714225 | *Ubxn2a* | 1.005748778 | 0.002311173 | *Nudt9* | 0.960660889 | 0.041729206 |
| *Gm16599* | 1.060196562 | 0.003283104 | *Cryba2* | 1.005748778 | 0.011712791 | *Agfg2* | 0.960660889 | 0.041729206 |
| *Ccnh* | 1.060196562 | 0.004917567 | *Pvr* | 1.005748778 | 0.018044356 | *Nfkbib* | 0.960660889 | 0.041729206 |
| *Pcyox1* | 1.060196562 | 0.007438468 | *Dusp16* | 1.005748778 | 0.02709625 | *Alkbh6* | 0.960660889 | 0.041729206 |
| *Decr2* | 1.060196562 | 0.007438468 | *Zfp536* | 1.005013647 | 2.22E-14 | *Nop16* | 0.960660889 | 0.041729206 |
| *Klhl21* | 1.060196562 | 0.007765472 | *Nmt2* | 1.001302873 | 0.016925928 | *Pdia5* | 0.960660889 | 0.002206545 |
| *9530077C05Rik* | 1.060196562 | 0.011507789 | *Atrip* | 0.998796018 | 0.017623544 | *Gstm5* | 0.960660889 | 0.007968195 |
| *Ocel1* | 1.060196562 | 0.011743563 | *Atp2b2* | 0.998796018 | 0.027343274 | *B930095G15Rik* | 0.960660889 | 0.007968195 |
| *Plscr3* | 1.060196562 | 0.016620216 | *Stk10* | 0.998796018 | 0.027343274 | *Wsb2* | 0.960660889 | 0.016861305 |
| *Asphd2* | 1.060196562 | 0.017661107 | *Ppfia1* | 0.997912284 | 1.70E-05 | *Pofut2* | 0.955859902 | 0.001271195 |
| *Nup205* | 1.060196562 | 0.017661107 | *Peli1* | 0.997186765 | 0.000344411 | *Slc9a7* | 0.955227003 | 0.004447202 |
| *Tns3* | 1.060196562 | 0.017768289 | *Neat1* | 0.996931669 | 2.45E-05 | *Ccn1* | 0.954401898 | 5.00E-05 |
| *Zw10* | 1.060196562 | 0.017795179 | *Crkl* | 0.996066225 | 0.011752716 | *Adss* | 0.954401898 | 0.002288472 |
| *Gsr* | 1.060196562 | 0.026716678 | *Cep68* | 0.996066225 | 0.011864276 | *Rb1* | 0.954401898 | 0.003281615 |
| *Obsl1* | 1.060196562 | 0.027015215 | *Mfn2* | 0.994608221 | 0.003366996 | *C9orf72* | 0.954401898 | 0.006928679 |
| *Mtfr1* | 1.060196562 | 0.040543469 | *Rtl8a* | 0.994608221 | 0.005011767 | *Mirg* | 0.953696941 | 1.92E-06 |
| *Slc25a22* | 1.060196562 | 0.040543469 | *Arc* | 0.99202506 | 0.000234561 | *Psat1* | 0.951672105 | 0.011962789 |

| **Supplemental Table S7E DEGS LAC2 Cluster; VCD/Vehicle.** Maroon=upregulated; | | | | | | | | |
| --- | --- | --- | --- | --- | --- | --- | --- | --- |
| **gene** | **avg_log2FC** | **p_val** | **gene** | **avg_log2FC** | **p_val** | **gene** | **avg_log2FC** | **p_val** |
| *Anxa2* | 0.951672105 | 0.012040141 | *Fmn2* | 0.908193469 | 4.62E-05 | *Tmf1* | 0.869513001 | 0.013804994 |
| *Epb41l4a* | 0.951672105 | 0.012277029 | *Cep135* | 0.908193469 | 0.027180295 | *Tmem201* | 0.867551484 | 0.042313557 |
| *Rab3gap2* | 0.94916525 | 0.000191297 | *Dpp3* | 0.908193469 | 0.027180295 | *Ica1* | 0.866921073 | 1.21E-07 |
| *Noc2l* | 0.94916525 | 0.027455178 | *Dnajc18* | 0.905868416 | 0.012218255 | *Slc6a17* | 0.866515438 | 4.76E-07 |
| *Ddx49* | 0.94916525 | 0.027455178 | *Phf6* | 0.90407736 | 0.024499844 | *Iqgap1* | 0.866024015 | 0.031496872 |
| *Rbl2* | 0.94916525 | 0.027455178 | *Nfil3* | 0.902655285 | 0.011019225 | *Wdr25* | 0.862257185 | 0.017484088 |
| *Ndst3* | 0.94916525 | 0.040661734 | *Aff2* | 0.902048729 | 8.00E-05 | *Nploc4* | 0.862257185 | 0.024499844 |
| *Slc7a1* | 0.945863887 | 0.001412501 | *Polr2b* | 0.901498816 | 0.007191644 | *Ralgapb* | 0.862257185 | 0.005561471 |
| *Aida* | 0.944719345 | 0.008027114 | *P2ry14* | 0.900539896 | 0.018168483 | *Grin1* | 0.860524217 | 0.001327479 |
| *Smarcb1* | 0.944719345 | 0.011481575 | *Rangap1* | 0.898445492 | 0.005071523 | *Slc31a1* | 0.859897912 | 0.003370164 |
| *Cxadr* | 0.944719345 | 0.03462901 | *Gtf3c1* | 0.897467062 | 0.001529086 | *Cdc5l* | 0.859897912 | 0.004939749 |
| *Fbxl16* | 0.943382897 | 8.37E-05 | *Fem1b* | 0.897467062 | 0.008057822 | *Tardbp* | 0.859377553 | 0.00112991 |
| *Rcc2* | 0.943382897 | 0.000123871 | *Arl5b* | 0.89637176 | 0.00044579 | *Elp5* | 0.858562701 | 0.026256839 |
| *Tlcd4* | 0.940196786 | 1.40E-05 | *Tsix* | 0.890271561 | 0.001016532 | *Flnb* | 0.858562701 | 0.027148308 |
| *Naa15* | 0.939181161 | 0.000139827 | *Lsm7* | 0.890271561 | 0.001492748 | *Cyb561* | 0.858562701 | 0.03758282 |
| *Wdr37* | 0.939181161 | 0.002399249 | *Ccng1* | 0.890271561 | 0.007590555 | *Ncald* | 0.857703698 | 0.012697099 |
| *Tmco3* | 0.939181161 | 0.007650212 | *Gm57212* | 0.890271561 | 0.011603378 | *Atp6v0a2* | 0.857104697 | 0.010495123 |
| *Mapk8* | 0.938206038 | 0.006423262 | *Smim22* | 0.890271561 | 0.026930198 | *C1qtnf4* | 0.857104697 | 0.014356537 |
| *Fntb* | 0.93466568 | 0.003950153 | *1110059G10Rik* | 0.890271561 | 0.027274604 | *Fam131a* | 0.853745685 | 0.004364214 |
| *Klf14* | 0.93466568 | 0.007191644 | *Ankmy2* | 0.890271561 | 0.027887735 | *Dnlz* | 0.853745685 | 0.027801465 |
| *Gls* | 0.93466568 | 0.010314433 | *Lrrc8d* | 0.890271561 | 0.039019811 | *Fbxo21* | 0.853745685 | 0.040186695 |
| *Lmf1* | 0.93466568 | 0.027519839 | *Dgke* | 0.890271561 | 0.039019811 | *Upf1* | 0.853745685 | 0.040186695 |
| *Slc2a13* | 0.932788393 | 2.00E-21 | *Shb* | 0.890271561 | 0.040273915 | *Rabgef1* | 0.853745685 | 0.000898451 |
| *mt-Atp8* | 0.932091736 | 0.000352827 | *Tjp1* | 0.890271561 | 0.041169814 | *Sgcz* | 0.853313805 | 1.99E-05 |
| *Spaca6* | 0.929799925 | 0.047821946 | *B3gntl1* | 0.890271561 | 0.041169814 | *Prkca* | 0.851100963 | 0.001555137 |
| *Srxn1* | 0.929265692 | 0.000151666 | *Gm14216* | 0.890271561 | 0.050786613 | *Dync1li1* | 0.850743197 | 0.009405698 |
| *Cep120* | 0.928599679 | 0.0001663 | *Ap2a2* | 0.890271561 | 0.00084469 | *Rcor1* | 0.850743197 | 0.013083749 |
| *Mycbp* | 0.927746266 | 0.027359214 | *Galnt2* | 0.890271561 | 0.005045051 | *Mad1l1* | 0.849629576 | 0.023997252 |
| *Tmem259* | 0.927746266 | 0.027359214 | *Rfc2* | 0.890271561 | 0.007955916 | *Gm40477* | 0.849629576 | 0.035740676 |
| *Nfyb* | 0.927746266 | 0.027359214 | *Arl6* | 0.890271561 | 0.016963062 | *Slc12a2* | 0.849629576 | 0.050709409 |
| *Ccl27a* | 0.927746266 | 0.040202205 | *Nelfa* | 0.890271561 | 0.025428107 | *Vps39* | 0.847202839 | 0.042288803 |
| *Ak6* | 0.927746266 | 0.040202205 | *Gm32014* | 0.890271561 | 0.026464403 | *Smarcd1* | 0.847202839 | 0.042288803 |
| *Map3k12* | 0.927746266 | 0.040202205 | *Fosb* | 0.887881013 | 8.50E-26 | *1600020E01Rik* | 0.845602511 | 0.00215408 |
| *H2-K1* | 0.927746266 | 0.041361343 | *Lrrc59* | 0.884624998 | 0.000125229 | *Blzf1* | 0.845602511 | 0.008005986 |
| *Jak2* | 0.925895471 | 0.005203741 | *Uhrf1bp1l* | 0.881859321 | 0.001374571 | *Pdcd7* | 0.844467871 | 0.025850831 |
| *Avl9* | 0.925895471 | 0.010641077 | *Ddr1* | 0.881226421 | 0.011107592 | *Hsph1* | 0.843385173 | 0.003084588 |
| *Rtl1* | 0.925399784 | 5.15E-05 | *Mical2* | 0.879624317 | 0.009804955 | *Zdbf2* | 0.843115495 | 3.19E-06 |
| *Ywhab* | 0.922693038 | 7.45E-05 | *Cyb5r3* | 0.878589756 | 0.003686329 | *Uqcc1* | 0.842965846 | 0.022476617 |
| *Lamc1* | 0.922693038 | 0.037829822 | *Mrpl11* | 0.878589756 | 0.005259134 | *Tro* | 0.842016392 | 0.004650927 |
| *Dot1l* | 0.920018904 | 0.003226157 | *Coq7* | 0.878589756 | 0.01505456 | *Chtop* | 0.842016392 | 0.012302513 |
| *Snapc1* | 0.917752297 | 0.001456958 | *Wdr47* | 0.877993231 | 0.001129257 | *Abhd17a* | 0.837804141 | 0.002967684 |
| *Dbpht2* | 0.916743772 | 8.12E-05 | *Gm10475* | 0.877332505 | 0.016685245 | *Ndufaf8* | 0.837804141 | 0.006988006 |
| *Rfx2* | 0.915806653 | 0.000904335 | *Fbl* | 0.876596624 | 0.000720832 | *Mllt11* | 0.837804141 | 0.011057233 |
| *Sbds* | 0.915806653 | 0.003637038 | *Cand1* | 0.875771991 | 0.002169213 | *Gstcd* | 0.837804141 | 0.016957735 |
| *Sap130* | 0.915806653 | 0.005243271 | *Itgb1bp1* | 0.875771991 | 0.017706186 | *Atn1* | 0.837804141 | 0.016957735 |
| *Snx15* | 0.915806653 | 0.007858042 | *Atp5md* | 0.875413602 | 6.24E-07 | *Sp1* | 0.837804141 | 0.016957735 |
| *Mapkapk2* | 0.915806653 | 0.007969933 | *Bin1* | 0.873783438 | 0.005560893 | *Coq8b* | 0.837804141 | 0.019505499 |
| *Enpp5* | 0.915806653 | 0.011190017 | *Arhgef18* | 0.873783438 | 0.026619323 | *Nrp1* | 0.837804141 | 0.023475635 |
| *Las1l* | 0.915806653 | 0.011190017 | *Pqbp1* | 0.873783438 | 0.026619323 | *Epn1* | 0.837804141 | 0.039569192 |
| *Taf1d* | 0.915806653 | 0.023102534 | *Ankrd37* | 0.873783438 | 0.038326931 | *Crb3* | 0.837804141 | 0.039569192 |
| *Stau1* | 0.913355174 | 0.005095903 | *Megf8* | 0.873783438 | 0.054574118 | *Bzw2* | 0.835409626 | 7.78E-05 |
| *Atxn2l* | 0.913355174 | 0.005159373 | *Cmip* | 0.873495848 | 2.58E-06 | *Thoc2l* | 0.835130007 | 0.002960473 |
| *Entpd3* | 0.912297867 | 2.24E-05 | *Ewsr1* | 0.872993569 | 0.001028514 | *Cadps2* | 0.834460698 | 2.62E-09 |
| *Slc30a4* | 0.911333176 | 0.041500344 | *Vwa5a* | 0.871162738 | 0.028033422 | *Clstn3* | 0.834315155 | 0.001335794 |
| *Snapin* | 0.911333176 | 0.041500344 | *Adrm1* | 0.871162738 | 0.040725645 | *Septin9* | 0.833688032 | 4.43E-05 |
| *1700029J07Rik* | 0.911333176 | 0.041500344 | *Dynlt3* | 0.869513001 | 0.004821987 | *Tomm7* | 0.833281582 | 0.000269302 |
| *Nufip1* | 0.911333176 | 0.042074817 | *Actl6b* | 0.869513001 | 0.004902782 | *Bzw1* | 0.832786066 | 0.007642717 |

| **Supplemental Table S7F DEGS LAC2 Cluster; VCD/Vehicle.** Maroon=upregulated; | | | | | | | | |
| --- | --- | --- | --- | --- | --- | --- | --- | --- |
| **gene** | **avg_log2FC** | **p_val** | **gene** | **avg_log2FC** | **p_val** | **gene** | **avg_log2FC** | **p_val** |
| *Sin3b* | 0.832786066 | 0.010682882 | *Gna11* | 0.80047489 | 0.000227326 | *Mvb12b* | 0.770689945 | 0.014707973 |
| *Lclat1* | 0.832786066 | 0.010901262 | *Syt4* | 0.797650295 | 2.08E-11 | *Atp6ap1* | 0.76968142 | 0.004358391 |
| *Anxa4* | 0.832786066 | 0.015671027 | *Ripk4* | 0.797162156 | 0.041332958 | *Utp14a* | 0.768965265 | 0.022292115 |
| *Gpat4* | 0.832786066 | 0.054367378 | *Bdp1* | 0.797162156 | 0.000287028 | *Rhot1* | 0.768965265 | 0.031761284 |
| *Hdac5* | 0.831377872 | 0.002231057 | *Mgrn1* | 0.797162156 | 0.013977085 | *Cblb* | 0.767684955 | 0.007801363 |
| *Fam118a* | 0.831377872 | 0.018733979 | *Snrpa1* | 0.797162156 | 0.015680394 | *Zcchc7* | 0.765362128 | 9.21E-08 |
| *Dlat* | 0.831377872 | 0.025408416 | *Rab8a* | 0.797162156 | 0.021275747 | *Tmem126a* | 0.764740679 | 0.003100514 |
| *Zcchc9* | 0.831377872 | 0.025917181 | *2810004N23Rik* | 0.797162156 | 0.034406733 | *Snhg3* | 0.764740679 | 0.013890848 |
| *Rab18* | 0.83032902 | 0.005853911 | *Iws1* | 0.797162156 | 0.034406733 | *Trim39* | 0.764740679 | 0.025369977 |
| *Jun* | 0.829008265 | 7.84E-08 | *Cmtr1* | 0.797162156 | 0.037375654 | *Taf7* | 0.764740679 | 0.025964952 |
| *Necap1* | 0.828871016 | 0.025029991 | *Sgk1* | 0.797162156 | 0.038238211 | *Gpbp1l1* | 0.764740679 | 0.026904197 |
| *Zfp516* | 0.82721939 | 0.000118469 | *Plppr2* | 0.797162156 | 0.052542512 | *Hdac9* | 0.764740679 | 0.04121996 |
| *Cers6* | 0.825731309 | 1.22E-05 | *Rpp14* | 0.797162156 | 0.052542512 | *Dusp10* | 0.764740679 | 0.028418259 |
| *Isg20* | 0.823157365 | 0.003458113 | *Gxylt1* | 0.797162156 | 0.05436739 | *Unc5c* | 0.76347903 | 0.039776172 |
| *Dmpk* | 0.823157365 | 0.018803578 | *Tnpo1* | 0.795368875 | 8.80E-06 | *Sorbs1* | 0.763214824 | 5.81E-06 |
| *Ascc2* | 0.823157365 | 0.027375846 | *Slc23a2* | 0.793595541 | 0.002174893 | *Gm12248* | 0.76063628 | 0.022969449 |
| *Tfcp2* | 0.823157365 | 0.038887105 | *Syn3* | 0.790735887 | 0.01421194 | *Gigyf1* | 0.76063628 | 0.029886739 |
| *Rbfa* | 0.823157365 | 0.039673274 | *Arfip1* | 0.790107399 | 0.022391251 | *Tpm1* | 0.76063628 | 0.029886739 |
| *Naa38* | 0.820369548 | 0.006705393 | *Elp1* | 0.789342652 | 0.007181277 | *Cab39l* | 0.76063628 | 0.040838266 |
| *Nek1* | 0.820369548 | 0.013345717 | *Rab5if* | 0.789342652 | 0.013612255 | *Cpeb4* | 0.76011517 | 8.44E-05 |
| *Tmem258* | 0.819568569 | 1.15E-05 | *Fermt2* | 0.789342652 | 0.005503882 | *Eif1a* | 0.759963538 | 0.007889474 |
| *Ube2q1* | 0.819188463 | 0.003202439 | *Map1a* | 0.788391947 | 0.015331579 | *Smg7* | 0.759027027 | 0.010183948 |
| *Plekhm2* | 0.819188463 | 0.024935859 | *Zer1* | 0.788391947 | 0.020712701 | *Meaf6* | 0.759027027 | 0.05468381 |
| *Exosc10* | 0.819188463 | 0.024935859 | *Chka* | 0.787701827 | 0.000242698 | *Tmem60* | 0.759027027 | 0.05468381 |
| *Otud7b* | 0.819188463 | 0.035139006 | *Iqsec3* | 0.787178068 | 0.002785865 | *Fam3c* | 0.759027027 | 0.05468381 |
| *Slc22a17* | 0.818121775 | 0.015984395 | *Cep41* | 0.787178068 | 0.044889972 | *Cmtm8* | 0.759027027 | 0.05468381 |
| *Pam* | 0.817626259 | 1.90E-13 | *Herpud1* | 0.786820212 | 0.003198424 | *Mast2* | 0.758168025 | 0.010672201 |
| *Zfp706* | 0.817340038 | 0.00091019 | *Clint1* | 0.786163157 | 0.000465043 | *Nbas* | 0.757821265 | 9.12E-07 |
| *Cdk13* | 0.817153632 | 7.23E-05 | *D630045J12Rik* | 0.785574182 | 0.036565607 | *Ap1s1* | 0.757633792 | 0.013528284 |
| *Tmx1* | 0.814720528 | 0.001294088 | *Adam10* | 0.785574182 | 0.036565607 | *Prkar2a* | 0.755341981 | 0.002053789 |
| *Ifrd1* | 0.813402797 | 5.55E-05 | *Pdzd8* | 0.785574182 | 0.051153613 | *Cse1l* | 0.755341981 | 0.02486783 |
| *Golt1b* | 0.813402797 | 0.002798933 | *Ankrd13c* | 0.78456212 | 0.002870576 | *Ythdf2* | 0.755341981 | 0.034008037 |
| *Acp1* | 0.813402797 | 0.002991847 | *Ncbp1* | 0.783356357 | 0.011549934 | *Nars2* | 0.755341981 | 0.034008037 |
| *Sel1l3* | 0.813402797 | 0.010974497 | *Fbxw2* | 0.7818954 | 0.003548354 | *Rtn4* | 0.754388133 | 0.00012731 |
| *Gm26658* | 0.813402797 | 0.025824887 | *Tex261* | 0.7818954 | 0.009337028 | *Marchf8* | 0.75395009 | 0.002493419 |
| *Ing1* | 0.812269049 | 0.017841639 | *Gm17056* | 0.780088643 | 0.026729638 | *Dicer1* | 0.753535224 | 0.001530647 |
| *Sik2* | 0.811597247 | 2.20E-07 | *Nap1l3* | 0.777796832 | 0.016627441 | *Opa3* | 0.752768037 | 0.021745445 |
| *Coro1c* | 0.811283265 | 0.001176608 | *Xpnpep1* | 0.776889761 | 0.006326039 | *Txnl4a* | 0.752343653 | 9.84E-05 |
| *Ide* | 0.810837093 | 0.008233952 | *Rgs17* | 0.776889761 | 0.011605989 | *Sec13* | 0.751751697 | 0.001549335 |
| *Ythdc1* | 0.810837093 | 4.22E-06 | *B230216N24Rik* | 0.776403596 | 0.001357603 | *Tubb2a* | 0.751751697 | 0.002825718 |
| *Zc3h12c* | 0.810582672 | 0.0003961 | *Hnrnpul2* | 0.776100541 | 0.000657578 | *Adcy6* | 0.750868504 | 0.009803253 |
| *Atg4a* | .810418253 | 0.00173218 | *Arih1* | 0.775929519 | 1.33E-07 | *Tiam1* | 0.750241109 | 0.004421504 |
| *Picalm* | 0.810418253 | 0.00797822 | *Tsc1* | 0.774794343 | 0.009325645 | *Tmem216* | 0.748252556 | 0.027564511 |
| *Igf2r* | 0.809653101 | 0.010317195 | *Erlin2* | 0.774794343 | 0.03573004 | *Wdr74* | 0.748252556 | 0.038753627 |
| *Nfkbil1* | 0.809653101 | 0.038152468 | *Wdr36* | 0.774794343 | 0.049755767 | *Ddx27* | 0.748252556 | 0.039646377 |
| *Rap2a* | 0.809653101 | 0.053914038 | *Or4e5* | 0.774794343 | 0.052863527 | *Gatb* | 0.748252556 | 0.054778107 |
| *Gnl3l* | 0.808657795 | 0.001771523 | *Atf3* | 0.774794343 | 1.42E-07 | *Map7d1* | 0.748252556 | 0.054778107 |
| *Plagl1* | 0.808200428 | 7.37E-17 | *Zzz3* | 0.77291461 | 0.007561534 | *Mrpl52* | 0.748252556 | 0.024598706 |
| *Tmem120b* | 0.807809401 | 0.049211726 | *Etv6* | 0.77291461 | 0.01376808 | *Irf2* | 0.746536083 | 0.045573922 |
| *Hspa5* | 0.806567304 | 3.40E-09 | *Pde10a* | 0.772264039 | 1.55E-20 | *1700016P03Rik* | 0.745827258 | 6.21E-08 |
| *Fhip1b* | 0.805382663 | 0.007271466 | *Ttc7b* | 0.772215799 | 0.014252633 | *Ythdf1* | 0.745323225 | 0.015255548 |
| *Plekhj1* | 0.804541687 | 0.012200633 | *Timm9* | 0.772215799 | 0.015487248 | *Hlcs* | 0.745323225 | 0.015255548 |
| *Neurl1a* | 0.804541687 | 0.016692615 | *Rps27* | 0.771743172 | 2.83E-12 | *Actr1b* | 0.745323225 | 0.020609667 |
| *Washc3* | 0.804541687 | 0.017636839 | *Ddah1* | 0.771627064 | 0.00386201 | *Uba1* | 0.743722897 | 0.034126492 |
| *Ccdc91* | 0.804541687 | 0.018609095 | *Ctxn3* | 0.770689945 | 0.006301141 | *Srsf7* | 0.740578628 | 0.000172628 |
| *Romo1* | 0.801734886 | 0.000422919 | *B3gnt2* | 0.770689945 | 0.011493852 | *Sec24a* | 0.740206272 | 0.001023245 |
| *Ppa1* | 0.800809933 | 0.000438541 | *Gna11* | 0.80047489 | 0.000227326 | *Eif4e* | 0.740206272 | 0.00588875 |

| **Supplemental Table S7G DEGS LAC2 Cluster; VCD/Vehicle.** Maroon=upregulated; | | | | | | | | |
| --- | --- | --- | --- | --- | --- | --- | --- | --- |
| **gene** | **avg_log2FC** | **p_val** | **gene** | **avg_log2FC** | **p_val** | **gene** | **avg_log2FC** | **p_val** |
| *Dpy30* | 0.738268467 | 0.006886589 | *Aggf1* | 0.716242161 | 0.030503997 | *Ttc39c* | 0.690246952 | 0.041659485 |
| *Wdr45b* | 0.738268467 | 0.009628393 | *Pwwp3b* | 0.716242161 | 0.030909912 | *Dhdds* | 0.689358867 | 0.02755627 |
| *Eps15l1* | 0.738268467 | 0.013997952 | *Hsd17b8* | 0.716242161 | 0.054168135 | *Pank2* | 0.689358867 | 0.05411079 |
| *B4galt6* | 0.738268467 | 0.023974396 | *Pigbos1* | 0.714020921 | 0.004992232 | *2310010J17Rik* | 0.689358867 | 0.05411079 |
| *Ganab* | 0.738268467 | 0.024453103 | *Cep170* | 0.714020921 | 0.012904721 | *Cacfd1* | 0.688227785 | 0.015701096 |
| *Snrnp40* | 0.738268467 | 0.032252579 | *Pdap1* | 0.713393799 | 0.00101111 | *Ndufb6* | 0.686738167 | 0.002589534 |
| *Heatr3* | 0.738268467 | 0.033165235 | *N4bp2l2* | 0.713393799 | 0.003393317 | *Hmgn5* | 0.686738167 | 0.025418087 |
| *Ptch1* | 0.738268467 | 0.04420064 | *Npat* | 0.712273259 | 0.047756516 | *Tfg* | 0.686738167 | 0.005584037 |
| *Sec23b* | 0.738268467 | 0.002077297 | *Spag9* | 0.711610712 | 5.21E-07 | *Ddx3x* | 0.686001158 | 0.004922583 |
| *Qser1* | 0.738268467 | 0.015883641 | *Zfp106* | 0.710612124 | 0.006456052 | *Lncpint* | 0.68549052 | 1.64E-10 |
| *St3gal5* | 0.738268467 | 0.051937596 | *Zfhx2* | 0.709699315 | 0.002802383 | *Zhx3* | 0.685451769 | 0.013082205 |
| *Bag6* | 0.738268467 | 0.051937596 | *Api5* | 0.709699315 | 0.00918153 | *Sipa1l2* | 0.685157131 | 0.001362881 |
| *Degs1* | 0.738268467 | 0.05340318 | *Tmem256* | 0.709699315 | 0.028760134 | *Pacrg* | 0.684687427 | 0.000492064 |
| *Cited1* | 0.738268467 | 0.054946656 | *Gid4* | 0.709699315 | 0.038900922 | *Mapk1ip1l* | 0.684687427 | 0.045150229 |
| *Gng4* | 0.735623746 | 0.000490225 | *Brix1* | 0.709699315 | 0.040307431 | *Tnik* | 0.684180952 | 0.011229018 |
| *Casz1* | 0.735469834 | 0.001260424 | *Mdn1* | 0.707894818 | 0.023234661 | *Brd3* | 0.683820683 | 0.006649193 |
| *Ppm1h* | 0.735469834 | 0.005219476 | *Rpl36* | 0.707630935 | 6.80E-23 | *Gtf2f2* | 0.682559035 | 0.000976084 |
| *Arhgef7* | 0.734321283 | 0.013158573 | *Hivep1* | 0.707407504 | 0.009729494 | *Rps27l* | 0.681684939 | 0.000920134 |
| *Larp4* | 0.733546057 | 0.009427875 | *Col27a1* | 0.706559608 | 0.008424779 | *Dnajc5* | 0.681684939 | 0.009101086 |
| *Rps29* | 0.732819923 | 1.08E-20 | *Cfap61* | 0.706559608 | 0.012381927 | *Kif18a* | 0.681684939 | 0.02057895 |
| *Wdr43* | 0.732391901 | 0.009337435 | *Hpf1* | 0.706559608 | 0.019225157 | *Plekhm3* | 0.681684939 | 0.034097693 |
| *G6pdx* | 0.732391901 | 0.012091503 | *Arntl* | 0.70584699 | 0.011166926 | *Ppm1b* | 0.681684939 | 0.043662114 |
| *Mtln* | 0.732391901 | 0.016323924 | *Hnrnpl* | 0.704715908 | 0.002784072 | *Eloa* | 0.681684939 | 0.054275379 |
| *Tssc4* | 0.732391901 | 0.022017353 | *Ilk* | 0.704715908 | 0.047796833 | *Nrep* | 0.680053853 | 0.009556506 |
| *Camsap1* | 0.732391901 | 0.040638631 | *Fmc1* | 0.703503049 | 0.028753248 | *Add1* | 0.680053853 | 0.011060827 |
| *Gps1* | 0.731573815 | 0.027394227 | *Xpot* | 0.703503049 | 0.029729454 | *Gosr2* | 0.67959256 | 0.009204301 |
| *Dstyk* | 0.731573815 | 0.037092792 | *Gna13* | 0.703503049 | 0.050466425 | *Zmiz1* | 0.678767456 | 0.00499739 |
| *Pgbd5* | 0.731072966 | 0.004468782 | *Pak1* | 0.702644558 | 0.010137398 | *Zfp407* | 0.678767456 | 0.015516178 |
| *Smchd1* | 0.730491117 | 0.000654735 | *Nsmce1* | 0.702644558 | 0.017271567 | *Siah2* | 0.676867923 | 0.002230936 |
| *Ebag9* | 0.730491117 | 0.023255313 | *Lsm12* | 0.701509917 | 0.008244758 | *Abhd16a* | 0.676867923 | 0.01485208 |
| *Tubgcp5* | 0.730491117 | 0.023911004 | *Mir670hg* | 0.699449218 | 0.002480529 | *Smim30* | 0.675931412 | 0.00232168 |
| *9330159F19Rik* | 0.730491117 | 0.023911004 | *Kdm4c* | 0.697626483 | 0.000495756 | *Kpna1* | 0.675931412 | 0.014615514 |
| *Depdc5* | 0.730491117 | 0.032277224 | *Usp47* | 0.697626483 | 0.000937281 | *Atp6v1e1* | 0.675532712 | 0.024129345 |
| *Ptprn* | 0.728990654 | 1.23E-07 | *U2af1* | 0.697626483 | 0.004800855 | *Atp2b1* | 0.675408443 | 2.19E-06 |
| *Nol8* | 0.728990654 | 0.050544912 | *Frs2* | 0.697626483 | 0.010382843 | *Morc3* | 0.67454287 | 0.05131556 |
| *Capn7* | 0.728990654 | 0.052009701 | *Rhoq* | 0.697626483 | 0.016111898 | *Rpl37* | 0.673313025 | 2.04E-20 |
| *Atad2b* | 0.728990654 | 0.008654571 | *Yrdc* | 0.697626483 | 0.016260665 | *Tubb4b* | 0.673173439 | 0.000251481 |
| *Htt* | 0.728352998 | 0.004274679 | *Pin1* | 0.697626483 | 0.036396107 | *Foxo1* | 0.673173439 | 0.034411526 |
| *Tfdp1* | 0.728000132 | 0.005834837 | *Fndc4* | 0.697626483 | 0.044999648 | *Rpl39* | 0.672624851 | 6.10E-16 |
| *Sptbn4* | 0.728000132 | 0.01854393 | *Arhgef5* | 0.697626483 | 0.046402063 | *Gdap1l1* | 0.672270909 | 0.017629718 |
| *Fgfr2* | 0.728000132 | 0.030348135 | *Fahd1* | 0.697626483 | 0.046402063 | *Zswim8* | 0.672270909 | 0.018250427 |
| *Uggt2* | 0.726772828 | 0.006575515 | *Man2a1* | 0.697626483 | 0.05143045 | *Snrpg* | 0.671925972 | 0.000921118 |
| *Tiparp* | 0.723810415 | 0.000152365 | *Irs2* | 0.696967868 | 1.23E-06 | *Rnps1* | 0.671631274 | 0.014278096 |
| *Gm37982* | 0.723161575 | 0.01501635 | *ENSMUSG00000121513* | 0.695500182 | 0.000498178 | *Zcchc12* | 0.671631274 | 0.014278096 |
| *Atp13a3* | 0.723161575 | 0.043630562 | *Stmp1* | 0.69454709 | 0.005191474 | *Trappc9* | 0.669737086 | 0.004017765 |
| *Brd2* | 0.722326923 | 0.000232478 | *Uqcr11* | 0.694351351 | 1.56E-06 | *Rpl41* | 0.669244681 | 4.41E-42 |
| *Dnttip1* | 0.721394649 | 0.015300437 | *Chrac1* | 0.693414232 | 0.012480179 | *Cdkl4* | 0.667879139 | 0.013250483 |
| *Arid2* | 0.720346559 | 0.009906073 | *Cops7a* | 0.692825497 | 0.01621526 | *Pik3r1* | 0.667879139 | 0.014937006 |
| *Vps4a* | 0.720346559 | 0.018720933 | *Ppm1g* | 0.692825497 | 0.020989928 | *Kazald1* | 0.667879139 | 0.036981637 |
| *Hax1* | 0.720346559 | 0.050606213 | *Tbce* | 0.692045451 | 0.02050423 | *Lemd3* | 0.667879139 | 0.037968421 |
| *Ccnl1* | 0.719159644 | 0.003539177 | *Tnrc18* | 0.692045451 | 0.036499565 | *Rsbn1* | 0.667879139 | 0.029263181 |
| *Lmo7* | 0.718791093 | 0.003388066 | *Ndufc1* | 0.691551968 | 0.002858974 | *Ctnnbl1* | 0.667879139 | 0.039678825 |
| *Rad23a* | 0.718505213 | 0.006995976 | *Rpl35a* | 0.690962753 | 1.83E-21 | *Scpep1* | 0.667879139 | 0.053269564 |
| *Polr2j* | 0.717804365 | 0.002112327 | *Pip4p2* | 0.690962753 | 0.043643251 | *Txndc11* | 0.666599587 | 0.008489359 |
| *Alg13* | 0.717804365 | 0.002112327 | *Plaa* | 0.690962753 | 0.043643251 | *Bbln* | 0.66505862 | 0.00435412 |
| *Ugp2* | 0.716242161 | 0.027460771 | *Atxn7* | 0.690246952 | 0.006334472 | *Nsf* | 0.66471186 | 3.46E-05 |
| *Stx18* | 0.716242161 | 0.004151491 | *Cep350* | 0.690246952 | 0.01093961 | *Ptpro* | 0.664406948 | 5.27E-06 |

| **Supplemental Table S7H DEGS LAC2 Cluster; VCD/Vehicle.** Maroon=upregulated; | | | | | | | | |
| --- | --- | --- | --- | --- | --- | --- | --- | --- |
| **gene** | **avg_log2FC** | **p_val** | **gene** | **avg_log2FC** | **p_val** | **gene** | **avg_log2FC** | **p_val** |
| *Rps26* | 0.663973059 | 4.06E-15 | *Acap2* | 0.633496145 | 0.009223524 | *Ptbp2* | 0.606478595 | 0.00542693 |
| *Mark3* | 0.662861065 | 0.005092703 | *Commd10* | 0.632775338 | 0.05411079 | *Tmpo* | 0.606478595 | 0.012477706 |
| *Ptpa* | 0.662861065 | 0.038401676 | *Mrpl23* | 0.631353263 | 0.003615543 | *Sos1* | 0.606478595 | 0.030551523 |
| *Skp1* | 0.661647186 | 0.001376409 | *Stam2* | 0.631353263 | 0.040355019 | *Med15* | 0.606478595 | 0.043861006 |
| *Nlk* | 0.661647186 | 0.003695986 | *Arhgef17* | 0.631353263 | 0.054142285 | *Ero1b* | 0.606478595 | 0.050927748 |
| *Stox2* | 0.660151777 | 0.005356304 | *Plpp5* | 0.630512287 | 0.029188601 | *Nsg1* | 0.605294602 | 0.021870302 |
| *Pianp* | 0.659658633 | 0.005335306 | *Arfgef2* | 0.629956656 | 0.042484348 | *5730522E02Rik* | 0.605294602 | 0.024641453 |
| *Dnmt1* | 0.659658633 | 0.028602626 | *Cisd2* | 0.62926769 | 0.016433813 | *Dazap1* | 0.603558158 | 0.040692715 |
| *Cbarp* | 0.659098254 | 0.003367037 | *Timm8b* | 0.627237155 | 0.012622568 | *Rpl9-ps6* | 0.603558158 | 0.040692715 |
| *Snhg18* | 0.658098119 | 0.000736976 | *Slmap* | 0.627237155 | 0.014132851 | *Ginm1* | 0.603558158 | 0.042298014 |
| *Elof1* | 0.658098119 | 0.00287487 | *Zdhhc1* | 0.627237155 | 0.019752302 | *Rpl35* | 0.603390413 | 7.60E-05 |
| *Oat* | 0.658098119 | 0.029469033 | *Prrt1* | 0.627237155 | 0.021234552 | *Dgkh* | 0.603241674 | 0.036073409 |
| *Polr2h* | 0.658098119 | 0.038510461 | *Pgm3* | 0.627237155 | 0.028167443 | *Atp6v0a1* | 0.602613368 | 0.02572239 |
| *Mrps36* | 0.658098119 | 0.047789008 | *Tmem14c* | 0.627237155 | 0.046437217 | *Cplx2* | 0.602613368 | 0.028090621 |
| *Reep3* | 0.657437393 | 0.003128592 | *Cox20* | 0.627237155 | 0.052289885 | *Sec61g* | 0.600764944 | 4.52E-07 |
| *Bola3* | 0.657437393 | 0.009331909 | *A230051N06Rik* | 0.627237155 | 0.052289885 | *Shank1* | 0.600764944 | 0.051225295 |
| *Pgp* | 0.657437393 | 0.011969456 | *Fxr1* | 0.624981181 | 0.03310124 | *Strap* | 0.600764944 | 0.006095822 |
| *Mrpl34* | 0.657437393 | 0.019979014 | *Cox7a2* | 0.624898913 | 1.12E-06 | *Rnf6* | 0.600764944 | 0.026356034 |
| *Riok1* | 0.657437393 | 0.044268911 | *Jund* | 0.624013254 | 3.70E-13 | *Map2k2* | 0.599413473 | 0.00311202 |
| *Agap3* | 0.657072384 | 0.043730948 | *Mob2* | 0.6233327 | 0.04879644 | *Bax* | 0.59896943 | 0.047875742 |
| *Creb3l2* | 0.656563826 | 0.003506392 | *Ppp2r2c* | 0.6233327 | 0.050486714 | *Sik3* | 0.598773096 | 1.68E-05 |
| *Igsf1* | 0.655806307 | 0.034902093 | *Iqcb1* | 0.62207545 | 0.032634521 | *Ociad1* | 0.598616477 | 0.02534995 |
| *Mroh1* | 0.655806307 | 0.037255815 | *mt-Nd3* | 0.621826559 | 0.000101183 | *Rlim* | 0.598616477 | 0.040063582 |
| *Elk3* | 0.655806307 | 0.001494719 | *Prickle2* | 0.621312321 | 0.028044738 | *Chd7* | 0.598090809 | 0.004548377 |
| *Parn* | 0.653021181 | 0.011446669 | *Wdfy3* | 0.621084928 | 0.012100775 | *Trpc1* | 0.598090809 | 0.023342877 |
| *Ddx6* | 0.652772247 | 0.004488092 | *Samd4b* | 0.621084928 | 0.01982688 | *Ints7* | 0.598090809 | 0.047355722 |
| *Xbp1* | 0.652111824 | 0.002478875 | *Zmat2* | 0.621084928 | 0.034380653 | *Thada* | 0.597224586 | 0.002541058 |
| *Gm32509* | 0.651391017 | 0.019723537 | *Rbm33* | 0.621084928 | 0.044736026 | *Fer* | 0.597224586 | 0.013498265 |
| *Magt1* | 0.650805626 | 0.02543051 | *Zranb2* | 0.620664501 | 0.015436289 | *Vwa8* | 0.596088456 | 0.004283402 |
| *Unc119b* | 0.650320768 | 0.02392957 | *Mest* | 0.619975873 | 3.54E-10 | *Fbxo42* | 0.595528295 | 0.047434882 |
| *Kdelr1* | 0.650320768 | 0.013386551 | *Crhbp* | 0.619680935 | 6.13E-20 | *Ubap2l* | 0.593554029 | 0.001762497 |
| *Prpf38b* | 0.648565664 | 0.014547702 | *Mrps12* | 0.619623971 | 0.017604755 | *Hmgn1* | 0.593415488 | 0.000447708 |
| *Snrnp27* | 0.648565664 | 0.019394146 | *Dnajc7* | 0.619623971 | 0.018349312 | *Rerg* | 0.593070552 | 0.022138029 |
| *Ppp1r12c* | 0.648565664 | 0.019394146 | *Cd24a* | 0.619623971 | 0.031976199 | *Phax* | 0.593070552 | 0.030034271 |
| *Immp2l* | 0.648070658 | 0.000194458 | *Fgd2* | 0.619623971 | 0.036580063 | *Nub1* | 0.593070552 | 0.035097848 |
| *Emd* | 0.648070658 | 0.023220694 | *Etnk1* | 0.619623971 | 0.040581619 | *Nr4a2* | 0.591604944 | 1.32E-08 |
| *Gripap1* | 0.647701257 | 0.013170182 | *Hadha* | 0.618192015 | 0.034456936 | *Vmp1* | 0.590711279 | 0.007633256 |
| *Cfap20* | 0.646714529 | 0.000688197 | *B830012L14Rik* | 0.617253066 | 4.54E-05 | *Snrpf* | 0.590711279 | 0.028701641 |
| *Arl4a* | 0.645159063 | 0.020219238 | *Rgs7* | 0.616706487 | 0.002370634 | *Tnpo3* | 0.590711279 | 0.042157708 |
| *Amph* | 0.645159063 | 0.020484684 | *Trpc4ap* | 0.616589911 | 0.030667236 | *Ago2* | 0.590711279 | 0.000248383 |
| *Srebf1* | 0.645159063 | 0.033450406 | *Impdh1* | 0.616096597 | 0.021457899 | *Gtf2h5* | 0.589190251 | 0.013558456 |
| *Pias2* | 0.645159063 | 0.039150543 | *Gtf3c2* | 0.616096597 | 0.0454542 | *Myh10* | 0.588890843 | 0.020820483 |
| *Arf4* | 0.64324186 | 7.05E-07 | *Bltp2* | 0.616096597 | 0.0454542 | *Scg2* | 0.588444672 | 0.000440397 |
| *Pard3* | 0.64283401 | 0.000498793 | *Mbd3* | 0.61541172 | 0.019215571 | *Med12l* | 0.587443565 | 0.003424361 |
| *Ssh2* | 0.642690807 | 0.010347619 | *Cdk11b* | 0.615164323 | 0.013572846 | *Cacna1g* | 0.587126942 | 0.013152034 |
| *Cltc* | 0.641426344 | 8.65E-05 | *Wac* | 0.614396809 | 0.00077853 | *Gm56999* | 0.586982419 | 0.001340617 |
| *U2surp* | 0.640820127 | 0.008467904 | *Cdc42bpa* | 0.613940332 | 0.000682481 | *Alkbh5* | 0.586879417 | 0.008315185 |
| *Sec31a* | 0.639769834 | 7.22E-05 | *Tmem248* | 0.612737585 | 0.020247409 | *Dmxl1* | 0.585658051 | 0.009450166 |
| *Tle4* | 0.639620879 | 4.66E-05 | *Mrpl1* | 0.612737585 | 0.049362515 | *Elavl1* | 0.584858553 | 0.032828495 |
| *Usp16* | 0.639620879 | 0.020057221 | *Rps28* | 0.611793722 | 1.30E-17 | *Ift88* | 0.584858553 | 0.03781187 |
| *mt-Nd4l* | 0.638732794 | 7.75E-06 | *Ndufa1* | 0.611555533 | 0.001083308 | *Msl2* | 0.584858553 | 0.051461807 |
| *Myl12b* | 0.638732794 | 0.030017081 | *Nin* | 0.610163642 | 0.02952644 | *Ric8b* | 0.583758518 | 0.014962956 |
| *Frmd5* | 0.638292343 | 0.000137114 | *Hspa13* | 0.610163642 | 0.049134869 | *6330403K07Rik* | 0.583758518 | 0.020101353 |
| *Meis1* | 0.63750549 | 0.034358148 | *Sec16a* | 0.609535153 | 0.007468319 | *Rufy3* | 0.583758518 | 0.022639236 |
| *Anapc13* | 0.636985131 | 0.015212422 | *Hikeshi* | 0.609535153 | 0.043764853 | *Pip5k1a* | 0.582149265 | 0.050209001 |
| *Pi4k2a* | 0.636985131 | 0.016555535 | *Tafa5* | 0.609089808 | 0.035721508 | *Atp5mpl* | 0.580844249 | 0.010542336 |
| *Pcca* | 0.634890727 | 0.000271696 | *Ubn2* | 0.606763684 | 5.87E-05 |  |  |  |

| **Supplemental Table S7I DEGS LAC2 Cluster; VCD/Vehicle.** Blue=downregulated | | | | | | | | |
| --- | --- | --- | --- | --- | --- | --- | --- | --- |
| **gene** | **avg_log2FC** | **p_val** | **gene** | **avg_log2FC** | **p_val** | **gene** | **avg_log2FC** | **p_val** |
| *Gm54798* | -0.587050217 | 0.053531794 | *Gm28529* | -1.524765939 | 0.052007017 |  |  |  |
| *C130073E24Rik* | -0.588268881 | 0.00282503 | *Fam53b* | -1.564294303 | 0.001193213 |  |  |  |
| *Uhrf2* | -0.633290395 | 0.032147752 | *Ctsc* | -1.595155266 | 0.016365851 |  |  |  |
| *Kcnip4* | -0.644064867 | 6.78E-05 | *Iah1* | -1.61222878 | 0.029128168 |  |  |  |
| *Gm20125* | -0.673629324 | 0.00319708 | *0610043K17Rik* | -1.640243156 | 0.035627755 |  |  |  |
| *Rsrp1* | -0.681735285 | 0.000484069 | *Bbs12* | -1.640243156 | 0.051885969 |  |  |  |
| *Hscb* | -0.70919051 | 0.052151437 | *AA465934* | -1.772693452 | 0.00984138 |  |  |  |
| *A630089N07Rik* | -0.712392942 | 0.050379485 | *Prrg1* | -1.832194464 | 5.06E-06 |  |  |  |
| *Aurkaip1* | -0.721163151 | 0.036147635 | *Gm2516* | -1.846694033 | 0.009805148 |  |  |  |
| *Fgf13* | -0.732208018 | 0.005558258 | *Rad52* | -1.846694033 | 0.040522554 |  |  |  |
| *Ppp1r1c* | -0.738890744 | 0.013564824 | *Gm34961* | -1.846694033 | 0.040522554 |  |  |  |
| *Pip4k2a* | -0.755063558 | 0.050119392 | *Gm48914* | -1.846694033 | 0.040522554 |  |  |  |
| *Ei24* | -0.756091485 | 0.035599169 | *Gm527* | -1.939803438 | 0.016624786 |  |  |  |
| *Ppfibp1* | -0.757426695 | 0.04233503 | *Gm16196* | -1.984197557 | 0.027054842 |  |  |  |
| *Lmo3* | -0.772693452 | 0.054782225 | *Gm47271* | -2.225205657 | 0.012133831 |  |  |  |
| *Tenm3* | -0.778522531 | 0.007575411 | *Misp* | -2.332120861 | 0.008145465 |  |  |  |
| *Gm45341* | -0.778522531 | 0.04837824 | *Serinc5* | -2.431656534 | 0.012099438 |  |  |  |
| *Exph5* | -0.797784433 | 0.049641343 | *C8g* | -2.524765939 | 0.024504954 |  |  |  |
| *1110059E24Rik* | -0.797784433 | 0.049641343 | *6430550D23Rik* | -2.524765939 | 0.024504954 |  |  |  |
| *Ano4* | -0.811070124 | 0.0047541 | *Nfatc2ip* | -2.524765939 | 0.024504954 |  |  |  |
| *Tmem106c* | -0.814272556 | 0.019224818 | *Jrkl* | -2.524765939 | 0.024504954 |  |  |  |
| *Qpct* | -0.832888234 | 0.008586465 | *Gm33091* | -2.524765939 | 0.024504954 |  |  |  |
| *Rpl10-ps3* | -0.832888234 | 0.020121529 | *Gm48383* | -2.524765939 | 0.024504954 |  |  |  |
| *Rasa4* | -0.846694033 | 0.044443738 | *Enpp2* | -2.524765939 | 0.024504954 |  |  |  |
| *Slc38a9* | -0.917083361 | 0.020833551 | *ENSMUSG00000121395* | -2.524765939 | 0.024504954 |  |  |  |
| *Ttf1* | -0.96533853 | 0.020408696 | *Acat2* | -2.524765939 | 0.024504954 |  |  |  |
| *Farsb* | -0.975427348 | 0.015298708 | *H2ac20* | -2.69469094 | 0.016148921 |  |  |  |
| *4930570G19Rik* | -1.016619035 | 0.014612556 | *Gm56964* | -2.69469094 | 0.016148921 |  |  |  |
| *Gm45323* | -1.022265598 | 0.018166426 | *Gm4577* | -2.69469094 | 0.016148921 |  |  |  |
| *Atxn3* | -1.048327895 | 0.050526476 | *Lmntd1* | -2.846694033 | 0.010691482 |  |  |  |
| *Dbnl* | -1.052012941 | 0.00839286 | *Pkp3* | -2.846694033 | 0.010691482 |  |  |  |
| *Gas6* | -1.055280655 | 0.050068542 | *Ucp1* | -3.109728439 | 0.010690938 |  |  |  |
| *Per3* | -1.06533432 | 0.027913755 | *Gm28529* | -1.524765939 | 0.052007017 |  |  |  |
| *2900005J15Rik* | -1.06533432 | 0.027913755 | *Fam53b* | -1.564294303 | 0.001193213 |  |  |  |
| *Ly6a* | -1.081159287 | 0.030498085 | *Ctsc* | -1.595155266 | 0.016365851 |  |  |  |
| *Zfp60* | -1.168622128 | 0.037277523 | *Iah1* | -1.61222878 | 0.029128168 |  |  |  |
| *Thap4* | -1.176842635 | 0.036684647 | *0610043K17Rik* | -1.640243156 | 0.035627755 |  |  |  |
| *Gm30866* | -1.225205657 | 0.049703896 | *Bbs12* | -1.640243156 | 0.051885969 |  |  |  |
| *Pml* | -1.290300685 | 0.045963485 | *AA465934* | -1.772693452 | 0.00984138 |  |  |  |
| *Pik3c2g* | -1.302373517 | 0.049498733 | *Prrg1* | -1.832194464 | 5.06E-06 |  |  |  |
| *Tmem184a* | -1.372762845 | 0.032748299 | *Gm2516* | -1.846694033 | 0.009805148 |  |  |  |
| *Enox1* | -1.431656534 | 0.041795479 | *Rad52* | -1.846694033 | 0.040522554 |  |  |  |
| *Zfp105* | -1.450765357 | 0.02324183 | *Gm34961* | -1.846694033 | 0.040522554 |  |  |  |
| *Pnp* | -1.450765357 | 0.032615021 | *Gm48914* | -1.846694033 | 0.040522554 |  |  |  |
| *Rpa1* | -1.524765939 | 0.009261436 | *Gm527* | -1.939803438 | 0.016624786 |  |  |  |
| *Nek3* | -1.524765939 | 0.011592506 | *Gm16196* | -1.984197557 | 0.027054842 |  |  |  |
| *Klf8* | -1.524765939 | 0.012930183 | *Gm47271* | -2.225205657 | 0.012133831 |  |  |  |
| *Abcb9* | -1.524765939 | 0.029225388 | *Misp* | -2.332120861 | 0.008145465 |  |  |  |
| *Zfp84* | -1.524765939 | 0.029225388 | *Serinc5* | -2.431656534 | 0.012099438 |  |  |  |
| *Ralb* | -1.524765939 | 0.032491629 | *C8g* | -2.524765939 | 0.024504954 |  |  |  |
| *Coa5* | -1.524765939 | 0.041661287 | *6430550D23Rik* | -2.524765939 | 0.024504954 |  |  |  |
| *2700069I18Rik* | -1.524765939 | 0.052007017 | *Nfatc2ip* | -2.524765939 | 0.024504954 |  |  |  |
| *Rnf122* | -1.524765939 | 0.052007017 | *Jrkl* | -2.524765939 | 0.024504954 |  |  |  |
| *Etohd2* | -1.524765939 | 0.052007017 | *Gm33091* | -2.524765939 | 0.024504954 |  |  |  |
| *Tspan14* | -1.524765939 | 0.052007017 | *Gm48383* | -2.524765939 | 0.024504954 |  |  |  |
| *Gm28529* | -1.524765939 | 0.052007017 |  |  |  |  |  |  |
| *Gm54798* | -0.587050217 | 0.053531794 |  |  |  |  |  |  |

| **Supplemental Table S8A DEGS LAC3 Cluster; VCD/Vehicle.** Maroon=upregulated; | | | | | | | | |
| --- | --- | --- | --- | --- | --- | --- | --- | --- |
| **gene** | **avg_log2FC** | **p_val** | **gene** | **avg_log2FC** | **p_val** | **gene** | **avg_log2FC** | **p_val** |
| *Mlst8* | 4.064386687 | 0.000599443 | *Gm56815* | 2.327421093 | 0.019821847 | *Wfikkn1* | 2.064386687 | 0.04870936 |
| *Minar1* | 3.912383594 | 0.001495574 | *Myo18b* | 2.327421093 | 0.019821847 | *Gm56507* | 2.064386687 | 0.04870936 |
| *Brinp3* | 3.742458593 | 0.009602706 | *Gm15492* | 2.327421093 | 0.019821847 | *Gm6133* | 2.064386687 | 0.04870936 |
| *Xrcc2* | 3.549813515 | 0.003764823 | *Gm15494* | 2.327421093 | 0.019821847 | *Pnldc1* | 2.064386687 | 0.04870936 |
| *Plet1* | 3.549813515 | 0.003764823 | *Zfp59* | 2.327421093 | 0.019821847 | *Tsc22d3* | 2.022566512 | 0.001125012 |
| *Tmcc3os* | 3.549813515 | 0.003764823 | *Plekhg2* | 2.327421093 | 0.019821847 | *Dmap1* | 1.964851014 | 0.017975698 |
| *P3h2* | 3.549813515 | 0.009602706 | *Dbndd1* | 2.327421093 | 0.019821847 | *Gm15417* | 1.964851014 | 0.0275055 |
| *4933439K11Rik* | 3.327421093 | 0.003764896 | *Nav3* | 2.327421093 | 0.019821847 | *1810059H22Rik* | 1.964851014 | 0.0275055 |
| *Zfp526* | 3.327421093 | 0.003764896 | *Gm57337* | 2.327421093 | 0.019821847 | *Foxred1* | 1.964851014 | 0.0275055 |
| *Gm6034* | 3.327421093 | 0.003764896 | *Sdc1* | 2.327421093 | 0.019821847 | *C920006O11Rik* | 1.964851014 | 0.0275055 |
| *Prps2* | 3.327421093 | 0.003764896 | *Gm50071* | 2.327421093 | 0.019821847 | *Gm26577* | 1.964851014 | 0.0275055 |
| *Suv39h2* | 3.327421093 | 0.00960265 | *Vwa7* | 2.327421093 | 0.019821847 | *Dcaf4* | 1.964851014 | 0.0275055 |
| *Dph1* | 3.327421093 | 0.00960265 | *Slc35g1* | 2.327421093 | 0.019821847 | *Tcp11* | 1.964851014 | 0.0275055 |
| *Gm47802* | 3.327421093 | 0.00960265 | *Cfap157* | 2.327421093 | 0.048601038 | *Zbtb12* | 1.964851014 | 0.0275055 |
| *Gckr* | 3.201890211 | 0.019685345 | *Cd53* | 2.327421093 | 0.048601038 | *Egr4* | 1.944092454 | 0.002520525 |
| *Cd86* | 3.201890211 | 0.019730612 | *1700019L13Rik* | 2.327421093 | 0.048601038 | *Pex12* | 1.93510367 | 0.002335519 |
| *Inpp4b* | 3.120970216 | 0.034398491 | *Mir142hg* | 2.327421093 | 0.048601038 | *Xlr3a* | 1.912383594 | 0.002384197 |
| *Atp2b4* | 3.064386687 | 0.009602725 | *Fam171a2* | 2.327421093 | 0.048601038 | *Gm6225* | 1.912383594 | 0.00251953 |
| *Cfap77* | 3.064386687 | 0.009602725 | *Rhbdf2* | 2.327421093 | 0.048601038 | *Srrm4os* | 1.912383594 | 0.012118545 |
| *Chac1* | 3.064386687 | 0.009602725 | *Ly86* | 2.327421093 | 0.048601038 | *Fam210b* | 1.912383594 | 0.015000417 |
| *Hao1* | 3.064386687 | 0.009602725 | *Dab2* | 2.327421093 | 0.048601038 | *Gm38948* | 1.912383594 | 0.015000417 |
| *Prkag2os1* | 3.064386687 | 0.009602725 | *Prodh* | 2.327421093 | 0.048601038 | *Slc22a4* | 1.912383594 | 0.032281303 |
| *Gm16223* | 3.064386687 | 0.009602725 | *Gm16278* | 2.327421093 | 0.048601038 | *Otub2* | 1.912383594 | 0.032281303 |
| *Gm56952* | 3.064386687 | 0.009602725 | *Fbxo46* | 2.201890211 | 0.002256449 | *Ndst2* | 1.879962116 | 0.003118321 |
| *9130019O22Rik* | 3.064386687 | 0.009602725 | *ENSMUSG00000121479* | 2.201890211 | 0.006781518 | *Gpn2* | 1.879962116 | 0.008112214 |
| *Pygo1* | 3.064386687 | 0.009602725 | *A430090L17Rik* | 2.201890211 | 0.032281303 | *Dock2* | 1.879962116 | 0.01219162 |
| *Gm56741* | 3.064386687 | 0.009602725 | *Iqcn* | 2.157496092 | 0.011998703 | *Slc27a4* | 1.879962116 | 0.01697017 |
| *Gm50012* | 3.064386687 | 0.009602725 | *Ulk3* | 2.157496092 | 0.011998703 | *Frat1* | 1.879962116 | 0.01697017 |
| *Gm14216* | 2.990386106 | 3.07E-05 | *Dnaaf4* | 2.157496092 | 0.011998703 | *Klf11* | 1.879962116 | 0.032336864 |
| *Runx1* | 2.964851014 | 0.000563552 | *Serpine2* | 2.157496092 | 0.027399468 | *H2-Ab1* | 1.879962116 | 0.036090473 |
| *Rfx4* | 2.912383594 | 0.001355979 | *Zbtb48* | 2.157496092 | 0.027399468 | *Them6* | 1.85793581 | 0.004376605 |
| *Rptoros* | 2.912383594 | 0.003300195 | *Il18bp* | 2.157496092 | 0.027399468 | *Amt* | 1.85793581 | 0.008981585 |
| *Smim41* | 2.912383594 | 0.008049832 | *Zfp3* | 2.157496092 | 0.027399468 | *Cyp51* | 1.80658893 | 0.000754598 |
| *Cidea* | 2.912383594 | 0.048386312 | *Fosl1* | 2.157496092 | 0.027399468 | *Gm42439* | 1.742458593 | 0.001467467 |
| *Chrna6* | 2.742458593 | 0.000402671 | *5730419F03Rik* | 2.120970216 | 0.001379552 | *Elac2* | 1.742458593 | 0.004425096 |
| *Col24a1* | 2.742458593 | 0.00516165 | *Apba3* | 2.064386687 | 0.002063236 | *Bora* | 1.742458593 | 0.004821104 |
| *Ak4* | 2.742458593 | 0.00806874 | *Mon1a* | 2.064386687 | 0.006820165 | *Taf1c* | 1.742458593 | 0.006932945 |
| *Prtn3* | 2.742458593 | 0.00806874 | *Gm56541* | 2.064386687 | 0.006820165 | *Samd11* | 1.742458593 | 0.008192789 |
| *Plk5* | 2.742458593 | 0.00806874 | *Gm17484* | 2.064386687 | 0.014919691 | *Zswim1* | 1.742458593 | 0.009594719 |
| *Cdkn1a* | 2.742458593 | 0.00806874 | *Haus8* | 2.064386687 | 0.014919691 | *Tm6sf1* | 1.742458593 | 0.015186187 |
| *H2-Q7* | 2.742458593 | 0.048601038 | *Igtp* | 2.064386687 | 0.014919691 | *Spsb3* | 1.742458593 | 0.017081598 |
| *Prss29* | 2.649349188 | 0.005172774 | *1700110C19Rik* | 2.064386687 | 0.032281303 | *Bcar3* | 1.742458593 | 0.018743569 |
| *Ovgp1* | 2.549813515 | 0.008088161 | *2810408I11Rik* | 2.064386687 | 0.04870936 | *Hmcn2* | 1.742458593 | 0.020050573 |
| *Nlrx1* | 2.549813515 | 0.008088161 | *Gm14286* | 2.064386687 | 0.04870936 | *Laptm5* | 1.742458593 | 0.027715623 |
| *Igflr1* | 2.549813515 | 0.019775862 | *Pdgfc* | 2.064386687 | 0.04870936 | *Lcmt2* | 1.742458593 | 0.032445774 |
| *Il20rb* | 2.549813515 | 0.019775862 | *Slc46a3* | 2.064386687 | 0.04870936 | *Ankef1* | 1.742458593 | 0.032445774 |
| *Ttll1* | 2.549813515 | 0.019775862 | *Lrfn1* | 2.064386687 | 0.04870936 | *Metrnl* | 1.742458593 | 0.032445774 |
| *Cd83* | 2.549813515 | 0.048601038 | *Scube2* | 2.064386687 | 0.04870936 | *Gm57271* | 1.742458593 | 0.032499007 |
| *Nkrf* | 2.479424187 | 0.00516165 | *Gm45307* | 2.064386687 | 0.04870936 | *Idua* | 1.742458593 | 0.032607234 |
| *Bmp6* | 2.479424187 | 0.027191437 | *Nmnat3* | 2.064386687 | 0.04870936 | *Trdmt1* | 1.742458593 | 0.035026173 |
| *Fam20a* | 2.442898311 | 0.001349927 | *Odad4* | 2.064386687 | 0.04870936 | *Mtx3* | 1.742458593 | 0.035490239 |
| *Dhx37* | 2.327421093 | 0.005183636 | *Gm34907* | 2.064386687 | 0.04870936 | *Pfkfb3* | 1.742458593 | 0.035986595 |
| *Gm26638* | 2.327421093 | 0.005183636 | *Csdc2* | 2.064386687 | 0.04870936 | *Shroom4* | 1.742458593 | 0.049033934 |
| *Gm17066* | 2.327421093 | 0.005183636 | *Ppl* | 2.064386687 | 0.04870936 | *Gm15283* | 1.703984445 | 9.24E-06 |
| *Gm42937* | 2.327421093 | 0.006744394 | *Gm49595* | 2.064386687 | 0.04870936 | *Zfp503* | 1.681058048 | 0.001486071 |
| *Gm38251* | 2.327421093 | 0.011950005 | *Plaat1* | 2.064386687 | 0.04870936 | *Fam118b* | 1.675344397 | 0.001454776 |
| *Mfsd2a* | 2.327421093 | 0.011950005 | *6330415G19Rik* | 2.064386687 | 0.04870936 | *Mob1a* | 1.649349188 | 0.004870687 |

| **Supplemental Table S8B DEGS LAC3 Cluster; VCD/Vehicle.** Maroon=upregulated; | | | | | | | | |
| --- | --- | --- | --- | --- | --- | --- | --- | --- |
| **gene** | **avg_log2FC** | **p_val** | **gene** | **avg_log2FC** | **p_val** | **gene** | **avg_log2FC** | **p_val** |
| *Rusc1* | 1.635543389 | 0.00147224 | *Ndufaf1* | 1.420530498 | 0.033004098 | *Fndc4* | 1.22788542 | 0.014737227 |
| *Tomm40l* | 1.635543389 | 0.036098644 | *Efna1* | 1.420530498 | 0.033004098 | *Lpin1* | 1.211943876 | 0.000182033 |
| *Zfp597* | 1.635543389 | 0.036098644 | *Homer1* | 1.406492664 | 2.91E-06 | *Ddx21* | 1.211943876 | 0.000983796 |
| *Emp1* | 1.626981375 | 0.036103371 | *Peds1* | 1.394535289 | 0.000750183 | *Tspan12* | 1.211943876 | 0.019063167 |
| *Bmper* | 1.621152296 | 2.27E-07 | *Fndc10* | 1.394535289 | 0.036673246 | *Zbtb5* | 1.211943876 | 0.030205037 |
| *Pygo2* | 1.61692771 | 0.018380955 | *Zfp651* | 1.394535289 | 0.036694765 | *Piga* | 1.211943876 | 0.033459937 |
| *Tmem129* | 1.61692771 | 0.018380955 | *Tnr* | 1.386314782 | 0.00041035 | *Tmsb4x* | 1.203299781 | 0.000242532 |
| *Zyx* | 1.61692771 | 0.018380955 | *Mt2* | 1.379888513 | 0.001754686 | *Evc2* | 1.201890211 | 0.018927448 |
| *Sinhcaf* | 1.590455499 | 0.009944884 | *Trim11* | 1.379888513 | 0.009778337 | *Ampd2* | 1.201890211 | 0.037114185 |
| *H2ac25* | 1.590455499 | 0.032661719 | *Acsl4* | 1.379888513 | 0.009936165 | *Mfsd13a* | 1.201890211 | 0.037146237 |
| *Gm4211* | 1.590455499 | 0.035241823 | *Wdr46* | 1.379888513 | 0.019127828 | *Dcaf12l1* | 1.201890211 | 0.037146237 |
| *Kcnh2* | 1.57895986 | 0.002784952 | *Src* | 1.379888513 | 0.019391002 | *Gpbp1l1* | 1.198138076 | 0.000623363 |
| *Pex11b* | 1.572533591 | 0.002631027 | *Chrnb3* | 1.379888513 | 0.019391002 | *Ate1* | 1.189917569 | 0.000927326 |
| *Tmem43* | 1.572533591 | 0.005325454 | *Adck5* | 1.379888513 | 0.03472568 | *9630028H03Rik* | 1.189917569 | 0.007980766 |
| *Edc3* | 1.572533591 | 0.010029494 | *Nif3l1* | 1.379888513 | 0.036241715 | *Pik3r2* | 1.189917569 | 0.0171242 |
| *Srfbp1* | 1.572533591 | 0.010029494 | *Vwa5b1* | 1.379888513 | 0.03695699 | *Hhat* | 1.189917569 | 0.019035181 |
| *Rab11fip5* | 1.572533591 | 0.034401908 | *Ints1* | 1.370489815 | 0.017930878 | *Gm57395* | 1.189917569 | 0.032123415 |
| *Gipc2* | 1.549813515 | 0.027821132 | *Slc37a1* | 1.370489815 | 0.033156736 | *Pnkp* | 1.189917569 | 0.033833942 |
| *1700109H08Rik* | 1.549813515 | 0.035490239 | *Atf3* | 1.368239998 | 1.56E-06 | *Samd10* | 1.189917569 | 0.054898617 |
| *Ntrk3* | 1.540824731 | 0.000123742 | *Zmat3* | 1.363946969 | 0.000112684 | *Psmg3* | 1.183031184 | 0.017631822 |
| *Tpst1* | 1.536007715 | 0.004718202 | *Hspa1b* | 1.363946969 | 0.029548291 | *Smad3* | 1.183031184 | 0.028462632 |
| *Gm56517* | 1.530954487 | 0.001491978 | *Pisd* | 1.359129953 | 0.005087156 | *Efcab10* | 1.183031184 | 0.028462632 |
| *Tmem141* | 1.530954487 | 0.005401951 | *Mta2* | 1.352512074 | 0.004194136 | *Mctp1* | 1.183031184 | 0.033422281 |
| *Mzt2* | 1.530954487 | 0.031078043 | *Klf4* | 1.34538764 | 7.32E-07 | *Tceal5* | 1.175418 | 0.004124354 |
| *Gm43847* | 1.520066171 | 0.036380879 | *Ccdc82* | 1.327421093 | 0.024322258 | *Mob1b* | 1.171301891 | 0.000431765 |
| *Rab34* | 1.520066171 | 0.036380879 | *Fhip2b* | 1.327421093 | 0.031558376 | *Uba5* | 1.163922361 | 0.000162324 |
| *Dennd3* | 1.520066171 | 0.036380879 | *Phospho2* | 1.327421093 | 0.034892491 | *Baiap2* | 1.157496092 | 0.00016545 |
| *Gm13269* | 1.520066171 | 0.036441426 | *Samhd1* | 1.327421093 | 0.034892491 | *Map2k7* | 1.157496092 | 0.001145738 |
| *Rap1gapos* | 1.520066171 | 0.036441426 | *Gm47096* | 1.327421093 | 0.035665822 | *Higd1a* | 1.157496092 | 0.005679057 |
| *Eif2b2* | 1.501450493 | 0.002839098 | *Cldn6* | 1.327421093 | 0.035665822 | *Mterf2* | 1.157496092 | 0.015895253 |
| *Mms19* | 1.49248034 | 0.000377345 | *Col20a1* | 1.327421093 | 0.036858943 | *Jpt1* | 1.157496092 | 0.018454122 |
| *Alg10b* | 1.479424187 | 0.002680527 | *5730403I07Rik* | 1.327421093 | 0.036858943 | *Nt5dc3* | 1.157496092 | 0.021010533 |
| *Rab2b* | 1.479424187 | 0.005300786 | *Map3k5* | 1.30433748 | 0.000846262 | *Gm35417* | 1.157496092 | 0.036598516 |
| *Snhg11* | 1.479424187 | 0.010452315 | *Ctso* | 1.301886001 | 0.003943956 | *Rnf215* | 1.157496092 | 0.055072246 |
| *Myb* | 1.479424187 | 0.01680463 | *Nars2* | 1.301886001 | 0.004198589 | *Mt1* | 1.153246619 | 0.000641877 |
| *Chil6* | 1.479424187 | 0.034401908 | *Zfp646* | 1.294999616 | 0.009016284 | *Pde1c* | 1.148450952 | 0.002390489 |
| *Gm57170* | 1.479424187 | 0.036495263 | *2700097O09Rik* | 1.289946388 | 0.009465094 | *Rhob* | 1.146848848 | 0.001659707 |
| *Slc15a4* | 1.479424187 | 0.036495263 | *Ppp2r3c* | 1.289946388 | 0.009465094 | *Ppfia3* | 1.134776015 | 0.015982056 |
| *Dhrs11* | 1.479424187 | 0.036495263 | *Rel* | 1.289946388 | 0.031975129 | *Ccdc97* | 1.134776015 | 0.044123811 |
| *Gm13944* | 1.479424187 | 0.049142307 | *Sdhaf3* | 1.283026974 | 0.031760224 | *Rbm10* | 1.131500883 | 0.000215028 |
| *Gm11762* | 1.479424187 | 0.049142307 | *Mtx1* | 1.283026974 | 0.054342068 | *Tsc22d2* | 1.13102388 | 0.001275738 |
| *Cd74* | 1.46682415 | 0.054079308 | *Nmt2* | 1.278511493 | 0.000463428 | *Tomm70a* | 1.129481716 | 0.001163479 |
| *Chka* | 1.459151792 | 0.0001171 | *Rgcc* | 1.272973309 | 0.028648559 | *Ergic2* | 1.129481716 | 0.001959569 |
| *Tmem184a* | 1.452951975 | 0.017726587 | *Zfp703* | 1.266020549 | 0.008124701 | *Isg20l2* | 1.129481716 | 0.010643438 |
| *Nfya* | 1.452951975 | 0.017726587 | *Fndc9* | 1.263290756 | 0.006363539 | *Apobec1* | 1.129481716 | 0.019267648 |
| *Nit2* | 1.442898311 | 0.035796565 | *Fam78b* | 1.257031765 | 0.036949435 | *Nudt19* | 1.129481716 | 0.047782886 |
| *Tmbim1* | 1.434336297 | 0.000821321 | *Maff* | 1.257031765 | 0.01757714 | *1500026H17Rik* | 1.129481716 | 0.047782886 |
| *Phaf1* | 1.434336297 | 0.00534766 | *Nadk2* | 1.249418581 | 0.00263842 | *Kcnk10* | 1.127483523 | 0.000332824 |
| *Gtpbp8* | 1.434336297 | 0.009201089 | *Trmt13* | 1.249418581 | 0.006702233 | *Mcmbp* | 1.125787232 | 0.003956094 |
| *Per2* | 1.434336297 | 0.016901193 | *Kbtbd11* | 1.244958933 | 0.001842959 | *4921511C10Rik* | 1.120970216 | 0.036781626 |
| *Ikzf4* | 1.426956767 | 3.42E-05 | *Pacs2* | 1.244958933 | 0.018244609 | *Gm15614* | 1.11442737 | 0.015110736 |
| *Sbds* | 1.420530498 | 5.35E-05 | *Eno1* | 1.244958933 | 0.050740006 | *Ak6* | 1.111692402 | 0.00773628 |
| *Atp6v0e2* | 1.420530498 | 0.004792782 | *Hsd3b7* | 1.239958252 | 0.008178599 | *Sgms2* | 1.110190377 | 0.003477025 |
| *Gm26532* | 1.420530498 | 0.008761933 | *Ttc1* | 1.239958252 | 0.022983968 | *Nphp4* | 1.105028672 | 0.017847508 |
| *Aacs* | 1.420530498 | 0.014115103 | *Tacc2* | 1.22788542 | 0.006085989 | *Vegfb* | 1.105028672 | 0.028813188 |
| *Axin2* | 1.420530498 | 0.014115103 | *Tcirg1* | 1.22788542 | 0.00840179 | *Kat5* | 1.105028672 | 0.047927281 |
| *Dph5* | 1.420530498 | 0.018519744 | *Gfod2* | 1.22788542 | 0.00914582 | *2510039O18Rik* | 1.105028672 | 0.024690213 |

| **Supplemental Table S8C DEGS LAC3 Cluster; VCD/Vehicle.** Maroon=upregulated; | | | | | | | | |
| --- | --- | --- | --- | --- | --- | --- | --- | --- |
| **gene** | **avg_log2FC** | **p_val** | **gene** | **avg_log2FC** | **p_val** | **gene** | **avg_log2FC** | **p_val** |
| *Naaladl2* | 1.098602403 | 0.017057758 | *Il13ra1* | 0.973784139 | 0.018968875 | *Creb3l1* | 0.872928524 | 2.93E-05 |
| *Wipi1* | 1.096095547 | 0.013300902 | *Slc7a6os* | 0.973784139 | 0.040005945 | *Zc3h7b* | 0.871741609 | 0.00667367 |
| *Dhx29* | 1.096095547 | 0.020127127 | *Sclt1* | 0.96716488 | 3.00E-05 | *Mt3* | 0.871741609 | 0.026820784 |
| *Hk1* | 1.090381896 | 0.002561152 | *Rnd3* | 0.964851014 | 0.00576427 | *Trpc1* | 0.867989475 | 0.030449507 |
| *Cdc42se2* | 1.086412994 | 0.006613018 | *Asnsd1* | 0.964851014 | 0.038879302 | *Gm26542* | 0.867989475 | 0.04434839 |
| *Nup205* | 1.08349551 | 0.027066518 | *Spg7* | 0.964851014 | 0.045215364 | *Arih1* | 0.866638004 | 4.54E-07 |
| *Zxdc* | 1.07949358 | 0.008550809 | *Ezr* | 0.961098879 | 0.000190351 | *Mapk9* | 0.864449117 | 0.036109691 |
| *Gm32509* | 1.072607194 | 8.41E-06 | *Snrpa1* | 0.956583398 | 0.009681747 | *Armc1* | 0.861103089 | 0.027469073 |
| *P2ry14* | 1.064386687 | 0.005486441 | *Msl3* | 0.953962698 | 0.014744956 | *Dpysl3* | 0.85793581 | 0.037328153 |
| *Tollip* | 1.064386687 | 0.006220447 | *Opa3* | 0.951911958 | 0.042520423 | *Actr3* | 0.856115374 | 0.005590662 |
| *Gcdh* | 1.064386687 | 0.01591201 | *Cep43* | 0.94890947 | 0.041331585 | *Trib1* | 0.854933322 | 0.020484357 |
| *Plaa* | 1.064386687 | 0.022788679 | *Fam184a* | 0.94890947 | 0.008278246 | *Znhit1* | 0.849373796 | 0.00536807 |
| *Gorasp1* | 1.064386687 | 0.029057378 | *Cox19* | 0.944092454 | 0.047501839 | *2510009E07Rik* | 0.849373796 | 0.024615963 |
| *Gm11734* | 1.064386687 | 0.029681612 | *Phlpp2* | 0.944092454 | 0.051833452 | *Uhrf1bp1l* | 0.846795252 | 0.005388101 |
| *Pde12* | 1.064386687 | 0.032600264 | *Syncrip* | 0.938855805 | 0.001851199 | *Ddx18* | 0.845552085 | 0.013554526 |
| *Gm13912* | 1.064386687 | 0.036359352 | *Tmem214* | 0.93510367 | 0.005139387 | *Tspan2* | 0.845552085 | 0.02934143 |
| *Gm38882* | 1.064386687 | 0.037414773 | *Rnf24* | 0.93510367 | 0.008425363 | *Cdv3* | 0.844338207 | 0.004519297 |
| *Ankrd16* | 1.064386687 | 0.038136396 | *Romo1* | 0.928871717 | 0.002828273 | *Nipa2* | 0.844338207 | 0.024549515 |
| *Repin1* | 1.064386687 | 0.051881 | *Cebpg* | 0.926883164 | 0.030788581 | *Zrsr2* | 0.841994266 | 0.003618209 |
| *Ino80e* | 1.064386687 | 0.051881 | *Tmem158* | 0.926883164 | 0.04767186 | *Brinp1* | 0.838673908 | 0.003033444 |
| *Gnpat* | 1.052798713 | 0.017589859 | *Bsn* | 0.923030838 | 0.01113322 | *Cfap418* | 0.837615826 | 0.02308405 |
| *Gm56830* | 1.052798713 | 0.020978201 | *Bag2* | 0.923030838 | 0.021334543 | *Gm11508* | 0.835567997 | 0.019433638 |
| *Tmem242* | 1.050580888 | 0.038667304 | *Afg3l2* | 0.923030838 | 0.028738863 | *Ing2* | 0.833606481 | 0.007029164 |
| *Mthfr* | 1.047313174 | 0.015328263 | *Zbtb11* | 0.919336355 | 0.000153404 | *Nub1* | 0.829921434 | 0.003104782 |
| *Imp4* | 1.042018874 | 0.01076302 | *Dcun1d2* | 0.919336355 | 0.017780031 | *Fam76b* | 0.829921434 | 0.025007026 |
| *1810062O18Rik* | 1.042018874 | 0.052015195 | *Ppp1cc* | 0.919336355 | 0.025041398 | *Sv2a* | 0.828188467 | 0.006630395 |
| *Socs1* | 1.042018874 | 0.052015195 | *Gon7* | 0.919336355 | 0.033581317 | *Arfip2* | 0.828188467 | 0.011324394 |
| *Flvcr1* | 1.037914476 | 0.022079626 | *Clpx* | 0.918308428 | 0.013050706 | *Btg1* | 0.826522857 | 0.013707888 |
| *Endog* | 1.037914476 | 0.025180695 | *Ugcg* | 0.917545299 | 0.042540795 | *Fstl5* | 0.824920753 | 0.006155582 |
| *Sox2ot* | 1.03196521 | 0.002923255 | *Dot1l* | 0.912383594 | 0.005106746 | *Msmo1* | 0.824920753 | 0.043052915 |
| *Tmem184b* | 1.03196521 | 0.00335348 | *Cflar* | 0.912383594 | 0.012611388 | *Tial1* | 0.822628941 | 0.000980264 |
| *Vdac1* | 1.03196521 | 0.011321948 | *Pip5k1c* | 0.912383594 | 0.024458105 | *Chp1* | 0.82189306 | 0.003189213 |
| *Wfs1* | 1.03196521 | 0.032576556 | *Mrpl41* | 0.909568578 | 0.009802158 | *Aven* | 0.816459174 | 0.026721885 |
| *Hmgcs1* | 1.03196521 | 0.035367879 | *Camsap1* | 0.909108462 | 0.001100536 | *Kpna1* | 0.811999526 | 0.001981448 |
| *Gm41604* | 1.03196521 | 0.038356267 | *Prkaca* | 0.904730021 | 0.000798945 | *Nploc4* | 0.809572788 | 0.013261445 |
| *Ankrd27* | 1.03196521 | 0.038808846 | *Igsf1* | 0.904209663 | 0.00222141 | *Odc1* | 0.809572788 | 0.003341485 |
| *Stat3* | 1.030260904 | 8.57E-05 | *Drosha* | 0.902923265 | 0.041944011 | *Gapvd1* | 0.807309737 | 0.001095709 |
| *Ankra2* | 1.022566512 | 0.004529854 | *Fbxo16* | 0.89999987 | 0.014383612 | *Uba52* | 0.80658893 | 0.031755211 |
| *Atp8b2* | 1.022566512 | 0.011757478 | *Dbpht2* | 0.896968542 | 0.000102236 | *Dnajb1* | 0.804742871 | 0.002001765 |
| *Dusp8* | 1.019992568 | 0.008520612 | *Lsr* | 0.894461686 | 0.014695965 | *Elovl6* | 0.804742871 | 0.028428578 |
| *Stip1* | 1.019298798 | 6.09E-05 | *Mtln* | 0.894461686 | 0.015810103 | *Cops6* | 0.803859137 | 0.028272418 |
| *Nr4a3* | 1.018093035 | 0.002592076 | *Dcaf11* | 0.894461686 | 0.015855475 | *Ptpn9* | 0.802579585 | 0.01625173 |
| *Klhl13* | 1.012547756 | 0.014558392 | *Myo9b* | 0.894461686 | 0.031044182 | *Foxo3* | 0.801352282 | 0.007960396 |
| *Agfg1* | 1.010947428 | 0.008785521 | *Prmt9* | 0.894461686 | 0.042627282 | *Gm41077* | 0.796906377 | 0.036841702 |
| *Rab3b* | 1.005492998 | 0.020868311 | *Eno2* | 0.888309459 | 0.00185126 | *Atrn* | 0.796906377 | 0.021492957 |
| *Zbtb7c* | 1.005492998 | 0.025022062 | *Sec24c* | 0.886848502 | 0.023964454 | *Rpa3* | 0.794926012 | 0.04362168 |
| *Efcab15* | 1.005492998 | 0.027418068 | *D830024N08Rik* | 0.886848502 | 0.052471662 | *Pptc7* | 0.793084666 | 0.017939702 |
| *Rab26os* | 1.005492998 | 0.041752818 | *B4galt6* | 0.883321128 | 0.022320507 | *Tns3* | 0.793084666 | 0.032135847 |
| *Rnf150* | 1.005492998 | 0.048020051 | *Smarcad1* | 0.883321128 | 0.036067076 | *Phf8* | 0.790552881 | 0.023211612 |
| *Ints3* | 0.997715648 | 0.013945202 | *Srrt* | 0.882636251 | 0.009584318 | *Acot7* | 0.789764307 | 0.048483194 |
| *Fam20c* | 0.990386106 | 0.027532063 | *Aff2* | 0.882183356 | 0.001159217 | *Nr3c2* | 0.789001178 | 0.016056602 |
| *Ttc32* | 0.990386106 | 0.041839933 | *Sec23a* | 0.88162134 | 0.000601067 | *Snrpe* | 0.788752245 | 0.002935421 |
| *Trp53i11* | 0.98757109 | 0.002019714 | *S100a1* | 0.879962116 | 0.039632716 | *Kat6a* | 0.785527314 | 0.004265012 |
| *Rcc2* | 0.981646256 | 0.00023096 | *Spaca6* | 0.879962116 | 0.052050643 | *Arhgap1* | 0.784278768 | 0.031796736 |
| *Pcyt1a* | 0.97949779 | 0.006974695 | *Cacna1b* | 0.876759684 | 0.009791531 | *Srprb* | 0.784278768 | 0.047951191 |
| *Gm48321* | 0.97949779 | 0.054418048 | *Scamp2* | 0.875725123 | 0.021816108 | *Git2* | 0.783100577 | 0.028044929 |
| *1600010M07Rik* | 0.976923846 | 0.008319023 | *Sypl* | 0.873703126 | 0.021340898 | *Ugdh* | 0.781986957 | 0.033740207 |

| **Supplemental Table S8D DEGS LAC3 Cluster; VCD/Vehicle.** Maroon=upregulated; | | | | | | | | |
| --- | --- | --- | --- | --- | --- | --- | --- | --- |
| **gene** | **avg_log2FC** | **p_val** | **gene** | **avg_log2FC** | **p_val** | **gene** | **avg_log2FC** | **p_val** |
| *Wdr37* | 0.781986957 | 0.053354796 | *Ckap4* | 0.706834683 | 0.026478912 | *Eif3g* | 0.654995751 | 0.044124145 |
| *Abcc5* | 0.78093274 | 0.014431246 | *Tmem147* | 0.705932717 | 0.006081578 | *Csnk2a2* | 0.651310704 | 0.045785759 |
| *Bcl7b* | 0.779933298 | 0.028139537 | *Foxk2* | 0.704983887 | 0.010546186 | *Dph3* | 0.650828117 | 0.009047357 |
| *Rundc1* | 0.777224011 | 0.021889161 | *Bhlhe40* | 0.703984445 | 0.023575328 | *9930021J03Rik* | 0.649349188 | 0.003792143 |
| *Lrrc73* | 0.777224011 | 0.025209682 | *Btbd7* | 0.703464461 | 0.019164448 | *Ndufa3* | 0.649349188 | 0.046900081 |
| *Mpp7* | 0.774342654 | 8.44E-05 | *Rpn1* | 0.702381153 | 0.028384543 | *Snrpg* | 0.648336415 | 0.002082571 |
| *Rnf4* | 0.774167452 | 0.024314075 | *Rps26* | 0.70091633 | 2.21E-07 | *Mast3* | 0.646880933 | 0.002517149 |
| *Hip1r* | 0.772832242 | 0.006638013 | *Btbd10* | 0.700638417 | 0.041664879 | *Myo16* | 0.645161391 | 0.010398097 |
| *Stx3* | 0.771027745 | 0.01672279 | *E530011L22Rik* | 0.699389871 | 0.005092495 | *Atp2a2* | 0.644278199 | 0.00126759 |
| *Larp4* | 0.769939329 | 0.007982281 | *Dop1b* | 0.698737215 | 0.006579773 | *Usp48* | 0.644278199 | 0.007081191 |
| *Rcn3* | 0.768930804 | 0.01740196 | *Phka1* | 0.698064473 | 0.00901782 | *Rpl39* | 0.644194576 | 2.33E-07 |
| *Hsph1* | 0.768690134 | 0.00185146 | *Rnf217* | 0.697720278 | 0.010261864 | *Nfkbia* | 0.643894758 | 0.005370928 |
| *Acvr1* | 0.767993685 | 0.021202224 | *Hibadh* | 0.695916007 | 0.016644193 | *Cpq* | 0.642922919 | 6.11E-06 |
| *Dpp6* | 0.766305334 | 0.000222207 | *Dock7* | 0.693548992 | 0.007935981 | *Pmepa1* | 0.642922919 | 0.010597538 |
| *Jmjd1c* | 0.765178669 | 1.40E-05 | *Cacna1e* | 0.693548992 | 0.042000739 | *Rasd1* | 0.642922919 | 0.035270537 |
| *Rwdd1* | 0.764826406 | 0.002164195 | *Spire1* | 0.692705557 | 0.02954533 | *Tbck* | 0.641175257 | 0.008107299 |
| *Ccnl1* | 0.757255594 | 0.002564423 | *Gm26802* | 0.692533367 | 0.000397756 | *Yipf6* | 0.640578979 | 0.052955991 |
| *Dynll1* | 0.752239613 | 6.07E-05 | *Tenm4* | 0.688010809 | 0.001665226 | *Zmpste24* | 0.6393651 | 0.039638053 |
| *Zdhhc5* | 0.742458593 | 0.01482821 | *Setx* | 0.688010809 | 0.004373991 | *Septin9* | 0.637489033 | 0.034133908 |
| *Trappc10* | 0.742458593 | 0.01489946 | *Pcsk5* | 0.688010809 | 0.021149015 | *Jun* | 0.637169694 | 1.07E-08 |
| *Abcc4* | 0.742458593 | 0.01619191 | *P4ha1* | 0.687317038 | 0.004918067 | *Smchd1* | 0.636663929 | 0.003423529 |
| *Cbfb* | 0.742458593 | 0.016328352 | *Herpud1* | 0.686605358 | 0.028546938 | *Med13* | 0.635543389 | 0.015156444 |
| *Desi1* | 0.742458593 | 0.017219573 | *Alk* | 0.685875064 | 0.034011409 | *Lin7a* | 0.635543389 | 0.022553154 |
| *Emsy* | 0.742458593 | 0.024107354 | *Rngtt* | 0.685875064 | 0.003981781 | *Nelfa* | 0.635543389 | 0.054859288 |
| *Prpf8* | 0.742458593 | 0.02796788 | *Cdc42* | 0.684511243 | 0.001544363 | *Pias1* | 0.633934136 | 0.035174794 |
| *Pcif1* | 0.742458593 | 0.028293183 | *Med15* | 0.681058048 | 0.01889883 | *Akirin2* | 0.632834101 | 0.039692882 |
| *Rerg* | 0.742458593 | 0.031788588 | *Med27* | 0.681058048 | 0.021904482 | *Itpk1* | 0.632275675 | 0.020234304 |
| *Cdk2ap1* | 0.742458593 | 0.032188865 | *Mettl9* | 0.681058048 | 0.027543599 | *Irs2* | 0.632034603 | 0.000650828 |
| *Rab33b* | 0.742458593 | 0.032595789 | *Vma21* | 0.681058048 | 0.039388875 | *Ptpro* | 0.630950277 | 0.0006254 |
| *Whamm* | 0.742458593 | 0.032654529 | *Zfp445* | 0.678328255 | 0.019294229 | *Ndufaf8* | 0.629983863 | 0.022500186 |
| *Ipo8* | 0.742458593 | 0.035550871 | *Phb2* | 0.678328255 | 0.051429434 | *Zup1* | 0.629983863 | 0.02816942 |
| *Rfx2* | 0.742458593 | 0.036192167 | *Asic2* | 0.67802672 | 0.00044274 | *Clip2* | 0.629983863 | 0.030778663 |
| *G6pc3* | 0.742458593 | 0.038763245 | *Pcgf5* | 0.677363564 | 0.001244777 | *Ptch1* | 0.629983863 | 0.053227757 |
| *Arl6ip5* | 0.742458593 | 0.040684382 | *Tiparp* | 0.677363564 | 0.01261526 | *Ncl* | 0.626981375 | 0.000876395 |
| *Brsk1* | 0.742458593 | 0.052910929 | *Mirg* | 0.677167132 | 0.001794993 | *Dctn4* | 0.626981375 | 0.015250072 |
| *Klhdc2* | 0.742458593 | 0.00016643 | *Lingo2* | 0.676870251 | 0.001310481 | *Cdk5rap2* | 0.626981375 | 0.01800772 |
| *Mvp* | 0.742458593 | 0.021928385 | *Nek9* | 0.676870251 | 0.027667716 | *Tbce* | 0.626981375 | 0.018231125 |
| *Gm26759* | 0.742458593 | 0.037414773 | *mt-Nd4l* | 0.676116098 | 0.000358117 | *Strn* | 0.626981375 | 0.045959759 |
| *Bcan* | 0.742458593 | 0.039524815 | *Snx30* | 0.675344397 | 0.009654495 | *Ssr1* | 0.623405921 | 0.004167022 |
| *Rhbdd2* | 0.742458593 | 0.048915219 | *Efemp1* | 0.67428709 | 0.008268268 | *Rpl22l1* | 0.621349507 | 4.18E-05 |
| *Gmcl1* | 0.742458593 | 0.054251337 | *Mapk14* | 0.67428709 | 0.020471413 | *Gpr180* | 0.621152296 | 0.029343537 |
| *Alg13* | 0.7273517 | 0.017963494 | *Abce1* | 0.673745842 | 0.037728053 | *Pop5* | 0.620468068 | 0.041944199 |
| *Rps27* | 0.726017445 | 5.37E-08 | *Dync1li1* | 0.670308807 | 0.043181207 | *Pi4ka* | 0.619974586 | 0.001673376 |
| *Nfkbiz* | 0.723599565 | 0.009446656 | *Ctsb* | 0.666509739 | 0.024142797 | *Tppp3* | 0.619601845 | 0.032951781 |
| *Pip5k1b* | 0.722829786 | 0.015110331 | *Anks1* | 0.665837311 | 0.006159907 | *Cct8* | 0.61692771 | 0.048786756 |
| *Fkbp3* | 0.719374979 | 0.011905056 | *Ap2b1* | 0.665290732 | 0.002059961 | *Cand1* | 0.61692771 | 0.014444525 |
| *Aebp1* | 0.718413119 | 0.047036629 | *Stk32a* | 0.664779229 | 2.33E-05 | *Mrps21* | 0.615346675 | 0.007351558 |
| *Syt4* | 0.715364932 | 0.000170961 | *Atp5e* | 0.663288379 | 0.000486491 | *Fgfr2* | 0.615079286 | 0.051355226 |
| *Akap8* | 0.714977856 | 0.035529745 | *Anapc4* | 0.663024125 | 0.039852517 | *Brd4* | 0.614703045 | 0.001073988 |
| *Lmna* | 0.714624384 | 4.09E-07 | *Ascc1* | 0.662288244 | 0.018398141 | *Ndufc2* | 0.612823312 | 0.006486834 |
| *Lars* | 0.714444216 | 0.024333712 | *Lmbr1* | 0.661538597 | 0.016273237 | *Arl5b* | 0.612398052 | 0.04178462 |
| *Srbd1* | 0.712084943 | 0.020143755 | *Vti1a* | 0.659203089 | 0.047647803 | *Nup153* | 0.612398052 | 0.044759514 |
| *Rimkla* | 0.712084943 | 0.033353839 | *Mast4* | 0.655746959 | 0.000161252 | *Cibar1* | 0.610008297 | 0.019681059 |
| *Rabgef1* | 0.711431697 | 0.053894141 | *Mtfr1l* | 0.654995751 | 0.050928001 | *Fosb* | 0.609670863 | 4.26E-10 |
| *Hyou1* | 0.710037115 | 0.051590805 | *Ddah1* | 0.654995751 | 0.001771706 | *Wdr47* | 0.608157501 | 0.012685446 |
| *Epb41* | 0.709291729 | 0.009980381 | *Camkmt* | 0.654995751 | 0.006973816 | *Tpp2* | 0.604955069 | 0.005508276 |
| *Rnf6* | 0.709291729 | 0.037576114 | *Pdap1* | 0.654995751 | 0.010569568 | *Ndufb7* | 0.604955069 | 0.013152573 |
| *Gpc4* | 0.708775466 | 0.000217287 | *Csnk1g3* | 0.654995751 | 0.021655049 | *Atp9a* | 0.604955069 | 0.023631807 |

| **Supplemental Table S8E DEGS LAC3 Cluster; VCD/Vehicle.** Maroon=upregulated; Blue=downregulated | | | | | | | | |
| --- | --- | --- | --- | --- | --- | --- | --- | --- |
| **gene** | **avg_log2FC** | **p_val** | **gene** | **avg_log2FC** | **p_val** | **gene** | **avg_log2FC** | **p_val** |
| *Prkca* | 0.604955069 | 0.037102574 | *Ccdc167* | -0.735588704 | 0.051899777 | *Ankrd49* | -1.132010525 | 0.052381312 |
| *Aff3* | 0.604955069 | 0.04605782 | *Ppp2r2d* | -0.737534349 | 0.051184344 | *Rab8b* | -1.140184457 | 0.002988366 |
| *Mast2* | 0.604955069 | 0.051552215 | *Mindy2* | -0.742968235 | 0.026105432 | *Rfc2* | -1.145066678 | 0.028147871 |
| *Kctd8* | 0.603674002 | 0.001458817 | *Ddx1* | -0.752306099 | 0.052977783 | *Zcchc8* | -1.145066678 | 0.035697316 |
| *Gtf2f2* | 0.602280934 | 0.008290526 | *Pdzd2* | -0.769440446 | 0.017672559 | *Ccdc60* | -1.150626204 | 0.040094719 |
| *Nfx1* | 0.601102743 | 0.020428631 | *Tomm22* | -0.77211458 | 0.042851317 | *Fam199x* | -1.164432003 | 0.030105323 |
| *Rps28* | 0.600797443 | 6.73E-07 | *Ndufs8* | -0.783610219 | 0.010815245 | *Tenm3* | -1.18715208 | 0.005847703 |
| *Atp5md* | 0.600014328 | 0.000586709 | *Rbck1* | -0.788056124 | 0.050628474 | *Triqk* | -1.221015531 | 0.013320219 |
| *Anapc13* | 0.599500639 | 0.053670412 | *Tatdn1* | -0.790973608 | 0.042143591 | *Serinc3* | -1.227167758 | 0.00308868 |
| *Tgoln1* | 0.599263957 | 0.003552631 | *Atg7* | -0.810082431 | 0.045276501 | *Gm3693* | -1.257541407 | 0.013191409 |
| *Gtdc1* | 0.598068683 | 0.046246765 | *Fbrs* | -0.812130259 | 0.025752912 | *Kpna6* | -1.257541407 | 0.013718974 |
| *Gm57212* | 0.597260677 | 0.006051003 | *Tapt1* | -0.815536861 | 0.020970795 | *Zfp780b* | -1.257541407 | 0.025489532 |
| *Pi4k2a* | 0.595617204 | 0.033714113 | *Eid2* | -0.818256362 | 0.022544631 | *Med7* | -1.257541407 | 0.030187278 |
| *Ube2d2a* | 0.595116876 | 0.015673778 | *Ddhd2* | -0.821442293 | 0.036212808 | *4930555F03Rik* | -1.257541407 | 0.039701504 |
| *Prkar2a* | 0.593080968 | 0.01158644 | *Oxa1l* | -0.842503908 | 0.031508145 | *Taf7* | -1.285022144 | 0.002764296 |
| *Upf3b* | 0.590455499 | 0.014259443 | *Brms1* | -0.842503908 | 0.05299617 | *2810403D21Rik* | -1.287288751 | 0.005826946 |
| *Trp53bp2* | 0.590455499 | 0.046914175 | *Amz2* | -0.842503908 | 0.022849893 | *Trim24* | -1.293165317 | 0.013109935 |
| *Brd2* | 0.589107816 | 0.026935274 | *Polr3c* | -0.842503908 | 0.035086482 | *Sphk2* | -1.297069772 | 0.032757829 |
| *Ergic1* | 0.588653256 | 0.005893754 | *Pepd* | -0.864198979 | 0.029511029 | *Smpdl3a* | -1.316435097 | 0.051603676 |
| *Tspyl1* | 0.585733089 | 0.017726721 | *Nova2* | -0.86897612 | 0.013945485 | *Gk* | -1.327930735 | 0.04463406 |
| *Yipf3* | 0.585733089 | 0.035219476 | *Bcl7a* | -0.872251252 | 0.025240451 | *Tsga10* | -1.357077081 | 0.017261917 |
| *Faf2* | 0.585733089 | 0.04093117 | *Gprin1* | -0.882032272 | 0.050089095 | *Gnpda2* | -1.367965397 | 0.000159315 |
| *Htt* | 0.584917316 | 0.034894471 | *Mir99ahg* | -0.887591798 | 0.019264827 | *Eif3b* | -1.373018625 | 0.00441805 |
| *Trip11* | 0.58402923 | 0.018484923 | *Parp6* | -0.905239664 | 0.046452583 | *Mrps27* | -1.373018625 | 0.007898103 |
| *Cpeb4* | 0.582587256 | 0.003039879 | *Ago1* | -0.909618104 | 0.02836529 | *Mrps23* | -1.373018625 | 0.007911958 |
| *Hnrnpu* | 0.58199392 | 0.001959672 | *Tmem192* | -0.909618104 | 0.04519253 | *Zfp319* | -1.373018625 | 0.036920603 |
| *Sec24a* | 0.581303801 | 0.031718433 | *Erg28* | -0.914653694 | 0.035179689 | *Arpc1b* | -1.373018625 | 0.051742204 |
| *Tomm7* | 0.58099517 | 0.008683276 | *Supt20* | -0.924966068 | 0.014509784 | *Map3k13* | -1.386824424 | 0.037563857 |
| *Kcnab1* | 0.580707523 | 0.01437837 | *Acot13* | -0.929966749 | 0.046973205 | *Rcbtb2* | -1.395044931 | 0.007726697 |
|  |  |  | *Pola1* | -0.942039582 | 0.034965872 | *Zcchc3* | -1.395044931 | 0.033459262 |
| *Csmd3* | -0.593824795 | 0.001320398 | *Trim66* | -0.949419112 | 0.043263086 | *Srr* | -1.415082684 | 0.003759899 |
| *Ndufs3* | -0.59739141 | 0.033113033 | *Erh* | -0.957981126 | 0.01531734 | *Ndufa9* | -1.427466409 | 0.029862939 |
| *Usp7* | -0.602676894 | 0.050698066 | *Gm29684* | -0.96803479 | 0.0465892 | *Gm16740* | -1.427466409 | 0.036531453 |
| *Mrpl32* | -0.611178362 | 0.048363087 | *Gucy2f* | -0.980477442 | 0.002693094 | *Akna* | -1.427466409 | 0.049212715 |
| *Impg1* | -0.620111487 | 0.041941732 | *Wwp1* | -0.985461862 | 0.019573267 | *2610044O15Rik8* | -1.427466409 | 0.049212715 |
| *Gm28905* | -0.625273192 | 0.001242431 | *Crb1* | -0.994507002 | 0.039695067 | *Efcab7* | -1.450186485 | 0.025902828 |
| *Ppfibp1* | -0.626775217 | 0.039204718 | *Lpcat3* | -1.004775337 | 0.02667481 | *Exoc7* | -1.459175269 | 0.033316745 |
| *Cuedc1* | -0.630999803 | 0.016404619 | *Mrap2* | -1.007563154 | 0.022781452 | *A930006I01Rik* | -1.459175269 | 0.044447864 |
| *Rc3h1* | -0.636053031 | 0.046964275 | *Frg1* | -1.016533308 | 0.034257786 | *Snrnp27* | -1.479933829 | 0.004289787 |
| *Cdr1os* | -0.640870047 | 0.016047826 | *Tpcn1* | -1.023076154 | 0.026504903 | *Cyp2j6* | -1.479933829 | 0.052330115 |
| *Vat1* | -0.648331361 | 0.026507439 | *Prelid3b* | -1.023076154 | 0.046092833 | *Dnaaf5* | -1.479933829 | 0.052330115 |
| *Sez6l* | -0.666346953 | 0.032994002 | *Ifi35* | -1.041812716 | 0.023802273 | *Armt1* | -1.479933829 | 0.052330115 |
| *Cetn3* | -0.672578907 | 0.023098319 | *Dmtn* | -1.046037302 | 0.030732166 | *Scn10a* | -1.505468921 | 0.010798018 |
| *Sash1* | -0.672578907 | 0.034891081 | *9530077C05Rik* | -1.06489633 | 0.006832222 | *Zkscan17* | -1.579469502 | 0.026856014 |
| *Mccc2* | -0.672578907 | 0.044590298 | *Bhlhe41* | -1.06489633 | 0.04574634 | *Jph4* | -1.579469502 | 0.027906763 |
| *Atg10* | -0.675267994 | 0.003326711 | *Apbb2* | -1.076969162 | 0.014497172 | *Hars2* | -1.579469502 | 0.036266778 |
| *Psmd7* | -0.676493957 | 0.028905611 | *Commd4* | -1.083512008 | 0.019065858 | *Pdp2* | -1.579469502 | 0.038030724 |
| *Macrod2* | -0.684962631 | 0.015357381 | *Knop1* | -1.083512008 | 0.038359642 | *Gm45341* | -1.579469502 | 0.048879747 |
| *C130073E24Rik* | -0.685402947 | 0.045894719 | *Msrb3* | -1.083512008 | 0.045597378 | *Gpank1* | -1.579469502 | 0.052189676 |
| *Olfm4* | -0.693640522 | 0.045655471 | *Zfp219* | -1.094042675 | 0.023363278 | *Gm35188* | -1.636053031 | 0.033174572 |
| *Marcks* | -0.69566252 | 0.052501177 | *Commd5* | -1.094042675 | 0.045515405 | *Arsa* | -1.672578907 | 0.01348228 |
| *Gm49542* | -0.70232625 | 0.050797918 | *Tafazzin* | -1.097076735 | 0.002187877 | *Gm56596* | -1.672578907 | 0.027618353 |
| *Sema3a* | -0.703943078 | 0.000664267 | *Scn2a* | -1.105538314 | 0.020391664 | *Zfp955a* | -1.672578907 | 0.027618353 |
| *Slc35f4* | -0.716973026 | 6.96E-06 | *Rbks* | -1.132010525 | 0.034191829 | *Gm16251* | -1.672578907 | 0.052058565 |
| *Dnm1l* | -0.725690243 | 0.049616634 | *Marchf11* | -1.132010525 | 0.034191829 | *Rad51c* | -1.672578907 | 0.052058565 |
| *Gm20125* | -0.726094416 | 0.013592195 | *Fbxw10* | -1.132010525 | 0.049922767 | *Zkscan16* | -1.690500815 | 0.018405198 |
| *Fnta* | -0.733979451 | 0.044269339 | *2610206C17Rik* | -1.132010525 | 0.051894175 | *Gm15535* | -1.716973026 | 0.019711317 |

| **Supplemental Table S8F DEGS LAC3 Cluster; VCD/Vehicle.** Maroon=upregulated; Blue=downregulated | | | | | | | | |
| --- | --- | --- | --- | --- | --- | --- | --- | --- |
| **gene** | **avg_log2FC** | **p_val** | **gene** | **avg_log2FC** | **p_val** | **gene** | **avg_log2FC** | **p_val** |
| *Aldh2* | -1.735588704 | 0.003624382 |  |  |  |  |  |  |
| *Coq8a* | -1.760041748 | 0.027539209 |  |  |  |  |  |  |
| *Ttc22* | -1.760041748 | 0.037828459 |  |  |  |  |  |  |
| *Ccdc163* | -1.760041748 | 0.051927517 |  |  |  |  |  |  |
| *Rnf32* | -1.77211458 | 0.00271055 |  |  |  |  |  |  |
| *Gm16835* | -1.781103364 | 0.010680998 |  |  |  |  |  |  |
| *Lgals1* | -1.842503908 | 0.018405194 |  |  |  |  |  |  |
| *Gm16093* | -1.842503908 | 0.019984706 |  |  |  |  |  |  |
| *Cfap276* | -1.842503908 | 0.036373597 |  |  |  |  |  |  |
| *4933406C10Rik* | -1.842503908 | 0.036373597 |  |  |  |  |  |  |
| *Nudt7* | -1.842503908 | 0.051053087 |  |  |  |  |  |  |
| *Hus1* | -1.842503908 | 0.051053087 |  |  |  |  |  |  |
| *Nek10* | -1.842503908 | 0.051053087 |  |  |  |  |  |  |
| *Gm17473* | -1.842503908 | 0.051053087 |  |  |  |  |  |  |
| *Gm50462* | -1.842503908 | 0.051053087 |  |  |  |  |  |  |
| *Mycbpap* | -1.889809623 | 0.007527402 |  |  |  |  |  |  |
| *Mex3d* | -1.92050642 | 0.014494365 |  |  |  |  |  |  |
| *Mtmr14* | -1.92050642 | 0.019932394 |  |  |  |  |  |  |
| *Nudt13* | -1.92050642 | 0.019932394 |  |  |  |  |  |  |
| *E330013P04Rik* | -1.957981126 | 0.005713615 |  |  |  |  |  |  |
| *Hnmt* | -1.957981126 | 0.036315408 |  |  |  |  |  |  |
| *Boc* | -1.957981126 | 0.036315408 |  |  |  |  |  |  |
| *Lrriq1* | -1.994507002 | 0.027467955 |  |  |  |  |  |  |
| *Gan* | -2.06489633 | 0.025877866 |  |  |  |  |  |  |
| *Donson* | -2.06489633 | 0.036261793 |  |  |  |  |  |  |
| *Prrg1* | -2.164432003 | 0.000631037 |  |  |  |  |  |  |
| *Zfp952* | -2.164432003 | 0.013218496 |  |  |  |  |  |  |
| *Anxa1* | -2.164432003 | 0.036208082 |  |  |  |  |  |  |
| *Rab13* | -2.257541407 | 0.040270146 |  |  |  |  |  |  |
| *Cdc7* | -2.257541407 | 0.040270146 |  |  |  |  |  |  |
| *Ccdc122* | -2.257541407 | 0.040270146 |  |  |  |  |  |  |
| *Onecut2* | -2.257541407 | 0.040270146 |  |  |  |  |  |  |
| *Cenpt* | -2.427466409 | 0.028249536 |  |  |  |  |  |  |
| *Tmem177* | -2.427466409 | 0.0402691 |  |  |  |  |  |  |
| *Vcan* | -2.427466409 | 0.0402691 |  |  |  |  |  |  |
| *C130060C02Rik* | -2.427466409 | 0.0402691 |  |  |  |  |  |  |
| *Xirp2* | -2.505468921 | 0.013136123 |  |  |  |  |  |  |
| *Trp53rka* | -2.579469502 | 0.019903721 |  |  |  |  |  |  |
| *C1qtnf12* | -2.579469502 | 0.019903721 |  |  |  |  |  |  |
| *Trpt1* | -2.579469502 | 0.019903721 |  |  |  |  |  |  |
| *4930525G20Rik* | -2.579469502 | 0.028248378 |  |  |  |  |  |  |
| *Efemp2* | -2.579469502 | 0.028248378 |  |  |  |  |  |  |
| *Cyria* | -2.579469502 | 0.040269548 |  |  |  |  |  |  |
| *Gm16894* | -2.64985883 | 0.003463836 |  |  |  |  |  |  |
| *4732491K20Rik* | -2.716973026 | 0.019902512 |  |  |  |  |  |  |
| *Pcdh18* | -2.716973026 | 0.028248812 |  |  |  |  |  |  |
| *Blm* | -2.842503908 | 0.014070662 |  |  |  |  |  |  |
| *Gm57243* | -2.842503908 | 0.019902915 |  |  |  |  |  |  |
| *Rangrf* | -2.957981126 | 0.019903217 |  |  |  |  |  |  |
| *Gm5577* | -3.06489633 | 0.005045873 |  |  |  |  |  |  |
| *Tspyl3* | -3.06489633 | 0.009975403 |  |  |  |  |  |  |
| *Gm2516* | -3.257541407 | 0.002567698 |  |  |  |  |  |  |
| *Thnsl1* | -3.427466409 | 0.002567044 |  |  |  |  |  |  |
|  |  |  |  |  |  |  |  |  |
|  |  |  |  |  |  |  |  |  |
|  |  |  |  |  |  |  |  |  |
|  |  |  |  |  |  |  |  |  |

| **Supplemental Table S9A DEGS LAC4 Cluster; VCD/Vehicle.** Maroon=upregulated; | | | | | | | | |
| --- | --- | --- | --- | --- | --- | --- | --- | --- |
| **gene** | **avg_log2FC** | **p_val** | **gene** | **avg_log2FC** | **p_val** | **gene** | **avg_log2FC** | **p_val** |
| *Tent5c* | 4.179323699 | 0.023329303 | *Fastkd2* | 2.956931278 | 0.023329303 | *Jagn1* | 2.956931278 | 0.023329303 |
| *Cd74* | 4.179323699 | 0.023329303 | *Zranb3* | 2.956931278 | 0.023329303 | *Tapbpl* | 2.956931278 | 0.023329303 |
| *Hipk1* | 3.956931278 | 0.023329303 | *Dcaf6* | 2.956931278 | 0.023329303 | *Plekha5* | 2.956931278 | 0.023329303 |
| *Fdps* | 3.693896872 | 0.001037805 | *Dusp12* | 2.956931278 | 0.023329303 | *Fgfr1op2* | 2.956931278 | 0.023329303 |
| *M6pr* | 3.693896872 | 0.001037805 | *Ubac1* | 2.956931278 | 0.023329303 | *Ppp6r1* | 2.956931278 | 0.023329303 |
| *Rmnd5b* | 3.693896872 | 0.001037805 | *Lhx3* | 2.956931278 | 0.023329303 | *Zfp329* | 2.956931278 | 0.023329303 |
| *Uqcc4* | 3.693896872 | 0.001037805 | *Brd3os* | 2.956931278 | 0.023329303 | *Fxyd5* | 2.956931278 | 0.023329303 |
| *Taok3* | 3.693896872 | 0.004925089 | *Urm1* | 2.956931278 | 0.023329303 | *Pdcd2l* | 2.956931278 | 0.023329303 |
| *Wapl* | 3.693896872 | 0.004925089 | *Gle1* | 2.956931278 | 0.023329303 | *Stim1* | 2.956931278 | 0.023329303 |
| *Mbp* | 3.693896872 | 0.004925089 | *Zdhhc12* | 2.956931278 | 0.023329303 | *Tmem219* | 2.956931278 | 0.023329303 |
| *Rere* | 3.693896872 | 0.023329303 | *Scrn3* | 2.956931278 | 0.023329303 | *Timm44* | 2.956931278 | 0.023329303 |
| *Dennd5b* | 3.693896872 | 0.023329303 | *Mir670hg* | 2.956931278 | 0.023329303 | *Mcf2l* | 2.956931278 | 0.023329303 |
| *Srgn* | 3.693896872 | 0.023329303 | *Bahd1* | 2.956931278 | 0.023329303 | *Ank1* | 2.956931278 | 0.023329303 |
| *Ripor2* | 3.693896872 | 0.023329303 | *Sirpa* | 2.956931278 | 0.023329303 | *Plekha2* | 2.956931278 | 0.023329303 |
| *Enox2* | 3.693896872 | 0.023329303 | *Pank2* | 2.956931278 | 0.023329303 | *Rbmxl1* | 2.956931278 | 0.023329303 |
| *Rc3h2* | 3.371968777 | 0.004929144 | *Zfp442* | 2.956931278 | 0.023329303 | *Prkaca* | 2.956931278 | 0.023329303 |
| *Dnajc24* | 3.371968777 | 0.004929144 | *Zcchc3* | 2.956931278 | 0.023329303 | *Katnb1* | 2.956931278 | 0.023329303 |
| *Aven* | 3.371968777 | 0.004929144 | *Pdrg1* | 2.956931278 | 0.023329303 | *Tk2* | 2.956931278 | 0.023329303 |
| *Magoh* | 3.371968777 | 0.004929144 | *Commd7* | 2.956931278 | 0.023329303 | *Cmtm4* | 2.956931278 | 0.023329303 |
| *Mmp23* | 3.371968777 | 0.004929144 | *Fam210b* | 2.956931278 | 0.023329303 | *Nol3* | 2.956931278 | 0.023329303 |
| *Gm56999* | 3.371968777 | 0.004929144 | *Stx16* | 2.956931278 | 0.023329303 | *Mlycd* | 2.956931278 | 0.023329303 |
| *Ppp1r37* | 3.371968777 | 0.004929144 | *Cables2* | 2.956931278 | 0.023329303 | *Chordc1* | 2.956931278 | 0.023329303 |
| *Sergef* | 3.371968777 | 0.004929144 | *Mtfr1* | 2.956931278 | 0.023329303 | *Fli1* | 2.956931278 | 0.023329303 |
| *Picalm* | 3.371968777 | 0.004929144 | *Larp1b* | 2.956931278 | 0.023329303 | *Ppil4* | 2.956931278 | 0.023329303 |
| *Bag3* | 3.371968777 | 0.004929144 | *Gm16740* | 2.956931278 | 0.023329303 | *Rev3l* | 2.956931278 | 0.023329303 |
| *Taldo1* | 3.371968777 | 0.004929144 | *Ensa* | 2.956931278 | 0.023329303 | *Eif4ebp2* | 2.956931278 | 0.023329303 |
| *4933406B17Rik* | 3.371968777 | 0.004929144 | *Wdr3* | 2.956931278 | 0.023329303 | *Hnrnph3* | 2.956931278 | 0.023329303 |
| *Fdx1* | 3.371968777 | 0.004929144 | *Rbm15* | 2.956931278 | 0.023329303 | *Zwint* | 2.956931278 | 0.023329303 |
| *Usp3* | 3.371968777 | 0.004929144 | *Prpf38b* | 2.956931278 | 0.023329303 | *Rrp1* | 2.956931278 | 0.023329303 |
| *D830035M03Rik* | 3.371968777 | 0.004929144 | *Larp7* | 2.956931278 | 0.023329303 | *Zfp938* | 2.956931278 | 0.023329303 |
| *Asf1a* | 3.371968777 | 0.004929144 | *Casp6* | 2.956931278 | 0.023329303 | *Mterf2* | 2.956931278 | 0.023329303 |
| *Ube2g2* | 3.371968777 | 0.004929144 | *Hs2st1* | 2.956931278 | 0.023329303 | *Llph* | 2.956931278 | 0.023329303 |
| *Ccng1* | 3.371968777 | 0.004929144 | *Asph* | 2.956931278 | 0.023329303 | *Rxylt1* | 2.956931278 | 0.023329303 |
| *Sap30l* | 3.371968777 | 0.004929144 | *Zfp292* | 2.956931278 | 0.023329303 | *Mars1* | 2.956931278 | 0.023329303 |
| *Arl4a* | 3.371968777 | 0.004929144 | *Rnf220* | 2.956931278 | 0.023329303 | *Snhg15* | 2.956931278 | 0.023329303 |
| *Wrnip1* | 3.371968777 | 0.004929144 | *Ppih* | 2.956931278 | 0.023329303 | *9130230N09Rik* | 2.956931278 | 0.023329303 |
| *Supt16* | 3.371968777 | 0.004929144 | *Srsf10* | 2.956931278 | 0.023329303 | *Zswim7* | 2.956931278 | 0.023329303 |
| *Tmem39a* | 3.371968777 | 0.004929144 | *Kdm1a* | 2.956931278 | 0.023329303 | *Pigl* | 2.956931278 | 0.023329303 |
| *Ptprs* | 3.371968777 | 0.004929144 | *Capzb* | 2.956931278 | 0.023329303 | *Senp3* | 2.956931278 | 0.023329303 |
| *Ralbp1* | 3.371968777 | 0.004929144 | *Camta1* | 2.956931278 | 0.023329303 | *Fbxl20* | 2.956931278 | 0.023329303 |
| *Ptpn2* | 3.371968777 | 0.004929144 | *Fam133b* | 2.956931278 | 0.023329303 | *Krt10* | 2.956931278 | 0.023329303 |
| *Emd* | 3.371968777 | 0.004929144 | *Prkag2* | 2.956931278 | 0.023329303 | *Cyb561* | 2.956931278 | 0.023329303 |
| *Tmem29* | 3.371968777 | 0.004929144 | *Hadhb* | 2.956931278 | 0.023329303 | *Nat9* | 2.956931278 | 0.023329303 |
| *Tram2* | 3.371968777 | 0.023329303 | *Abhd1* | 2.956931278 | 0.023329303 | *Galk1* | 2.956931278 | 0.023329303 |
| *1700025G04Rik* | 3.371968777 | 0.023329303 | *Gm42984* | 2.956931278 | 0.023329303 | *Ubald2* | 2.956931278 | 0.023329303 |
| *Gm20186* | 3.371968777 | 0.023329303 | *Slain2* | 2.956931278 | 0.023329303 | *Kidins220* | 2.956931278 | 0.023329303 |
| *Akr1b3* | 3.371968777 | 0.023329303 | *Mob1b* | 2.956931278 | 0.023329303 | *Sec23a* | 2.956931278 | 0.023329303 |
| *Rab21* | 3.371968777 | 0.023329303 | *Bri3bp* | 2.956931278 | 0.023329303 | *Arid4a* | 2.956931278 | 0.023329303 |
| *Scfd1* | 3.371968777 | 0.023329303 | *Gtf2ird2* | 2.956931278 | 0.023329303 | *Max* | 2.956931278 | 0.023329303 |
| *Psmc1* | 3.371968777 | 0.023329303 | *Tmem184a* | 2.956931278 | 0.023329303 | *Ahsa1* | 2.956931278 | 0.023329303 |
| *Zfyve21* | 3.371968777 | 0.023329303 | *Snd1* | 2.956931278 | 0.023329303 | *Ppp4r3a* | 2.956931278 | 0.023329303 |
| *Npas4* | 3.371968777 | 0.023329303 | *Agbl3* | 2.956931278 | 0.023329303 | *Tmem179* | 2.956931278 | 0.023329303 |
| *Xbp1* | 3.179323699 | 0.001563817 | *Ccdc126* | 2.956931278 | 0.023329303 | *Gng4* | 2.956931278 | 0.023329303 |
| *Ly6a* | 3.179323699 | 0.006559231 | *E230016M11Rik* | 2.956931278 | 0.023329303 | *Agtpbp1* | 2.956931278 | 0.023329303 |
| *Dstn* | 2.956931278 | 0.00166726 | *Isy1* | 2.956931278 | 0.023329303 | *Clptm1l* | 2.956931278 | 0.023329303 |
| *Rbm25* | 2.956931278 | 0.007153623 | *Foxp1* | 2.956931278 | 0.023329303 | *Atg10* | 2.956931278 | 0.023329303 |
| *Coa5* | 2.956931278 | 0.023329303 | *Eif4e3* | 2.956931278 | 0.023329303 | *Wdr41* | 2.956931278 | 0.023329303 |

| **Supplemental Table S9B DEGS LAC4 Cluster; VCD/Vehicle.** Maroon=upregulated; | | | | | | | | |
| --- | --- | --- | --- | --- | --- | --- | --- | --- |
| **gene** | **avg_log2FC** | **p_val** | **gene** | **avg_log2FC** | **p_val** | **gene** | **avg_log2FC** | **p_val** |
| *Srek1ip1* | 2.956931278 | 0.023329303 | *Tm9sf4* | 2.371968777 | 0.032619731 | *Psmb3* | 2.050040682 | 0.037805494 |
| *Ube2e2* | 2.956931278 | 0.023329303 | *BC029722* | 2.371968777 | 0.032619731 | *Top1* | 1.956931278 | 0.008799984 |
| *Ghitm* | 2.956931278 | 0.023329303 | *Pkig* | 2.371968777 | 0.032619731 | *Mpc2* | 1.956931278 | 0.021263535 |
| *Dhrs1* | 2.956931278 | 0.023329303 | *Nmd3* | 2.371968777 | 0.032619731 | *Hnrnpa3* | 1.956931278 | 0.021263535 |
| *Sdr39u1* | 2.956931278 | 0.023329303 | *Mcl1* | 2.371968777 | 0.032619731 | *Edem2* | 1.956931278 | 0.021263535 |
| *Mbnl2* | 2.956931278 | 0.023329303 | *Runx1t1* | 2.371968777 | 0.032619731 | *Coq7* | 1.956931278 | 0.021263535 |
| *Arc* | 2.956931278 | 0.023329303 | *Smim1* | 2.371968777 | 0.032619731 | *Sf3b6* | 1.956931278 | 0.021263535 |
| *C030006K11Rik* | 2.956931278 | 0.023329303 | *Gatad1* | 2.371968777 | 0.032619731 | *Mtch1* | 1.956931278 | 0.021263535 |
| *Myh9* | 2.956931278 | 0.023329303 | *Ccz1* | 2.371968777 | 0.032619731 | *H2aj* | 1.956931278 | 0.023595192 |
| *Dnal4* | 2.956931278 | 0.023329303 | *Immt* | 2.371968777 | 0.032619731 | *Ap2s1* | 1.956931278 | 0.027356172 |
| *Tob2* | 2.956931278 | 0.023329303 | *Vamp8* | 2.371968777 | 0.032619731 | *Ncl* | 1.956931278 | 0.030431204 |
| *Cpne8* | 2.956931278 | 0.023329303 | *Tomm40* | 2.371968777 | 0.032619731 | *Atp5j2* | 1.88654195 | 0.004967775 |
| *Yaf2* | 2.956931278 | 0.023329303 | *Fbl* | 2.371968777 | 0.032619731 | *Uqcrc2* | 1.857395605 | 0.01498798 |
| *Txndc11* | 2.956931278 | 0.023329303 | *Bola2* | 2.371968777 | 0.032619731 | *Mcts1* | 1.857395605 | 0.042694459 |
| *Thap7* | 2.956931278 | 0.023329303 | *Micu3* | 2.371968777 | 0.032619731 | *Ndufa4* | 1.841454061 | 0.000456054 |
| *Timmdc1* | 2.956931278 | 0.023329303 | *Mrpl4* | 2.371968777 | 0.032619731 | *Fkbp1a* | 1.831400396 | 0.00828786 |
| *Mrps6* | 2.956931278 | 0.023329303 | *Pts* | 2.371968777 | 0.032619731 | *Cltb* | 1.787006277 | 0.027356172 |
| *Psmb8* | 2.956931278 | 0.023329303 | *Hdac2* | 2.371968777 | 0.032619731 | *Txnl1* | 1.787006277 | 0.027356172 |
| *Agpat1* | 2.956931278 | 0.023329303 | *Ahsa2* | 2.371968777 | 0.032619731 | *Prkacb* | 1.787006277 | 0.037424686 |
| *2610029K11Rik* | 2.956931278 | 0.023329303 | *Rab10* | 2.371968777 | 0.032619731 | *Srsf7* | 1.787006277 | 0.037424686 |
| *Riok3* | 2.956931278 | 0.023329303 | *Snapc1* | 2.371968777 | 0.032619731 | *Cuedc2* | 1.787006277 | 0.037424686 |
| *Impact* | 2.956931278 | 0.023329303 | *Brix1* | 2.371968777 | 0.032619731 | *Elob* | 1.734538857 | 0.044027728 |
| *Wdr33* | 2.956931278 | 0.023329303 | *Cbx6* | 2.371968777 | 0.032619731 | *Slc25a5* | 1.734538857 | 0.044027728 |
| *Hsbp1l1* | 2.956931278 | 0.023329303 | *Pacsin2* | 2.371968777 | 0.032619731 | *Psma7* | 1.693896872 | 0.006612818 |
| *Stx5a* | 2.956931278 | 0.023329303 | *Smim22* | 2.371968777 | 0.032619731 | *Prdx6* | 1.693896872 | 0.032814379 |
| *Fads1* | 2.956931278 | 0.023329303 | *Gnptg* | 2.371968777 | 0.032619731 | *Cox17* | 1.661475395 | 0.036713135 |
| *Fen1* | 2.956931278 | 0.023329303 | *Tsr3* | 2.371968777 | 0.032619731 | *B9d1* | 1.635003183 | 0.025120458 |
| *Shtn1* | 2.956931278 | 0.023329303 | *Csnk2b* | 2.371968777 | 0.032619731 | *Cmpk1* | 1.635003183 | 0.048997238 |
| *Hmgb3* | 2.956931278 | 0.023329303 | *Rsph9* | 2.371968777 | 0.032619731 | *Dcun1d5* | 1.635003183 | 0.048997238 |
| *Mpp1* | 2.956931278 | 0.023329303 | *Naa12* | 2.371968777 | 0.032619731 | *Snapc5* | 1.635003183 | 0.048997238 |
| *Pja1* | 2.956931278 | 0.023329303 | *Rce1* | 2.371968777 | 0.032619731 | *Srsf3* | 1.635003183 | 0.048997238 |
| *Trappc2* | 2.956931278 | 0.023329303 | *Mrpl16* | 2.371968777 | 0.032619731 | *Oard1* | 1.635003183 | 0.048997238 |
| *Cystm1* | 2.787006277 | 0.001504396 | *Cdc37l1* | 2.371968777 | 0.032619731 | *Gng3* | 1.594361199 | 0.017939274 |
| *Snx3* | 2.787006277 | 0.00194964 | *Ctps2* | 2.371968777 | 0.032619731 | *Bex4* | 1.594361199 | 0.017939274 |
| *Arpc2* | 2.693896872 | 0.007559647 | *Cox7a2* | 2.278859373 | 0.000130901 | *Ndufa5* | 1.594361199 | 0.032706315 |
| *Acyp1* | 2.693896872 | 0.007559647 | *Ifi27* | 2.219965684 | 0.00339173 | *Dynll2* | 1.594361199 | 0.032706315 |
| *B230219D22Rik* | 2.693896872 | 0.007559647 | *Uqcrfs1* | 2.179323699 | 0.005711779 | *Cox7c* | 1.578419655 | 0.022317599 |
| *Arfgap3* | 2.693896872 | 0.007559647 | *Ppp1r14b* | 2.179323699 | 0.005711779 | *Map1lc3b* | 1.564613855 | 0.012191466 |
| *Slc3a2* | 2.693896872 | 0.007559647 | *Tle5* | 2.179323699 | 0.006722935 | *BC031181* | 1.564613855 | 0.021315092 |
| *Ube2j1* | 2.693896872 | 0.031076219 | *Set* | 2.179323699 | 0.018800582 | *Cope* | 1.564613855 | 0.033071896 |
| *Idh3a* | 2.693896872 | 0.031076219 | *Ssb* | 2.179323699 | 0.018800582 | *Ubl5* | 1.564613855 | 0.05154064 |
| *Mrps12* | 2.594361199 | 0.006496542 | *Supt4a* | 2.179323699 | 0.018800582 | *Ndufa2* | 1.523971871 | 0.044758767 |
| *Timm10b* | 2.541893779 | 0.000318898 | *Trappc3* | 2.108934372 | 0.02820036 | *Rpl35a* | 1.520067416 | 0.001277193 |
| *Kdelr1* | 2.371968777 | 0.001404823 | *Bpgm* | 2.108934372 | 0.02820036 | *Atp5pb* | 1.497499659 | 0.031758469 |
| *Ppid* | 2.371968777 | 0.004935546 | *Fmc1* | 2.108934372 | 0.02820036 | *Selenok* | 1.445969359 | 0.000150966 |
| *Gpr85* | 2.371968777 | 0.004935546 | *Tpi1* | 2.108934372 | 0.02820036 | *Rps29* | 1.416362897 | 0.000981733 |
| *Tsn* | 2.371968777 | 0.007178926 | *Josd2* | 2.108934372 | 0.02820036 | *Gng5* | 1.371968777 | 0.001618824 |
| *S100a11* | 2.371968777 | 0.007178926 | *Ei24* | 2.108934372 | 0.02820036 | *Uqcr11* | 1.371968777 | 0.004815126 |
| *Gng10* | 2.371968777 | 0.007178926 | *Pafah1b2* | 2.108934372 | 0.02820036 | *Spcs1* | 1.371968777 | 0.009005955 |
| *Tbrg1* | 2.371968777 | 0.007178926 | *Mrpl54* | 2.108934372 | 0.02820036 | *Rps21* | 1.371968777 | 0.009521455 |
| *Jkamp* | 2.371968777 | 0.007178926 | *Arl6* | 2.108934372 | 0.02820036 | *Rnasek* | 1.371968777 | 0.009713254 |
| *Gnb2* | 2.371968777 | 0.011108578 | *Tapbp* | 2.108934372 | 0.02820036 | *Ywhaq* | 1.371968777 | 0.015467307 |
| *Vps29* | 2.371968777 | 0.025892377 | *Matr3* | 2.108934372 | 0.02820036 | *Srpr* | 1.371968777 | 0.025070311 |
| *Phf3* | 2.371968777 | 0.032619731 | *Calb1* | 2.072408496 | 0.026353122 | *Ndufc1* | 1.371968777 | 0.038730422 |
| *Sf3b1* | 2.371968777 | 0.032619731 | *Snhg18* | 2.050040682 | 0.012699016 | *Psma3* | 1.371968777 | 0.038730422 |
| *Gm2000* | 2.371968777 | 0.032619731 | *Hsd17b8* | 2.050040682 | 0.012699016 | *Ndufs3* | 1.371968777 | 0.040112641 |
| *Tor2a* | 2.371968777 | 0.032619731 | *Trir* | 2.050040682 | 0.037805494 | *Psme2* | 1.371968777 | 0.040112641 |

| **Supplemental Table S9C DEGS LAC4 Cluster; VCD/Vehicle.** Maroon=upregulated; Blue=downregulated | | | | | | | | |
| --- | --- | --- | --- | --- | --- | --- | --- | --- |
| **gene** | **avg_log2FC** | **p_val** | **gene** | **avg_log2FC** | **p_val** | **gene** | **avg_log2FC** | **p_val** |
| *Sub1* | 1.371968777 | 0.040112641 |  |  |  |  |  |  |
| *Morf4l2* | 1.371968777 | 0.040112641 |  |  |  |  |  |  |
| *Rnaset2a* | 1.371968777 | 0.05420615 |  |  |  |  |  |  |
| *Rps28* | 1.33349463 | 0.000544389 |  |  |  |  |  |  |
| *H2bc4* | 1.304854582 | 0.006745893 |  |  |  |  |  |  |
| *Slc25a4* | 1.265053573 | 0.006468645 |  |  |  |  |  |  |
| *Eif3f* | 1.246437895 | 0.030940525 |  |  |  |  |  |  |
| *Rps17* | 1.24364468 | 0.000641033 |  |  |  |  |  |  |
| *Sem1* | 1.234465254 | 0.027469246 |  |  |  |  |  |  |
| *Ndufa13* | 1.219965684 | 0.010123718 |  |  |  |  |  |  |
| *Sdf4* | 1.219965684 | 0.029336019 |  |  |  |  |  |  |
| *Tmed3* | 1.1655179 | 0.022829746 |  |  |  |  |  |  |
| *Rpl36* | 1.162515412 | 0.026677469 |  |  |  |  |  |  |
| *Rplp2* | 1.157843972 | 0.028981589 |  |  |  |  |  |  |
| *Atp5j* | 1.137503524 | 0.02670448 |  |  |  |  |  |  |
| *Rpl37a* | 1.13380904 | 0.005281079 |  |  |  |  |  |  |
| *Rplp1* | 1.095128572 | 0.037946636 |  |  |  |  |  |  |
| *Rps27* | 1.067114196 | 0.024153762 |  |  |  |  |  |  |
| *Rpl34* | 1.062640719 | 0.007659403 |  |  |  |  |  |  |
| *Cox8a* | 1.060024771 | 0.02181034 |  |  |  |  |  |  |
| *Rpl39* | 1.03093186 | 0.012461298 |  |  |  |  |  |  |
| *Atpif1* | 1.009398698 | 0.014273158 |  |  |  |  |  |  |
| *Pfdn5* | 0.993457154 | 0.017559993 |  |  |  |  |  |  |
| *Rpl41* | 0.93900937 | 0.001028985 |  |  |  |  |  |  |
| *Chchd2* | 0.895530733 | 0.028165391 |  |  |  |  |  |  |
| *Rps18* | 0.872397768 | 0.050723245 |  |  |  |  |  |  |
| *Spcs2* | 0.857395605 | 0.032818937 |  |  |  |  |  |  |
| *Rps13* | 0.839747739 | 0.020981157 |  |  |  |  |  |  |
| *Rpl30* | 0.833548863 | 0.031037616 |  |  |  |  |  |  |
| *Ndfip1* | 0.827648261 | 0.025075663 |  |  |  |  |  |  |
| *Rpl37* | 0.817379926 | 0.01572072 |  |  |  |  |  |  |
| *Rpl22* | 0.808067892 | 0.02014227 |  |  |  |  |  |  |
| *Rpl27* | 0.800812076 | 0.047249362 |  |  |  |  |  |  |
| *Fau* | 0.797732683 | 0.024015722 |  |  |  |  |  |  |
| *Rps25* | 0.787006277 | 0.04248427 |  |  |  |  |  |  |
| *Rpl36al* | 0.738096676 | 0.035910218 |  |  |  |  |  |  |
| *Rpl24* | 0.734538857 | 0.041434608 |  |  |  |  |  |  |
| *Rpl11* | 0.732850507 | 0.049442351 |  |  |  |  |  |  |
| *Rps11* | 0.731511164 | 0.052094201 |  |  |  |  |  |  |
| *Rpl12* | 0.717104263 | 0.051660556 |  |  |  |  |  |  |
| *Rpl31* | 0.713005695 | 0.027910483 |  |  |  |  |  |  |
| *Rps14* | 0.681072832 | 0.051277193 |  |  |  |  |  |  |
|  |  |  |  |  |  |  |  |  |
|  |  |  |  |  |  |  |  |  |
| *Gal* | -0.937359281 | 0.026916834 |  |  |  |  |  |  |
| *Ikbip* | -1.534921818 | 0.051193173 |  |  |  |  |  |  |
| *Nup210l* | -2.087462841 | 0.046871383 |  |  |  |  |  |  |
|  |  |  |  |  |  |  |  |  |
|  |  |  |  |  |  |  |  |  |
|  |  |  |  |  |  |  |  |  |
|  |  |  |  |  |  |  |  |  |
|  |  |  |  |  |  |  |  |  |
|  |  |  |  |  |  |  |  |  |

Supplemental Tables 6-9. Differentially expressed genes in LAC clusters 1-4.

| Supplemental Table S10A. Differentially Expressed Genes Representing G Protein Receptor Canonical Pathway in Lactotropes  VCD vs Vehicle control females | | | | | | | |
| --- | --- | --- | --- | --- | --- | --- | --- |
|  |  | **Lactotrope 1 (LAC1)** | | **Lactotrope 2 (LAC2)** | | **Lactotrope 3 (LAC3)** | |
|  | **Gene** | **Expr Log Ratio** | **Expr p-value** | **Expr Log Ratio** | **Expr p-value** | **Expr Log Ratio** | **Expr p-value** |
| adenylate cyclase 6 | ADCY6 |  |  | 0.751 | 0.0098 |  |  |
| adhesion G protein-coupled receptor A1 | ADGRA1 |  |  | 1.35 | 0.0053 |  |  |
| adhesion G protein-coupled receptor A3 | ADGRA3 |  |  | 1.227 | 0.00191 |  |  |
| adhesion G protein-coupled receptor L1 | ADGRL1 | 0.783 | 0.00329 |  |  |  |  |
| AKT serine/threonine kinase 1 | AKT1 | 1.714 | 0.000684 |  |  |  |  |
| cadherin EGF LAG seven-pass G-type receptor 3 | CELSR3 | 0.677 | 0.0016 |  |  |  |  |
| calcineurin like EF-hand protein 1 | CHP1 | 0.626 | 0.0113 | 1.26 | 0.0000656 | 0.822 | 0.00319 |
| cell division cycle 42 | CDC42 |  |  |  |  | 0.685 | 0.00154 |
| cholinergic receptor muscarinic 3 | CHRM3 | 2.505 | 0.0208 |  |  |  |  |
| cyclic nucleotide gated channel subunit beta 3 | CNGB3 | 1.768 | 0.0168 | 1.89 | 0.0283 |  |  |
| cAMP responsive element binding protein 3 like 1 | CREB3L1 | 0.938 | 4.65E-08 | 1.304 | 3.93E-26 | 0.873 | 0.0000293 |
| cAMP responsive element binding protein 3 like 2 | CREB3L2 |  |  | 0.657 | 0.00351 |  |  |
| elongator acetyltransferase complex subunit 1 | ELP1 | 0.562 | 0.0484 | 0.789 | 0.00718 |  |  |
| frizzled class receptor 8 | FZD8 | 2.353 | 0.0387 |  |  |  |  |
| forkhead box O1 | FOXO1 |  |  | 0.673 | 0.0344 |  |  |
| forkhead box O3 | FOXO3 |  |  |  |  | 0.801 | 0.00796 |
| G protein subunit alpha 11 | GNA11 |  |  | 0.8 | 0.000227 |  |  |
| G protein subunit alpha 13 | GNA13 |  |  | 0.704 | 0.0505 |  |  |
| G protein subunit alpha L | GNAL |  |  | 1.212 | 0.0365 |  |  |
| G protein subunit gamma 4 | GNG4 |  |  | 0.736 | 0.00049 |  |  |
| G protein-coupled receptor 3 | GPR3 |  |  | 2.283 | 0.0183 |  |  |
| G protein-coupled receptor 52 | GPR52 |  |  | 2.06 | 0.039 |  |  |
| G protein-coupled receptor 83 | GPR83 |  |  | 3.06 | 0.00829 |  |  |
| **G protein regulated inducer of neurite outgrowth 1** | **GPRIN1** |  |  |  |  | **-0.882** | **0.0501** |
| G protein-coupled receptor 180 | GPR180 |  |  |  |  | 0.621 | 0.0293 |
| G protein-coupled receptor 137 | GPR137 | 0.894 | 0.0436 |  |  |  |  |
| G protein-coupled receptor 171 | GPR171 | 1.669 | 0.023 |  |  |  |  |
| G protein-coupled receptor 18 | GPR18 | -1.495 | 0.0351 |  |  |  |  |
| histamine receptor H4 | HRH4 | 4.09 | 0.00072 |  |  |  |  |
| 5-hydroxytryptamine receptor 7 | HTR7 | 2.184 | 0.0387 |  |  |  |  |
| Jun proto-oncogene, AP-1 transcription factor subunit | JUN | 0.651 | 0.0000986 | 0.829 | 7.84E-08 | 0.637 | 1.07E-08 |
| potassium calcium-activated channel subfamily N member 1 | KCNN1 | -1.986 | 0.051 |  |  |  |  |
| potassium voltage-gated channel subfamily H member 2 | KCNN2 |  |  |  |  | 1.579 | 0.00278 |
| potassium voltage-gated channel subfamily Q member 3 | KCNQ3 | 2.283 | 0.0000181 |  |  |  |  |
| leucine rich repeat containing G protein-coupled receptor 4 | LGR4 | 0.884 | 0.0192 |  |  |  |  |
| mitogen-activated protein kinase kinase kinase 11 | MAP3K11 | 1.768 | 0.0349 |  |  |  |  |
| mitogen-activated protein kinase kinase 2 | MAP2K2 |  |  | 0.599 | 0.00311 |  |  |
| mitogen-activated protein kinase kinase 6 | MAP2K6 |  |  | 1.212 | 0.0365 |  |  |
| mitogen-activated protein kinase kinase kinase 12 | MAP3K12 |  |  | 0.928 | 0.0402 |  |  |
| mitogen-activated protein kinase kinase kinase 4 | MAP3K4 |  |  | 1.376 | 0.00152 |  |  |
| mitogen-activated protein kinase 8 | MAPK8 |  |  | 0.938 | 0.00642 |  |  |
| mitogen-activated protein kinase 9 | MAPK9 |  |  | 1.382 | 0.00261 | 0.864 | 0.0361 |
| mitogen-activated protein kinase kinase 7 |  |  |  |  |  | 1.157 | 0.00115 |
| **mitogen-activated protein kinase kinase kinase 13** |  |  |  |  |  | **-1.387** | **0.0376** |

| Supplemental Table S10B. Differentially Expressed Genes Representing G Protein Receptor Canonical Pathway in Lactotropes  VCD vs Vehicle control females | | | | | | | |
| --- | --- | --- | --- | --- | --- | --- | --- |
|  | | **Lactotrope 1 (LAC1)** | | **Lactotrope 2 (LAC2)** | | **Lactotrope 3 (LAC3)** | |
| **Gene** | | **Expr Log Ratio** | **Expr p-value** | **Expr Log Ratio** | **Expr p-value** | **Expr Log Ratio** | **Expr p-value** |
| mitogen-activated protein kinase kinase kinase 5 | MAP3K5 |  |  |  |  | 1.304 | 0.000846 |
| mitogen-activated protein kinase 14 | MAPK14 |  |  |  |  | 0.674 | 0.0205 |
| mitogen-activated protein kinase 9 | MAPK9 |  |  | 1.382 | 0.00261 | 0.864 | 0.0361 |
| metallophosphoesterase domain containing 2 | MPPED2 | 0.983 | 0.0166 | 1.06 | 0.00271 |  |  |
| myocardin related transcription factor A | MRTFA | 1.012 | 0.000677 |  |  |  |  |
| myosin light chain 12B | Myl12b |  |  | 0.639 | 0.03 |  |  |
| NFKB inhibitor alpha | NFKBIA |  |  |  |  | 0.644 | 0.00537 |
| NFKB inhibitor beta | NFKBIB |  |  | 0.961 | 0.0417 |  |  |
| nuclear factor of activated T cells 3 | NFATC3 | 0.709 | 0.00606 |  |  |  |  |
| **opioid receptor mu 1** | **OPRM1** | **-1.369** | **0.00252** |  |  |  |  |
| purinergic receptor | P2Y14 |  |  | 0.901 | 0.0182 | 1.064 | 0.00549 |
| p21 (RAC1) activated kinase 1 | PAK1 |  |  | 0.703 | 0.0101 |  |  |
| phosphodiesterase 10A | PDE10A | 0.694 | 6.31E-17 | 0.772 | 1.55E-20 |  |  |
| phosphodiesterase 12 | PDE12 |  |  |  |  | 1.064 | 0.0326 |
| phosphodiesterase 1C | PDE1C | 0.8 | 0.00635 |  |  | 1.148 | 0.00239 |
| **phosphatidylinositol-4-phosphate 3-kinase catalytic subunit type 2 gamma** | **PIK3C2G** |  |  | **-1.302** | **0.0495** |  |  |
| phosphoinositide-3-kinase regulatory subunit 1 | PIK3R1 |  |  | 0.668 | 0.0149 |  |  |
| phosphoinositide-3-kinase regulatory subunit 2 | PIK3R2 |  |  |  |  | 1.19 | 0.0171 |
| phosphoinositide-3-kinase regulatory subunit 3 | PIK3R3 | 0.621 | 0.00116 |  |  |  |  |
| protein kinase cAMP-dependent type II regulatory subunit alpha | PRKAR2A |  |  | 0.755 | 0.00205 | 0.593 | 0.0116 |
| protein kinase cAMP-activated catalytic subunit alpha | PRKACA |  |  |  |  | 0.905 | 0.000799 |
| protein kinase C alpha | PRKCA |  |  | 0.851 | 0.00156 | 0.605 | 0.0371 |
| protein kinase C gamma | PRKCG |  |  | 1.838 | 0.00553 |  |  |
| RAS like proto-oncogene B | RALB |  |  | 1.074 | 0.000934 |  |  |
| Ras related dexamethasone induced 1 | RASD1 |  |  |  |  | 0.643 | 0.0353 |
| RAP1B, member of RAS oncogene family | RAP1B |  |  | 1.074 | 0.000934 |  |  |
| RAP2A, member of RAS oncogene family | RAP2A |  |  | 0.81 | 0.0539 |  |  |
| REL proto-oncogene, NF-kB subunit | REL |  |  | 1.668 | 0.00999 | 1.29 | 0.032 |
| regulator of G protein signaling 7 | RGS7 |  |  | 0.617 | 0.00237 |  |  |
| ribosomal protein S6 kinase A1 | RPS6KA1 | 3.643 | 0.00251 |  |  |  |  |
| SOS Ras/Rac guanine nucleotide exchange factor 1 | SOS1 |  |  | 0.606 | 0.0306 |  |  |
| **sphingomyelin phosphodiesterase acid like 3A** | **SMPDL3A** | **-1.401** | **0.0238** |  |  | **-1.316** | **0.0516** |
| SRC proto-oncogene, non-receptor tyrosine kinase | SRC |  |  |  |  | 1.38 | 0.0194 |
| signal transducer and activator of transcription 3 | STAT3 |  |  |  |  | 1.03 | 0.0000857 |
| synaptic Ras GTPase activating protein 1 | SYNGAP1 | 1.381 | 0.000235 |  |  |  |  |
| WW domain containing transcription regulator 1 | WWTR1 | 3.184 | 0.00892 |  |  |  |  |
| Yes1 associated transcriptional regulator | YAP1 | 1.768 | 0.0349 |  |  |  |  |
|  |  |  |  |  |  |  |  |
| **G protein pathway DEGs for LAC4** | **GENE** | **Expr Log Ratio** | **Expr p-value** |  |  |  |  |
| G protein subunit beta 2 | GNB2 | 2.372 | 0.0111 |  |  |  |  |
| G protein subunit gamma 10 | GNG10 | 2.372 | 0.00718 |  |  |  |  |
| G protein subunit gamma 3 | GNG3 | 1.594 | 0.0179 |  |  |  |  |
| G protein subunit gamma 4 | GNG4 | 2.957 | 0.0233 |  |  |  |  |
| G protein subunit gamma 5 | GNG5 | 1.372 | 0.00162 |  |  |  |  |
| G protein-coupled receptor 85 | GPR85 | 2.372 | 0.00494 |  |  |  |  |
| protein kinase cAMP-activated catalytic subunit alpha | PRKACA | 2.957 | 0.0233 |  |  |  |  |
| protein kinase cAMP-activated catalytic subunit beta | PRKACB | 1.787 | 0.0374 |  |  |  |  |
| protein kinase AMP-activated non-catalytic subunit gamma 2 | PRKAG2 | 2.957 | 0.0233 |  |  |  |  |

| Supplemental Table S11A. Differentially Expressed Genes Representing Estrogen Receptor Signaling in Lactotropes  VCD vs Vehicle control females | | | | | | | |
| --- | --- | --- | --- | --- | --- | --- | --- |
|  | | **Lactotrope 1 (LAC1)** | | **Lactotrope 2 (LAC2)** | | **Lactotrope 3 (LAC3)** | |
| **Gene** | | **Expr Log Ratio** | **Expr p-value** | **Expr Log Ratio** | **Expr p-value** | **Expr Log Ratio** | **Expr p-value** |
| adenylate cyclase 6 | ADCY6 |  |  | 0.751 | 0.0098 |  |  |
| AKT serine/threonine kinase 1 | AKT1 | 1.714 | 0.000684 |  |  |  |  |
| calcium voltage-gated channel subunit alpha1 B | CACNA1B |  |  |  |  | 0.877 | 0.00979 |
| calcium voltage-gated channel subunit alpha1 E | CACNA1E |  |  |  |  | 0.694 | 0.042 |
| cyclin C | CCNC | -0.703 | 0.0275 |  |  |  |  |
| cyclin dependent kinase inhibitor 1A | CDKN1A |  |  |  |  | 2.742 | 0.00807 |
| cAMP responsive element binding protein 3 like 1 | CREB3L1 | 0.938 | 4.65E-08 | 1.304 | 3.93E-26 | 0.873 | 0.0000293 |
| cAMP responsive element binding protein 3 like 2 | CREB3L2 |  |  | 0.657 | 0.00351 |  |  |
| C-terminal binding protein 1 | CTBP1 | 0.884 | 0.00669 |  |  |  |  |
| eukaryotic translation initiation factor 2B subunit beta | EIFB2 |  |  |  |  | 1.501 | 0.00284 |
| eukaryotic translation initiation factor 4E | EIF4E | 0.573 | 0.0177 | 0.74 | 0.00589 |  |  |
| eukaryotic translation initiation factor 2B subunit delta | EIF2B4 | 1.05 | 0.0119 |  |  |  |  |
| forkhead box O1 | FOXO1 |  |  | 0.673 | 0.0344 |  |  |
| forkhead box O3 | FOXO3 |  |  |  |  | 0.801 | 0.00796 |
| G protein subunit alpha 11 | GNA11 |  |  | 0.8 | 0.000227 |  |  |
| G protein subunit alpha 13 | GNA13 |  |  | 0.704 | 0.0505 |  |  |
| G protein subunit alpha L | GNAL |  |  | 1.212 | 0.0365 |  |  |
| G protein subunit gamma 4 | GNG4 |  |  | 0.736 | 0.00049 |  |  |
| insulin like growth factor 2 receptor | IGF2R |  |  | 0.81 | 0.0103 |  |  |
| G protein subunit alpha 11 | GNA11 |  |  | 0.8 | 0.000227 |  |  |
| Janus kinase 2 | JAK2 |  |  | 0.926 | 0.0052 |  |  |
| Jun proto-oncogene, AP-1 transcription factor subunit | JUN | 0.651 | 0.0000986 | 0.829 | 7.84E-08 | 0.637 | 1.07E-08 |
| mitogen-activated protein kinase kinase 2 | MAP2K2 |  |  | 0.599 | 0.00311 |  |  |
| mediator complex subunit 12L | MED12L |  |  | 0.587 | 0.00342 |  |  |
| mediator complex subunit 13 | MED13 |  |  |  |  | 0.636 | 0.0152 |
| mediator complex subunit 15 | MED15 |  |  | 0.606 | 0.0439 | 0.681 | 0.0189 |
| mediator complex subunit 16 | MED16 |  |  | 1.368 | 0.0315 |  |  |
| mediator complex subunit 27 | MED27 |  |  |  |  | 0.681 | 0.0219 |
| myosin light chain 12B | Myl12b |  |  | 0.639 | 0.03 |  |  |
| NADH:ubiquinone oxidoreductase subunit A3 | NDUFA3 |  |  |  |  | 0.649 | 0.0469 |
| **NADH:ubiquinone oxidoreductase subunit A9** | **NDUFA9** |  |  |  |  | **-1.427** | **0.0299** |
| NADH:ubiquinone oxidoreductase subunit B6 | NDUFB6 |  |  | 0.687 | 0.00259 |  |  |
| NADH:ubiquinone oxidoreductase subunit B7 | NDUFB7 |  |  |  |  | 1.421 | 0.033 |
| NADH:ubiquinone oxidoreductase core subunit S3 | NDUFS3 |  |  | 0.56 | 0.0136 | 0.605 | 0.0132 |
| NADH:ubiquinone oxidoreductase core subunit S8 | NDUFS8 |  |  |  |  | -0.597 | 0.0331 |
| NADH:ubiquinone oxidoreductase subunit V3 | NDUFV3 | 0.626 | 0.0161 |  |  |  |  |
| p21 (RAC1) activated kinase 1 | PAK1 |  |  | 0.703 | 0.0101 |  |  |
| platelet derived growth factor C | PDGFC |  |  |  |  |  |  |
| proliferating cell nuclear antigen | PCNA | 1.473 | 0.00828 |  |  |  |  |
| **phosphatidylinositol-4-phosphate 3-kinase catalytic subunit type 2 gamma** | **PIK3C2G** |  |  | **-1.302** | **0.0495** |  |  |
| phosphoinositide-3-kinase regulatory subunit 1 | PIK3R1 |  |  | 0.668 | 0.0149 |  |  |
| phosphoinositide-3-kinase regulatory subunit 2 | PIK3R2 |  |  |  |  | 1.19 | 0.0171 |
| **phosphatidylinositol-4-phosphate 3-kinase catalytic subunit type 2 gamma** | **PIK3C2G** |  |  | **-1.302** | **0.0495** |  |  |
| phosphoinositide-3-kinase regulatory subunit 1 | PIK3R1 |  |  | 0.668 | 0.0149 |  |  |
| phosphoinositide-3-kinase regulatory subunit 3 | PIK3R3 | 0.621 | 0.00116 |  |  |  |  |
| phospholipase C like 1 (inactive) | PLCL1 | 1.447 | 0.00537 |  |  |  |  |
| protein kinase AMP-activated non-catalytic subunit beta 2 | PRKAB2 | 0.753 | 0.0368 |  |  |  |  |
| protein kinase cAMP-activated catalytic subunit alpha | PRKACA |  |  |  |  | 0.905 | 0.000799 |
| protein kinase cAMP-dependent type II regulatory subunit alpha | PRKAR2A |  |  | 0.755 | 0.00205 | 0.593 | 0.0116 |
| protein kinase C alpha | PRKCA |  |  | 0.851 | 0.00156 | 0.605 | 0.0371 |
| protein kinase C gamma | PRKCG | 0.698 | 0.197 | 1.838 | 0.00553 |  |  |
| ras related dexamethasone induced 1 | RASD1 |  |  |  |  | 0.643 | 0.0353 |

| Supplemental Table S11B. Differentially Expressed Genes Representing Estrogen Receptor Signaling in Lactotropes  VCD vs Vehicle control females | | | | | | | | |
| --- | --- | --- | --- | --- | --- | --- | --- | --- |
|  |  | **Lactotrope 1 (LAC1)** | | **Lactotrope 2 (LAC2)** | | | **Lactotrope 3 (LAC3)** | |
|  | **Gene** | **Expr Log Ratio** | **Expr p-value** | **Expr Log Ratio** | | **Expr p-value** | **Expr Log Ratio** | **Expr p-value** |
| **RAS like proto-oncogene B** | **RALB** |  |  | **-1.525** | **0.0325** | |  |  |
| RAP1B, member of RAS oncogene family | RAP1B |  |  | 1.074 | 0.000934 | |  |  |
| RAP2A, member of RAS oncogene family | RAP2A |  |  | 0.81 | 0.0539 | |  |  |
| REL proto-oncogene, NF-kB subunit | REL |  |  | 1.668 | 0.00999 | | 1.29 | 0.032 |
| SOS Ras/Rac guanine nucleotide exchange factor 1 | SOS1 |  |  | 0.606 | 0.0306 | |  |  |
| Protein kinase AMP-activated non-catalytic subunit beta 2 | PRKAB2 | 0.753 | 0.0368 |  |  | |  |  |
| RNA polymerase II subunit B | POLR2B |  |  | 0.901 | 0.00719 | |  |  |
| **RAS like proto-oncogene B** | **RALB** |  |  | **-1.525** | **0.0325** | |  |  |
| **succinate dehydrogenase complex subunit D** | **SDHD** | **-1.755** | **0.0322** |  |  | |  |  |
| Sp1 transcription factor | SP1 | 0.768 | 0.0217 | 0.838 | 0.017 | |  |  |
| transformation/transcription domain associated protein | TRRAP | 0.636 | 0.00477 |  |  | |  |  |
| tyrosine kinase 2 | TYK2 | 0.841 | 0.0295 |  |  | |  |  |
| ubiquinol-cytochrome c reductase core protein 2 | UQCRC2 | 0.887 | 0.015 |  |  | |  |  |
| vascular endothelial growth factor A | VEGFA | 0.949 | 0.00684 |  |  | |  |  |
| vascular endothelial growth factor B | VEGFB |  |  |  |  | | 1.105 | 0.0288 |
| zinc finger DHHC-type palmitoyltransferase 7 | ZDHHC7 | 1.127 | 0.0104 | 1.838 | 0.00165 | |  |  |
|  |  |  |  |  |  | |  |  |
| **DEGs for Lactotrope 4 Cluster** |  | **Lactotrope 4 (LAC4)** | | **Lactotrope 2 (LAC2)** | | | **Lactotrope 3 (LAC3** | |
| ATP synthase peripheral stalk-membrane subunit b | ATP5PB | 1.497 | 0.0318 |  |  | |  |  |
| G protein subunit beta 2 | GNB2 | 2.372 | 0.0111 |  |  | |  |  |
| G protein subunit gamma 10 | GNG10 | 2.372 | 0.00718 |  |  | |  |  |
| G protein subunit gamma 3 | GNG3 | 1.594 | 0.0179 |  |  | |  |  |
| G protein subunit gamma 4 | GNG4 | 2.957 | 0.0233 | 0.736 | 0.00049 | |  |  |
| G protein subunit gamma 5 | GNG5 | 1.372 | 0.00162 |  |  | | 0.567 | 0.00851 |
| matrix metallopeptidase 23B | MMP23B | 3.372 | 0.00493 |  |  | |  |  |
| NADH:ubiquinone oxidoreductase subunit A13 | NDUFA13 | 1.22 | 0.0101 |  |  | |  |  |
| NADH:ubiquinone oxidoreductase subunit A2 | NDUFA2 | 1.524 | 0.0448 |  |  | |  |  |
| NDUFA4 mitochondrial complex associated | NDUFA4 | 1.841 | 0.000456 |  |  | |  |  |
| NADH:ubiquinone oxidoreductase subunit A5 | NDUFA5 | 1.594 | 0.0327 | 0.56 | 0.0136 | |  |  |
| **NADH:ubiquinone oxidoreductase core subunit S3** | NDUFS3 | 1.372 | 0.0401 |  |  | | -0.597 | 0.0331 |
| protein kinase cAMP-activated catalytic subunit alpha | PRKACA | 2.957 | 0.0233 |  |  | | 0.905 | 0.000799 |
| protein kinase cAMP-activated catalytic subunit beta | PRKACB | 1.787 | 0.0374 |  |  | |  |  |
| protein kinase AMP-activated non-catalytic subunit gamma 2 | PRKAG2 | 2.957 | 0.0233 |  |  | |  |  |
| ubiquinol-cytochrome c reductase core protein 2 | UQCRC2 | 1.857 | 0.015 |  |  | |  |  |
| ubiquinol-cytochrome c reductase, Rieske iron-sulfur polypeptide 1 | UQCRFS1 | 2.179 | 0.00571 |  |  | |  |  |

| **Supplemental Table S12A DEGS Thyrotrope Cluster; VCD/Vehicle.** Maroon=upregulated; | | | | | | | | |
| --- | --- | --- | --- | --- | --- | --- | --- | --- |
| **gene** | **avg_log2FC** | **p_val** | **gene** | **avg_log2FC** | **p_val** | **gene** | **avg_log2FC** | **p_val** |
| *Ptk2b* | 4.459431619 | 0.002654034 | *2610008E11Rik* | 2.807354922 | 0.041655546 | *Nbr1* | 2.321928095 | 0.022663096 |
| *Zfp236* | 3.700439718 | 0.002652822 | *Dph3* | 2.807354922 | 0.041655546 | *Lactb2* | 2.321928095 | 0.041673046 |
| *Ckb* | 3.584962501 | 0.002654034 | *Cd200* | 2.807354922 | 0.041655546 | *Snhg7* | 2.321928095 | 0.041673046 |
| *2900026A02Rik* | 3.459431619 | 0.010773441 | *G6pdx* | 2.807354922 | 0.041655546 | *Aqr* | 2.321928095 | 0.041673046 |
| *Gdpd3* | 3.321928095 | 0.002610611 | *Dbpht2* | 2.736965594 | 0.016279863 | *Lurap1l* | 2.321928095 | 0.041673046 |
| *Zfp407* | 3.321928095 | 0.00262038 | *Cul4a* | 2.700439718 | 0.005448963 | *Rnf220* | 2.321928095 | 0.041673046 |
| *Uqcrc2* | 3.321928095 | 0.005376822 | *Clk4* | 2.700439718 | 0.021233903 | *Yrdc* | 2.321928095 | 0.041673046 |
| *Ppa1* | 3.321928095 | 0.005376822 | *Cdkl1* | 2.700439718 | 0.021959478 | *Kcnh2* | 2.321928095 | 0.041673046 |
| *Msh3* | 3.321928095 | 0.005376822 | *Wls* | 2.700439718 | 0.022663096 | *Tada2b* | 2.321928095 | 0.041673046 |
| *Phf21b* | 3.321928095 | 0.005376822 | *Anapc1* | 2.584962501 | 0.021318647 | *Pop5* | 2.321928095 | 0.041673046 |
| *Zcchc17* | 3.321928095 | 0.021282416 | *Fam76a* | 2.584962501 | 0.021318647 | *Ogfod2* | 2.321928095 | 0.041673046 |
| *Cd164* | 3.321928095 | 0.041655546 | *Cox19* | 2.584962501 | 0.021318647 | *Bola3* | 2.321928095 | 0.041673046 |
| *Cmas* | 3.169925001 | 0.005340993 | *Fuz* | 2.584962501 | 0.021318647 | *Pianp* | 2.321928095 | 0.041673046 |
| *Zswim6* | 3.169925001 | 0.005340993 | *Knop1* | 2.584962501 | 0.021318647 | *Ing4* | 2.321928095 | 0.041673046 |
| *Sec23ip* | 3.169925001 | 0.010773441 | *Inpp5f* | 2.584962501 | 0.021318647 | *Sirt2* | 2.321928095 | 0.041673046 |
| *Ginm1* | 3.169925001 | 0.010773441 | *Cherp* | 2.584962501 | 0.021318647 | *Cep89* | 2.321928095 | 0.041673046 |
| *Usp7* | 3.169925001 | 0.010773441 | *Ube2d1* | 2.584962501 | 0.021318647 | *Jakmip3* | 2.321928095 | 0.041673046 |
| *Fgfr1op2* | 3.169925001 | 0.021282416 | *Smarcd2* | 2.584962501 | 0.021318647 | *Abce1* | 2.321928095 | 0.041673046 |
| *Psmb4* | 3.169925001 | 0.041655546 | *Csnk1d* | 2.584962501 | 0.021318647 | *Wdr59* | 2.321928095 | 0.041673046 |
| *Kazn* | 3.000000000 | 0.002466012 | *Isca1* | 2.584962501 | 0.021318647 | *Gm56878* | 2.321928095 | 0.041673046 |
| *Susd4* | 3.000000000 | 0.010726726 | *Zfp944* | 2.584962501 | 0.021318647 | *Jph3* | 2.321928095 | 0.041673046 |
| *Ncbp1* | 3.000000000 | 0.010726726 | *Naa10* | 2.584962501 | 0.021318647 | *Prkar2a* | 2.321928095 | 0.041673046 |
| *Ran* | 3.000000000 | 0.010726726 | *F8* | 2.584962501 | 0.021318647 | *Acss3* | 2.321928095 | 0.041673046 |
| *Msl2* | 3.000000000 | 0.010726726 | *Stx18* | 2.584962501 | 0.021959478 | *Trp53* | 2.321928095 | 0.041673046 |
| *Psmd11* | 3.000000000 | 0.010726726 | *Atg4b* | 2.584962501 | 0.041603056 | *Sp2* | 2.321928095 | 0.041673046 |
| *Rcan1* | 3.000000000 | 0.021282416 | *Bmi1* | 2.584962501 | 0.041603056 | *Psma6* | 2.321928095 | 0.041673046 |
| *Tecr* | 3.000000000 | 0.041603056 | *Snrnp200* | 2.584962501 | 0.041603056 | *Slc39a9* | 2.321928095 | 0.041673046 |
| *Plekha1* | 3.000000000 | 0.041655546 | *Pygb* | 2.584962501 | 0.041603056 | *Isca2* | 2.321928095 | 0.041673046 |
| *Jak2* | 3.000000000 | 0.041655546 | *Yars* | 2.584962501 | 0.041603056 | *Cltb* | 2.321928095 | 0.041673046 |
| *Fam171a1* | 3.000000000 | 0.041673046 | *Zfp398* | 2.584962501 | 0.041603056 | *Smad5* | 2.321928095 | 0.041673046 |
| *St6gal1* | 3.000000000 | 0.041673046 | *Zfp783* | 2.584962501 | 0.041603056 | *Smn1* | 2.321928095 | 0.041673046 |
| *Sem1* | 2.807354922 | 0.010697548 | *Gm26944* | 2.584962501 | 0.041603056 | *Cdk7* | 2.321928095 | 0.041673046 |
| *Sec16a* | 2.807354922 | 0.010820254 | *Crtc1* | 2.584962501 | 0.041603056 | *Il17rd* | 2.321928095 | 0.041673046 |
| *Dcun1d5* | 2.807354922 | 0.010820254 | *6430548M08Rik* | 2.584962501 | 0.041603056 | *Dcaf13* | 2.321928095 | 0.041673046 |
| *Msantd2* | 2.807354922 | 0.010820254 | *Glce* | 2.584962501 | 0.041603056 | *Alg10b* | 2.321928095 | 0.041673046 |
| *Cebpz* | 2.807354922 | 0.010820254 | *Zdhhc3* | 2.584962501 | 0.041603056 | *Calcoco1* | 2.321928095 | 0.041673046 |
| *Unc50* | 2.807354922 | 0.021228114 | *Patz1* | 2.584962501 | 0.041603056 | *Hnrnpul2* | 2.321928095 | 0.041673046 |
| *Pea15a* | 2.807354922 | 0.021228114 | *Dynll2* | 2.584962501 | 0.041603056 | *Las1l* | 2.321928095 | 0.041673046 |
| *Bpnt1* | 2.807354922 | 0.021228114 | *Itga3* | 2.584962501 | 0.041603056 | *Pgk1* | 2.321928095 | 0.041673046 |
| *Atp5g3* | 2.807354922 | 0.021228114 | *Bcap29* | 2.584962501 | 0.041603056 | *Hsd17b10* | 2.321928095 | 0.041673046 |
| *Akirin1* | 2.807354922 | 0.021228114 | *Mta1* | 2.584962501 | 0.041603056 | *Shb* | 2.321928095 | 0.042770724 |
| *Ptpn11* | 2.807354922 | 0.021228114 | *Socs4* | 2.584962501 | 0.041603056 | *Hspa9* | 2.321928095 | 0.042770724 |
| *Clcn4* | 2.807354922 | 0.021228114 | *Supt16* | 2.584962501 | 0.041603056 | *Canx* | 2.321928095 | 0.045500264 |
| *Hid1* | 2.807354922 | 0.021228114 | *Zc3h7b* | 2.584962501 | 0.041603056 | *Ints8* | 2.222392421 | 0.008666757 |
| *Ap5m1* | 2.807354922 | 0.021228114 | *Arvcf* | 2.584962501 | 0.041603056 | *Lgr4* | 2.222392421 | 0.017252215 |
| *Sugt1* | 2.807354922 | 0.021228114 | *Igf2r* | 2.584962501 | 0.041603056 | *Kpna3* | 2.169925001 | 0.012468091 |
| *Gm56714* | 2.807354922 | 0.021228114 | *Rttn* | 2.584962501 | 0.041603056 | *Inpp4a* | 2.169925001 | 0.023342202 |
| *Shisal1* | 2.807354922 | 0.021228114 | *Banp* | 2.459431619 | 0.005754769 | *Ccnl2* | 2.169925001 | 0.023342202 |
| *Snx2* | 2.807354922 | 0.021228114 | *Hmgcs1* | 2.459431619 | 0.021965604 | *Atp6ap1* | 2.169925001 | 0.023342202 |
| *Wdr1* | 2.807354922 | 0.041603056 | *Cyb5a* | 2.459431619 | 0.021965604 | *Krtcap2* | 2.169925001 | 0.044144307 |
| *Pon2* | 2.807354922 | 0.041603056 | *Dhx40* | 2.459431619 | 0.022663096 | *Gm10069* | 2.169925001 | 0.044144307 |
| *Grid2* | 2.807354922 | 0.041603056 | *Epha3* | 2.459431619 | 0.022663096 | *Mcph1* | 2.169925001 | 0.044144307 |
| *Map3k4* | 2.807354922 | 0.041603056 | *Atf3* | 2.392317423 | 0.004965551 | *Copb2* | 2.169925001 | 0.044144307 |
| *4732440D04Rik* | 2.807354922 | 0.041655546 | *Gm973* | 2.321928095 | 0.011725883 | *Cuedc2* | 2.169925001 | 0.044144307 |
| *Pde4b* | 2.807354922 | 0.041655546 | *Rc3h2* | 2.321928095 | 0.011725883 | *Sec24d* | 2.115477217 | 0.001651395 |
| *Stmn1* | 2.807354922 | 0.041655546 | *Pcsk6* | 2.321928095 | 0.013344788 | *Psap* | 2.115477217 | 0.003945616 |
| *Grk3* | 2.807354922 | 0.041655546 | *Flvcr1* | 2.321928095 | 0.015335817 | *Prpf40a* | 2.115477217 | 0.009162607 |

| **Supplemental Table S12B DEGS Thyrotrope Cluster; VCD/Vehicle.** Maroon=upregulated; blue=downregulated | | | | | | | | |
| --- | --- | --- | --- | --- | --- | --- | --- | --- |
| **gene** | **avg_log2FC** | **p_val** | **gene** | **avg_log2FC** | **p_val** | **gene** | **avg_log2FC** | **p_val** |
| *Supt6* | 2.115477217 | 0.019252851 | *Smg1* | 1.584962501 | 0.0470317 | *Rfx7* | -1.678071905 | 0.043166219 |
| *Ddx50* | 2.115477217 | 0.033375421 | *Nup160* | 1.584962501 | 0.053970857 | *Fyn* | -1.700439718 | 0.043652633 |
| *Wdr7* | 2.000000000 | 0.006920201 | *Atp5l* | 1.584962501 | 0.054095427 | *Prkcb* | -1.765534746 | 0.043104065 |
| *Sgms2* | 2.000000000 | 0.023168623 | *Ern1* | 1.514573173 | 0.05134434 | *Fggy* | -1.807354922 | 0.043645597 |
| *Slc25a12* | 2.000000000 | 0.027140446 | *Prpf39* | 1.502500341 | 0.04715958 | *Reep3* | -1.874469118 | 0.028039738 |
| *Katnbl1* | 2.000000000 | 0.045500264 | *Zmiz1* | 1.485426827 | 0.031093201 | *Lrp1b* | -2 | 0.007692656 |
| *Fhip1a* | 2.000000000 | 0.045500264 | *Usp3* | 1.485426827 | 0.05183783 | *Pigp* | -2 | 0.024547158 |
| *Eps15* | 2.000000000 | 0.045500264 | *Ppm1l* | 1.473931188 | 0.034191514 | *Slc44a1* | -2 | 0.045500264 |
| *Rbbp6* | 2.000000000 | 0.045500264 | *Abi2* | 1.459431619 | 0.045018495 | *Sec24a* | -2.099535674 | 0.040359794 |
| *Zfp827* | 2.000000000 | 0.045500264 | *Tpr* | 1.459431619 | 0.045018495 | *Tlk2* | -2.115477217 | 0.0172562 |
| *Ldlr* | 2.000000000 | 0.045500264 | *Sbf2* | 1.459431619 | 0.045018495 | *Mrtfb* | -2.169925001 | 0.009234954 |
| *Ireb2* | 2.000000000 | 0.045500264 | *Nemf* | 1.459431619 | 0.045018495 | *Drg1* | -2.169925001 | 0.023342202 |
| *Gna11* | 2.000000000 | 0.045500264 | *Dicer1* | 1.459431619 | 0.05056115 | *Dpy19l4* | -2.169925001 | 0.044144307 |
| *Cep68* | 2.000000000 | 0.045500264 | *Cdkal1* | 1.440572591 | 0.01195717 | *Celf6* | -2.169925001 | 0.044144307 |
| *Med1* | 2.000000000 | 0.045500264 | *Pdia3* | 1.432959407 | 0.015988033 | *Gm48678* | -2.169925001 | 0.044144307 |
| *Sfxn1* | 2.000000000 | 0.045500264 | *Srrm3* | 1.415037499 | 0.012202907 | *Smad2* | -2.169925001 | 0.044144307 |
| *Btf3* | 2.000000000 | 0.045500264 | *Ogdh* | 1.415037499 | 0.021841403 | *Cul4b* | -2.169925001 | 0.044144307 |
| *Ift88* | 2.000000000 | 0.045500264 | *Isl1* | 1.378511623 | 0.033485286 | *Epc1* | -2.222392421 | 0.033375421 |
| *Hnrnpa1* | 2.000000000 | 0.045500264 | *Akap11* | 1.362570079 | 0.049495187 | *Rp9* | -2.321928095 | 0.022663096 |
| *Zdhhc23* | 2.000000000 | 0.045500264 | *Cystm1* | 1.353636955 | 0.043291923 | *Prkar1b* | -2.321928095 | 0.023342202 |
| *Arl13b* | 2.000000000 | 0.045500264 | *Relch* | 1.353636955 | 0.051334816 | *Pafah2* | -2.321928095 | 0.041673046 |
| *Strn* | 2.000000000 | 0.045500264 | *Kcnh7* | 1.349584438 | 0.024014338 | *Tmem209* | -2.321928095 | 0.041673046 |
| *Drap1* | 2.000000000 | 0.045500264 | *Rpl41* | 1.333423734 | 0.006982782 | *Dennd11* | -2.321928095 | 0.041673046 |
| *Taf1* | 2.000000000 | 0.045500264 | *Nfasc* | 1.321928095 | 0.010711814 | *C2cd3* | -2.321928095 | 0.041673046 |
| *Rbfox1* | 1.95419631 | 0.041557423 | *Pja2* | 1.321928095 | 0.023031789 | *Gcdh* | -2.321928095 | 0.041673046 |
| *Myo5a* | 1.925999419 | 0.007373513 | *Hnrnpa2b1* | 1.321928095 | 0.024647874 | *Ddx19b* | -2.321928095 | 0.041673046 |
| *Zfp462* | 1.874469118 | 0.017414791 | *Tlcd4* | 1.321928095 | 0.031060299 | *Kif5a* | -2.321928095 | 0.041673046 |
| *Cep170* | 1.874469118 | 0.019252851 | *Sgip1* | 1.321928095 | 0.035951689 | *Actr1a* | -2.321928095 | 0.041673046 |
| *Prdx1* | 1.874469118 | 0.028039738 | *Ndufb11* | 1.321928095 | 0.053970857 | *Clybl* | -2.415037499 | 0.016268425 |
| *Echdc3* | 1.874469118 | 0.037214299 | *Rims2* | 1.297680549 | 0.003169487 | *Ezh1* | -2.584962501 | 0.021318647 |
| *Stk39* | 1.874469118 | 0.037214299 | *Ppm1h* | 1.295455884 | 0.045723923 | *Zeb2* | -2.584962501 | 0.041603056 |
| *Nell1* | 1.847996907 | 0.04489975 | *Eif4g2* | 1.263034406 | 0.046493887 | *Phc3* | -2.584962501 | 0.041603056 |
| *Nme2* | 1.807354922 | 0.021775401 | *Rpl39* | 1.2410081 | 0.04670044 | *Ctso* | -2.584962501 | 0.041603056 |
| *Gorasp2* | 1.807354922 | 0.039026805 | *Cep350* | 1.222392421 | 0.041900594 | *Slc25a51* | -2.584962501 | 0.041603056 |
| *Hycc2* | 1.807354922 | 0.047466515 | *Camta1* | 1.182864057 | 0.001355598 | *Tmem245* | -2.584962501 | 0.041603056 |
| *Ssr3* | 1.807354922 | 0.047466515 | *Cblb* | 1.115477217 | 0.035376856 | *Ndufs5* | -2.584962501 | 0.041603056 |
| *Mrpl1* | 1.807354922 | 0.047466515 | *Ssh2* | 1.070389328 | 0.038882505 | *Gm12216* | -2.584962501 | 0.041603056 |
| *Pfdn1* | 1.807354922 | 0.047466515 | *Pde1c* | 1 | 0.05091422 | *Polk* | -2.584962501 | 0.041603056 |
| *Ehmt1* | 1.765534746 | 0.042921058 | *Csnk1a1* | 0.932885804 | 0.044087977 | *Epha6* | -2.584962501 | 0.041603056 |
| *Marchf4* | 1.765534746 | 0.047508686 | *Ubn2* | 0.91753784 | 0.041927946 | *Ypel5* | -2.584962501 | 0.041603056 |
| *mt-Nd3* | 1.736965594 | 0.009507189 | *Pcnx* | 0.906890596 | 0.054548798 | *Mcts1* | -2.584962501 | 0.041603056 |
| *Ptges3* | 1.736965594 | 0.037214299 | *Ube2b* | 0.893084796 | 0.020702878 | *Rbmx* | -2.807354922 | 0.010820254 |
| *Prkag2* | 1.736965594 | 0.052411791 | *Nktr* | 0.713695815 | 0.039757657 | *Sh3bgrl* | -2.807354922 | 0.010820254 |
| *Setx* | 1.716207034 | 0.007603344 | *Nsmce2* | 0.710493383 | 0.039006694 | *Bsn* | -2.807354922 | 0.021228114 |
| *Btg1* | 1.700439718 | 0.023067785 | *Nr6a1* | 0.607682577 | 0.048650985 | *Cxcl14* | -2.807354922 | 0.021228114 |
| *Slc39a11* | 1.700439718 | 0.023067785 | *Farp1* | 0.602664502 | 0.046951156 | *Wwp1* | -2.807354922 | 0.041655546 |
| *Hmbox1* | 1.700439718 | 0.024998354 |  |  |  | *4930512B01Rik* | -2.807354922 | 0.041655546 |
| *Gnb1* | 1.662965013 | 0.049259949 | *Cacnb2* | -0.575312331 | 0.040380039 | *Mrps30* | -2.807354922 | 0.041655546 |
| *Cdk12* | 1.584962501 | 0.006900895 | *Syt1* | -0.854802084 | 0.009638922 | *Zfp397* | -2.807354922 | 0.041655546 |
| *Usp47* | 1.584962501 | 0.009931474 | *Tenm2* | -1 | 0.043978503 | *Ulk2* | -3.000000000 | 0.010726726 |
| *Eps15l1* | 1.584962501 | 0.017535797 | *Shroom3* | -1.087462841 | 0.040101604 | *Prr14* | -3.000000000 | 0.021228114 |
| *Smc5* | 1.584962501 | 0.024392607 | *Dock1* | -1.144389909 | 0.0232848 | *Sema3a* | -3.000000000 | 0.021282416 |
| *Add1* | 1.584962501 | 0.02991742 | *Maml2* | -1.152003093 | 0.013459565 | *Tfg* | -3.169925001 | 0.010726726 |
| *Bhlhb9* | 1.584962501 | 0.02991742 | *Tenm1* | -1.192645078 | 0.045307707 | *Ociad1* | -3.169925001 | 0.021309587 |
| *Ttc28* | 1.584962501 | 0.030163099 | *Prl* | -1.256117544 | 0.022215245 | *Hdgfl3* | -3.169925001 | 0.021309587 |
| *Tra2b* | 1.584962501 | 0.031731791 | *Nsd1* | -1.299560282 | 0.040661874 | *Etv1* | -3.169925001 | 0.041655546 |
| *Cdc40* | 1.584962501 | 0.040436838 | *Hsp90aa1* | -1.523561956 | 0.051354634 | *Tmem176a* | -3.321928095 | 0.021300529 |
|  |  |  | *Pcdh9* | -1.584962501 | 0.013220224 | *Gm12353* | -3.459431619 | 0.010773441 |
|  |  |  | *Hmgn3* | -1.584962501 | 0.042617619 | *Grm5* | -3.807354922 | 0.021300529 |

| Supplemental Table S13. Differentially Expressed Genes Representing Estrogen Receptor Signaling in Thyrotropes and 2 Lactotrope clusters: VCD vs Vehicle control females | | | | | | | | |
| --- | --- | --- | --- | --- | --- | --- | --- | --- |
|  |  | **Thyrotropes** | | **Lactotrope 2 (LAC2)** | | | **Lactotrope 4 (LAC4)** | |
|  | **Gene** | **Expr Log Ratio** | **Expr p-value** | **Expr Log Ratio** | | **Expr p-value** | **Expr Log Ratio** | **Expr p-value** |
| G protein subunit alpha 11 | Gna11 | 2 | 0.0455 | 0.8 | 0.000227 | |  |  |
| G protein subunit beta 1 | Gnb1 | 1.663 | 0.0493 |  |  | |  |  |
| heat shock protein 90 alpha family class A member 1 | Hsp90aa1 | -1.524 | 0.0514 |  |  | |  |  |
| insulin like growth factor 2 receptor | Igf2r | 2.585 | 0.0416 | 0.81 | 0.0103 | |  |  |
| Janus kinase 2 | Jak2 | 3 | 0.0417 | 0.926 | 0.0052 | |  |  |
| mediator complex subunit 1 | Med1 | 2 | 0.0455 |  |  | |  |  |
| NADH:ubiquinone oxidoreductase subunit B11 | Ndufb11 | 1.322 | 0.054 |  |  | |  |  |
| NADH:ubiquinone oxidoreductase subunit S5 | Ndufs5 | -2.585 | 0.0416 |  |  | |  |  |
| protein disulfide isomerase family A member 3 | Pdia3 | 1.433 | 0.016 |  |  | |  |  |
| protein kinase AMP-activated non-catalytic subunit gamma 2 | Prkag2 | 1.737 | 0.0524 |  |  | | 2.957 | 0.0233 |
| protein kinase cAMP-dependent type I regulatory subunit beta | Prkar1b | -2.322 | 0.0233 |  |  | |  |  |
| protein kinase cAMP-dependent type II regulatory subunit alpha | Prkar2a | 2.322 | 0.0417 | 0.755 | 0.00205 | |  |  |
| protein kinase C beta | Prkcb | -1.766 | 0.0431 |  |  | |  |  |
| tumor protein p53 | Trp53 | 2.322 | 0.0417 |  |  | |  |  |
| ubiquinol-cytochrome c reductase core protein 2 | Uqcrc2 | 3.322 | 0.00538 |  |  | |  |  |

| **Supplemental Table S14A Differentially Expressed 17-β Estradiol Target Genes in Thyrotropes and 3 Lactotrope clusters**  **VCD vs Vehicle control females** | | | | | | | | | |
| --- | --- | --- | --- | --- | --- | --- | --- | --- | --- |
|  |  | **Thyrotropes** | | **LAC1** | | **LAC2** | | **LAC3** | |
|  | **Gene** | **Expr Log Ratio** | **Expr p-value** | **Expr Log Ratio** | **Expr p-value** | **Expr Log Ratio** | **Expr p-value** | **Expr Log Ratio** | **Expr p-value** |
| ALG1 chitobiosyldiphosphodolichol beta-mannosyltransferase | *Alg1* |  |  | -1.319 | 0.0485 |  |  |  |  |
| ATP binding cassette subfamily C member 5 | *ABCC5* |  |  |  |  |  |  | 0.781 | 0.0144 |
| AE binding protein 1 | *AEBP1* |  |  |  |  |  |  | 0.718 | 0.047 |
| ALK receptor tyrosine kinase | *ALK* |  |  |  |  |  |  |  |  |
| anaphase promoting complex subunit 4 | *ANAPC4* |  |  |  |  |  |  |  |  |
| annexin A1 | *Anxa1* |  |  |  |  |  |  | -2.164 | 0.0362 |
| annexin A2 | *Anxa2* |  |  | 1.242 | 0.00654 | 0.952 | 0.012 |  |  |
| annexin A4 | *Anxa4* |  |  | 1.215 | 0.0139 | 0.833 | 0.0157 |  |  |
| **activating transcription factor 3** | ***ATF3*** | 2.392 | 0.00497 | 0.917 | 0.0000231 | 0.775 | 0.00000014 | 1.368 | 0.00000156 |
| ataxin 3 | *Atxn3* |  |  | 0.972 | 0.0196 | -1.048 | 0.0505 |  |  |
| **ATPase H+ transporting accessory protein 1** | ***ATP6AP1*** | 2.17 | 0.0233 |  |  | 0.77 | 0.00436 |  |  |
| RIKEN cDNA 4930570G19 gene | *4930570G19Rik* |  |  |  |  | -1.017 | 0.0146 |  |  |
| argonaute RISC catalytic component 2 | *AGO2* |  |  |  |  | 0.591 | 0.000248 | 0.577 | 0.00151 |
| aldehyde dehydrogenase 2 family member | *ALDH2* |  |  |  |  | 1.138 | 0.0113 | -1.736 | 0.00362 |
| adaptor related protein complex 1 subunit sigma 1 | *AP1S1* |  |  |  |  | 0.758 | 0.0135 |  |  |
| apolipoprotein E | *APOE* |  |  |  |  | 2.127 | 0.0309 |  |  |
| activity regulated cytoskeleton associated protein | *ARC* |  |  |  |  | 0.992 | 0.000235 |  |  |
| ARF like GTPase 4A | *ARL4A* |  |  |  |  | 0.645 | 0.0202 |  |  |
| acidic residue methyltransferase 1 | *ARMT1* |  |  |  |  |  |  | -1.48 | 0.0523 |
| arylsulfatase A | *ARSA* |  |  |  |  |  |  | -1.673 | 0.0135 |
| actin related protein 2/3 complex subunit 1B | *ARPC1B* |  |  |  |  | 2.797 | 0.00409 | -1.373 | 0.0517 |
| ATPase plasma membrane Ca2+ transporting 1 | *ATP2B1* |  |  |  |  | 0.675 | 0.00000219 |  |  |
| ATPase plasma membrane Ca2+ transporting 2 | *ATP2B2* |  |  |  |  | 0.999 | 0.0273 |  |  |
| ataxin 3 | *ATXN3* |  |  |  |  | -1.048 | 0.0505 |  |  |
| beta-1,4-galactosyltransferase 5 | *B4GALT5* |  |  |  |  | 1.546 | 0.00738 |  |  |
| beta-secretase 1 | *BACE1* |  |  |  |  | 1.122 | 0.00501 |  |  |
| basic helix-loop-helix family member e40 | *BHLHE40* |  |  |  |  |  |  | 0.704 | 0.0236 |
| bone morphogenetic protein 6 | *BMP6* |  |  |  |  |  |  | 2.479 | 0.0272 |
| bromodomain containing 4 | *BRD4* |  |  |  |  |  |  | 0.615 | 0.00107 |
| BTG anti-proliferation factor 1 | *BTG1* |  |  |  |  |  |  | 0.827 | 0.0137 |
| BCL2 associated X, apoptosis regulator | *BAX* |  |  |  |  | 0.599 | 0.0479 |  |  |
| bridging integrator 1 | *BIN1* |  |  |  |  | 0.874 | 0.00556 |  |  |
| baculoviral IAP repeat-containing 3 | *Birc3* |  |  |  |  | 2.698 | 0.000447 |  |  |
| biogenesis of lysosomal organelles complex 1 subunit 6 | *BLOC1S6* |  |  |  |  | 1.475 | 0.0115 |  |  |
| BRCA2 DNA repair associated | *BRCA2* |  |  |  |  |  |  |  |  |
| BCL10 immune signaling adaptor | *Bcl10* |  |  | 1.283 | 0.0482 |  |  |  |  |
| BCL2 like 11 | *Bcl2l11* |  |  | 0.718 | 0.0123 |  |  |  |  |
| baculoviral IAP repeat-containing 3 | *Birc3* |  |  | 1.406 | 0.0315 | 2.698 | 0.000447 |  |  |
| BTG anti-proliferation factor 1 | *BTG1* | 1.7 | 0.0231 |  |  |  |  |  |  |
| calcium voltage-gated channel subunit alpha1 G | *Cacna1g* |  |  | 0.761 | 0.00167 | 0.587 | 0.0132 |  |  |
| carbamoyl-phosphate synthetase 2, aspartate transcarbamylase, and dihydroorotase | *Cad* |  |  | 1.669 | 0.00972 |  |  |  |  |
| calreticulin | *Calr* |  |  | 0.591 | 0.00798 |  |  |  |  |
| cellular communication network factor 1 | *Ccn1* |  |  | 0.938 | 0.000845 | 0.954 | 0.00005 |  |  |
| cyclin G1 | *CCNG1* |  |  |  |  | 0.89 | 0.00759 |  |  |
| CD24 molecule | *CD24* |  |  |  |  | 0.62 | 0.032 |  |  |
| CD68 molecule | *CD68* |  |  |  |  | 2.06 | 0.039 |  |  |
| CDC42 binding protein kinase alpha | *CDC42BPA* |  |  |  |  | 0.614 | 0.000682 |  |  |
| chromatin licensing and DNA replication factor 1 | *CDT1* |  |  |  |  | 2.06 | 0.039 |  |  |
| CFAP97 domain containing 2 | *CFAP97D2* |  |  |  |  | 1.138 | 0.0402 |  |  |
| checkpoint kinase 1 | *CHEK1* |  |  |  |  | 1.89 | 0.0283 |  |  |
| Cbp/p300 interacting transactivator with Glu/Asp rich carboxy-terminal domain 1 | *CITED1* |  |  | 2.184 | 0.00954 | 0.738 | 0.0549 |  |  |
| claudin 23 | *CLDN23* |  |  |  |  | 1.475 | 0.0179 |  |  |
| cytochrome c oxidase assembly factor 5 | *COA5* |  |  |  |  | -1.525 | 0.0417 |  |  |

| **Supplemental Table S14B Differentially Expressed 17-β Estradiol Target Genes in Thyrotropes and 3 Lactotrope clusters**  **VCD vs Vehicle control females** | | | | | | | | | |
| --- | --- | --- | --- | --- | --- | --- | --- | --- | --- |
|  |  | **Thyrotropes** | | **LAC1** | | **LAC2** | | **LAC3** | |
|  | **Gene** | **Expr Log Ratio** | **Expr p-value** | **Expr Log Ratio** | **Expr p-value** | **Expr Log Ratio** | **Expr p-value** | **Expr Log Ratio** | **Expr p-value** |
| cytochrome c oxidase assembly factor COX20 | *COX20* |  |  |  |  | 0.627 | 0.0523 |  |  |
| cytochrome c oxidase subunit 7A2 | *COX7A2* |  |  |  |  | 0.625 | 0.00000112 |  |  |
| **cAMP responsive element binding protein 3 like 1** | ***CREB3L1*** |  |  | 0.938 | 4.65E-08 | 1.304 | 3.93E-26 | 0.873 | 0.0000293 |
| cAMP responsive element binding protein 3 like 2 | *CREB3L2* |  |  |  |  | 0.657 | 0.00351 |  |  |
| CRK like proto-oncogene, adaptor protein | *CRKL* |  |  |  |  | 0.996 | 0.0118 |  |  |
| cytokine receptor like factor 1 | *CRLF1* |  |  |  |  | 1.645 | 0.0371 |  |  |
| chromosome segregation 1 like | *CSE1L* |  |  |  |  | 0.755 | 0.0249 |  |  |
| CXADR Ig-like cell adhesion molecule | *CXADR* |  |  |  |  | 0.945 | 0.0346 |  |  |
| cytochrome b561 | *CYB561* |  |  |  |  | 0.859 | 0.0376 |  |  |
| CD74 molecule | *CD74* |  |  |  |  |  |  | 1.467 | 0.0541 |
| CD83 molecule | *CD83* |  |  |  |  |  |  | 2.55 | 0.0486 |
| CD86 molecule | *CD86* |  |  |  |  |  |  | 3.202 | 0.0197 |
| cell division cycle 42 | *CDC42* |  |  |  |  |  |  | 0.685 | 0.00154 |
| cyclin dependent kinase inhibitor 1A | *CDKN1A* |  |  |  |  |  |  | 2.742 | 0.00807 |
| cerebellar degeneration related antigen 1, opposite strand | *Cdr1os* |  |  |  |  |  |  | -0.641 | 0.016 |
| CCAAT enhancer binding protein gamma | *CEBPG* |  |  |  |  |  |  | 0.927 | 0.0308 |
| claudin 6 | *CLDN6* |  |  |  |  |  |  | 1.327 | 0.0357 |
| cathepsin B | *CTSB* |  |  |  |  |  |  | 0.667 | 0.0241 |
| cytochrome P450 family 51 subfamily A member 1 | *CYP51A1* |  |  |  |  |  |  | 1.807 | 0.000755 |
| cell division cycle 23 | *Cdc23* |  |  | 0.849 | 0.0468 |  |  |  |  |
| cerebellar degeneration related protein 2 | *Cdr2* |  |  | 0.92 | 0.00518 |  |  |  |  |
| chromosome 1 open reading frame 226 | *C1of 226* |  |  | 2.184 | 0.0387 |  |  |  |  |
| cholinergic receptor muscarinic 3 | *Chrm3* |  |  | 2.505 | 0.0208 |  |  |  |  |
| **citramalyl-CoA lyase** | ***CLYBL*** | -2.415 | 0.0163 | 0.59 | 0.0000929 |  |  |  |  |
| creatine kinase B | *CKB* | 3.585 | 0.00265 |  |  |  |  |  |  |
| capicua transcriptional repressor | *Cic* |  |  | 0.664 | 0.0357 |  |  |  |  |
| crooked neck pre-mRNA splicing factor 1 | *Crnkl1* |  |  | 0.742 | 0.00865 |  |  |  |  |
| crystallin zeta | *Cryz* |  |  | -1.028 | 0.0266 |  |  |  |  |
| cullin 4A | *CUL4A* | 2.7 | 0.00545 |  |  |  |  |  |  |
| cyclin dependent kinase 5 regulatory subunit 1 | *Cdk5r1* |  |  | -1.454 | 0.0245 |  |  |  |  |
| ***cytochrome c, somatic*** | ***Cycs*** |  |  | 1.938 | 0.000663 | 1.037 | 0.000382 |  |  |
| cytochrome b5 type A | *CYB5A* | 2.459 | 0.022 |  |  |  |  |  |  |
| cysteine rich transmembrane module containing 1 | *CYSTM1* | 1.354 | 0.0433 |  |  |  |  |  |  |
| dedicator of cytokinesis 1 | *DOCK1* | -1.144 | 0.0233 |  |  |  |  |  |  |
| developmentally regulated GTP binding protein 1 | *DRG1* | -2.17 | 0.0233 |  |  |  |  |  |  |
| dimethylarginine dimethylaminohydrolase 1 | ***Ddah1*** |  |  | 0.673 | 0.0326 | 0.772 | 0.00386 | 0.655 | 0.00177 |
| discoidin domain receptor tyrosine kinase 1 | *DDR1* |  |  |  |  | 0.881 | 0.0111 |  |  |
| DNA methyltransferase 1 | *DNMT1* |  |  | 0.731 | 0.0116 | 0.66 | 0.0286 |  |  |
| DOT1 like histone lysine methyltransferase | *DOT1L* |  |  | 0.949 | 0.000173 | 0.92 | 0.00323 | 0.912 | 0.00511 |
| dual specificity phosphatase 5 | *DUSP5* |  |  | 4.353 | 0.00072 | 1.854 | 0.0128 |  |  |
| dual specificity phosphatase 10 | *DUSP10* |  |  |  |  | 0.765 | 0.0284 |  |  |
| derlin 1 | *Derl1* |  |  | 0.7 | 0.00201 |  |  |  |  |
| dolichyl-phosphate mannosyltransferase subunit 3, regulatory | *Dpm3* |  |  | 0.786 | 0.0265 |  |  |  |  |
| DAB adaptor protein 2 | *DAB2* |  |  |  |  |  |  | 2.327 | 0.0486 |
| dematin actin binding protein | *DMTN* |  |  |  |  |  |  | -1.046 | 0.0307 |
| dynein light chain LC8-type 1 | *DYNLL1* |  |  |  |  | 0.525 | 0.000693 | 0.752 | 0.0000607 |

| **Supplemental Table S14C Differentially Expressed 17-β Estradiol Target Genes in Thyrotropes and 3 Lactotrope clusters**  **VCD vs Vehicle control females** | | | | | | | | | |
| --- | --- | --- | --- | --- | --- | --- | --- | --- | --- |
|  |  | **Thyrotropes** | | **LAC1** | | **LAC2** | | **LAC3** | |
|  | **Gene** | **Expr Log Ratio** | **Expr p-value** | **Expr Log Ratio** | **Expr p-value** | **Expr Log Ratio** | **Expr p-value** | **Expr Log Ratio** | **Expr p-value** |
| enoyl-CoA hydratase domain containing 2 | *ECHDC2* |  |  |  |  | 1.153 | 0.0383 |  |  |
| early growth response 2 | *EGR2* |  |  |  |  | 1.475 | 0.0281 |  |  |
| early growth response 4 | *EGR4* |  |  |  |  | 2.645 | 0.000157 | 1.944 | 0.00252 |
| ETS transcription factor ELK3 | *ELK3* |  |  |  |  | 0.656 | 0.00149 |  |  |
| ectodermal-neural cortex 1 | *ENC1* |  |  |  |  | 0.982 | 0.0397 |  |  |
| ectonucleotide pyrophosphatase/phosphodiesterase 2 | *ENPP2* |  |  |  |  | -2.525 | 0.0245 |  |  |
| ETS variant transcription factor 5 | *ETV5* |  |  |  |  | 1.575 | 0.00466 |  |  |
| ETS variant transcription factor 6 | *ETV6* |  |  |  |  | 0.773 | 0.0138 |  |  |
| EWS RNA binding protein 1 | *EWSR1* |  |  |  |  | 0.873 | 0.00103 |  |  |
| ETS variant transcription factor 1 | *ETV1* | **-3.17** | **0.0417** |  |  |  |  |  |  |
| EGF containing fibulin extracellular matrix protein 1 | *Efemp1* |  |  | 0.636 | 0.0124 |  |  | 0.674 | 0.00827 |
| EGF containing fibulin extracellular matrix protein 1 | *EFEMP1* |  |  |  |  |  |  | 0.674 | 0.00827 |
| ephrin A1 | *EFNA1* |  |  |  |  |  |  | 1.421 | 0.033 |
| eukaryotic translation initiation factor 3 subunit G | *EIF3G* |  |  |  |  |  |  | 0.655 | 0.0441 |
| epithelial membrane protein 1 | *EMP1* |  |  |  |  |  |  | 1.627 | 0.0361 |
| enolase 1 | *ENO1* |  |  |  |  |  |  | 1.245 | 0.0507 |
| ERH mRNA splicing and mitosis factor | *ERH* |  |  |  |  |  |  | -0.958 | 0.0153 |
| eukaryotic translation initiation factor 3 subunit B | *Eif3b* |  |  | 0.968 | 0.0477 |  |  | -1.373 | 0.00442 |
| ELAV like RNA binding protein 1 | *Elavl1* |  |  | 0.645 | 0.00664 | 0.585 | 0.0328 |  |  |
| enhancer of zeste 2 polycomb repressive complex 2 subunit | *Ezh2* |  |  | 2.254 | 0.00462 |  |  |  |  |
| ezrin | *Ezr* |  |  | 0.599 | 0.00449 |  |  | 0.961 | 0.00019 |
| phenylalanyl-tRNA synthetase subunit beta | *FARSB* |  |  |  |  | -0.975 | 0.0153 |  |  |
| farnesyl-diphosphate farnesyltransferase 1 | *FDFT1* |  |  |  |  | 1.191 | 0.0105 |  |  |
| fibroblast growth factor receptor 2 | *FGFR2* |  |  |  |  | 0.728 | 0.0303 |  |  |
| four and a half LIM domains 1 | *FHL1* |  |  | 1.143 | 0.0131 | 1.143 | 0.00447 |  |  |
| filamin B | *FLNB* |  |  |  |  | 0.859 | 0.0271 |  |  |
| forkhead box O1 | *FOXO1* |  |  |  |  | 0.673 | 0.0344 |  |  |
| fibroblast growth factor receptor substrate 2 | *FRS2* |  |  |  |  | 0.698 | 0.0104 |  |  |
| FERM, ARH/RhoGEF and pleckstrin domain protein 1 | *FARP1* | 0.603 | 0.047 |  |  |  |  |  |  |
| FosB proto-oncogene, AP-1 transcription factor subunit | *Fosb* |  |  | 0.761 | 3.26E-08 | 0.888 | 8.5E-26 | 0.61 | 4.26E-10 |
| FOS like 1, AP-1 transcription factor subunit | *Fosl1* |  |  | 1.991 | 0.0388 |  |  | 2.157 | 0.0274 |
| F-box protein 46 | *FBXO46* |  |  |  |  |  |  | 2.202 | 0.00226 |
| fibroblast growth factor receptor 2 | *FGFR2* |  |  |  |  |  |  | 0.615 | 0.0514 |
| forkhead box O3 | *FOXO3* |  |  |  |  |  |  | 0.801 | 0.00796 |
| follicle stimulating hormone subunit beta | *Fshb* |  |  | -1.567 | 0.00197 |  |  |  |  |
| frizzled class receptor 8 | *Fzd8* |  |  | 2.353 | 0.0387 |  |  |  |  |
| FYN proto-oncogene, Src family tyrosine kinase | *FYN* | -1.7 | 0.0437 |  |  |  |  |  |  |
| **glucose-6-phosphate dehydrogenase** | ***G6PD*** | 2.807 | 0.0417 |  |  | 0.732 | 0.0121 |  |  |
| glucosidase II alpha subunit | *GANAB* |  |  |  |  | 0.738 | 0.0245 |  |  |
| galactosidase alpha | *GLA* |  |  |  |  | 1.212 | 0.0157 |  |  |
| glutaminase | *GLS* |  |  | 0.615 | 0.00542 | 0.935 | 0.0103 |  |  |
| predicted gene, 20125 | *Gm20125* |  |  |  |  | -0.674 | 0.0032 |  |  |
| predicted gene, 32014 | *Gm32014* |  |  |  |  | 0.89 | 0.0265 |  |  |
| GIPC PDZ domain containing family member 2 | *GIPC2* |  |  |  |  |  |  | 1.55 | 0.0278 |
| glycerol kinase | *GK* |  |  |  |  |  |  | -1.328 | 0.0446 |
| predicted gene, 20125 | *Gm20125* |  |  |  |  | -0.674 | 0.0032 | -0.726 | 0.0136 |
| N-acetylglucosamine-1-phosphate transferase subunits alpha and beta | *GNPTAB* |  |  |  |  | 1.668 | 0.00557 |  |  |

| **Supplemental Table S14D Differentially Expressed 17-β Estradiol Target Genes in Thyrotropes and 3 Lactotrope clusters**  **VCD vs Vehicle control females** | | | | | | | | | |
| --- | --- | --- | --- | --- | --- | --- | --- | --- | --- |
|  |  | **Thyrotropes** | | **LAC1** | | **LAC2** | | **LAC3** | |
|  | **Gene** | **Expr Log Ratio** | **Expr p-value** | **Expr Log Ratio** | **Expr p-value** | **Expr Log Ratio** | **Expr p-value** | **Expr Log Ratio** | **Expr p-value** |
| golgi SNAP receptor complex member 2 | *GOSR2* |  |  |  |  | 0.68 | 0.0092 |  |  |
| growth factor receptor bound protein 7 | *GRB7* |  |  |  |  | 1.35 | 0.0499 |  |  |
| glutamate ionotropic receptor NMDA type subunit 1 | *GRIN1* |  |  |  |  | 0.861 | 0.00133 |  |  |
| glutathione-disulfide reductase | *GSR* |  |  |  |  | 1.06 | 0.0267 |  |  |
| glutamate dehydrogenase 1 | *Glud1* |  |  | 0.833 | 0.00493 |  |  |  |  |
| G protein-coupled receptor 171 | *Gpr171* |  |  | 1.669 | 0.023 |  |  |  |  |
| GTF2I repeat domain containing 1 | *Gtf21rd1* |  |  | 0.583 | 0.000108 |  |  |  |  |
| **G protein subunit alpha 11** | ***GNA11*** | 2 | 0.0455 |  |  | 0.8 | 0.000227 |  |  |
| G protein subunit beta 1 | *GNB1* | 1.663 | 0.0493 |  |  |  |  |  |  |
| G protein-coupled receptor kinase 3 | *GRK3* | 2.807 | 0.0417 |  |  |  |  |  |  |
| glutamate metabotropic receptor 5 | *GRM5* | -3.807 | 0.0213 |  |  |  |  |  |  |
| homocysteine inducible ER protein with ubiquitin like domain 1 | *HERPUD1* |  |  |  |  | 0.787 | 0.0032 | 0.687 | 0.0285 |
| HIG1 hypoxia inducible domain family member 1A | *HIGD1A* |  |  |  |  | 0.506 | 0.0523 | 1.157 | 0.00568 |
| major histocompatibility complex, class I, A | *HLA-A* |  |  | 1.032 | 0.00000152 | 0.928 | 0.0414 | 2.742 | 0.0486 |
| hemicentin 2 | *HMCN2* |  |  | 0.961 | 0.0114 | 2.401 | 0.00182 | 1.742 | 0.0201 |
| 3-hydroxy-3-methylglutaryl-CoA synthase 1 | *HMGCS1* | 2.459 | 0.022 |  |  |  |  | 1.032 | 0.0354 |
| histone deacetylase 5 | *HDAC5* |  |  |  |  | 0.831 | 0.00223 |  |  |
| homer scaffold protein 1 | *HOMER1* |  |  | 1.151 | 0.000212 | 1.279 | 1.29E-10 | 1.406 | 0.00000291 |
| heat shock protein family H (Hsp110) member 1 | *HSPH1* |  |  | 0.724 | 0.000684 | 0.843 | 0.00308 | 0.769 | 0.00185 |
| HIVEP zinc finger 1 | *HIVEP1* |  |  |  |  | 0.707 | 0.00973 |  |  |
| HCLS1 binding protein 3 | *HS1BP3* |  |  |  |  | 1.253 | 0.0543 |  |  |
| heat shock protein family A (Hsp70) member 5 | *HSPA5* |  |  | 0.676 | 0.00852 | 0.807 | 3.4E-09 | 0.525 | 0.00000637 |
| heat shock protein family A (Hsp70) member 9 | *HSPA9* | 2.322 | 0.0428 |  |  |  |  |  |  |
| heterogeneous nuclear ribonucleoprotein A1 | *HNRNPA1* | 2 | 0.0455 |  |  |  |  |  |  |
| histone deacetylase 4 | *Hdac4* |  |  | 0.744 | 0.0000317 |  |  |  |  |
| high density lipoprotein binding protein | *Hdlbp* |  |  | 0.606 | 0.000453 |  |  |  |  |
| major histocompatibility complex, class I, A | *H2-Q6* |  |  | 1.032 | 0.00000152 | 0.928 | 0.0414 | 2.742 | 0.0486 |
| heat shock transcription factor 1 | *Hsf1* |  |  | 0.698 | 0.0476 |  |  |  |  |
| 5-hydroxytryptamine receptor 7 | *Htr7* |  |  | 2.184 | 0.0387 | 0.843 | 0.00308 | 0.769 | 0.00185 |
| interferon induced protein 35 | *IFI35* |  |  |  |  |  |  | -1.042 | 0.0238 |
| immunoglobulin superfamily member 1 | *IGSF1* |  |  |  |  |  |  | 0.904 | 0.00222 |
| interleukin 13 receptor subunit alpha 1 | *IL13RA1* |  |  |  |  |  |  | 0.974 | 0.019 |
| inositol polyphosphate-4-phosphatase type II B | *INPP4B* |  |  |  |  |  |  | 3.121 | 0.0344 |
| insulin receptor substrate 2 | *IRS2* |  |  | 0.594 | 0.000184 | 0.697 | 0.00000123 | 0.632 | 0.000651 |
| inositol-tetrakisphosphate 1-kinase | *ITPK1* |  |  |  |  |  |  | 0.632 | 0.0202 |
| insulin degrading enzyme | *IDE* |  |  |  |  |  |  |  |  |
| interferon related developmental regulator 1 | *IFRD1* |  |  | 0.579 | 0.000206 | 0.811 | 0.00823 |  |  |
| interferon related developmental regulator 2 | *IFRD2* |  |  |  |  | 0.813 | 0.0000555 |  |  |
| immunoglobulin superfamily member 1 | *IGSF1* |  |  |  |  | 0.81 | 0.0103 | 0.904 | 0.00222 |
| IQ motif containing GTPase activating protein 1 | *IQGAP1* |  |  |  |  | 0.656 | 0.0349 |  |  |
| integrin subunit alpha 9 | *ITGA9* |  |  |  |  | 0.697 | 0.00000123 |  |  |
| integrin subunit alpha V | *ITGAV* |  |  |  |  | 1.23 | 0.011 |  |  |
| **insulin like growth factor 2 receptor** | ***IGF2R*** | 2.585 | 0.0416 |  |  | 1.1 | 0.000000791 |  |  |
| interleukin 17 receptor D | *IL17RD* | 2.322 | 0.0417 |  |  |  |  |  |  |

| **Supplemental Table S14E Differentially Expressed 17-β Estradiol Target Genes in Thyrotropes and 3 Lactotrope clusters**  **VCD vs Vehicle control females** | | | | | | | | | |
| --- | --- | --- | --- | --- | --- | --- | --- | --- | --- |
|  |  | **Thyrotropes** | | **LAC1** | | **LAC2** | | **LAC3** | |
|  | **Gene** | **Expr Log Ratio** | **Expr p-value** | **Expr Log Ratio** | **Expr p-value** | **Expr Log Ratio** | **Expr p-value** | **Expr Log Ratio** | **Expr p-value** |
| inositol polyphosphate-5-phosphatase F | *INPP5F* | 2.585 | 0.0213 |  |  |  |  |  |  |
| interleukin 1 receptor type 1 | *Il1r1* |  |  | -1.71 | 0.00995 |  |  |  |  |
| integrin subunit alpha 11 | *Itga11* |  |  | 1.884 | 0.00962 |  |  |  |  |
| integrin subunit beta 1 | *Itgb1* |  |  | 0.689 | 0.00255 |  |  |  |  |
| integrin subunit alpha 3 | *ITGA3* | 2.585 | 0.0416 |  |  |  |  |  |  |
| Janus kinase 2 | *JAK2* | 3 | 0.0417 |  |  | 0.926 | 0.0052 |  |  |
| Jun proto-oncogene, AP-1 transcription factor subunit | *Jun* |  |  | 0.651 | 0.0000986 | 0.829 | 7.84E-08 | 0.637 | 1.07E-08 |
| JunD proto-oncogene, AP-1 transcription factor subunit | *Jund* |  |  | 0.624 | 3.7E-13 | 0.539 | 0.00000359 |  |  |
| KLF transcription factor 4 | *KLF4* |  |  | 1.502 | 5.62E-09 | 1.504 | 7.49E-21 | 1.345 | 0.000000732 |
| potassium voltage-gated channel subfamily A regulatory beta subunit 1 | *Kcnab1* |  |  | 0.805 | 0.00322 |  |  | 0.581 | 0.0144 |
| lysine acetyltransferase 6A | *KAT6A* |  |  |  |  |  |  | 0.786 | 0.00427 |
| lysine demethylase 4B | *Kdm4b* |  |  | 0.582 | 0.05 |  |  |  |  |
| KLF transcription factor 2 | *Klf2* |  |  | 1.707 | 0.018 |  |  |  |  |
| KLF transcription factor 9 | *Klf9* |  |  | 0.729 | 0.046 | 1.504 | 7.49E-21 | 1.345 | 0.000000732 |
| kazrin, periplakin interacting protein | *Kazn* | 3 | 0.00247 |  |  |  |  |  |  |
| La ribonucleoprotein 6, translational regulator |  |  |  |  |  |  |  |  |  |
| long intergenic non-protein coding RNA, p53 induced transcript |  |  |  |  |  |  |  |  |  |
| LIM domain only 3 |  |  |  |  |  |  |  |  |  |
| LIM domain 7 |  |  |  |  |  |  |  |  |  |
| low density lipoprotein receptor | *LDLR* | 2 | 0.0455 |  |  |  |  |  |  |
| leucine rich repeat containing G protein-coupled receptor 4 | *LGR4* | 2.222 | 0.0173 | 0.884 | 0.0192 |  |  |  |  |
| lamin A/C | *Lmna* |  |  | 1.039 | 8.26E-08 | 0.541 | 0.00000248 | 0.715 | 0.000000409 |
| lin-7 homolog A, crumbs cell polarity complex component | *LIN7A* |  |  |  |  |  |  | 0.636 | 0.0226 |
| leucine rich repeat and Ig domain containing 2 | *LINGO2* |  |  |  |  |  |  | 0.677 | 0.00131 |
| lipin 1 | *LPIN1* |  |  |  |  |  |  | 1.212 | 0.000182 |
| lysosomal protein transmembrane 5 | *LAPTM5* |  |  |  |  |  |  | 1.742 | 0.0277 |
| mitogen-activated protein kinase kinase 2 | *MAP2K2* |  |  |  |  | 0.599 | 0.00311 |  |  |
| mitogen-activated protein kinase kinase 6 | *MAP2K6* |  |  |  |  | 1.212 | 0.0365 |  |  |
| mitogen-activated protein kinase 8 | *MAPK8* |  |  |  |  | 0.938 | 0.00642 |  |  |
| mitogen-activated protein kinase 14 | *MAPK14* |  |  |  |  |  |  | 0.674 | 0.0205 |
| myristoylated alanine rich protein kinase C substrate | *MARCKS* |  |  |  |  |  |  | -0.696 | 0.0525 |
| membrane integral NOTCH2 associated receptor 1 | *MINAR1* |  |  |  |  |  |  | 3.912 | 0.0015 |
| melanocortin 2 receptor accessory protein 2 | *MRAP2* |  |  |  |  | 1.167 | 0.0164 | -1.008 | 0.0228 |
| mitochondrial ribosomal protein L41 | *MRPL41* |  |  |  |  |  |  | 0.91 | 0.0098 |
| mitochondrial ribosomal protein S23 | *MRPS23* |  |  |  |  | 0.961 | 0.0181 | -1.373 | 0.00791 |
| mitochondrial ribosomal protein S27 | *MRPS27* |  |  |  |  |  |  | -1.373 | 0.0079 |
| methylsterol monooxygenase 1 | *MSMO1* |  |  |  |  |  |  | 0.825 | 0.0431 |
| metallothionein 2A | *MT2A* |  |  |  |  |  |  | 1.38 | 0.00175 |
| Meis homeobox 2 | *Meis2* |  |  | 0.595 | 0.0195 |  |  |  |  |
| alpha-1,3-mannosyl-glycoprotein 2-beta-N-acetylglucosaminyltransferase | *Mgat1* |  |  | 1.184 | 0.0154 |  |  |  |  |
| mitofusin 1 | *MFN1* |  |  |  |  | 1.085 | 0.0176 |  |  |
| microtubule associated monooxygenase, calponin and LIM domain containing 2 | *MICAL2* |  |  |  |  | 0.88 | 0.0098 |  |  |
| MIR670 host gene (non-protein coding) | *Mir670hg* |  |  |  |  | 0.699 | 0.00248 |  |  |
| miRNA containing gene | *Mirg* |  |  |  |  | 0.954 | 0.00000192 | 0.677 | 0.00179 |
| metallophosphoesterase domain containing 2 | *MPPED2* |  |  | 0.983 | 0.0166 | 1.06 | 0.00271 |  |  |
| mitochondrial ribosomal protein L52 | *MRPL52* |  |  |  |  | 0.748 | 0.0246 |  |  |
| mitochondrial ribosomal protein S12 | *MRPS12* |  |  |  |  | 0.62 | 0.0176 |  |  |
| MYB proto-oncogene, transcription factor | *MYB* |  |  |  |  |  |  | 1.479 | 0.0168 |
| myotubularin related protein 4 | *MTMR4* |  |  |  |  | 1.35 | 0.00571 |  |  |
| matrix remodeling associated 7 | *MXRA7* |  |  |  |  | 1.35 | 0.0499 |  |  |
| MYC binding protein | *MYCBP* |  |  |  |  | 0.928 | 0.0274 |  |  |
| myosin light chain 12B | *Myl12b* |  |  |  |  | 0.639 | 0.03 |  |  |
| MX dynamin-like GTPase 2 | *Mx2* |  |  | 2.353 | 0.00951 |  |  |  |  |

| **Supplemental Table S14F Differentially Expressed 17-β Estradiol Target Genes in Thyrotropes and 3 Lactotrope clusters**  **VCD vs Vehicle control females** | | | | | | | | | |
| --- | --- | --- | --- | --- | --- | --- | --- | --- | --- |
|  |  | **Thyrotropes** | | **LAC1** | | **LAC2** | | **LAC3** | |
|  | **Gene** | **Expr Log Ratio** | **Expr p-value** | **Expr Log Ratio** | **Expr p-value** | **Expr Log Ratio** | **Expr p-value** | **Expr Log Ratio** | **Expr p-value** |
| myosin heavy chain 10 | *Myh10* |  |  | 0.617 | 0.0097 | 0.589 | 0.0208 |  |  |
| myosin VB | *Myo5B* |  |  | 1.505 | 0.00558 |  |  |  |  |
| **NADH:ubiquinone oxidoreductase subunit B11** | ***NDUFB11*** | 1.322 | 0.054 | 0.709 | 0.00606 |  |  |  |  |
| nuclear factor of activated T cells 3 | *Nfatc3* |  |  | 1.437 | 0.00683 |  |  |  |  |
| NFE2 like bZIP transcription factor 3 | *Nfe2l3* |  |  | 1.122 | 0.0159 |  |  |  |  |
| nuclear transcription factor Y subunit alpha | *Nfya* |  |  | 1.884 | 0.0167 | 1.36 | 0.00367 | 1.453 | 0.0177 |
| noggin | *Nog* |  |  | 0.644 | 0.0112 |  |  |  |  |
| NOP56 ribonucleoprotein | *Nop56* |  |  | 0.956 | 0.000427 |  |  |  |  |
| neuronal pentraxin receptor | *Nptxr* |  |  | 0.619 | 0.0413 | 0.575 | 0.0113 | 0.483 | 0.0397 |
| nuclear receptor subfamily 4 group A member 3 | *Nr4a3* |  |  | 1.454 | 2.89E-11 | 1.385 | 6.76E-14 | 1.018 | 0.00259 |
| neurotrophic receptor tyrosine kinase 3 | *Ntrk3* |  |  | -1.986 | 0.051 | 1.133 | 0.00000209 | 1.541 | 0.000124 |
| nudix hydrolase 1 | *Nudt1* |  |  | 1.669 | 0.0382 |  |  |  |  |
| nucleoporin 37 | *Nup37* |  |  | 2.505 | 0.00338 |  |  |  |  |
| nucleoredoxin | *Nxn* |  |  | 0.709 | 0.00606 | 2.797 | 0.00338 |  |  |
| NADH:ubiquinone oxidoreductase subunit A3 | *NDUFA3* |  |  |  |  |  |  | 0.649 | 0.0469 |
| NFKB inhibitor alpha | *NFKBIA* |  |  |  |  |  |  | 0.644 | 0.00537 |
| nuclear receptor subfamily 3 group C member 2 | *NR3C2* |  |  |  |  |  |  | 0.789 | 0.0161 |
| nudix hydrolase 7 | *NUDT7* |  |  |  |  |  |  | -1.843 | 0.0511 |
| NADH:ubiquinone oxidoreductase complex assembly factor 4 | *NDUFAF4* |  |  |  |  | 0.967 | 0.00334 |  |  |
| nuclear receptor subfamily 4 group A member 2 | *NR4A2* |  |  |  |  | 0.592 | 1.32E-08 |  |  |
| neuropilin 1 | *NRP1* |  |  |  |  | 0.838 | 0.0235 |  |  |
| nudix hydrolase 9 | *NUDT9* |  |  |  |  | 0.961 | 0.0417 |  |  |
| olfactomedin 4 | *OLFM4* |  |  |  |  |  |  | -0.694 | 0.0457 |
| OTU deubiquitinase, ubiquitin aldehyde binding 2 | *OTUB2* |  |  |  |  |  |  | 1.912 | 0.0323 |
| ornithine decarboxylase 1 | *Odc1* |  |  | 0.719 | 0.018 |  |  | 0.81 | 0.00334 |
| ornithine aminotransferase | *Oat* |  |  |  |  |  |  |  |  |
| opsin 3 | *Opn3* |  |  | 0.954 | 0.0139 |  |  |  |  |
| opioid receptor mu 1 | *Oprm1* |  |  | -1.369 | 0.00252 |  |  |  |  |
| oxoglutarate dehydrogenase | *OGDH* | 1.415 | 0.0218 |  |  |  |  |  |  |
| oxysterol binding protein like 5 | *OSBPL5* |  |  |  |  | 2.06 | 0.039 |  |  |
| oxysterol binding protein like 7 | *OSBPL7* |  |  |  |  | 1.253 | 0.0543 |  |  |
| prolyl 4-hydroxylase subunit alpha 1 | *P4HA1* |  |  | 0.756 | 0.000234 |  |  | 0.687 | 0.00492 |
| PDGFA associated protein 1 | *PDAP1* |  |  |  |  | 0.713 | 0.00101 | 0.655 | 0.0106 |
| platelet derived growth factor C | *PDGFC* |  |  |  |  |  |  | 2.064 | 0.0487 |
| prohibitin 2 | *PHB2* |  |  |  |  |  |  | 0.678 | 0.0514 |
| phosphoinositide-3-kinase regulatory subunit 2 | *PIK3R2* |  |  |  |  |  |  | 1.19 | 0.0171 |
| phosphatidylinositol-4-phosphate 5-kinase type 1 beta | *PIP5K1B* |  |  |  |  |  |  | 0.723 | 0.0151 |
| phospholipase A2 activating protein | *PLAA* |  |  |  |  | 0.691 | 0.0436 | 1.064 | 0.0228 |
| DNA polymerase alpha 1, catalytic subunit | *POLA1* |  |  | 0.809 | 0.00791 |  |  | -0.942 | 0.035 |
| periplakin | *PPL* |  |  |  |  |  |  | 2.064 | 0.0487 |
| patched 1 | *PTCH1* |  |  |  |  | 0.738 | 0.0442 |  |  |
| phosphoglycerate kinase 1 | *PGK1* | 2.322 | 0.0417 |  |  |  |  |  |  |
| PHD finger protein 21B | *PHF21B* | 3.322 | 0.00538 |  |  |  |  |  |  |
| paraoxonase 2 | *PON2* | 2.807 | 0.0416 |  |  |  |  |  |  |
| pyruvate carboxylase | *Pcx* |  |  | -1.219 | 0.0197 |  |  |  |  |
| phosphoenolpyruvate carboxykinase 2, mitochondrial | *Pck2* |  |  | 2.353 | 0.0387 |  |  |  |  |
| proliferating cell nuclear antigen | *Pcna* |  |  | 1.473 | 0.00828 |  |  |  |  |
| pyruvate dehydrogenase kinase 2 | *Pdk2* |  |  | -3.986 | 0.000417 |  |  |  |  |
| phosphoinositide-3-kinase regulatory subunit 3 | *Pik3r3* |  |  | 0.621 | 0.00116 |  |  |  |  |
| Pim-2 proto-oncogene, serine/threonine kinase | *Pim2* |  |  | 1.11 | 0.0348 |  |  |  |  |
| protein phosphatase, Mg2+/Mn2+ dependent 1D | *Ppm1d* |  |  | 1.032 | 0.00522 |  |  |  |  |

| **Supplemental Table S14G Differentially Expressed 17-β Estradiol Target Genes in Thyrotropes and 3 Lactotrope clusters**  **VCD vs Vehicle control females** | | | | | | | | | |
| --- | --- | --- | --- | --- | --- | --- | --- | --- | --- |
|  |  | **Thyrotropes** | | **LAC1** | | **LAC2** | | **LAC3** | |
|  | Gene | Expr Log Ratio | Expr p-value | Expr Log Ratio | Expr p-value | Expr Log Ratio | Expr p-value | Expr Log Ratio | Expr p-value |
| protein tyrosine phosphatase 4A2 | *Ptp4a2* |  |  | 0.625 | 0.0226 | 0.463 | 0.0131 |  |  |
| **protein tyrosine phosphatase non-receptor type 11** | ***Ptpn11*** | 2.807 | 0.0212 | 0.74 | 0.00326 |  |  |  |  |
| protein tyrosine phosphatase non-receptor type 12 | *Ptpn12* |  |  | 1.032 | 0.0311 |  |  |  |  |
| protein tyrosine phosphatase receptor type N | *Ptprn* |  |  | 0.635 | 0.0000353 | 0.729 | 0.000000123 |  |  |
| protein tyrosine phosphatase receptor type S | *Ptprs* |  |  | 0.705 | 4.85E-09 | 0.508 | 0.0000808 |  |  |
| pseudouridine synthase 7 | *Pus7* |  |  | 0.666 | 0.0266 |  |  |  |  |
| protein disulfide isomerase family A member 3 | *PDIA3* | 1.433 | 0.016 |  |  |  |  |  |  |
| protein kinase C beta | *PRKCB* | -1.766 | 0.0431 |  |  |  |  |  |  |
| parkin coregulated | *PACRG* |  |  |  |  | 0.685 | 0.000492 |  |  |
| p21 (RAC1) activated kinase 1 | *PAK1* |  |  |  |  | 0.703 | 0.0101 |  |  |
| peptidylglycine alpha-amidating monooxygenase | *PAM* |  |  |  |  | 0.818 | 1.9E-13 |  |  |
| -- | *PANK1* |  |  |  |  | 2.06 | 0.039 |  |  |
| par-3 family cell polarity regulator | *PARD3* |  |  |  |  | 0.643 | 0.000499 |  |  |
| Purkinje cell protein 4 | *PCP4* |  |  |  |  | 2.163 | 0.00000364 |  |  |
| prenylcysteine oxidase 1 | *PCYOX1* |  |  |  |  | 1.06 | 0.00744 |  |  |
| PDGFA associated protein 1 | *PDAP1* |  |  |  |  | 0.713 | 0.00101 |  |  |
| PDZK1 interacting protein 1 | *PDZK1IP1* |  |  |  |  | 1.186 | 0.0245 |  |  |
| phosphoinositide-3-kinase regulatory subunit 1 | *PIK3R1* |  |  |  |  | 0.668 | 0.0149 |  |  |
| phosphatidylinositol-5-phosphate 4-kinase type 2 alpha | *PIP4K2A* |  |  |  |  | -0.755 | 0.0501 |  |  |
| PLAG1 like zinc finger 1 | *PLAGL1* |  |  |  |  | 0.808 | 7.37E-17 |  |  |
| purine nucleoside phosphorylase | *PNP* |  |  |  |  | -1.451 | 0.0326 |  |  |
| protein phosphatase 2 regulatory subunit B gamma | *PPP2R2C* |  |  | 0.914 | 0.00134 | 0.623 | 0.0505 |  |  |
| protein phosphatase 5 catalytic subunit | *PPP5C* |  |  |  |  | 1.02 | 0.0115 |  |  |
| phosphoserine aminotransferase 1 | *PSAT1* |  |  |  |  | 0.952 | 0.012 |  |  |
| protein tyrosine phosphatase 4A2 | *Ptp4a2* |  |  | 0.625 | 0.0226 | 0.463 | 0.0131 |  |  |
| protein disulfide isomerase family A member 3 | *PDIA3* | 1.433 | 0.016 |  |  |  |  |  |  |
| parkin coregulated | *PACRG* |  |  |  |  | 0.685 | 0.000492 |  |  |
| p21 (RAC1) activated kinase 1 | *PAK1* |  |  |  |  | 0.703 | 0.0101 |  |  |
| peptidylglycine alpha-amidating monooxygenase | *PAM* |  |  |  |  | 0.818 | 1.9E-13 |  |  |
| par-3 family cell polarity regulator | *PARD3* |  |  |  |  | 0.643 | 0.000499 |  |  |
| Purkinje cell protein 4 | *PCP4* |  |  |  |  | 2.163 | 0.00000364 |  |  |
| prenylcysteine oxidase 1 | *PCYOX1* |  |  |  |  | 1.06 | 0.00744 |  |  |
| PDZK1 interacting protein 1 | *PDZK1IP1* |  |  |  |  | 1.186 | 0.0245 |  |  |
| phosphoinositide-3-kinase regulatory subunit 1 | *PIK3R1* |  |  |  |  | 0.668 | 0.0149 |  |  |
| phosphatidylinositol-5-phosphate 4-kinase type 2 alpha | *PIP4K2A* |  |  |  |  | -0.755 | 0.0501 |  |  |
| PLAG1 like zinc finger 1 | *PLAGL1* |  |  |  |  | 0.808 | 7.37E-17 |  |  |
| polo like kinase 2 | *PLK2* |  |  |  |  | 1.35 | 0.0154 |  |  |
| protein phosphatase 5 catalytic subunit | *PPP5C* |  |  |  |  | 1.02 | 0.0115 |  |  |
| prickle planar cell polarity protein 2 | *PRICKLE2* |  |  |  |  | 0.621 | 0.028 |  |  |
| phosphoserine aminotransferase 1 | *PSAT1* |  |  |  |  | 0.952 | 0.012 |  |  |
| polypyrimidine tract binding protein 2 | *PTBP2* |  |  |  |  | 0.606 | 0.00543 |  |  |
| prostaglandin-endoperoxide synthase 2 | *PTGS2* |  |  |  |  | 3.645 | 0.00829 |  |  |
| phosphodiesterase 4B | *PDE4B* | 2.807 | 0.0417 |  |  |  |  |  |  |
| phosphoglycerate kinase 1 | *PGK1* | 2.322 | 0.0417 |  |  |  |  |  |  |
| PHD finger protein 21B | *PHF21B* | 3.322 | 0.00538 |  |  |  |  |  |  |
| paraoxonase 2 | *PON2* | 2.807 | 0.0416 |  |  |  |  |  |  |
| prolyl 4-hydroxylase subunit alpha 1 | *P4ha1* |  |  | 0.756 | 0.000234 |  |  | 0.687 | 0.00492 |
| pyruvate carboxylase | *Pcx* |  |  | -1.219 | 0.0197 |  |  |  |  |
| phosphoenolpyruvate carboxykinase 2, mitochondrial | *Pck2* |  |  | 2.353 | 0.0387 |  |  |  |  |
| pyruvate dehydrogenase kinase 2 | *Pdk2* |  |  | -3.986 | 0.000417 |  |  |  |  |
| phosphoinositide-3-kinase regulatory subunit 3 | *Pik3r3* |  |  | 0.621 | 0.00116 |  |  |  |  |

| **Supplemental Table S14H Differentially Expressed 17-β Estradiol Target Genes in Thyrotropes and 3 Lactotrope clusters**  **VCD vs Vehicle control females** | | | | | | | | | |
| --- | --- | --- | --- | --- | --- | --- | --- | --- | --- |
|  |  | **Thyrotropes** | | **LAC1** | | **LAC2** | | **LAC3** | |
|  | **Gene** | **Expr Log Ratio** | **Expr p-value** | **Expr Log Ratio** | **Expr p-value** | **Expr Log Ratio** | **Expr p-value** | **Expr Log Ratio** | **Expr p-value** |
| Pim-2 proto-oncogene, serine/threonine kinase | *Pim2* |  |  | 1.11 | 0.0348 |  |  |  |  |
| DNA polymerase alpha 1, catalytic subunit | *Pola1* |  |  | 0.809 | 0.00791 |  |  | -0.942 | 0.035 |
| protein phosphatase, Mg2+/Mn2+ dependent 1D | *Ppm1d* |  |  | 1.032 | 0.00522 |  |  |  |  |
| PPARG related coactivator 1 | *Pprc1* |  |  | 1.184 | 0.00259 |  |  |  |  |
| Prolactin | *Prl* | -1.256 | 0.0222 |  |  |  |  |  |  |
| prosaposin | *BTG1* | 2.115 | 0.00395 |  |  |  |  |  |  |
| RAN, member RAS oncogene family |  | 3 | 0.0107 |  |  |  |  |  |  |
| ras related dexamethasone induced 1 | *RASD1* |  |  |  |  |  |  | 0.643 | 0.0353 |
| REL proto-oncogene, NF-kB subunit | *REL* |  |  |  |  | 1.668 | 0.00999 | 1.29 | 0.032 |
| ring finger protein 4 | *RNF4* |  |  |  |  |  |  | 0.774 | 0.0243 |
| ribophorin I | *RPN1* |  |  |  |  |  |  | 0.702 | 0.0284 |
| RUNX family transcription factor 1 | *RUNX1* |  |  |  |  |  |  | 2.965 | 0.000564 |
| regulator of calcineurin 1 | *RAN* | 3 | 0.0213 |  |  |  |  |  |  |
| RAB31, member RAS oncogene family | *Rab31* |  |  | 1.245 | 0.0141 |  |  |  |  |
| retinoic acid receptor alpha | *Rara* |  |  | -1.179 | 0.0489 |  |  |  |  |
| retinol binding protein 1 | *Rbp1* |  |  | 1.92 | 0.021 |  |  |  |  |
| retinol dehydrogenase 10 | *Rdh10* |  |  | 1.09 | 0.0186 |  |  |  |  |
| RAS like estrogen regulated growth inhibitor | *Rerg* |  |  | 0.658 | 0.0402 | 0.593 | 0.0221 | 0.742 | 0.0318 |
| replication factor C subunit 1 | *Rfc1* |  |  | 0.611 | 0.0264 |  |  |  |  |
| Rho family GTPase 3 | *Rnd3* |  |  | 0.768 | 0.00222 | 0.325 | 0.0326 | 0.965 | 0.00576 |
| ring finger protein 128 | *Rnf128* |  |  | 0.681 | 0.0241 | 0.556 | 0.0407 |  |  |
| ribosome binding protein 1 | *Rrbp1* |  |  | 0.724 | 0.00000164 | 0.403 | 0.00717 |  |  |
| reticulon 2 | *Rtn2* |  |  | 0.959 | 0.0169 |  |  |  |  |
| RAB5C, member RAS oncogene family | *RAB5C* |  |  |  |  | 1.298 | 0.00995 |  |  |
| RB transcriptional corepressor 1 | *RB1* |  |  |  |  | 0.954 | 0.00328 |  |  |
| RB transcriptional corepressor like 2 | *RBL2* |  |  |  |  | 0.949 | 0.0275 |  |  |
| REST corepressor 1 | *RCOR1* |  |  |  |  | 0.851 | 0.0131 |  |  |
| regulator of G protein signaling 7 | *RGS7* |  |  |  |  | 0.617 | 0.00237 |  |  |
| ras homolog family member Q | *RHOQ* |  |  |  |  | 0.698 | 0.0161 |  |  |
| ring finger protein, LIM domain interacting | *RLIM* |  |  |  |  | 0.599 | 0.0401 |  |  |
| **semaphorin 3A** | ***SEMA3A*** | -3 | 0.0213 |  |  |  |  | -0.704 | 0.000664 |
| solute carrier family 25 member 12 | *SLC25A12* | 2 | 0.0271 |  |  |  |  |  |  |
| solute carrier family 37 member 1 | *SLC37A1* |  |  |  |  |  |  | 1.37 | 0.0332 |
| SMAD family member 2 | *SMAD2* | -2.17 | 0.0441 | 0.991 | 0.0385 |  |  |  |  |
| SMAD family member 3 | *SMAD3* |  |  |  |  |  |  | 1.183 | 0.0285 |
| signal sequence receptor subunit 3 | *SSR3* | 1.807 | 0.0475 |  |  |  |  |  |  |
| signal sequence receptor subunit 1 | *SSR1* |  |  |  |  |  |  | 0.623 | 0.00417 |
| ST6 beta-galactoside alpha-2,6-sialyltransferase 1 | *ST6GAL1* | 3 | 0.0417 |  |  |  |  |  |  |
| serine/threonine kinase 39 |  | 1.874 | 0.0372 |  |  |  |  |  |  |
| Scm polycomb group protein like 4 | *Scml4* |  |  | -0.986 | 0.0107 |  |  |  |  |
| syndecan 1 | *SDC1* |  |  |  |  |  |  | 2.327 | 0.0198 |
| syndecan 2 | *Sdc2* |  |  | 0.801 | 0.00000908 |  |  | 1.327 | 0.0349 |
| SAM and HD domain containing deoxynucleoside triphosphate triphosphohydrolase 1 | *SAMHD1* |  |  |  |  |  |  |  |  |
| SUMO specific peptidase 5 | *Senp5* |  |  | 0.676 | 0.00915 | 0.523 | 0.0324 |  |  |
| SIN3 transcription regulator family member B | *Sin3b* |  |  | 0.614 | 0.049 | 0.833 | 0.0107 |  |  |

| **Supplemental Table S14I Differentially Expressed 17-β Estradiol Target Genes in Thyrotropes and 3 Lactotrope clusters**  **VCD vs Vehicle control females** | | | | | | | | | |
| --- | --- | --- | --- | --- | --- | --- | --- | --- | --- |
|  |  | **Thyrotropes** | | **LAC1** | | **LAC2** | | **LAC3** | |
|  | **Gene** | **Expr Log Ratio** | **Expr p-value** | **Expr Log Ratio** | **Expr p-value** | **Expr Log Ratio** | **Expr p-value** | **Expr Log Ratio** | **Expr p-value** |
| solute carrier family 2 member 3 | *Slc2A3* |  |  | 1.991 | 0.0388 |  |  |  |  |
| solute carrier family 38-member 2 | *Slc38a2* |  |  | 0.622 | 0.000396 |  |  |  |  |
| solute carrier family 6-member 17 | *Slc6A17* |  |  | 0.583 | 0.000765 | 0.867 | 4.76E-6 |  |  |
| sphingomyelin phosphodiesterase acid like 3A | *SmpDLl3A* |  |  | -1.401 | 0.0238 |  |  | -1.316 | 0.0516 |
| sorbitol dehydrogenase | *Sord* |  |  | -2.517 | 0.0509 |  |  |  |  |
| suppressor of cytokine signaling 1 | *SOCS1* |  |  |  |  |  |  | 1.042 | 0.052 |
| SRC proto-oncogene, non-receptor tyrosine kinase | *Src* |  |  |  |  |  |  | 1.38 | 0.0194 |
| SRY-box transcription factor 5 | *Sox5* |  |  | 0.715 | 0.0273 |  |  |  |  |
| Sp1 transcription factor | *Sp1* |  |  | 0.768 | 0.0217 | 0.838 | 0.017 |  |  |
| squalene epoxidase | *Sqle* |  |  | 1.562 | 0.0384 |  |  |  |  |
| serine and arginine rich splicing factor 2 | *Srsf2* |  |  | 0.607 | 0.00316 |  |  |  |  |
| ST8 alpha-N-acetyl-neuraminide alpha-2,8-sialyltransferase 1 | *St8sia1* |  |  | -2.138 | 0.051 |  |  |  |  |
| secretogranin II | *SCG2* |  |  |  |  | 0.588 | 0.00044 |  |  |
| serum/glucocorticoid regulated kinase 1 | *SGK1* |  |  |  |  | 0.797 | 0.0382 |  |  |
| siah E3 ubiquitin protein ligase 2 | *SIAH2* |  |  |  |  | 0.677 | 0.00223 |  |  |
| SIN3 transcription regulator family member B | *SIN3B* |  |  |  |  | 0.833 | 0.0107 |  |  |
| solute carrier family 12 member 2 | *SLC12A2* |  |  |  |  | 0.85 | 0.0507 |  |  |
| solute carrier family 19 member 2 | *SLC19A2* |  |  |  |  | 2.212 | 0.00695 |  |  |
| solute carrier family 2 member 13 | *SLC2A13* |  |  |  |  | 0.933 | 2E-21 |  |  |
| solute carrier family 31 member 1 | *SLC31A1* |  |  |  |  | 0.86 | 0.00337 |  |  |
| solute carrier family 38 member 9 | *SLC38A9* |  |  |  |  | -0.917 | 0.0208 |  |  |
| solute carrier family 6 member 8 | *SLC6A8* |  |  |  |  | 1.283 | 0.0343 |  |  |
| SOS Ras/Rac guanine nucleotide exchange factor 1 | *SOS1* |  |  |  |  | 0.606 | 0.0306 |  |  |
| sperm associated antigen 9 | *SPAG9* |  |  |  |  | 0.712 | 5.21E-7 |  |  |
| sprouty RTK signaling antagonist 2 | *SPRY2* |  |  |  |  | 2.475 | 0.0183 |  |  |
| signal transducer and activator of transcription 3 | *STAT3* |  |  |  |  |  |  | 1.03 | 8.5E-5 |
| stress induced phosphoprotein 1 | *STIP1* |  |  |  |  |  |  | 1.019 | 6E-5 |
| syntaxin 3 | *STX3* |  |  |  |  |  |  | 0.771 | 0.0167 |
| sterol regulatory element binding transcription factor 1 | *SREBF1* |  |  |  |  | 0.645 | 0.0335 |  |  |
| sterol regulatory element binding transcription factor 2 | *SREBF2* |  |  |  |  | 0.982 | 0.027 |  |  |
| serine and arginine rich splicing factor 7 | *SRSF7* |  |  |  |  | 0.741 | 0.00017 |  |  |
| transmembrane protein 245 | *TMEM245* | -2.585 | 0.0416 |  |  |  |  |  |  |
| transcription factor 7 | *Tcf7* |  |  | 3.184 | 0.00892 |  |  |  |  |
| thyroid hormone receptor alpha | *Thra* |  |  | 0.724 | 0.0169 |  |  |  |  |
| thyroid hormone receptor beta | *Thrb* |  |  | 0.826 | 5.51E-08 | 0.571 | 0.00085 |  |  |
| translocase of inner mitochondrial membrane 8 homolog B | *Timm8b* |  |  | 1.09 | 0.03 | 0.627 | 0.0126 |  |  |
| tight junction associated protein 1 | *Tjap1* |  |  | 1.02 | 0.00803 |  |  |  |  |
| transmembrane protein 164 | *Tmem164* |  |  | 1.14 | 0.019 |  |  |  |  |
| transmembrane protein 258 | *Tmem258* |  |  | 0.753 | 0.00587 | 0.859 | 0.00113 |  |  |
| transmembrane inner ear | *Tmie* |  |  | 0.977 | 0.0533 |  |  |  |  |
| TNF alpha induced protein 3 | *Tnfaip3* |  |  | 2.032 | 0.0166 | 1.797 | 0.0369 |  |  |
| tropomyosin 3 | *Tpm3* |  |  | 0.799 | 0.0105 |  |  |  |  |
| TNF receptor associated factor 3 |  |  |  | 0.822 | 0.00462 |  |  |  |  |
| tripartite motif containing 25 | *Trim25* |  |  | 1.698 | 0.000946 |  |  |  |  |
| tyrosine kinase 2 | *Tyk2* |  |  | 0.841 | 0.0295 |  |  |  |  |
| tumor protein p53 | *TP53* | 2.322 | 0.0417 |  |  |  |  |  |  |
| translocated promoter region, nuclear basket protein | *TPR* | 1.459 | 0.045 |  |  |  |  |  |  |
| **transformer 2 beta homolog** | ***Tra2b*** | 1.585 | 0.0317 |  |  | 0.859 | 0.00113 |  |  |
| transmembrane protein 120B | *TMEM120B* |  |  |  |  | 0.808 | 0.0492 |  |  |
| TAR DNA binding protein | *Tardbp* |  |  |  |  | 1.122 | 0.00136 |  |  |
| TATA element modulatory factor 1 | *TMF1* |  |  |  |  | 0.87 | 0.0138 |  |  |
| thymopoietin | *TMPO* |  |  |  |  | 0.606 | 0.0125 |  |  |
| TNF alpha induced protein 3 | *TNFAIP3* |  |  | 2.032 | 0.0166 | 1.797 | 0.0369 |  |  |
| tensin 3 | *TNS3* |  |  |  |  | 1.06 | 0.0178 | 0.793 | 0.0321 |
| testis associated actin remodelling kinase 1 | *TESK1* |  |  |  |  | 0.75 | 0.00442 |  |  |
| TIAM Rac1 associated GEF 1 | *TIAM1* |  |  | 1.09 | 0.03 | 0.627 | 0.0126 |  |  |

| **Supplemental Table S14J Differentially Expressed 17-β Estradiol Target Genes in Thyrotropes and 3 Lactotrope clusters**  **VCD vs Vehicle control females** | | | | | | | | | |
| --- | --- | --- | --- | --- | --- | --- | --- | --- | --- |
|  |  | **Thyrotropes** | | **LAC1** | | **LAC2** | | **LAC3** | |
|  | **Gene** | **Expr Log Ratio** | **Expr p-value** | **Expr Log Ratio** | **Expr p-value** | **Expr Log Ratio** | **Expr p-value** | **Expr Log Ratio** | **Expr p-value** |
| translocase of inner mitochondrial membrane 8 homolog B | *TIMM8B* |  |  |  |  | 0.89 | 0.0412 |  |  |
| tight junction protein 1 | *TJP1* |  |  |  |  | 0.82 | 0.0000115 |  |  |
| thymosin beta 4 X-linked | *TMSB4X* |  |  |  |  |  |  | 1.203 | 0.000243 |
| tumor protein p53 inducible protein 11 | *TP53I11* |  |  |  |  |  |  | 0.988 | 0.00202 |
| tribbles pseudokinase 1 | *TRIB1* |  |  |  |  |  |  | 0.855 | 0.0205 |
| tripartite motif containing 24 | *TRIM24* |  |  |  |  |  |  | -1.293 | 0.0131 |
| tropomyosin 1 | *TPM1* |  |  |  |  | 0.761 | 0.0299 | 0.793 | 0.0321 |
| transient receptor potential cation channel subfamily C member 1 | *TRPC1* |  |  |  |  | 0.598 | 0.0233 | 0.868 | 0.0304 |
| TSC complex subunit 1 | *TSC1* |  |  |  |  | 0.775 | 0.00933 |  |  |
| tubulin alpha 1b | *TUBA1B* |  |  |  |  | 1.011 | 0.00201 |  |  |
| tubulin beta 4B class IVb | *TUBB4B* |  |  | 1.146 | 0.00211 | 0.673 | 0.000251 |  |  |
| thioredoxin like 4A | *TXNL4A* |  |  |  |  |  |  |  |  |
| Thyroid hormone receptor interactor 11 | *TRIP11* |  |  |  |  |  |  | 0.584 | 0.0185 |
| TSC22 domain family member 2 | *TSC22D2* |  |  |  |  |  |  | 1.131 | 0.00128 |
| TSC22 domain family member 3 | *TSC22D3* |  |  |  |  |  |  | 2.023 | 0.00113 |
| ubiquitin conjugating enzyme E2 B |  | 0.893 | 0.0207 |  |  |  |  |  |  |
| uridine-cytidine kinase 2 | *Uck2* |  |  | 0.644 | 0.00464 | 0.578 | 0.00159 |  |  |
| unc-5 netrin receptor C | *Unc5c* |  |  | 0.62 | 0.00118 | 0.763 | 0.0398 |  |  |
| ubiquitin like modifier activating enzyme 5 | *UBA5* |  |  |  |  | 1.164 | 0.000162 |  |  |
| ubiquitin specific peptidase 48 | *USP48* |  |  |  |  | 0.644 | 0.00708 |  |  |
| ubiquinol-cytochrome c reductase, complex III subunit XI | *UQCR11* |  |  |  |  | 0.694 | 0.0000016 |  |  |
| ubiquitin specific peptidase 35 | *USP35* |  |  |  |  | 1.088 | 0.0177 |  |  |
| vav guanine nucleotide exchange factor 3 | *Vav3* |  |  | 1.66 | 0.0000048 |  |  |  |  |
| versican | *VCAN* |  |  |  |  | -2.427 | 0.0403 |  |  |
| VGF nerve growth factor inducible | *VGF* |  |  |  |  | 1.923 | 0.000542 |  |  |
| WAP four-disulfide core domain 2 | *WFDC2* |  |  |  |  | 3.176 | 0.0388 |  |  |
| vascular endothelial growth factor A | *Vegf* |  |  | 0.949 | 0.00684 |  |  |  |  |
| Wnt ligand secretion mediator | *UBE2B* | 2.7 | 0.0227 |  |  |  |  |  |  |
| vitamin D receptor | *Vdr* |  |  | 2.184 | 0.0387 |  |  |  |  |
| X-box binding protein 1 |  |  |  | 0.603 | 0.0035 | 0.652 | 0.00248 |  |  |
| YTH N6-methyladenosine RNA binding protein F2 |  |  |  |  |  | 0.755 | 0.034 |  |  |
| YY1 transcription factor | *Yy1* |  |  | 0.581 | 0.00506 |  |  |  |  |
| zinc finger and BTB domain containing 10 | *Zbtb10* |  |  | 1.353 | 0.00314 |  |  |  |  |
| zinc finger and BTB domain containing 16 | *Zbtb16* |  |  | 1.321 | 0.0144 |  |  |  |  |
| zinc finger DHHC-type palmitoyltransferase 7 | *Zdhhc7* |  |  | 1.127 | 0.0104 | 1.838 | 0.00165 |  |  |
| zinc finger homeobox 2 | *Zfhx2* |  |  | 0.825 | 0.00125 | 0.71 | 0.0028 |  |  |
| zinc finger with KRAB and SCAN domains 1 | *Zkscan1* |  |  | 0.674 | 0.0282 |  |  |  |  |
| zinc finger MYM-type containing 3 | *Zmym3* |  |  | 0.663 | 0.0529 |  |  |  |  |
| zinc finger SWIM-type containing 6 | *Zswim6* |  |  | 0.878 | 0.000587 |  |  |  |  |
| zinc finger E-box binding homeobox 2 | *WLS* | 2.70 | 0.02266 |  |  |  |  |  |  |
| zinc finger SWIM-type containing 6 | *ZEB2* | 3.17 | 0.00534 |  |  |  |  |  |  |
| zinc finger and BTB domain containing 2 | *ZBTB2* |  |  |  |  | 1.563 | 0.0197 |  |  |
| zinc finger CCHC-type containing 12 | *ZCCHC12* |  |  |  |  | 0.672 | 0.0143 |  |  |
| zinc finger protein 317 | *ZNF317* |  |  |  |  | 1.35 | 0.0499 |  |  |
| zinc finger protein 516 | *ZNF516* |  |  |  |  | 0.827 | 0.000118 |  |  |
| zinc finger ZZ-type containing 3 | *ZZZ3* |  |  |  |  | 0.773 | 0.00756 |  |  |
| zinc finger matrin-type 3 | *ZMAT3* |  |  |  |  |  |  | 1.364 | 0.000113 |
| zinc finger protein 219 | *ZNF219* |  |  |  |  |  |  | -1.094 | 0.0234 |
| zinc finger protein 503 | *ZNF503* |  |  |  |  |  |  | 1.681 | 0.00149 |
| zyxin | *ZYX* |  |  |  |  |  |  | 1.617 | 0.0184 |

| **Supplemental Table S15A DEGS Corticotrope Cluster. VCD/Vehicle.** Maroon=upregulated | | | | | | | | |
| --- | --- | --- | --- | --- | --- | --- | --- | --- |
| **gene** | **avg_log2FC** | **p_val** | **gene** | **avg_log2FC** | **p_val** | **gene** | **avg_log2FC** | **p_val** |
| *Atad3a* | 3.5474878 | 0.0027334 | *Dab1* | 2.5474878 | 0.0113732 | *Gm56636* | 2.5474878 | 0.03583761 |
| *Ddx60* | 3.5474878 | 0.0027334 | *Mtfr1* | 2.5474878 | 0.01359289 | *Ndn* | 2.47348721 | 0.00067646 |
| *Farsa* | 3.5474878 | 0.00639136 | *Rusc1* | 2.5474878 | 0.01359289 | *Zfp1* | 2.35484272 | 0.00123562 |
| *Gm47204* | 3.5474878 | 0.00639136 | *Gm12160* | 2.5474878 | 0.01359289 | *Ss18l1* | 2.35484272 | 0.0137616 |
| *Chia1* | 3.35484272 | 0.00273591 | *Derl2* | 2.5474878 | 0.01359289 | *Fbxo21* | 2.35484272 | 0.0137616 |
| *Acnat1* | 3.35484272 | 0.00638959 | *5031439G07Rik* | 2.5474878 | 0.01359289 | *Trim56* | 2.35484272 | 0.0137616 |
| *Kank2* | 3.35484272 | 0.00638959 | *Rgmb* | 2.5474878 | 0.03057007 | *4930557K07Rik* | 2.35484272 | 0.0137616 |
| *Ccdc126* | 3.35484272 | 0.01503224 | *Osgepl1* | 2.5474878 | 0.03583761 | *Zfp235* | 2.35484272 | 0.0137616 |
| *Sinhcaf* | 3.35484272 | 0.01503224 | *Casp8* | 2.5474878 | 0.03583761 | *2810001G20Rik* | 2.35484272 | 0.0137616 |
| *Snapc2* | 3.35484272 | 0.01503224 | *Serpinb8* | 2.5474878 | 0.03583761 | *Nras* | 2.35484272 | 0.02026704 |
| *Ppcdc* | 3.1324503 | 0.00111621 | *Stk35os1* | 2.5474878 | 0.03583761 | *Angptl2* | 2.35484272 | 0.03090152 |
| *Epha6* | 3.1324503 | 0.00174985 | *Gm14280* | 2.5474878 | 0.03583761 | *Noct* | 2.35484272 | 0.03090152 |
| *Alg5* | 3.1324503 | 0.00639253 | *Acss1* | 2.5474878 | 0.03583761 | *Elavl3* | 2.35484272 | 0.03090152 |
| *Wdr93* | 3.1324503 | 0.00639253 | *Nfs1* | 2.5474878 | 0.03583761 | *Gm12592* | 2.35484272 | 0.03090152 |
| *Llgl2* | 3.1324503 | 0.00639253 | *Slc17a9* | 2.5474878 | 0.03583761 | *Cluh* | 2.35484272 | 0.03090152 |
| *Ggnbp1* | 3.1324503 | 0.00639253 | *Gpr149* | 2.5474878 | 0.03583761 | *Cacna1g* | 2.35484272 | 0.03090152 |
| *4833419F23Rik* | 3.1324503 | 0.00639253 | *Chrnb2* | 2.5474878 | 0.03583761 | *Tmco6* | 2.35484272 | 0.03090152 |
| *Slc25a53* | 3.1324503 | 0.00639253 | *Gm15265* | 2.5474878 | 0.03583761 | *Eps15l1* | 2.31302254 | 0.00322764 |
| *Pak4* | 3.1324503 | 0.01503015 | *Svbp* | 2.5474878 | 0.03583761 | *Sik1* | 2.31302254 | 0.00630821 |
| *Cib1* | 3.1324503 | 0.01503015 | *Snip1* | 2.5474878 | 0.03583761 | *Nup93* | 2.28445339 | 0.0012729 |
| *Galc* | 3.1324503 | 0.01503015 | *Mfsd10* | 2.5474878 | 0.03583761 | *Anxa4* | 2.28445339 | 0.00433538 |
| *Gch1* | 3.1324503 | 0.01503015 | *Zcchc4* | 2.5474878 | 0.03583761 | *Naa30* | 2.28445339 | 0.00931459 |
| *Pgap6* | 3.1324503 | 0.01503015 | *Vsig10* | 2.5474878 | 0.03583761 | *2510009E07Rik* | 2.28445339 | 0.01986688 |
| *Pygm* | 3.1324503 | 0.01503015 | *Hrk* | 2.5474878 | 0.03583761 | *Spdya* | 2.28445339 | 0.02024172 |
| *Tekt2* | 3.1324503 | 0.03583591 | *Gm44129* | 2.5474878 | 0.03583761 | *Mt3* | 2.24792751 | 0.00446579 |
| *Nfya* | 3.1324503 | 0.03583591 | *Gm19265* | 2.5474878 | 0.03583761 | *Cfap418* | 2.1324503 | 0.00280027 |
| *Spout1* | 2.86941589 | 0.01503294 | *Prkcg* | 2.5474878 | 0.03583761 | *Lrrc75a* | 2.1324503 | 0.00646557 |
| *Dolpp1* | 2.86941589 | 0.01503294 | *Zfp446* | 2.5474878 | 0.03583761 | *Uchl5* | 2.1324503 | 0.00952738 |
| *A430018G15Rik* | 2.86941589 | 0.01503294 | *Fam187b* | 2.5474878 | 0.03583761 | *Ddx47* | 2.1324503 | 0.00952738 |
| *Mavs* | 2.86941589 | 0.01503294 | *Fes* | 2.5474878 | 0.03583761 | *Edrf1* | 2.1324503 | 0.00952738 |
| *Taf6* | 2.86941589 | 0.01503294 | *Folr1* | 2.5474878 | 0.03583761 | *Surf2* | 2.1324503 | 0.01198483 |
| *Lrrc23* | 2.86941589 | 0.01503294 | *Lcmt1* | 2.5474878 | 0.03583761 | *mt-Atp8* | 2.1324503 | 0.01198483 |
| Zfp420 | 2.86941589 | 0.01503294 | *Sap30* | 2.5474878 | 0.03583761 | *Gm973* | 2.1324503 | 0.01439557 |
| Ccdc84 | 2.86941589 | 0.01503294 | *Gm16853* | 2.5474878 | 0.03583761 | *Elk3* | 2.1324503 | 0.01439557 |
| Oxsm | 2.86941589 | 0.01503294 | *Gm40528* | 2.5474878 | 0.03583761 | *Rexo4* | 2.1324503 | 0.02024172 |
| C2cd2 | 2.86941589 | 0.01503294 | *Ccdc71* | 2.5474878 | 0.03583761 | *Flrt3* | 2.1324503 | 0.02024172 |
| Med20 | 2.86941589 | 0.01503294 | *Ggt5* | 2.5474878 | 0.03583761 | *Sike1* | 2.1324503 | 0.02024172 |
| Esrra | 2.86941589 | 0.01503294 | *Fzr1* | 2.5474878 | 0.03583761 | *Mroh7* | 2.1324503 | 0.02024172 |
| 1700018L02Rik | 2.86941589 | 0.01503294 | *Ccdc117* | 2.5474878 | 0.03583761 | *Mrps12* | 2.1324503 | 0.02024172 |
| Ntng1 | 2.86941589 | 0.01949509 | *Gm57397* | 2.5474878 | 0.03583761 | *Xndc1* | 2.1324503 | 0.02024172 |
| Nrip3 | 2.86941589 | 0.03023786 | *Sez6* | 2.5474878 | 0.03583761 | *Gm28791* | 2.1324503 | 0.03125973 |
| Polr1b | 2.86941589 | 0.03583591 | *B230217C12Rik* | 2.5474878 | 0.03583761 | *Ncbp1* | 2.1324503 | 0.03125973 |
| Plrg1 | 2.86941589 | 0.03583591 | *Mxra7* | 2.5474878 | 0.03583761 | *Plekhn1* | 2.1324503 | 0.03125973 |
| Tlr4 | 2.86941589 | 0.03583591 | *Gm48512* | 2.5474878 | 0.03583761 | *Srrd* | 2.1324503 | 0.03125973 |
| Slc46a3 | 2.86941589 | 0.03583591 | *Gm30025* | 2.5474878 | 0.03583761 | *Kri1* | 2.1324503 | 0.03125973 |
| Rassf4 | 2.86941589 | 0.03583591 | *Gm56546* | 2.5474878 | 0.03583761 | *Rmnd5b* | 2.1324503 | 0.03125973 |
| Ecsit | 2.86941589 | 0.03583591 | *Nop16* | 2.5474878 | 0.03583761 | *Septin8* | 2.1324503 | 0.03125973 |
| Wdr72 | 2.86941589 | 0.03583591 | *3830408C21Rik* | 2.5474878 | 0.03583761 | *G6pdx* | 2.1324503 | 0.03125973 |
| Coro6 | 2.86941589 | 0.03583591 | *Naga* | 2.5474878 | 0.03583761 | *Pwp1* | 2.1324503 | 0.04255341 |
| Bcas3os1 | 2.86941589 | 0.03583591 | *Zfp263* | 2.5474878 | 0.03583761 | *Gan* | 2.1324503 | 0.04332343 |
| *Elf4* | 2.86941589 | 0.03583591 | *Cfap91* | 2.5474878 | 0.03583761 | *Arid5a* | 2.06206097 | 0.05306047 |
| *Nmi* | 2.7174128 | 0.00595724 | *Msh5* | 2.5474878 | 0.03583761 | *Bub3* | 2.00691941 | 0.01229839 |
| *Nedd9* | 2.7174128 | 0.03023863 | *Gm16283* | 2.5474878 | 0.03583761 | *Daglb* | 2.00691941 | 0.05066487 |
| *Lsm1* | 2.66296501 | 0.00084257 | *Cd74* | 2.5474878 | 0.03583761 | *Bbs5* | 1.96252529 | 0.0206677 |
| *Zfp397* | 2.66296501 | 0.00414778 | *Mppe1* | 2.5474878 | 0.03583761 | *Mrpl22* | 1.96252529 | 0.0206677 |
| *Atp11a* | 2.5474878 | 0.00550829 | *Sorcs3* | 2.5474878 | 0.03583761 | *Prpsap1* | 1.96252529 | 0.0206677 |
| *Acbd7* | 2.5474878 | 0.00603705 | *Qrfpr* | 2.5474878 | 0.03583761 | *Neu1* | 1.96252529 | 0.0206677 |
| *Fez2* | 2.5474878 | 0.00603705 | *Rnf207* | 2.5474878 | 0.03583761 | *Zfp474* | 1.96252529 | 0.0206677 |
| Syt7 | 2.5474878 | 0.00603705 | *Mnx1* | 2.5474878 | 0.03583761 | *Tbc1d13* | 1.96252529 | 0.04332343 |

| **Supplemental Table S15B DEGS Corticotrope Cluster. VCD/Vehicle.** Maroon=upregulated | | | | | | | | |
| --- | --- | --- | --- | --- | --- | --- | --- | --- |
| **gene** | **avg_log2FC** | **p_val** | **gene** | **avg_log2FC** | **p_val** | **gene** | **avg_log2FC** | **p_val** |
| *Sez6l* | 1.96252529 | 0.04332343 | *Man2a2* | 1.66296501 | 0.00875459 | *Snrnp48* | 1.31302254 | 0.01981841 |
| *Eef1akmt2* | 1.96252529 | 0.04332343 | *Bag2* | 1.66296501 | 0.01548051 | *Scml4* | 1.3064797 | 0.02779809 |
| *Septin9* | 1.96252529 | 0.04332343 | *Anapc13* | 1.66296501 | 0.01548051 | *Ergic2* | 1.3064797 | 0.03113597 |
| *Tmem181a* | 1.96252529 | 0.04332343 | *Rimkla* | 1.66296501 | 0.0296919 | *Imp3* | 1.3023753 | 0.00768198 |
| *Reep2* | 1.96252529 | 0.04332343 | *Dcun1d4* | 1.64702347 | 0.00883935 | *Zbtb16* | 1.29750954 | 0.00455509 |
| *Gm50232* | 1.96252529 | 0.04332343 | *Nsrp1* | 1.64702347 | 0.00883935 | *Srxn1* | 1.28445339 | 0.00249357 |
| *Fundc1* | 1.96252529 | 0.04332343 | *Map3k7* | 1.64702347 | 0.0310581 | *Hspe1* | 1.28445339 | 0.0120498 |
| *Tjp2* | 1.92599942 | 0.0012503 | *Cops3* | 1.64702347 | 0.0310581 | *Sort1* | 1.28445339 | 0.02536722 |
| *Plec* | 1.92599942 | 0.00720177 | *Cdk9* | 1.63495064 | 0.00932355 | *U2af1* | 1.28445339 | 0.03443711 |
| *Serp1* | 1.92599942 | 0.01266016 | *Pifo* | 1.63495064 | 0.00932355 | *Fbxl5* | 1.25798118 | 0.019864 |
| *Fibp* | 1.92599942 | 0.01426102 | *Epb41l4b* | 1.63495064 | 0.01715584 | *Arhgef2* | 1.24792751 | 0.02021653 |
| *Cse1l* | 1.92599942 | 0.02768462 | *Ube2f* | 1.61787712 | 0.00149551 | *Syt11* | 1.24792751 | 0.02021653 |
| *Ube2v2* | 1.86941589 | 0.00226293 | *Qser1* | 1.5474878 | 0.00519527 | *Clcn5* | 1.2255597 | 0.01472014 |
| *Pwwp3b* | 1.86941589 | 0.01009548 | *Prpf8* | 1.5474878 | 0.00918052 | *Ube2d2a* | 1.2255597 | 0.01590265 |
| *Prkra* | 1.86941589 | 0.01267815 | *Rnf138* | 1.5474878 | 0.01778327 | *Clic1* | 1.2255597 | 0.02082052 |
| *Rad17* | 1.86941589 | 0.01535578 | *Hyou1* | 1.5474878 | 0.0220597 | *Pla2g12a* | 1.2255597 | 0.03558906 |
| *Cdc27* | 1.86941589 | 0.01594835 | *Gpr19* | 1.5474878 | 0.02733725 | *Ankle2* | 1.2255597 | 0.03558906 |
| *Leng9* | 1.86941589 | 0.02553571 | *Slc24a4* | 1.5474878 | 0.02783444 | *Acvr1* | 1.21045281 | 0.02215709 |
| *Snrpa1* | 1.86941589 | 0.02553571 | *Cycs* | 1.5474878 | 0.03042708 | *Ppp1cb* | 1.20645088 | 0.00803507 |
| *Rbms2* | 1.86941589 | 0.02553571 | *Atf1* | 1.5474878 | 0.03201635 | *Sec63* | 1.19956449 | 0.01176469 |
| *Abhd6* | 1.86941589 | 0.03013308 | *Adar* | 1.5474878 | 0.03230563 | *Atp5d* | 1.18903382 | 0.00491294 |
| *Zfp9* | 1.86941589 | 0.05066487 | *Srm* | 1.5474878 | 0.03300542 | *Lamc2* | 1.17975601 | 0.01210299 |
| *Prmt7* | 1.86941589 | 0.05066487 | *Myo1h* | 1.5474878 | 0.03300542 | *Adam9* | 1.16897617 | 0.01220588 |
| *Dennd5a* | 1.83699441 | 0.00847602 | *Fosl1* | 1.5474878 | 0.04576956 | *Psmb4* | 1.16897617 | 0.05000729 |
| *Mapk14* | 1.82050629 | 0.00247346 | *Mlf1* | 1.5474878 | 0.05320723 | *Synpo* | 1.16415916 | 0.01760914 |
| *Prpf6* | 1.8105222 | 0.00410361 | *Snrnp40* | 1.5474878 | 0.05320723 | *Pum2* | 1.15517037 | 0.00719692 |
| *Fem1b* | 1.8105222 | 0.00750401 | *Il17ra* | 1.5474878 | 0.05320723 | *Smarce1* | 1.1324503 | 0.03729156 |
| *Emg1* | 1.8105222 | 0.01471662 | *Egfros* | 1.5474878 | 0.05320723 | *Mcf2l* | 1.10002882 | 0.02873051 |
| *Crip1* | 1.8105222 | 0.01471662 | *Tmem170b* | 1.5474878 | 0.05320723 | *Stk40* | 1.10002882 | 0.05387505 |
| *Dnlz* | 1.76988022 | 0.04416299 | *Rrp1b* | 1.5474878 | 0.05320723 | *Bckdhb* | 1.08805618 | 0.0360195 |
| *Rabepk* | 1.76988022 | 0.04416299 | *Dusp12* | 1.5474878 | 0.05359316 | *Ift52* | 1.07800251 | 0.03868596 |
| *Ypel4* | 1.76988022 | 0.04416299 | *Cox6b1* | 1.49090427 | 0.00883687 | *Bcar1* | 1.07800251 | 0.03868596 |
| *Psmc3* | 1.76988022 | 0.04416299 | *Entrep1* | 1.4803736 | 0.0095197 | *Atp1b1* | 1.06206097 | 0.02339761 |
| *Pex16* | 1.76988022 | 0.04416299 | *Sirpa* | 1.4803736 | 0.02661656 | *Trnp1* | 1.06206097 | 0.03944328 |
| *Uckl1* | 1.76988022 | 0.04416299 | *Syap1* | 1.47348721 | 0.03085308 | *B4galt5* | 1.06206097 | 0.05229324 |
| *E2f5* | 1.76988022 | 0.04416299 | *Pgls* | 1.46502564 | 0.03380626 | *Sgms2* | 1.05734884 | 0.0033447 |
| *Kcnc4* | 1.76988022 | 0.04416299 | *Wwp2* | 1.45437839 | 0.01863657 | *Xpo6* | 1.03291462 | 0.03731133 |
| *P2rx4* | 1.76988022 | 0.04416299 | *Spindoc* | 1.45437839 | 0.03288853 | *Tm2d1* | 1.03291462 | 0.03905839 |
| *Ppp2r3d* | 1.76988022 | 0.04416299 | *Nmd3* | 1.44057259 | 0.01798513 | *Bhlhe41* | 1 | 0.03331179 |
| *Pwwp2a* | 1.76988022 | 0.04416299 | *Rassf1* | 1.44057259 | 0.01874635 | *Mtcl1* | 0.98358691 | 0.02285492 |
| *Camta2* | 1.76988022 | 0.04416299 | *Tmem107* | 1.44057259 | 0.03359166 | *Fbxo11* | 0.97633109 | 0.04043869 |
| *Siah3* | 1.76988022 | 0.04416299 | *Socs7* | 1.44057259 | 0.03359166 | *Tacc2* | 0.97375255 | 0.04037804 |
| *Lbh* | 1.76988022 | 0.04416299 | *Eif2a* | 1.42195691 | 0.01345072 | *Spata13* | 0.96252529 | 0.02877474 |
| *Azin1* | 1.7174128 | 0.01662198 | *Aamp* | 1.42195691 | 0.03152639 | *Hdx* | 0.96252529 | 0.03759247 |
| *Peg10* | 1.7174128 | 0.02625699 | *Golph3l* | 1.3954847 | 0.02801506 | *Cmtr1* | 0.96252529 | 0.05441723 |
| *Frmd6* | 1.7174128 | 0.02625699 | *Ankrd50* | 1.3954847 | 0.05486282 | *Btbd8* | 0.93980522 | 0.03706175 |
| *Rnf213* | 1.7174128 | 0.03055881 | *Nomo1* | 1.3954847 | 0.05486282 | *Sumo1* | 0.92935843 | 0.01019237 |
| *Ivns1abp* | 1.7174128 | 0.05187089 | *Arfgef3* | 1.38702312 | 0.01581844 | *Sirt2* | 0.92599942 | 0.03917498 |
| *Alkbh3* | 1.7174128 | 0.05187089 | *Dnm1l* | 1.37061003 | 0.02934489 | *Rgs9* | 0.92599942 | 0.04297265 |
| *Pofut1* | 1.7174128 | 0.05187089 | *Ap2a2* | 1.35484272 | 0.03211917 | *Wasl* | 0.91005787 | 0.01845436 |
| *Yars* | 1.7174128 | 0.05187089 | *Zfp451* | 1.35484272 | 0.03433895 | *Srpk2* | 0.91005787 | 0.0331615 |
| *Vac14* | 1.7174128 | 0.05187089 | *Slc25a46* | 1.35484272 | 0.04658858 | *Copb2* | 0.88452278 | 0.02200256 |
| *C1qbp* | 1.7174128 | 0.05187089 | *Ttll7* | 1.32509537 | 0.00839059 | *Golm2* | 0.88006313 | 0.05343854 |
| *Mipep* | 1.7174128 | 0.05187089 | *Cramp1* | 1.32509537 | 0.01864383 | *Btg1* | 0.8786937 | 0.02429684 |
| *Fam131a* | 1.7174128 | 0.05187089 | *Snhg14* | 1.32509537 | 0.03439661 | *Tra2b* | 0.86941589 | 0.04027276 |
| *Ube4a* | 1.69949089 | 0.00488429 | *Rad54l2* | 1.32509537 | 0.03439661 | *Rpl37* | 0.83699441 | 0.01865515 |
| *Hapstr1* | 1.69949089 | 0.05066091 | *Heatr5a* | 1.32509537 | 0.03517688 | *Efr3a* | 0.82312224 | 0.02326235 |
| *Sv2b* | 1.69949089 | 0.05550933 | *Rnf11* | 1.31800595 | 0.03107128 | *Fat3* | 0.79141338 | 0.0423453 |
| *Dusp14* | 1.68499132 | 0.01527919 | *Ccz1* | 1.31302254 | 0.01921845 | *Foxo1* | 0.78849589 | 0.02827345 |

| **Supplemental Table S15C DEGS Corticotrope Cluster. VCD/Vehicle.** Maroon=upregulated; Blue=downregulated | | | | | | | | |
| --- | --- | --- | --- | --- | --- | --- | --- | --- |
| **gene** | **avg_log2FC** | **p_val** | **gene** | **avg_log2FC** | **p_val** | **gene** | **avg_log2FC** | **p_val** |
| *Scfd2* | 0.78195305 | 0.0063979 | *Crb1* | -1.0374747 | 0.05310854 | *Mrps7* | -1.6749046 | 0.03741877 |
| *Hivep2* | 0.78195305 | 0.03461808 | *Ctnna1* | -1.0847804 | 0.04468571 | *Rfc2* | -1.6749046 | 0.05453386 |
| *Fkbp2* | 0.77881334 | 0.03471447 | *Tm9sf3* | -1.1045889 | 0.0139579 | *Btbd1* | -1.6895514 | 0.01546908 |
| *Otud7a* | 0.77696964 | 0.02576894 | *Tifa* | -1.1154772 | 0.05057509 | *Mydgf* | -1.7004397 | 0.01993213 |
| *Capn15* | 0.76988022 | 0.04223197 | *Chd3* | -1.1405682 | 0.04275449 | *Ifitm2* | -1.7004397 | 0.04103085 |
| *Rps28* | 0.76988022 | 0.04294752 | *Itpr2* | -1.1476576 | 0.00330448 | *Usp33* | -1.7744403 | 0.00167087 |
| *Pcf11* | 0.7616126 | 0.0515621 | *Mef2a* | -1.1577689 | 0.00195775 | *Uhrf2* | -1.7744403 | 0.0402676 |
| *Malt1* | 0.74388501 | 0.04185216 | *Atf7* | -1.1687192 | 0.02458724 | *Nono* | -1.7744403 | 0.04180152 |
| *Sod1* | 0.74013287 | 0.03922221 | *Sfi1* | -1.1687192 | 0.03654254 | *Mrto4* | -1.7744403 | 0.04383694 |
| *Ypel2* | 0.72969113 | 0.02685011 | *Plekhb2* | -1.1687192 | 0.04998154 | *Mgmt* | -1.7744403 | 0.04383694 |
| *Hnrnph3* | 0.72436556 | 0.05404826 | *Phyh* | -1.1894778 | 0.03558906 | *Tbc1d9* | -1.7744403 | 0.04383694 |
| *Rplp1* | 0.71098653 | 0.00223147 | *Rrp7a* | -1.1894778 | 0.04709736 | *Calml4* | -1.7744403 | 0.04383694 |
| *Fkbp5* | 0.70618554 | 0.02094152 | *Gria4* | -1.218047 | 0.04677902 | *Taf2* | -1.7744403 | 0.04383694 |
| *Rps6ka3* | 0.69432918 | 0.05372004 | *Pcbp1* | -1.2367835 | 0.02616698 | *Lpgat1* | -1.7744403 | 0.05380428 |
| *Gas5* | 0.68075433 | 0.03852543 | *Pan3* | -1.2439256 | 0.0092342 | *Mrps26* | -1.7744403 | 0.05453386 |
| *Smg6* | 0.67581189 | 0.04375436 | *Taf3* | -1.2598671 | 0.01765683 | *6430548M08Rik* | -1.8448296 | 0.0286804 |
| *Adcy2* | 0.66823995 | 0.00493381 | *Ube2w* | -1.2598671 | 0.02486014 | *Map3k8* | -1.8675497 | 0.03641371 |
| *Sbf2* | 0.66613229 | 0.02652723 | *Pgap2* | -1.2598671 | 0.034781 | *Ascc2* | -1.9119438 | 0.02903602 |
| *Ptpn1* | 0.66296501 | 0.05141214 | *Csad* | -1.2598671 | 0.04079497 | *Phyhd1* | -1.9119438 | 0.04340689 |
| *Tbx19* | 0.65245735 | 0.03633573 | *Zdhhc13* | -1.3005091 | 0.03268488 | *Sumf2* | -1.9119438 | 0.04340689 |
| *Dclk2* | 0.64702347 | 0.03448222 | *Fam114a2* | -1.3104932 | 0.04281937 | *Nol11* | -1.9119438 | 0.04340689 |
| *Rps21* | 0.63584367 | 0.0275961 | *Efl1* | -1.3269813 | 0.03146848 | *Yipf5* | -1.9550125 | 0.03691562 |
| *Elapor1* | 0.62196601 | 0.02620988 | *Krtcap2* | -1.3269813 | 0.04641499 | *Myo1b* | -2.0374747 | 0.02872624 |
| *Rps15a* | 0.61460199 | 0.0441376 | *Stx8* | -1.3594028 | 0.04282074 | *Stk4* | -2.0374747 | 0.02872624 |
| *Mast4* | 0.60520329 | 0.03660394 | *Coq7* | -1.3594028 | 0.04446073 | *Slbp* | -2.0374747 | 0.02872624 |
|  |  |  | *Actr1a* | -1.3594028 | 0.04446073 | *Zcchc8* | -2.0374747 | 0.02872624 |
| *Rps3* | -0.5520479 | 0.03850424 | *Chn1* | -1.3911117 | 0.02181981 | *Tnfaip1* | -2.0374747 | 0.02872624 |
| *Fhod3* | -0.5768403 | 0.03557224 | *Dnajc10* | -1.3911117 | 0.04778419 | *Casr* | -2.0770031 | 0.00211913 |
| *Pid1* | -0.5849625 | 0.05555814 | *Olfm3* | -1.4244978 | 0.01031847 | *Arhgap39* | -2.1154772 | 0.00338703 |
| *Ubb* | -0.685173 | 0.05178598 | *E230001N04Rik* | -1.4525122 | 0.0115983 | *Msh3* | -2.1529519 | 0.01898549 |
| *Fut8* | -0.7004397 | 0.0439209 | *Itprid2* | -1.4525122 | 0.01682242 | *Rubcn* | -2.1529519 | 0.01898549 |
| *Rorb* | -0.7019885 | 0.0552602 | *Jkamp* | -1.4525122 | 0.0312301 | *Gm7967* | -2.1529519 | 0.02850615 |
| *Bex3* | -0.7420188 | 0.04552258 | *Med1* | -1.4525122 | 0.03205969 | *Gng10* | -2.2073997 | 0.00170474 |
| *Cdc42bpa* | -0.7462434 | 0.05168149 | *Cops8* | -1.4525122 | 0.04336575 | *Ccl25* | -2.2598671 | 0.00836729 |
| *Nucks1* | -0.7479681 | 0.03175008 | *Ppp4r3b* | -1.4525122 | 0.04399763 | *Kif3c* | -2.2598671 | 0.01252566 |
| *Cop1* | -0.7520725 | 0.04712582 | *Nova1* | -1.4525122 | 0.04496246 | *Slain2* | -2.2598671 | 0.01884123 |
| *Ncor1* | -0.7531717 | 0.01420562 | *Il1rap* | -1.4525122 | 0.04496246 | *Dlg3* | -2.2598671 | 0.01884123 |
| *Clta* | -0.8061492 | 0.02340135 | *Lrrtm4* | -1.4525122 | 0.04563026 | *Ampd2* | -2.2598671 | 0.04051341 |
| *mt-Nd5* | -0.8358408 | 0.00298475 | *Cpd* | -1.5114059 | 0.03299376 | *Gm11817* | -2.2598671 | 0.04051341 |
| *Cdc73* | -0.8675497 | 0.04681532 | *Xrcc5* | -1.539975 | 0.02977505 | *Klf3* | -2.2598671 | 0.04051341 |
| *R3hdm2* | -0.8675497 | 0.05381468 | *Rint1* | -1.539975 | 0.02977505 | *Vhl* | -2.2598671 | 0.04051341 |
| *Pbx3* | -0.8854716 | 0.03609163 | *Gm20275* | -1.539975 | 0.04258031 | *Ptcd2* | -2.2598671 | 0.04051341 |
| *Acaca* | -0.8930848 | 0.02510008 | *Nudc* | -1.5520479 | 0.00814485 | *Apex1* | -2.2598671 | 0.04051341 |
| *Wdr1* | -0.8930848 | 0.04473317 | *Ppp4r1l-ps* | -1.5679894 | 0.01123963 | *Gm32618* | -2.2598671 | 0.04051341 |
| *Brk1* | -0.8999712 | 0.03968815 | *Thap3* | -1.5679894 | 0.02084243 | *Rab31* | -2.2598671 | 0.04051341 |
| *Cracdl* | -0.9119438 | 0.02250832 | *Brix1* | -1.5679894 | 0.02084243 | *Mir133a-1hg* | -2.2598671 | 0.04051341 |
| *Nf1* | -0.9428378 | 0.02081072 | *Zranb2* | -1.5679894 | 0.04166994 | *Wnk3* | -2.2598671 | 0.04051341 |
| *Ctnnb1* | -0.9550125 | 0.03553605 | *Mmaa* | -1.5679894 | 0.05526586 | *Lig3* | -2.2598671 | 0.04307313 |
| *Wwc1* | -0.9594722 | 0.04134032 | *Zcchc14* | -1.5679894 | 0.05526586 | *Rbfox3* | -2.4525122 | 0.01233627 |
| *Abat* | -0.9670854 | 0.05032701 | *Cenpx* | -1.5679894 | 0.05526586 | *Ormdl1* | -2.4525122 | 0.02647856 |
| *Smarca1* | -0.9670854 | 0.05405688 | *3830406C13Rik* | -1.5679894 | 0.05526586 | *Gpsm1* | -2.4525122 | 0.02647856 |
| *Mvb12b* | -0.9760742 | 0.02684368 | *Asrgl1* | -1.5679894 | 0.05526586 | *Tmem64* | -2.4525122 | 0.02647856 |
| *Cep112* | -0.9760742 | 0.0551775 | *Tmem47* | -1.5679894 | 0.05526586 | *Gamt* | -2.4525122 | 0.02647856 |
| *Prickle1* | -0.9797592 | 0.03874521 | *Plekha6* | -1.5817952 | 0.01480132 | *Sirt7* | -2.4525122 | 0.02647856 |
| *Cdh18* | -0.9814407 | 0.03785708 | *Supt20* | -1.5900157 | 0.0216618 | *Gm4419* | -2.4525122 | 0.02647856 |
| *Fgd4* | -1 | 0.03663285 | *Lmbr1* | -1.6224372 | 0.01096381 | *Eri2* | -2.4525122 | 0.0404947 |
| *Rack1* | -1.0374747 | 0.00101229 | *Psmd12* | -1.6224372 | 0.02031277 | *Zfp839* | -2.4525122 | 0.0404947 |
| *Gm46367* | -1.0374747 | 0.01778327 | *Hopx* | -1.6749046 | 0.03741877 | *Pitrm1* | -2.4525122 | 0.0404947 |
| *Ryk* | -1.0374747 | 0.05179281 | *Cttn* | -1.6749046 | 0.03741877 | *Pdzd11* | -2.4525122 | 0.0404947 |

| **Supplemental Table S15D DEGS Corticotropes Cluster; VCD/Vehicle.** Blue=downregulated | | | | | | | | |
| --- | --- | --- | --- | --- | --- | --- | --- | --- |
| **gene** | **avg_log2FC** | **p_val** |  |  |  |  |  |  |
| *Nol12* | -2.539975 | 0.0053766 |  |  |  |  |  |  |
| *Tgs1* | -2.6224372 | 0.01735255 |  |  |  |  |  |  |
| *Ccdc15* | -2.6224372 | 0.02645733 |  |  |  |  |  |  |
| *Med4* | -2.6224372 | 0.04050406 |  |  |  |  |  |  |
| *Fem1a* | -2.6224372 | 0.04050406 |  |  |  |  |  |  |
| *Sart1* | -2.6224372 | 0.04050406 |  |  |  |  |  |  |
| *Glod4* | -2.7744403 | 0.01138678 |  |  |  |  |  |  |
| *Nt5c3* | -2.7744403 | 0.02646643 |  |  |  |  |  |  |
| *Mpnd* | -2.7744403 | 0.04050967 |  |  |  |  |  |  |
| *Trmt1l* | -2.9119438 | 0.01733869 |  |  |  |  |  |  |
| *Reck* | -2.9119438 | 0.01733869 |  |  |  |  |  |  |
| *Crnkl1* | -3.0374747 | 0.01734463 |  |  |  |  |  |  |
| *Mgat4a* | -3.1529519 | 0.00745386 |  |  |  |  |  |  |
| *Lrrn2* | -3.2598671 | 0.00319801 |  |  |  |  |  |  |
| *Dynlt2a2* | -3.2598671 | 0.00745999 |  |  |  |  |  |  |
| *Kras* | -3.4525122 | 0.00746476 |  |  |  |  |  |  |

| **Supplemental Table S16. Differentially Expressed Genes Representing Beta-Catenin independent WNT signaling. VCD vs Vehicle Control Females** | | | |
| --- | --- | --- | --- |
|  |  | **Corticotropes** | |
|  | **Gene** | **Expr Log Ratio** | **Expr p-value** |
| adaptor related protein complex 2 subunit alpha 2 | *Ap2a2* | 1.355 | 0.0321 |
| clathrin light chain A | *Clta* | -0.806 | 0.0234 |
| catenin beta 1 | *Ctnnb1* | -0.955 | 0.0355 |
| G protein subunit gamma 10 | *Gng10* | -2.207 | 0.0017 |
| inositol 1,4,5-trisphosphate receptor type 2 | *Itpr2* | -1.148 | 0.0033 |
| KRAS proto-oncogene, GTPase | *Kras* | -3.453 | 0.00746 |
| mitogen-activated protein kinase kinase kinase 7 | *Map3k7* | 1.647 | 0.0311 |
| prickle planar cell polarity protein 1 | *Prickle1* | -0.98 | 0.0387 |
| protein kinase C gamma | *Prkcg* | 2.547 | 0.0358 |
| proteasome 20S subunit beta 4 | *Psmb4* | 1.169 | 0.05 |
| proteasome 26S subunit, ATPase 3 | *Psmc3* | 1.77 | 0.0442 |
| proteasome 26S subunit, non-ATPase 12 | *Psmd12* | -1.622 | 0.0203 |
| receptor like tyrosine kinase | *Ryk* | -1.037 | 0.0518 |
| serine threonine kinase 4 | *Stk4* | -2.037 | 0.0287 |
| serine threonine kinase 40 | *Stk40* | 1.1 | 0.0539 |
| ubiquitin B | *Ubb* | -0.685 | 0.0518 |

| **Supplemental Table S17. Differentially Expressed Genes in Corticotrope TCF dependent signaling in response to WNT. VCD vs Vehicle Control Females** | | | |
| --- | --- | --- | --- |
|  |  | **Corticotropes** | |
|  | **Gene** | **Expr Log Ratio** | **Expr p-value** |
| cell division cycle 73 | Cdc73 | -0.868 | 0.0468 |
| catenin beta 1 | Ctnnb1 | -0.955 | 0.0355 |
| proteasome 20S subunit beta 4 | Psmb4 | 1.169 | 0.05 |
| proteasome 26S subunit, ATPase 3 | Psmc3 | 1.77 | 0.0442 |
| proteasome 26S subunit, non-ATPase 12 | Psmd12 | -1.622 | 0.0203 |
| receptor like tyrosine kinase | Ryk | -1.037 | 0.0518 |
| ubiquitin B | Ubb | -0.685 | 0.0518 |

| **Supplemental Table S18. Differentially Expressed Genes in Corticotropes Representing Protein Kinase A Signaling. VCD vs Vehicle Control Females** | | | |
| --- | --- | --- | --- |
|  |  | **Corticotropes** | |
|  | **Gene** | **Expr Log Ratio** | **Expr p-value** |
| adenylate cyclase 2 | Adcy2 | 0.668 | 0.00493 |
| anaphase promoting complex subunit 13 | Anapc13 | 1.663 | 0.0155 |
| apurinicapyrimidinic endodeoxyribonuclease 1 | Apex1 | -2.26 | 0.0405 |
| activating transcription factor 1 | Atf1 | 1.547 | 0.032 |
| cell division cycle 27 | Cdc27 | 1.869 | 0.0159 |
| catenin beta 1 | Ctnnb1 | -0.955 | 0.0355 |
| dual specificity phosphatase 12 | Dusp12 | 1.547 | 0.0536 |
| G protein subunit gamma 10 | Gng10 | -2.207 | 0.0017 |
| inositol 1,4,5-trisphosphate receptor type 2 | Itpr2 | -1.148 | 0.0033 |
| metallophosphoesterase 1 | Mppe1 | 2.547 | 0.0358 |
| protein phosphatase 1 catalytic subunit beta | Ppp1cb | 1.206 | 0.00804 |
| protein kinase C gamma | Prkcg | 2.547 | 0.0358 |
| protein tyrosine phosphatase non-receptor type 1 | Ptpn1 | 0.663 | 0.0514 |
| glycogen phosphorylase, muscle associated | Pygm | 3.132 | 0.015 |
| signal regulatory protein alpha | Sirpa | 1.48 | 0.0266 |

| **Supplemental Table S19. Differentially Expressed Genes in Corticotropes Representing JAK/STAT signaling. VCD vs Vehicle Control Females** | | | |
| --- | --- | --- | --- |
|  |  | **Corticotropes** | |
|  | **Gene** | **Expr Log Ratio** | **Expr p-value** |
| KRAS proto-oncogene, GTPase | *Kras* | -3.453 | 0.00746 |
| NRAS proto-oncogene, GTPase | *Nras* | 2.355 | 0.0203 |
| protein tyrosine phosphatase non-receptor type 1 | *Ptpn1* | 0.663 | 0.0514 |
| suppressor of cytokine signaling 7 | *Socs7* | 1.441 | 0.0336 |
|  |  |  |  |

| **Supplemental Table S20A Differentially Expressed 17-β Estradiol Target Genes**  **Comparing Corticotropes and Thyrotropes  VCD vs Vehicle control females** | | | | | |
| --- | --- | --- | --- | --- | --- |
|  |  | **Corticotropes** | | **Thyrotropes** | |
|  | **Gene** | **Expr Log Ratio** | **Expr p-value** | **Expr Log Ratio** | **Expr p-value** |
| 4-aminobutyrate aminotransferase | *Abat* | -0.967 | 0.0503 |  |  |
| acyl-CoA synthetase short chain family member 1 | *Acss1* | 2.547 | 0.0358 |  |  |
| ALG5 dolichyl-phosphate beta-glucosyltransferase | *Alg5* | 3.132 | 0.00639 |  |  |
| annexin A4 | *Anxa4* | 2.284 | 0.00434 |  |  |
| apurinic/apyrimidinic endodeoxyribonuclease 1 | *Apex1* | -2.26 | 0.0405 |  |  |
| Rho/Rac guanine nucleotide exchange factor 2 | *Arhgef2* | 1.248 | 0.0202 |  |  |
| ATPase Na+/K+ transporting subunit beta 1 | *Atp1b1* | 1.062 | 0.0234 |  |  |
| activating transcription factor 3 | *Atf3* |  |  | 2.392 | 0.00497 |
| ATPase H+ transporting accessory protein 1 | *Atp6ap1* |  |  | 2.17 | 0.0233 |
| BTG anti-proliferation factor 1 | *Btg1* |  |  | 1.7 | 0.02 |
| beta-1,4-galactosyltransferase 5 | *B4galt5* | 1.062 | 0.0523 |  |  |
| BCAR1 scaffold protein, Cas family member | *Bcar1* | 1.078 | 0.0387 |  |  |
| brain expressed X-linked 3 | *Bex3* | -0.742 | 0.0455 |  |  |
| BTG anti-proliferation factor 1 | *Btg1* | 0.879 | 0.0243 | 1.7 | 0.0231 |
| BUB3 mitotic checkpoint protein | *Bub3* | 2.007 | 0.0123 |  |  |
| beta-1,4-galactosyltransferase 5 | *B4galt5* | 1.062 | 0.0523 |  |  |
| creatine kinase B | *Ckb* |  |  | 3.585 | 0.00265 |
| citramalyl-CoA lyase | *Clybl* |  |  | -2.415 | 0.0163 |
| cullin 4A | *Cul4a* |  |  | 2.7 | 0.00545 |
| cytochrome b5 type A | *Cyb5a* |  |  | 2.459 | 0.022 |
| cysteine rich transmembrane module containing 1 | *Cystm1* |  |  | 1.354 | 0.0433 |
| complement C1q binding protein | *C1qbp* | 1.717 | 0.0519 |  |  |
| calcium voltage-gated channel subunit alpha1 G | *Cacna1g* | 2.355 | 0.0309 |  |  |
| calmodulin like 4 | *Calml4* | -1.774 | 0.0438 |  |  |
| C-C motif chemokine ligand 25 | *Ccl25* | -2.26 | 0.00837 |  |  |
| CD74 molecule | *Cd74* | 2.547 | 0.0358 |  |  |
| CDC42 binding protein kinase alpha | *Cdc42bpa* | -0.746 | 0.0517 |  |  |
| cell division cycle 73 | *Cdc73* | -0.868 | 0.0468 |  |  |
| crooked neck pre-mRNA splicing factor 1 | *Crnkl1* | -3.037 | 0.0173 |  |  |
| chromosome segregation 1 like | *Cse1l* | 1.926 | 0.0277 |  |  |
| catenin alpha 1 | *Ctnna1* | -1.085 | 0.0447 |  |  |
| catenin beta 1 | *Ctnnb1* | -0.955 | 0.0355 |  |  |
| cortactin | *Cttn* | -1.675 | 0.0374 |  |  |
| creatine kinase B | *Ckb* |  |  | 3.585 | 0.00265 |
| citramalyl-CoA lyase | *Clybl* |  |  | -2.415 | 0.0163 |
| cullin 4A | *Cul4a* |  |  | 2.7 | 0.00545 |
| cytochrome c, somatic | *Cycs* | 1.547 | 0.0304 |  |  |
| developmentally regulated GTP binding protein 1 | *Drg1* |  |  | -2.17 | 0.0233 |
| dedicator of cytokinesis 1 | *Dock1* |  |  | -1.144 | 0.0233 |
| developmentally regulated GTP binding protein 1 | *Drg1* |  |  | -2.17 | 0.0233 |
| discs large MAGUK scaffold protein 3 | *Dlg3* | -2.26 | 0.0188 |  |  |
| ETS variant transcription factor 1 | *Etv1* |  |  | -3.17 | 0.0417 |
| ETS transcription factor ELK3 | *Elk3* | 2.132 | 0.0144 |  |  |
| EMG1 N1-specific pseudouridine methyltransferase | *Emg1* | 1.811 | 0.0147 |  |  |
| estrogen related receptor alpha | *Esrra* | 2.869 | 0.015 |  |  |
| FERM, ARH/RhoGEF and pleckstrin domain protein 1 | *Farp1* |  |  | 0.603 | 0.047 |
| FYN proto-oncogene, Src family tyrosine kinase | *Fyn* |  |  | -1.7 | 0.0437 |
| phenylalanyl-tRNA synthetase subunit alpha | *Farsa* | 3.547 | 0.00639 |  |  |
| fasciculation and elongation protein zeta 2 | *Fez2* | 2.547 | 0.00604 |  |  |
| fibronectin leucine rich transmembrane protein 3 | *Flrt3* | 2.132 | 0.0202 |  |  |
| folate receptor alpha | *Folr1* | 2.547 | 0.0358 |  |  |
| FOS like 1, AP-1 transcription factor subunit | *Fosl1* | 1.547 | 0.0458 |  |  |
| forkhead box O1 | *Foxo1* | 0.788 | 0.0283 |  |  |
| fucosyltransferase 8 | *Fut8* | -0.7 | 0.0439 |  |  |
| **glucose-6-phosphate dehydrogenase** | ***G6pdx*** | **2.132** | **0.0313** | **2.807** | **0.0417** |
| growth arrest specific 5 | *Gas5* | 0.681 | 0.0385 |  |  |
| GTP cyclohydrolase 1 | *Gch1* | 3.132 | 0.015 |  |  |

| **Supplemental Table S20B Differentially Expressed 17-β Estradiol Target Genes**  **Comparing Corticotropes and Thyrotropes  VCD vs Vehicle control females** | | | | | |
| --- | --- | --- | --- | --- | --- |
|  |  | **Corticotropes** | | **Thyrotropes** | |
|  | **Gene** | **Expr Log Ratio** | **Expr p-value** | **Expr Log Ratio** | **Expr p-value** |
| glucose-6-phosphate dehydrogenase | *G6pdx* |  |  | 2.807 | 0.0417 |
| G protein subunit alpha 11 | *Gna11* |  |  | 2 | 0.0455 |
| G protein subunit beta 1 | *Gnb1* |  |  | 1.663 | 0.0493 |
| G protein-coupled receptor kinase 3 | *Grk3* |  |  | 2.807 | 0.0417 |
| glutamate metabotropic receptor 5 | *Grm5* |  |  | -3.807 | 0.0213 |
| HIVEP zinc finger 2 | *Hivep2* | 0.782 | 0.0346 |  |  |
| harakiri, BCL2 interacting protein | *Hrk* | 2.547 | 0.0358 |  |  |
| 3-hydroxy-3-methylglutaryl-CoA synthase 1 | *Hmgcs1* |  |  | 2.459 | 0.022 |
| heterogeneous nuclear ribonucleoprotein A1 | *Hnrnpa1* |  |  | 2 | 0.0455 |
| heat shock protein family A (Hsp70) member 9 | *Hspa9* |  |  | 2.322 | 0.0428 |
| insulin like growth factor 2 receptor | *Igf2r* |  |  | 2.585 | 0.0416 |
| interleukin 17 receptor D | *Il17rd* |  |  | 2.322 | 0.0417 |
| inositol polyphosphate-5-phosphatase F | *Inpp5f* |  |  | 2.585 | 0.0213 |
| integrin subunit alpha 3 | *Itga3* |  |  | 2.585 | 0.0416 |
| interleukin 1 receptor accessory protein | *Il1rap* | -1.453 | 0.045 |  |  |
| Janus kinase 2 | *Jak2* |  |  | 3 | 0.0417 |
| kazrin, periplakin interacting protein | *Kazn* |  |  | 3 | 0.00247 |
| low density lipoprotein receptor | *Ldr* |  |  | 2 | 0.0455 |
| leucine rich repeat containing G protein-coupled receptor 4 | *Lgr4* |  |  | 2.222 | 0.0173 |
| mitogen-activated protein kinase kinase kinase 7 | *Map3k7* | 1.647 | 0.0311 |  |  |
| mitogen-activated protein kinase kinase kinase 8 | *Map3k8* | -1.868 | 0.0364 |  |  |
| mitogen-activated protein kinase 14 | *Mapk14* | 1.821 | 0.00247 |  |  |
| myocyte enhancer factor 2A | *Mef2a* | -1.158 | 0.00196 |  |  |
| mitochondrial ribosomal protein S12 | *Mrps12* | 2.132 | 0.0202 |  |  |
| mitochondrial ribosomal protein S26 | *Mrps26* | -1.774 | 0.0545 |  |  |
| matrix remodeling associated 7 | *Mxra7* | 2.547 | 0.0358 |  |  |
| myosin IB | *Myo1b* | -2.037 | 0.0287 |  |  |
| nuclear receptor corepressor 1 | *Ncor1* | -0.753 | 0.0142 |  |  |
| neural precursor cell expressed, developmentally down-regulated 9 | *Nedd9* | 2.717 | 0.0302 |  |  |
| nuclear transcription factor Y subunit alpha | *Nfya* | 3.132 | 0.0358 |  |  |
| nuclear receptor interacting protein 3 | *Nrip3* | 2.869 | 0.0302 |  |  |
| NADH: ubiquinone oxidoreductase subunit B11 | *Ndufb11* |  |  | 1.322 | 0.054 |
| oxoglutarate dehydrogenase | *Ogdh* |  |  | 1.415 | 0.0218 |
| purinergic receptor P2X 4 | *P2rx4* | 1.77 | 0.0442 |  |  |
| paternally expressed 10 | *Peg10* | 1.717 | 0.0263 |  |  |
| pleckstrin homology domain containing A6 | *Plekha6* | -1.582 | 0.0148 |  |  |
| phosphodiesterase 4B | *Pde4b* |  |  | 2.807 | 0.0417 |
| protein disulfide isomerase family A member 3 | *Pdia3* |  |  | 1.433 | 0.016 |
| phosphoglycerate kinase 1 | *Pgk1* |  |  | 2.322 | 0.0417 |
| PHD finger protein 21B | *Phf21b* |  |  | 3.322 | 0.00538 |
| paraoxonase 2 | *Pon2* |  |  | 2.807 | 0.0416 |
| protein kinase C beta | *Prkcb* |  |  | -1.766 | 0.0431 |
| protein phosphatase 1 catalytic subunit beta | *Ppp1cb* | 1.206 | 0.00804 |  |  |
| prickle planar cell polarity protein 1 | *Prickle1* | -0.98 | 0.0387 |  |  |
| proteasome 26S subunit, ATPase 3 | *Psmc3* | 1.77 | 0.0442 |  |  |
| protein tyrosine phosphatase non-receptor type 1 | *Ptpn1* | 0.663 | 0.0514 |  |  |
| pyroglutamylated RFamide peptide receptor | *Qrfpr* | 2.547 | 0.0358 |  |  |
| prolactin | *Prl* |  |  | -1.256 | 0.0222 |
| prosaposin | *Psap* |  |  | 2.115 | 0.00395 |
| protein tyrosine phosphatase non-receptor type 11 | *Ptpn11* |  |  | 2.807 | 0.0212 |
| RAN, member RAS oncogene family | *Ran* |  |  | 3 | 0.0107 |
| RAB31, member RAS oncogene family | *Rab31* | -2.26 | 0.0405 |  |  |
| receptor for activated C kinase 1 | *Rack1* | -1.037 | 0.00101 |  |  |
| regulator of G protein signaling 9 | *Rgs9* | 0.926 | 0.043 |  |  |
| regulator of calcineurin 1 | *Rcan1* |  |  | 3 | 0.0213 |
| semaphorin 3A | *Sema3a* |  |  | -3 | 0.0213 |

| **Supplemental Table S20C Differentially Expressed 17-β Estradiol Target Genes**  **Comparing Corticotropes and Thyrotropes  VCD vs Vehicle control females** | | | | | |
| --- | --- | --- | --- | --- | --- |
|  |  | **Corticotropes** | | **Thyrotropes** | |
|  | **Gene** | **Expr Log Ratio** | **Expr p-value** | **Expr Log Ratio** | **Expr p-value** |
| SMAD family member 2 | *Smad2* |  |  | -2.17 | 0.0441 |
| signal sequence receptor subunit 3 | *Ser* |  |  | 1.807 | 0.0475 |
| ST6 beta-galactoside alpha-2,6-sialyltransferase 1 | *St6gal1* |  |  | 3 | 0.0417 |
| serine/threonine kinase 39 | *Stk39* |  |  | 1.874 | 0.0372 |
| Sin3A associated protein 30 | *Sap30* | 2.547 | 0.0358 |  |  |
| Scm polycomb group protein like 4 | *Scml4* | 1.306 | 0.0278 |  |  |
| seizure related 6 homolog | *Sez6* | 2.547 | 0.0358 |  |  |
| salt inducible kinase 1 | *Sik1* | 2.313 | 0.00631 |  |  |
| superoxide dismutase 1 | *Sod1* | 0.74 | 0.0392 |  |  |
| sortilin related VPS10 domain containing receptor 3 | *Sorcs3* | 2.547 | 0.0358 |  |  |
| synaptopodin | *Synpo* | 1.164 | 0.0176 |  |  |
| synaptotagmin 11 | *Syt11* | 1.248 | 0.0202 |  |  |
| TBC1 domain family member 9 | *Tbc1d9* | -1.774 | 0.0438 |  |  |
| tight junction protein 2 | *Tjp2* | 1.926 | 0.00125 |  |  |
| toll like receptor 4 | *Tlr4* | 2.869 | 0.0358 |  |  |
| transformer 2 beta homolog | *Tra2b* | 0.869 | 0.0403 | 1.585 | 0.0317 |
| transmembrane protein 245 | *Tmem245* |  |  | -2.585 | 0.0416 |
| tumor protein p53 | *Trp53* |  |  | 2.322 | 0.0417 |
| translocated promoter region, nuclear basket protein | *Tpr* |  |  | 1.459 | 0.045 |
| ubiquitin conjugating enzyme E2 B | *Ube2b* |  |  | 0.893 | 0.0207 |
| von Hippel-Lindau tumor suppressor | *Vhl* | -2.26 | 0.0405 |  |  |
| WW and C2 domain containing 1 | *Wwc1* | -0.959 | 0.0413 |  |  |
| Wnt ligand secretion mediator | *Wls* |  |  | 2.7 | 0.0227 |
| zinc finger E-box binding homeobox 2 | *Zeb2* |  |  | -2.585 | 0.0416 |
| zinc finger SWIM-type containing 6 | *Zswim6* |  |  | 3.17 | 0.00534 |
| zinc finger and BTB domain containing 16 | *Zbtb16* | 1.298 | 0.00456 |  |  |
|  |  |  |  |  |  |
